# Supplementary material for: Deletion of EP3 prostaglandin receptor in murine macrophages aggravates diet-induced obesity by suppressing SPARC
Source: EMBO J. 2025 Jul 23;44(18):4962–83. doi: 10.1038/s44318-025-00508-y (PMC12436609; doi:10.1038/s44318-025-00508-y)
Supplement: Supplementary file 1 — Appendix [file 44318_2025_508_MOESM1_ESM.pdf]

Appendix

Deletion of EP3 prostaglandin receptor in murine macrophages aggravates diet-induced obesity by suppressing SPARC

Wenlong Shang[#1], Yinxiu Li[#1], Lu Wang[2], Jiao Liu[1], Huiwen Ren[1], Qian Liu[1], Shumin Guo[1], Yuhong Wang[1], Yubo Ma[1], Tianyi You[2], Yujun Shen[1], Yu Zhou[3], Danyang Tian*[1, 4], Ying Yu*[1]

Table of contents

Appendix Figure S1……page 2
Appendix Figure S2……page 3
Appendix Figure S3……page 4
Appendix Figure S4……page 5-6
Appendix Figure S5……page 7
Appendix Figure S6……page 8-9
Appendix Figure S7……page 10
Appendix Figure S8……page 11
Appendix Figure S9……page 12
Appendix Figure S10…… page 13
Appendix Figure S11…… page 14
Appendix Figure S12…… page 15
Appendix Table S1…… page 16
Appendix Table S2…… page 17-18
Appendix Table S3…… page 19

Appendix Figure S1

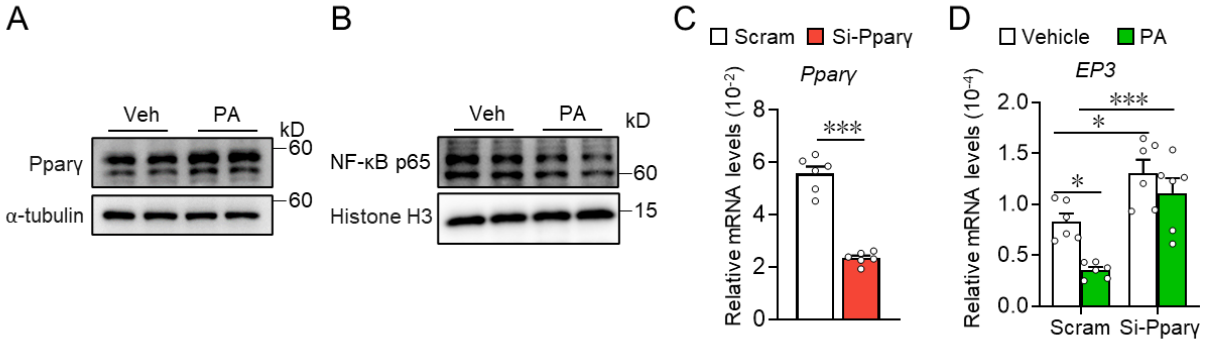

Appendix Figure S1. PA suppresses EP3 expression in BMDMs via Pparγ/ NF-κB pathway.
(**A**), western blot analysis of Pparγ in PA-treated BMDMs.
(**B**), western blot analysis of nuclear NF-κB p65 expression in BMDMs after PA treatment for 24h.
(**C**), Pparγ siRNA knockdown efficiency in BMDMs (n=6).
(**D**), qRT-PCR analysis of the relative mRNA levels of *EP3* in BMDMs with PA treatment after Pparγ siRNA transfection (n=6).
Data information: Data represent the mean ± SEM. Statistics: Unpaired Student's t-test (**C**), Two-way ANOVA (**D**). **C-D**, P–values are indicated by asterisks, with *P<0.05, ***P<0.001.

Appendix Figure S2

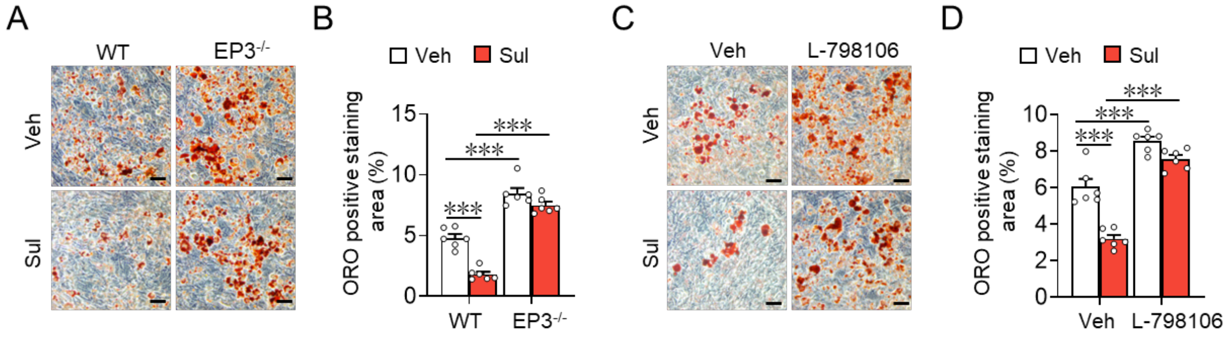

Appendix Figure S2. PGE$_2$/EP3 axis inhibits adipogenesis.

(**A**), Representative images of Oil Red O staining of differentiated 3T3-L1 cells cocultured with WT and EP3$^{-/-}$ BMDMs with and without sulprostone treatment. (Scale bar: 50 μm).

(**B**), Quantification of Oil Red O staining in (**A**) (n=6).

(**C**), Effect of L-798106 on adipocyte differentiation of 3T3-L1 cells cocultured with sulprostone-treated BMDMs. (Scale bar: 50 μm).

(**D**), Quantification of Oil Red O staining in (**C**) (n=6).

Data information: Data represent the mean ± SEM. Statistics: Two-way ANOVA (**B**, **D**). **B**, **D**, P–values are indicated by asterisks, with ***P<0.001.

Appendix Figure S3

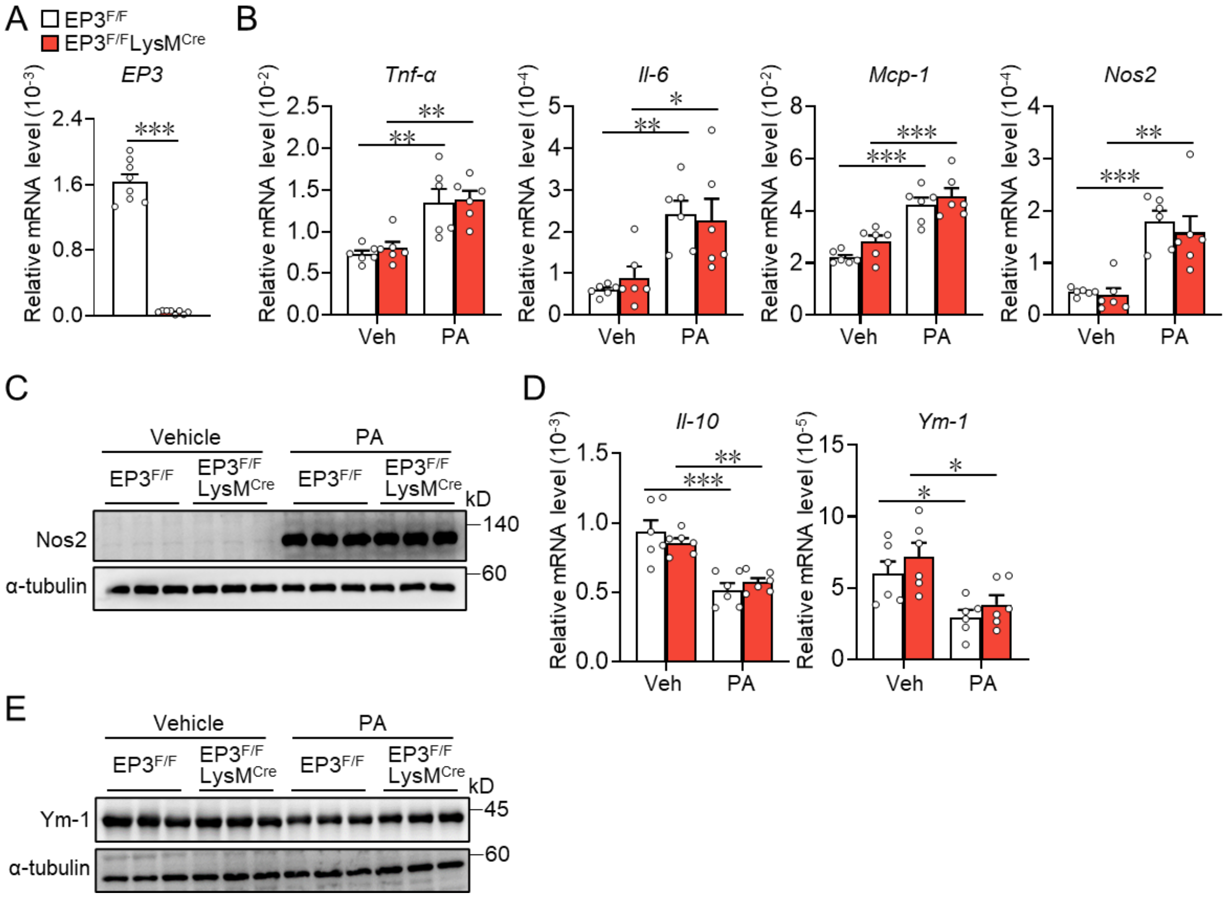

Appendix Figure S3. EP3 deletion does not influence macrophage polarization.

(**A**), qRT-PCR analysis of the relative mRNA levels of *EP3* in BMDMs from EP3$^{F/F}$ and EP3$^{F/F}$LysM^{Cre} mice (n=8).

(**B**), qRT-PCR analysis of the relative mRNA levels of pro-inflammatory genes in PA-treated BMDMs from EP3$^{F/F}$ and EP3$^{F/F}$LysM^{Cre} mice (n=6).

(**C**), western blot analysis of Nos2 in PA-treated BMDMs from EP3$^{F/F}$ and EP3$^{F/F}$LysM^{Cre} mice.

(**D**), qRT-PCR analysis of the relative mRNA levels of anti-inflammatory genes in PA-treated BMDMs from EP3$^{F/F}$ and EP3$^{F/F}$LysM^{Cre} mice (n=6).

(**E**), western blot analysis of Ym-1 in PA-treated BMDMs from EP3$^{F/F}$ and EP3$^{F/F}$LysM^{Cre} mice.

Data information: Data represent the mean ± SEM. Statistics: Mann-Whitney U-test (**A**), Two-way ANOVA (**B**, **D**). **A**–**B**, **D**, P–values are indicated by asterisks, with *P<0.05, **P<0.01, ***P<0.001.

Appendix Figure S4

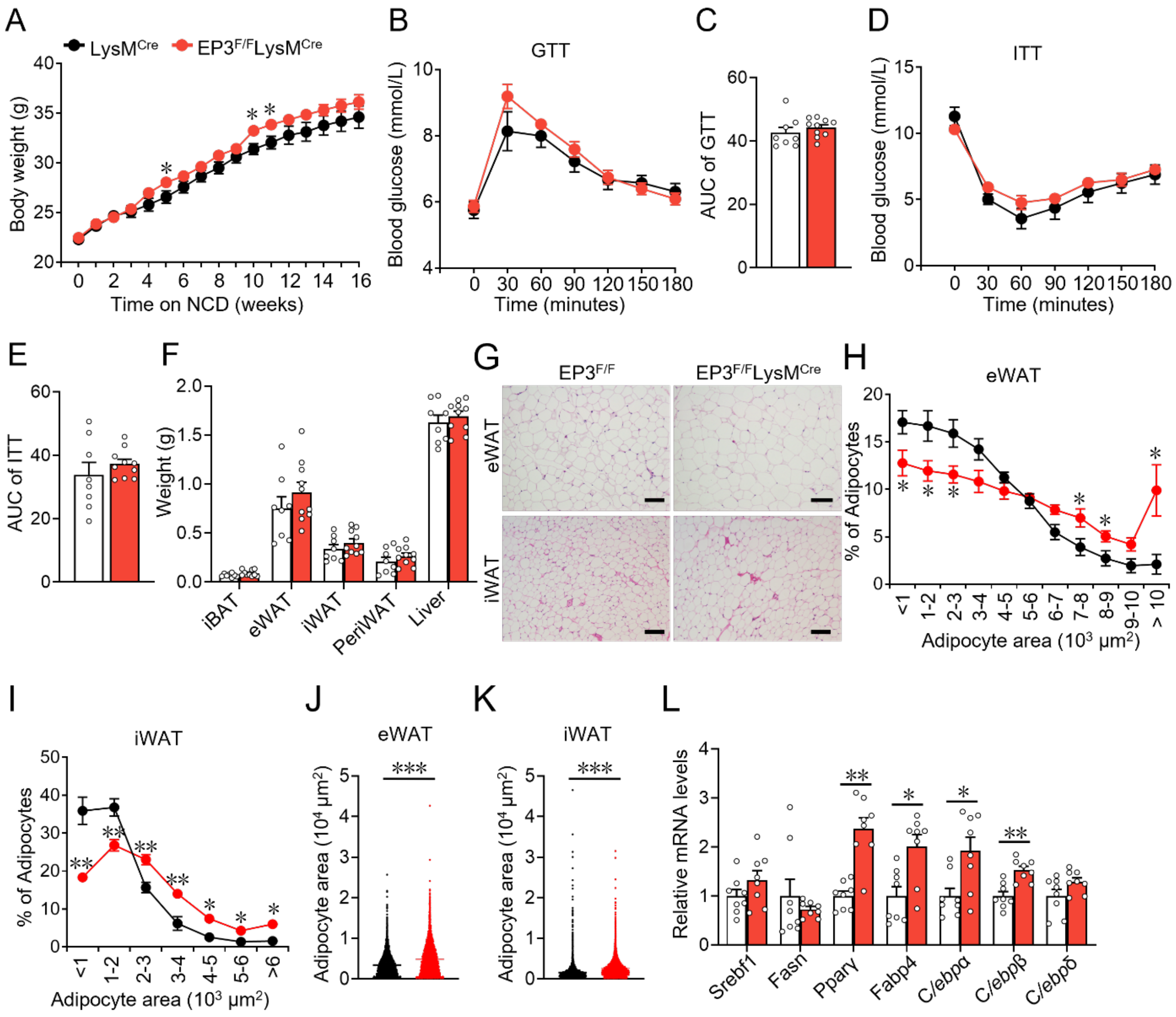

Appendix Figure S4. Macrophage-specific deletion of EP3 had slightly increased body weights in mice fed chow diet.

(**A**), Body weight analysis of LysM^{Cre} and EP3$^{F/F}$LysM^{Cre} mice fed with chow diet (n=8–10).

(**B**), GTT in EP3$^{F/F}$ and EP3$^{F/F}$LysM^{Cre} mice fed with chow diet (n=8–10).

(**C**), AUC of GTT described in (**B**) (n=8–10).

(**D**), ITT in EP3$^{F/F}$ and EP3$^{F/F}$LysM^{Cre} mice fed with chow diet (n=8–10).

(**E**), AUC of ITT described in (**D**) (n=8–10).

(**F**), The weights of adipose tissues of iBAT, eWAT, iWAT, periWAT and liver in EP3$^{F/F}$ and EP3$^{F/F}$LysM^{Cre} mice fed with chow diet (n=8–10).

(**G**), Representative image of H&E staining for eWAT and iWAT from EP3$^{F/F}$ and EP3$^{F/F}$LysM^{Cre} mice fed with chow diet. (Scale bar: 100 μm)

(**H-K**), Quantification of adipocyte area of eWAT (**H** and **J**) and iWAT (**I** and **K**) from images in (**G**) (n>5388 adipocytes measured from 6–8 mice in each group).
(**L**), qRT-PCR analysis of the relative mRNA levels of fatty acid synthesis and adipogenesis genes in eWAT from $EP3^{F/F}$ and $EP3^{F/F}LysM^{Cre}$ mice (n=8).
Data information: Data represent the mean ± SEM. Data are pooled from two independent experiments with biological replicates (**A**-**F**, **H**-**L**). Statistics: Mann-Whitney U-test (**I**-**L**), Unpaired Student's t-test (**A**, **H**). **A**, **H**-**L**, P–values are indicated by asterisks, with *P<0.05, **P<0.01.

Appendix Figure S5

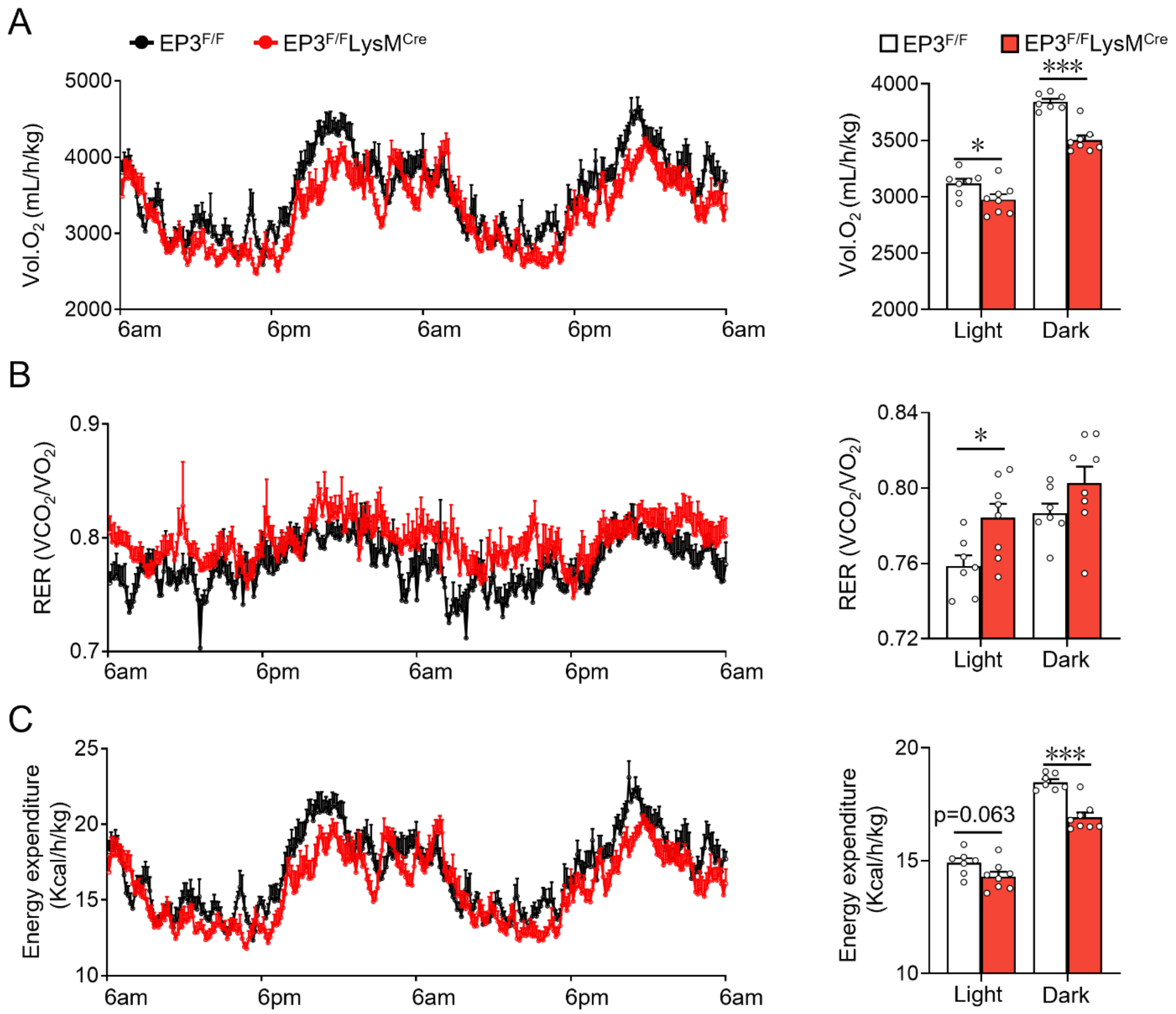

Appendix Figure S5. Macrophage-specific deletion of EP3 exacerbates HFD-induced obesity in mice with decreased energy expenditure.

(**A–C**), Metabolic studies of oxygen consumption rate (**A**) (n=7–8), RER (**B**) (n=7–8), and energy expenditure measurements (**C**) (n=7–8) were measured in $EP3^{F/F}$ and $EP3^{F/F}LysM^{Cre}$ mice fed with HFD for 3 weeks using CLAMS.

Data information: Data represent the mean ± SEM. Data are pooled from two independent experiments with biological replicates (**A-C**). Statistics: Unpaired Student's t-test (**A–C**). **A–C**, P–values are indicated by asterisks, with *P<0.05, **P<0.01, ***P<0.001. RER, respiratory exchange ratio.

Appendix Figure S6

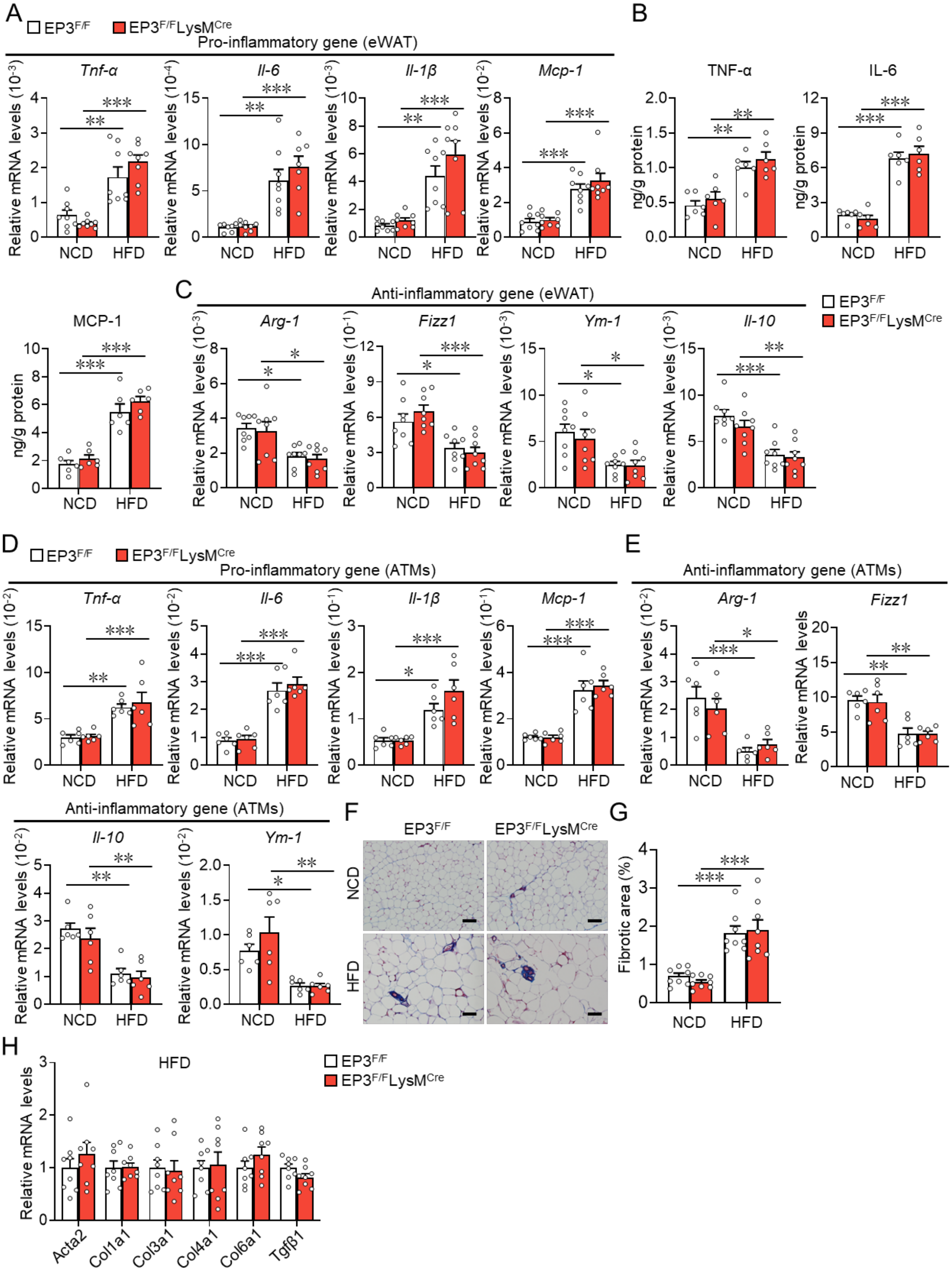

Appendix Figure S6. Macrophage EP3 deletion does not influence inflammation and fibrosis in adipose tissues in mice.

(**A**), qRT-PCR analysis of the relative mRNA levels of pro-inflammatory genes in eWAT from HFD-fed $EP3^{F/F}$ and $EP3^{F/F}LysM^{Cre}$ mice (n=7-8).

(**B**), Elisa analysis of TNF-α, IL-6 and MCP-1 levels in eWAT from HFD-fed $EP3^{F/F}$ and $EP3^{F/F}LysM^{Cre}$ mice (n=6).

(**C**), qRT-PCR analysis of the relative mRNA levels of anti-inflammatory genes in eWAT from HFD-fed $EP3^{F/F}$ and $EP3^{F/F}LysM^{Cre}$ mice (n=8).

(**D**), qRT-PCR analysis of the relative mRNA levels of pro-inflammatory genes in ATMs from HFD-fed $EP3^{F/F}$ and $EP3^{F/F}LysM^{Cre}$ mice(n=6).

(**E**), qRT-PCR analysis of the relative mRNA levels of anti-inflammatory genes in ATMs from HFD-fed $EP3^{F/F}$ and $EP3^{F/F}LysM^{Cre}$ mice (n=6).

(**F**), Representative images of Masson's trichrome staining of eWAT from HFD-fed $EP3^{F/F}$ and $EP3^{F/F}LysM^{Cre}$ mice. (Scale bar: 50 μm).

(**G**), Quantification of fibrotic area of eWAT in (**F**) (n=8).

(**H**), qRT-PCR analysis of the relative mRNA levels of fibrosis genes in eWAT from HFD-fed $EP3^{F/F}$ and $EP3^{F/F}LysM^{Cre}$ mice (n=8).

Data information: Data represent the mean ± SEM. Data are pooled from two independent experiments with biological replicates (**A**-**E**, **G**-**H**). Statistics: Two-way ANOVA (**A**-**E**, **G**). **A**-**E**, **G**, P–values are indicated by asterisks, with *P<0.05, **P<0.01, ***P<0.001.

Appendix Figure S7

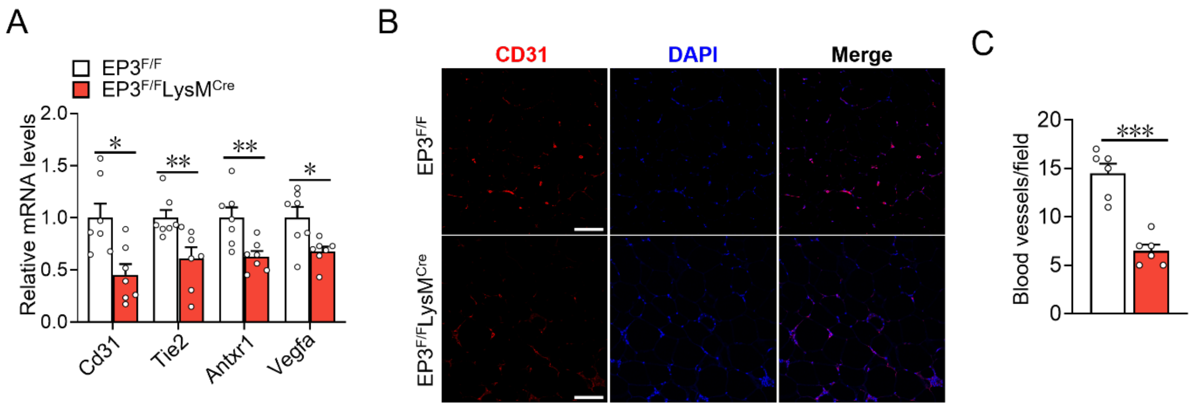

Appendix Figure S7. Macrophage EP3 deletion impairs angiogenesis in mice.

(**A**), qRT-PCR analysis of the relative mRNA levels of angiogenesis-associated genes in eWAT from HFD-fed EP3$^{F/F}$ and EP3$^{F/F}$LysM^{Cre} mice (n=7).

(**B**), Representative images of CD31 immunostaining of the eWAT from HFD-fed EP3$^{F/F}$ and EP3$^{F/F}$LysM^{Cre} mice. Scale bar: 100 μm.

(**C**), Quantification CD31 immunofluorescent staining for blood vessels in (**B**) (n=6).

Data information: Data represent the mean ± SEM. Data are pooled from two independent experiments with biological replicates (**A**, **C**). Statistics: Mann-Whitney U-test (**A**). Unpaired Student's t-test (**C**). **A**, **C**, P–values are indicated by asterisks, with *P<0.05, **P<0.01, ***P<0.001.

Appendix Figure S8

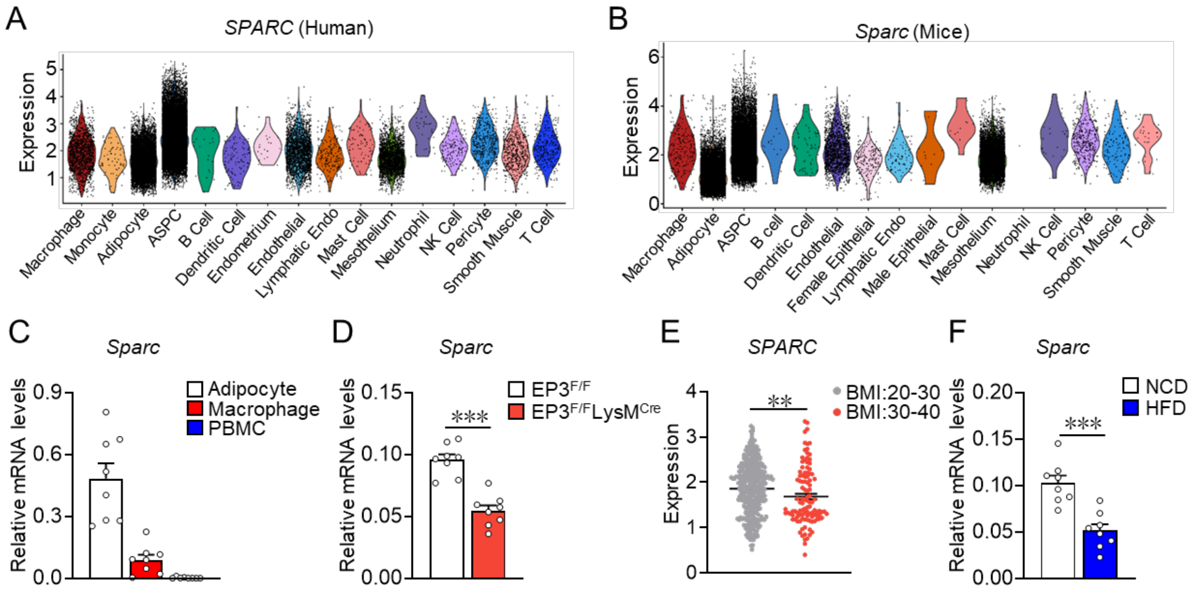

Appendix Figure S8. SPARC is downregulated in ATMs in patients with obesity and HFD-induced mice.

(**A**), Violin plots presenting cellular expression profiles of *SPARC* in white adipose tissues from human subjects with the BMI range of 20-30 (GSE176067, GSE176171).

(**B**), Violin plots presenting cellular expression profiles of *Sparc* in mouse white adipose tissues (GSE176171).

(**C**), qRT-PCR analysis of *Sparc* in macrophages and adipocytes of eWAT and peripheral blood mononuclear cells (PBMCs) in mice.

(**D**), qRT-PCR analysis of the relative mRNA levels of *Sparc* in eWAT macrophages from $EP3^{F/F}$ and $EP3^{F/F}LysM^{Cre}$ mice (n=8).

(**E**), The expression of *SPARC* in human visceral adipose tissue macrophages from obese patients with different BMIs (GSE176171).

(**F**), qRT-PCR analysis of the relative mRNA levels of *Sparc* in eWAT macrophages from HFD-fed mice (n=8).

Data information: Data represent the mean ± SEM. Data are representative of two independent experiments with biological replicates (**C**-**D**, **F**). Statistics: Mann-Whitney U-test (**E**), Unpaired Student's t-test (**D**, **F**). **D**-**F**, P–values are indicated by asterisks, with **P<0.01, ***P<0.001.

Appendix Figure S9

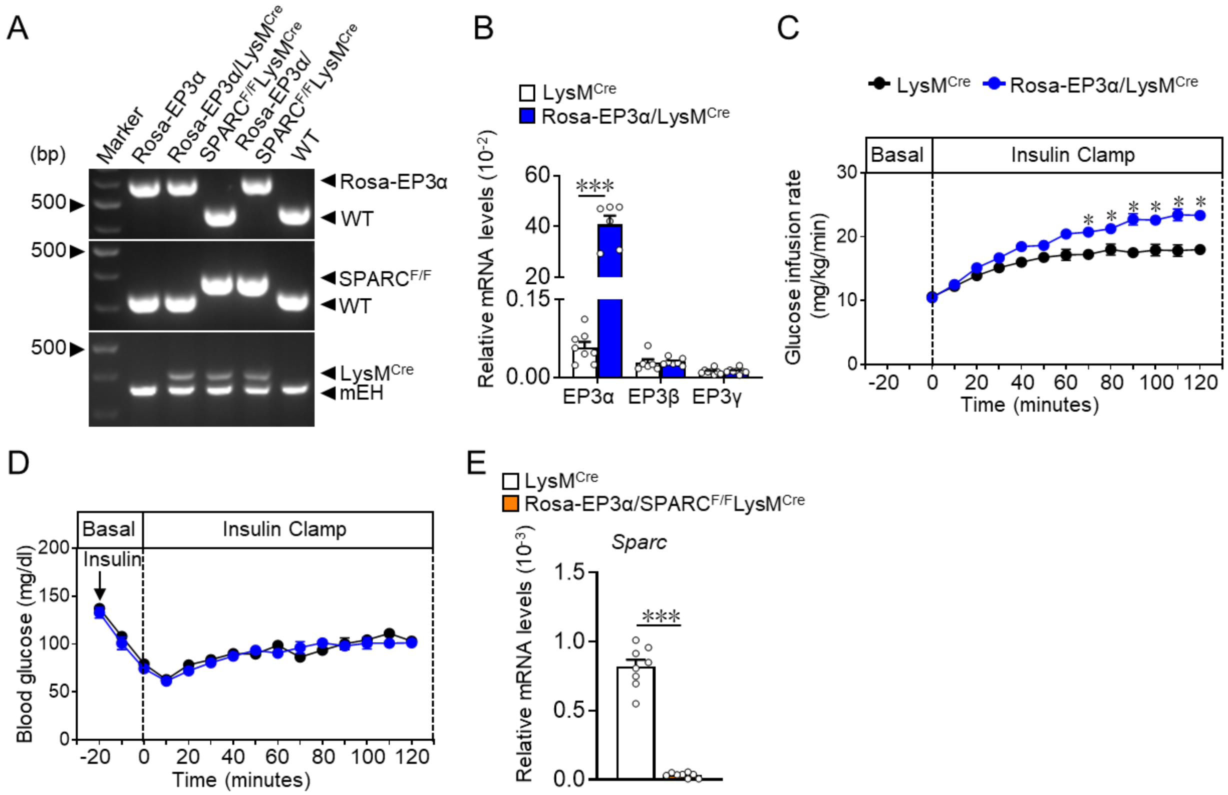

Appendix Figure S9. Generation of macrophage-specific SPARC-deleted and EP3 overexpressed mice.

(**A**)**,** Genotyping of macrophage-specific SPARC-deleted and EP3 overexpressed mice by PCR of genomic DNA extracted from tail biopsies.

(**B**)**,** qRT-PCR analysis of the relative mRNA levels of EP3 isoforms in BMDMs from LysM^{Cre} and Rosa-EP3α/LysM^{Cre} mice (n=6–8).

(**C-D**)**,** Hyperinsulinemic-euglycemic clamp test of LysM^{Cre} and Rosa-EP3α/LysM^{Cre} mice fed with HFD (n=4).

(**E**)**,** qRT-PCR analysis of relative mRNA levels of *Sparc* in BMDMs from LysM^{Cre} and Rosa-EP3α/SPARC$^{F/F}$LysM^{Cre} mice (n=8).

Data information: Data represent the mean ± SEM. Statistics: Mann-Whitney U-test (**B-C**), Unpaired Student's t-test (**E**). **B**–**C**, **E**, P–values are indicated by asterisks with *P<0.05, ***P<0.001.

Appendix Figure S10

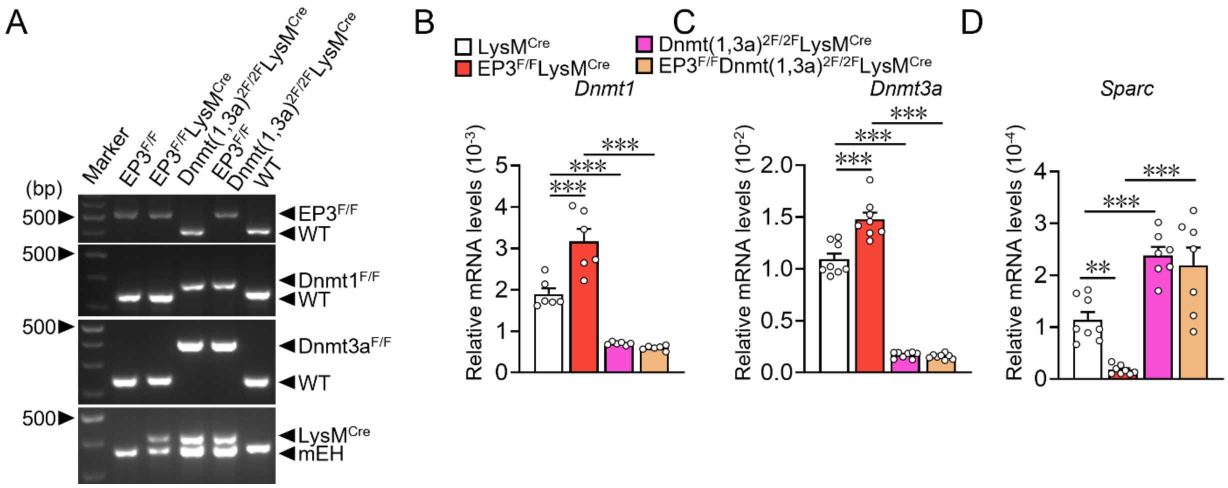

Appendix Figure S10. Generation of macrophage-specific EP3 and Dnmt1/3a-deleted mice. (**A**), Genotyping of macrophage-specific EP3 and Dnmt1/3a-deleted mice by PCR of genomic DNA extracted from the tail biopsies.

(**B-D**), qRT-PCR analysis of the relative mRNA levels of *Dnmt1* (**B**) (n=6), *Dnmt3a* (**C**) (n=8), *Sparc* (**D**) (n=7–8) in BMDMs from $LysM^{Cre}$, $EP3^{F/F}LysM^{Cre}$, $Dnmt(1,3a)^{2F/2F}LysM^{Cre}$, and $EP3^{F/F}Dnmt(1,3a)^{2F/2F}$ $LysM^{Cre}$ mice.

Data information: Data represent the mean ± SEM. Statistics: Two-way ANOVA (**B–D**). **B–D**, P–values are indicated by asterisks, with **P<0.01, ***P<0.001.

Appendix Figure S11

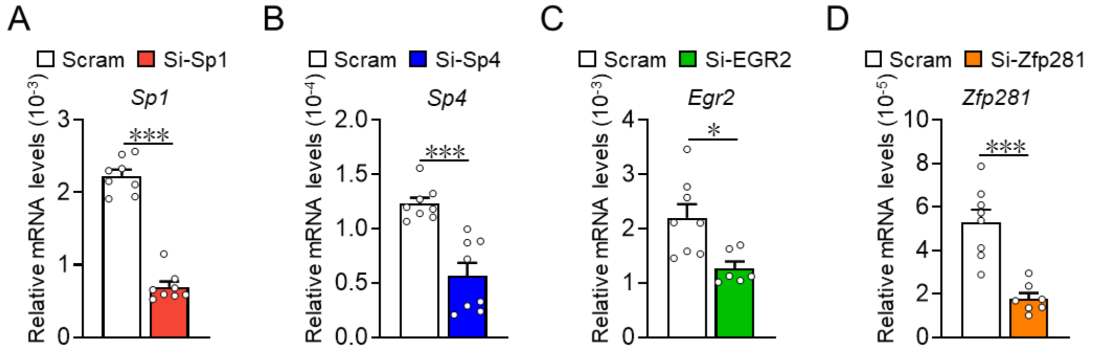

Appendix Figure S11. Knockout efficiency of transcription factors in macrophages.
(**A–D**), qRT-PCR analysis of the relative mRNA levels of *Sp1* (**A**) (n=8), *Sp4* (**B**) (n=8), *Egr2* (**C**) (n=6–8) and *Zfp281*(**D**) (n=7–8) in BMDMs after transfection with Sp1, Sp4, EGR2 and Zfp281 siRNA.
Data information: Data represent the mean ± SEM. Statistics: Mann-Whitney U-test (**A**, **C**), Unpaired Student's t-test (**B**, **D**). **A**–**D**, P–values are indicated by asterisks, with *P<0.05, ***P<0.001.

Appendix Figure S12

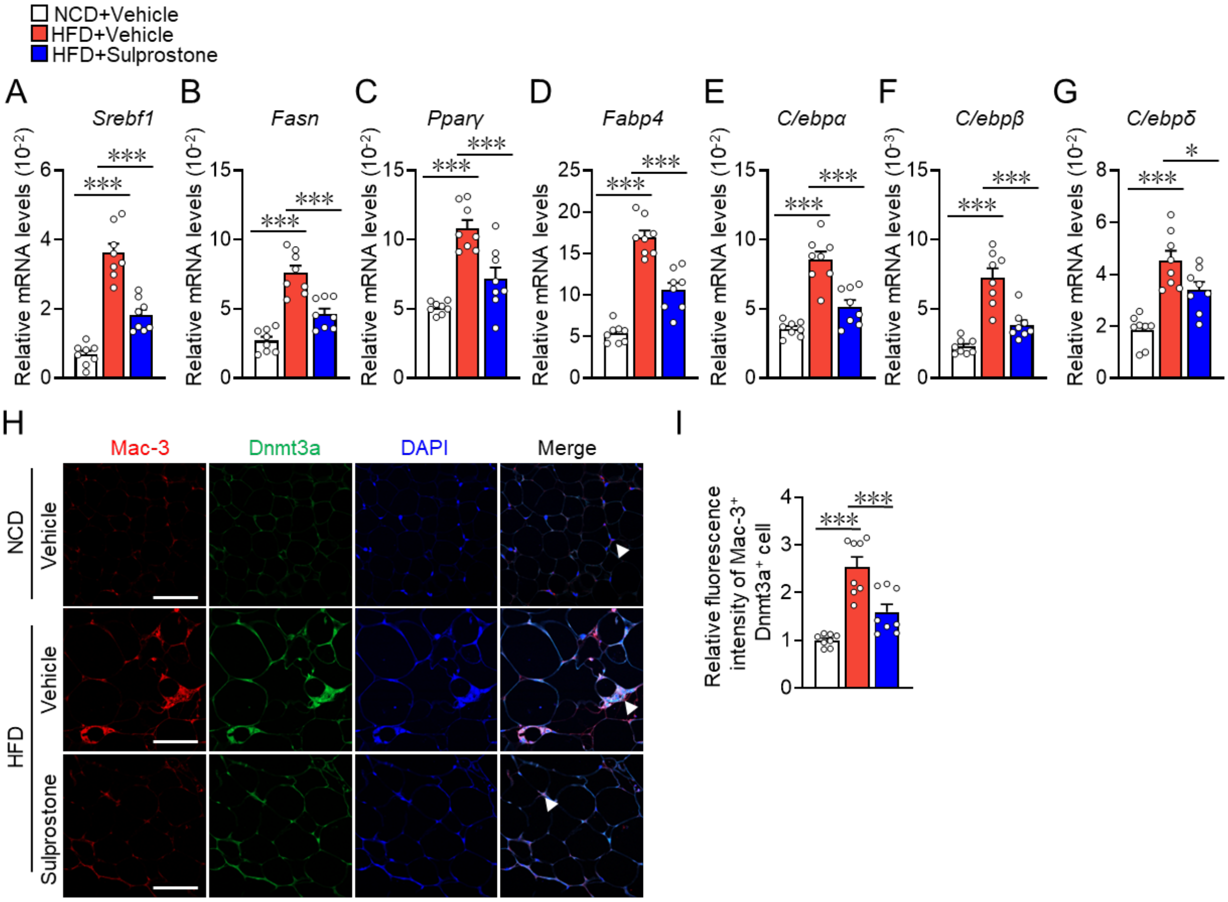

Appendix Figure S12. EP3 agonist sulprostone treatment attenuates adipogenesis in adipose tissues of HFD-induced mice.

(**A–G**), qRT-PCR analysis of the relative mRNA levels of fatty acid synthesis and adipogenesis genes in eWAT from HFD-challenged mice with or without sulprostone treatment (n=8).

(**H**), Representative images of Mac-3, Dnmt3a immunostaining of the eWAT from HFD-challenged mice with or without Sulprostone treatment (Scale bar: 50 μm). White arrowheads indicate Mac-3^{+}Dnmt3a^{+}cell.

(**I**), Quantification of Dnmt3a fluorescence intensity in (**H**) (n=8).

Data information: Data represent the mean ± SEM. Data are pooled from two independent experiments with biological replicates (**A-G**, **I**). Statistics: One-way ANOVA (**A–G**, **I**). **A–G**, **I**, P–values are indicated by asterisks, with *P<0.05, ***P<0.001.

Appendix Tables

Appendix Table S1. Nucleotide sequences of siRNA used for transfection of BMDMs.

Duplex no.	Forward sequences	Reverse sequences
SPARC Duplex 1	CCAGAACCAUCAUUGCAAATT	UUUGCAAUGAUGGUUCUGGTT
SPARC Duplex 2	GCAAUGACAACAAGACCUUTT	AAGGUCUUGUUGUCAUUGCTT
Dnmt1 Duplex 1	GCAAAGAGUAUGAGCCAAUTT	AUUGGCUCAUACUCUUUGCTT
Dnmt1 Duplex 2	CCAGGAGUACGCAAGGUUUTT	AAACCUUGCGUACUCCUGGTT
Dnmt3a Duplex 1	CCAUGUACCGCAAAGCCAUTT	AUGGCUUUGCGGUACAUGGTT
Dnmt3a Duplex 2	GCAUGUGCCAGAACUGUAATT	UUACAGUUCUGGCACAUGCTT
Sp1 Duplex 1	CCUUCACAACUCAAGCUAUUU	AAAUAGCUUGAGUUGUGAAGG
Sp1 Duplex 2	GCAGCAGUAAUACCACCCUAA	UUAGGGUGGUAUUACUGCUGC
Sp4 Duplex 1	CGUUUCAAUACCAUUGCAAUU	AAUUGCAAUGGUAUUGAAACG
Sp4 Duplex 2	CCAAUGGGAAUCAGUUAGUUU	AAACUAACUGAUUCCCAUUGG
Zfp281 Duplex 1	ACCUUUAUCACUAACUCUAAU	AUUAGAGUUAGUGAUAAAGGU
Zfp281 Duplex 2	CUUGGCAUCGUAUCGAAUAA	AUUAUUCGAUACGAUGCCAA
EGR2 Duplex 1	GCUGUACAGGAGAUCUCUA	UAGAGAUCUCCUGUACAGC
EGR2 Duplex 2	GCUGCUAUCCAGAAGGUAU	AUACCUUCUGGAUAGCAGC
Pparγ Duplex 1	GACAUGAAUUCCUUAAUGATT	UCAUUAAGGAAUUCAUGUCTT
Pparγ Duplex 2	GCUCCACACUAUGAAGACAUUTT	AAUGUCUUCAUAGUGUGGAGCTT

Appendix Table S2. Primers used for RT-qPCR.

Gene name	Forward primer sequence (5'-3')	Reverse primer sequence (5'-3')
EP1	TAACGATGGTCACGCGATGG	ATGCAGTAGTGGGCTTAGGG
EP2	GCTCGCCTGCAACATCAGCGTTA	AGCTCGGAGGTCCCACTTTTCCT
EP3	GGATCATGTGTGTGCTGTCC	GCAGAACTTCCGAAGAAGGA
EP4	GTTCCGAGACAGCAAAAGC	CACCCCGAAGATGAACATCAC
FP	CTGGACTCATCGCAAACACAA	AGGAAGCCTTTGACTTCTGTCTA
DP1	AACCTCTATGACATGCACAGGCG	AAGGCTTGGAGGTCTTCTGAGTC
DP2	TCTCAACCAATCAGCACACCCGA	GATGTAGCGGAGGCTAGAGTTGC
TP	TGCATCCGCGGTTCAGTTCACAGC	TGGGGCAGGGCACAGGTCAATGG
IP	CGGGCACGAGAGGATGAAGTTTAC	GGTTGAAGGCGTTGAAGCGGAAGG
EP3α	GGATCATGTGTGTGCTGTCC	GCAGAACTTCCGAAGAAGGA
EP3β	TGAACAACCTGAAGTGGACTTTC	ATTCTCAGACCCAGGGAAACAGG
EP3γ	TTCGCTGAACCAGATCTTGGATC	TAGACAATGAGATGGCCTGCCCT
C/ebpα	CAAGAACAGCAACGAGTACCG	GTCACTGGTCAACTCCAGCAC
C/ebpβ	GCTGAGCGACGAGTACAAGA	TTGAACAAGTTCCGCAGGGT
C/ebpδ	GCCATGTACGACGACGAGAG	GGTTGCTGTTGAAGAGGTCG
Fabp4	GCGTGGAATTCGATGAAATCA	CCCGCCATCTAGGGTTATGA
Pparγ	TTCGATCCGTAGAAGCCGTG	TCCTTGGCCCTCTGAGATGA
Srebf1	GGAGCCATGGATTGCACATT	GGCCCGGGAAGTCACTGT
Fasn	GAGTTCTCAGGCCGGGATAGGT	TGGTATAGACGACGGGCACAGA
Sparc	GTGGAAATGGGAGAATTTGAGGA	CTCACACACCTTGCCATGTTT
Dnmt1	ACCGCTTCTACTTCCTCGAGGCCTA	GTTGCAGTCCTCTGTGAACACTGTGG
Dnmt3a	AGCGTCACACAGAAGCATATCCAGGAG	GGCCAGTACCCTCATAAAGTCCCTTGC
Dnmt3b	AGCGGGTATGAGGAGTGCAT	GGGAGCATCCTTCGTGTCTG
Tet1	ACACAGTGGTGCTAATGCAG	AGCATGAACGGGAGAATCGG
Tet2	AGAGAAGACAATCGAGAAGTCGG	CCTTCCGTACTCCCAAACTCAT
Tet3	AGGCAGCTAAGCACCTCAG	GGCCCCGTAAGATGACACAG
Sp1	AGGGTCCGAGTCAGTCAGG	CTCGCTGCCATTGGTACTGTT
Sp4	ATGAGCGATCAGAAGAAGGAGG	GGAGTCCCTATTTTGCTGCAAG
Egr2	TCAGTGGTTTTATGCACCAGC	GAAGCTACTCGGATACGGGAG
Zfp281	CGCAGTGCGTGTTATCCTC	TCCTTCTTGAAAGTCATGTCCG
Ciead	ATCACAACTGGCCTGGTTACG	TACTACCCGGTGTCCATTTCT
Prdm16	CAGCACGGTGAAGCCATTC	GCGTGCATCCGCTTGTG
Ucp1	CTGCCAGGACAGTACCCAAG	TCAGCTGTTCAAAGCACACA
Pgc-1α	TATGGAGTGACATAGAGTGTGCT	CCACTTCAATCCACCCAGAAAG
Dio-2	AATTATGCCTCGGAGAAGACCG	GGCAGTTGCCTAGTGAAAGGT
Agt	TCTCCTTTACCACAACAAGAGCA	CTTCTCATTCACAGGGGAGGT
Psat1	AAGCCACCAAGCAAGTGGTTA	GATGCCGAGTCCTCTGTAGTC
Tnf-α	CTGAACTTCGGGGTGATCGG	GGCTTGTCACTCGAATTTTGAGA
Il-6	CTGCAAGAGACTTCCATCCAG	AGTGGTATAGACAGGTCTGTTGG

Il-1β	GCAACTGTTCCTGAACTCAACT	ATCTTTTGGGGTCCGTCAACT
Mcp-1	TTAAAAACCTGGATCGGAACCAA	GCATTAGCTTCAGATTTACGGGT
Nos2	GTTCTCAGCCCAACAATACAAGA	GTGGACGGGTCGATGTCAC
Il-10	GCTCTTACTGACTGGCATGAG	CGCAGCTCTAGGAGCATGTG
Arg-1	CTCCAAGCCAAAGTCCTTAGAG	AGGAGCTGTCATTAGGGACATC
Ym-1	CAGGTCTGGCAATTCTTCTGAA	GTCTTGCTCATGTGTGTAAGTGA
Fizz1	CCAATCCAGCTAACTATCCCTCC	ACCCAGTAGCAGTCATCCCA
Acta2	GTCCCAGACATCAGGGAGTAA	TCGGATACTTCAGCGTCAGGA
Tgfb1	CTCCCGTGGCTTCTAGTGC	GCCTTAGTTTGGACAGGATCTG
Co1a1	TAAGGGTCCCCAATGGTGAGA	GGGTCCCTCGACTCCTACAT
Col3a1	ACGTAGATGAATTGGGATGCAG	GGGTTGGGGCAGTCTAGTG
Col4a1	CTGGCACAAAAGGGACGAG	ACGTGGCCGAGAATTTCACC
Col6a1	GGATCTATTCTTCGTGCTCGAC	TCTCAGGTTGTCAATGAAGCG
Cd31	ACCGGGTGCTGTTCTATAAGG	TCACCTCGTACTCAATCGTGG
Tie2	GAGTCAGCTTGCTCCTTTATGG	AGACACAAGAGGTAGGGAATTGA
Vegfa	GTCCGATTGAGACCCTGGTG	TTGACCCTTTCCCTTTCCTCG
Antxr1	ACGGAGGATTCGACCTCTACT	CCTTGTCGGATCTGTTCCCTG
GAPDH	TGGCCTTCCGTGTTCCTAC	GAGTTGCTGTTGAAGTCGCA

Appendix Table S3. Primer design for BSP (Bisulfite Sequencing PCR) methylation analysis.

SPARC	Forward primer sequence(5'-3')	Reverse primer sequence(5'-3')
	GGTTGATAGGGAGGTGTATATTT	TCCCCCACTACCATACCAAC
