# Supplementary figures and images for: Deletion of EP3 prostaglandin receptor in murine macrophages aggravates diet-induced obesity by suppressing SPARC (part 1 of 2)
Source: EMBO J. 2025 Jul 23;44(18):4962–83. doi: 10.1038/s44318-025-00508-y (PMC12436609; doi:10.1038/s44318-025-00508-y)

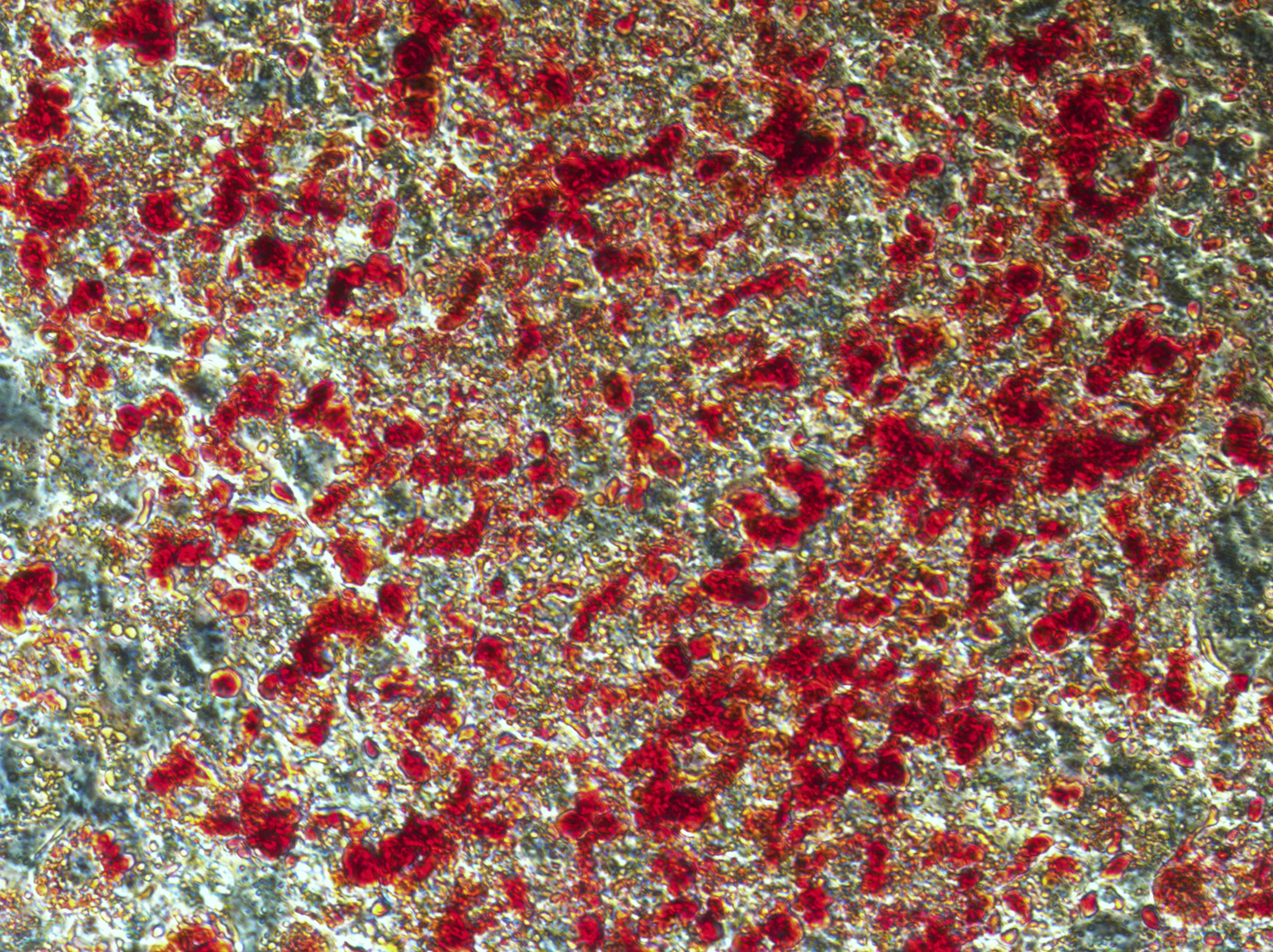

Supplement: Supplementary file 3 — Source data Fig. 1 [file 44318_2025_508_MOESM3_ESM.zip › Source data Fig.1/Figure 1/Figure 1G/L-798106.tif]

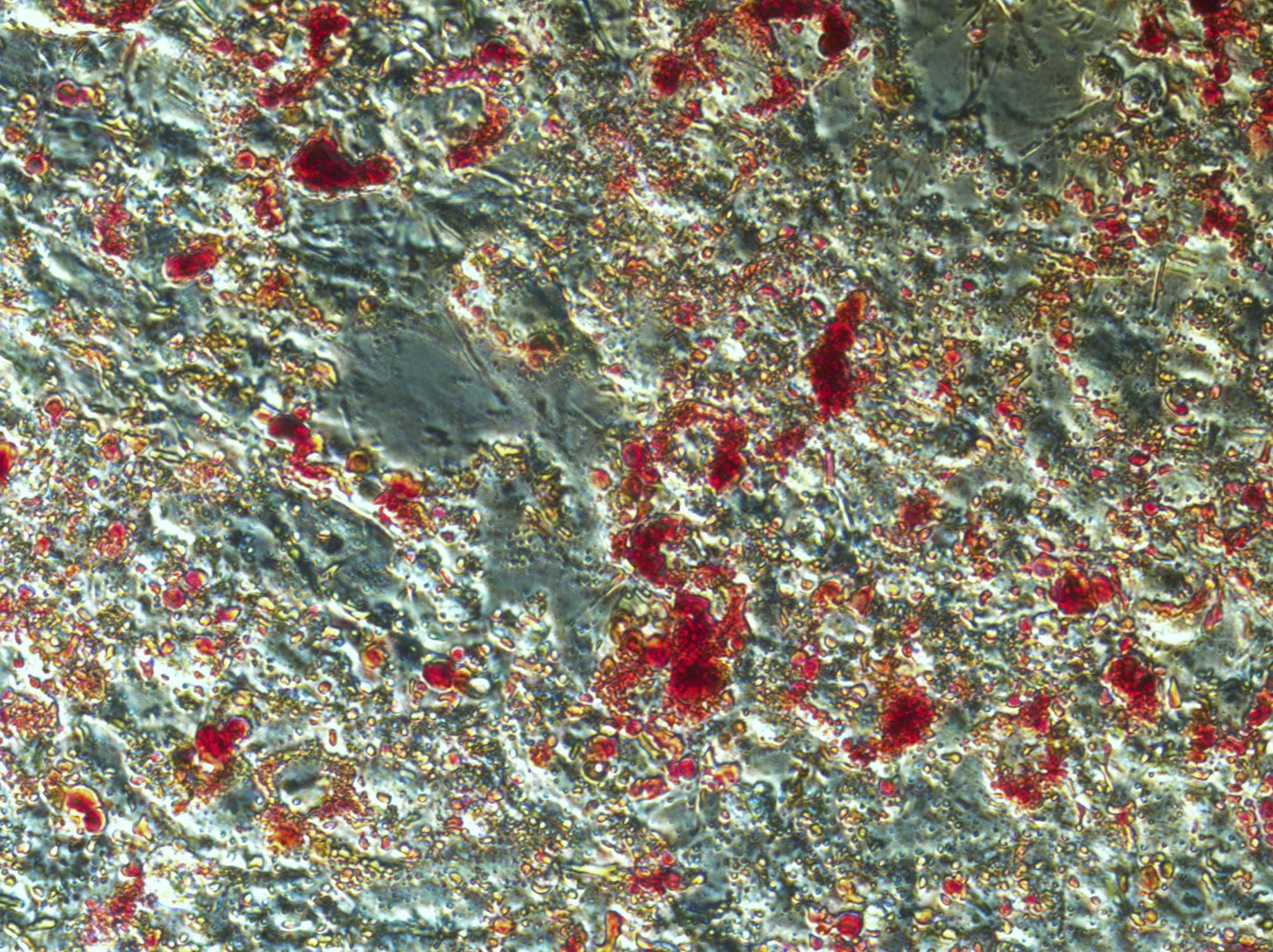

Supplement: Supplementary file 3 — Source data Fig. 1 [file 44318_2025_508_MOESM3_ESM.zip › Source data Fig.1/Figure 1/Figure 1G/Sulprostone.tif]

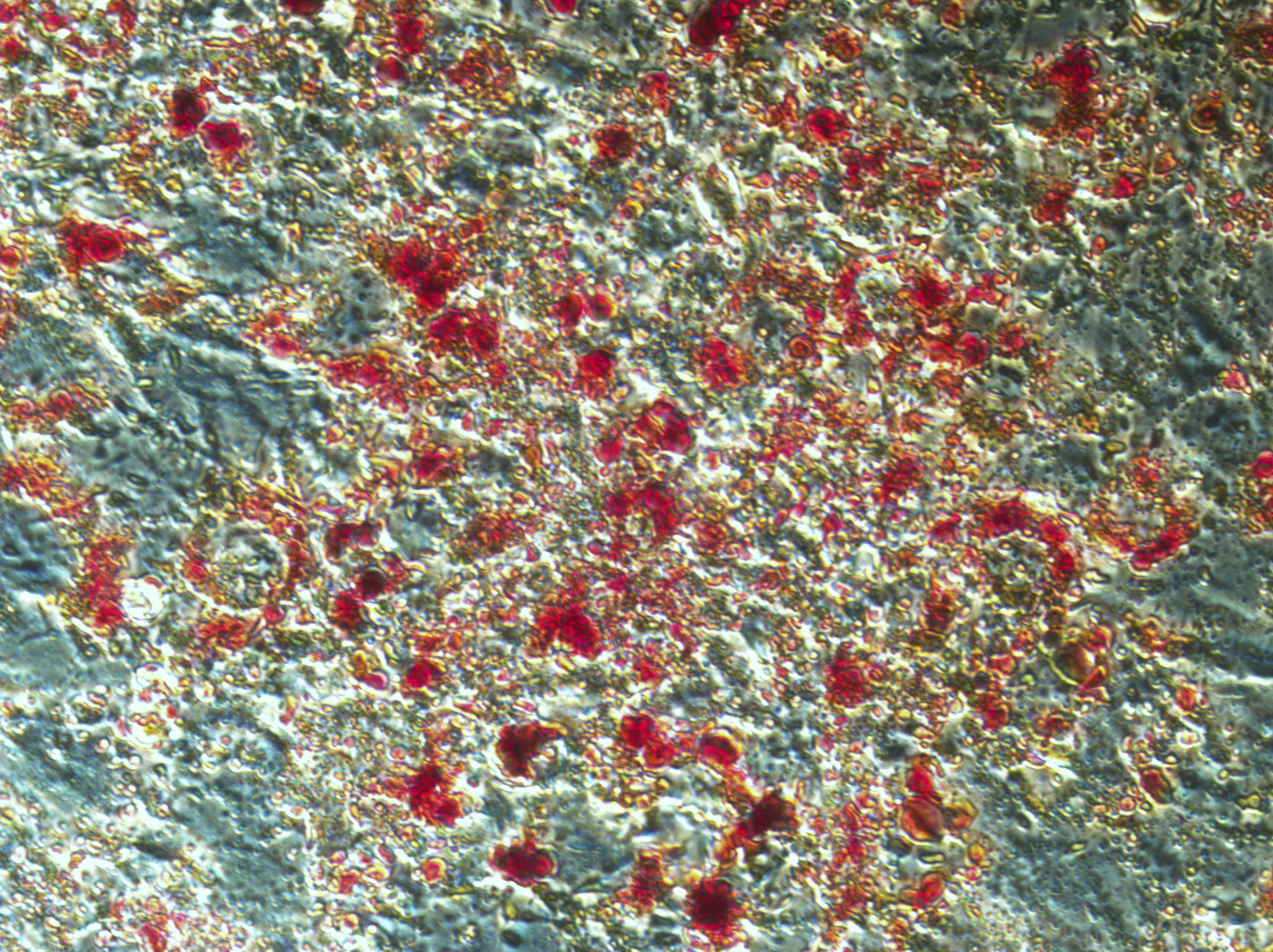

Supplement: Supplementary file 3 — Source data Fig. 1 [file 44318_2025_508_MOESM3_ESM.zip › Source data Fig.1/Figure 1/Figure 1G/Vehicle.tif]

Figure 1J

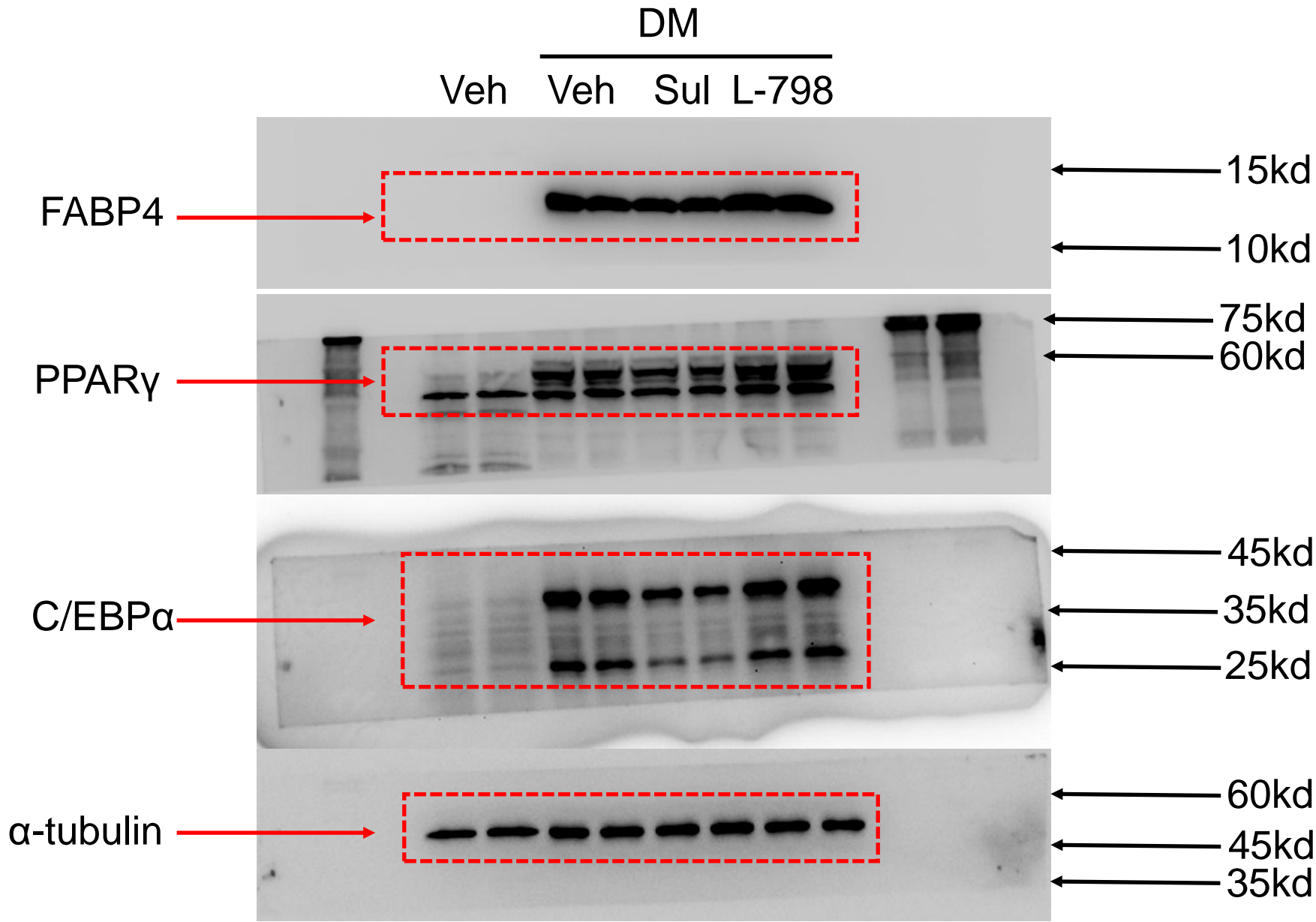

Supplement: Supplementary file 3 — Source data Fig. 1 [file 44318_2025_508_MOESM3_ESM.zip › Source data Fig.1/Figure 1/Figure 1J/Figure 1J.pdf]

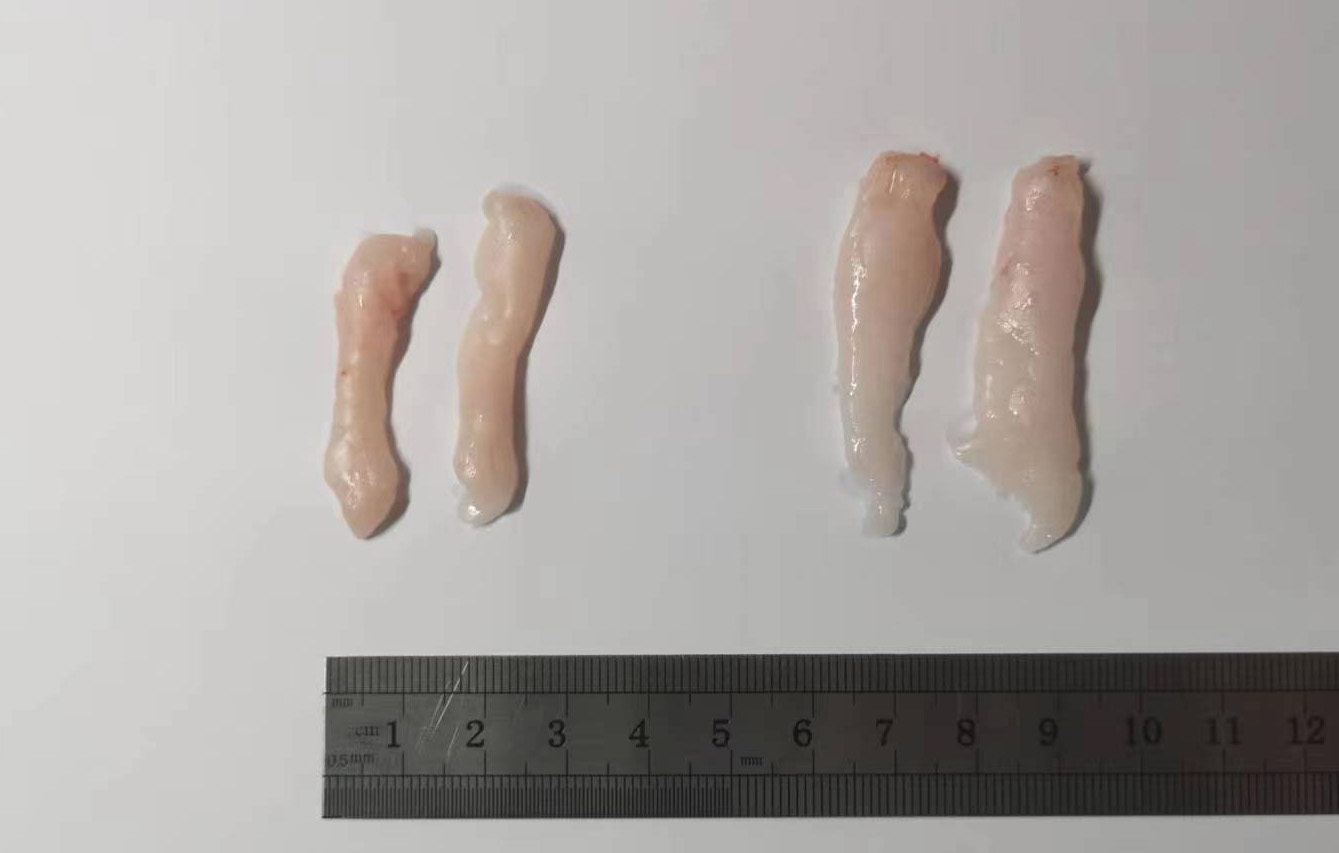

Supplement: Supplementary file 4 — Source data Fig. 2 [file 44318_2025_508_MOESM4_ESM.zip › Source data Fig.2/Figure 2E/eWAT.jpg]

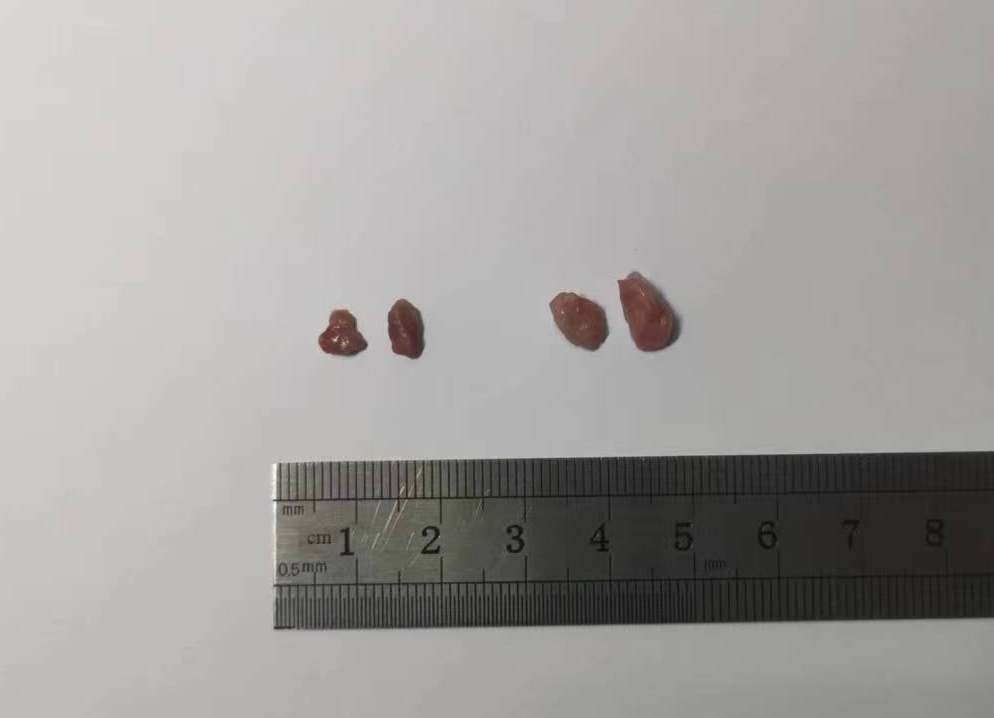

Supplement: Supplementary file 4 — Source data Fig. 2 [file 44318_2025_508_MOESM4_ESM.zip › Source data Fig.2/Figure 2E/iBAT.jpg]

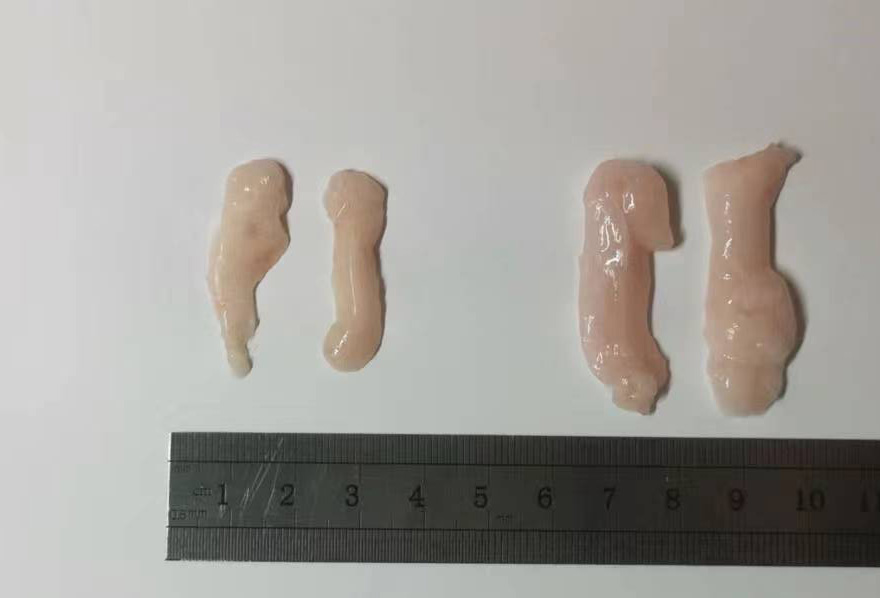

Supplement: Supplementary file 4 — Source data Fig. 2 [file 44318_2025_508_MOESM4_ESM.zip › Source data Fig.2/Figure 2E/IWAT.jpg]

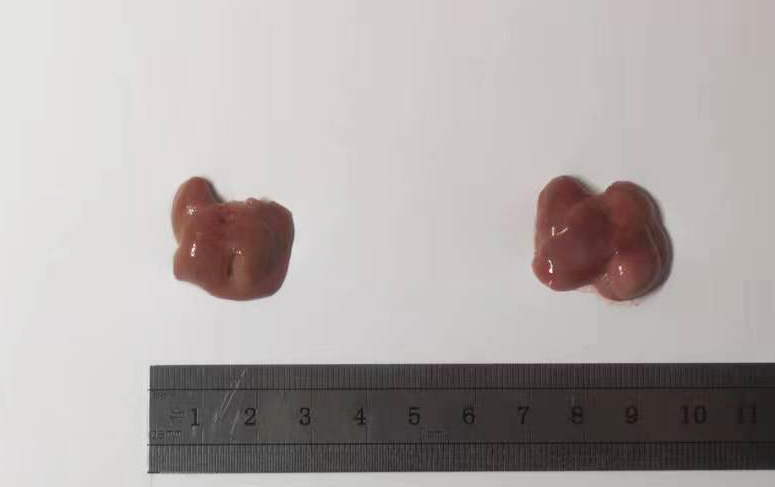

Supplement: Supplementary file 4 — Source data Fig. 2 [file 44318_2025_508_MOESM4_ESM.zip › Source data Fig.2/Figure 2E/Liver.jpg]

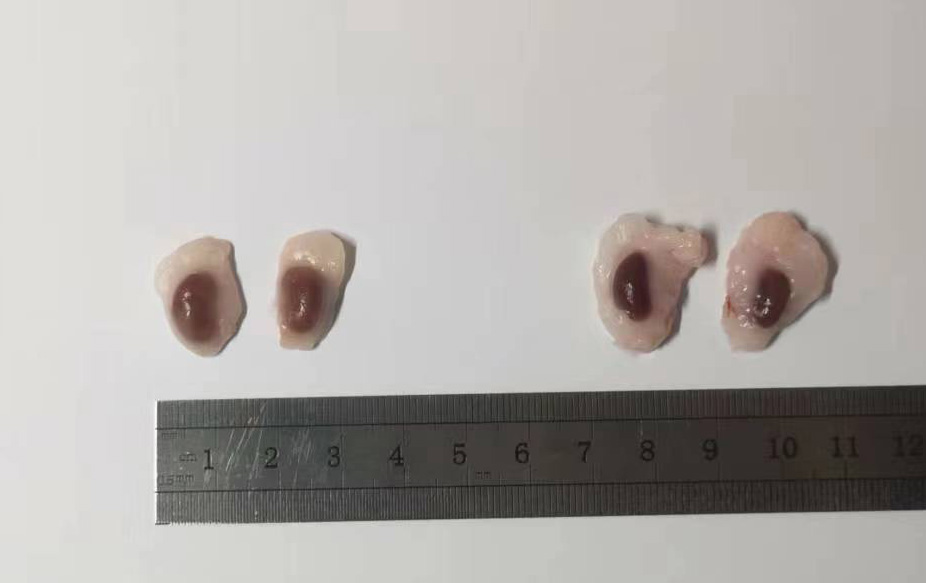

Supplement: Supplementary file 4 — Source data Fig. 2 [file 44318_2025_508_MOESM4_ESM.zip › Source data Fig.2/Figure 2E/PeriWAT.jpg]

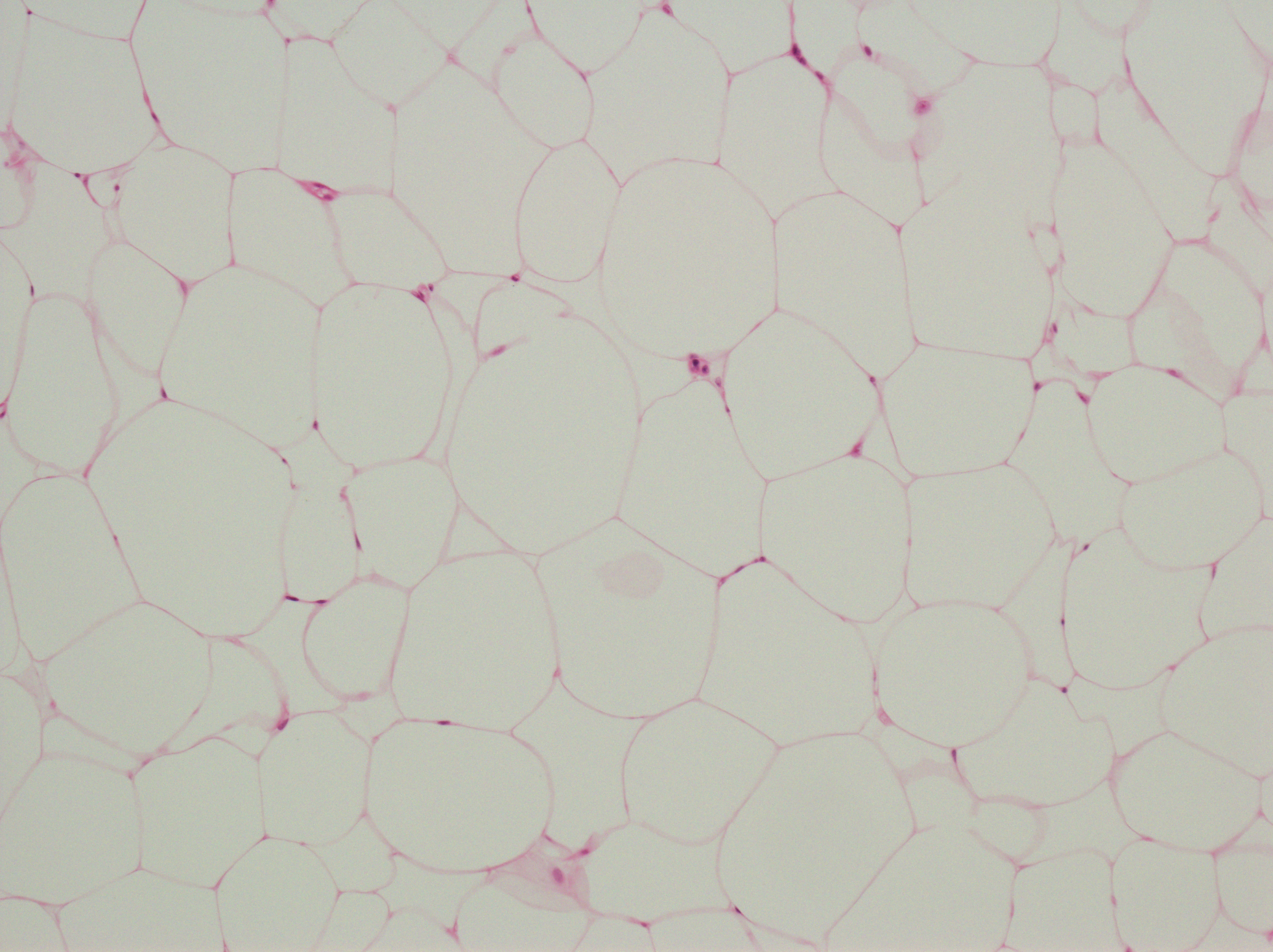

Supplement: Supplementary file 4 — Source data Fig. 2 [file 44318_2025_508_MOESM4_ESM.zip › Source data Fig.2/Figure 2G/eWAT-EP3Flox.tif]

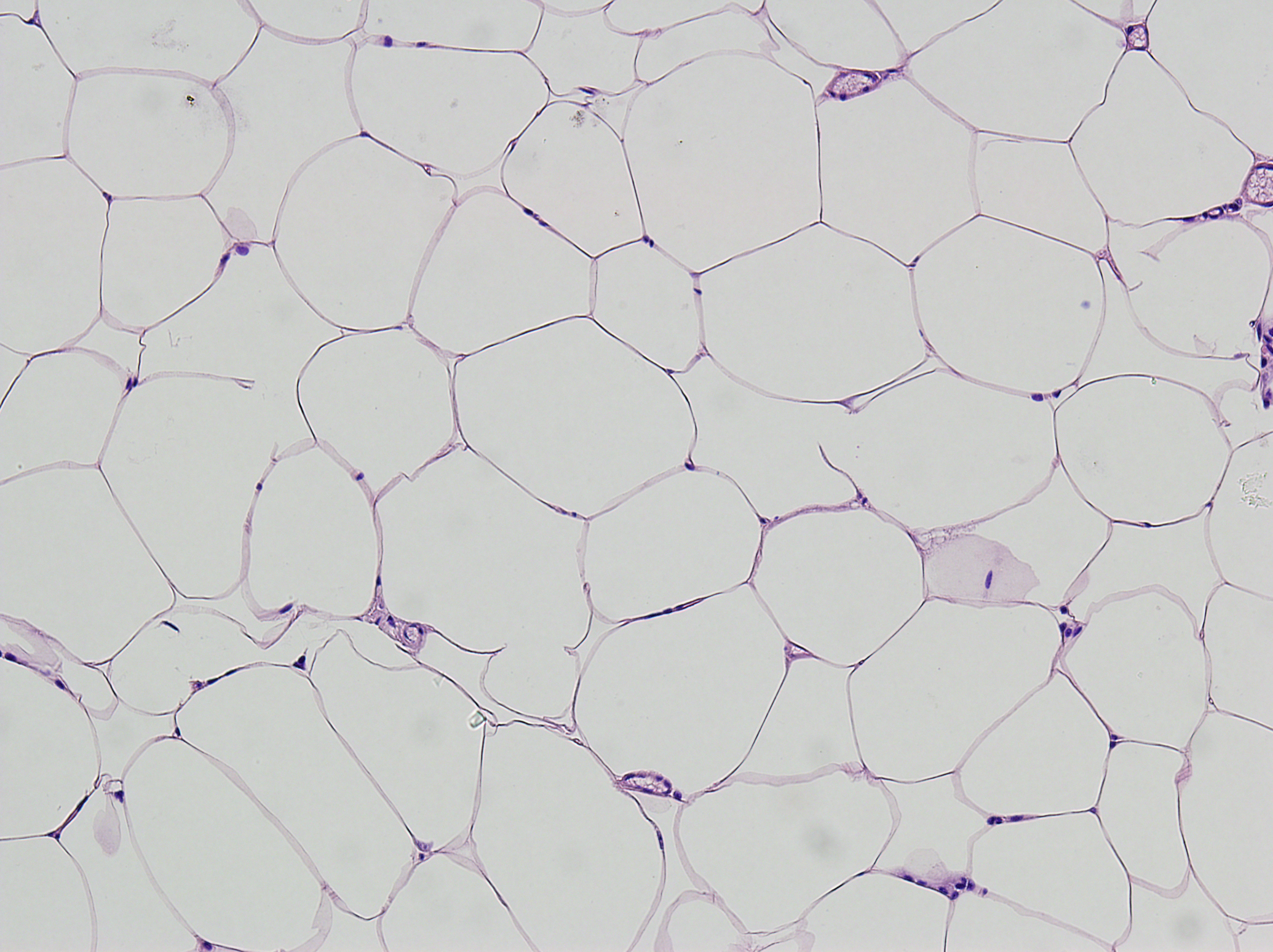

Supplement: Supplementary file 4 — Source data Fig. 2 [file 44318_2025_508_MOESM4_ESM.zip › Source data Fig.2/Figure 2G/eWAT-EP3FloxLysMCre.tif]

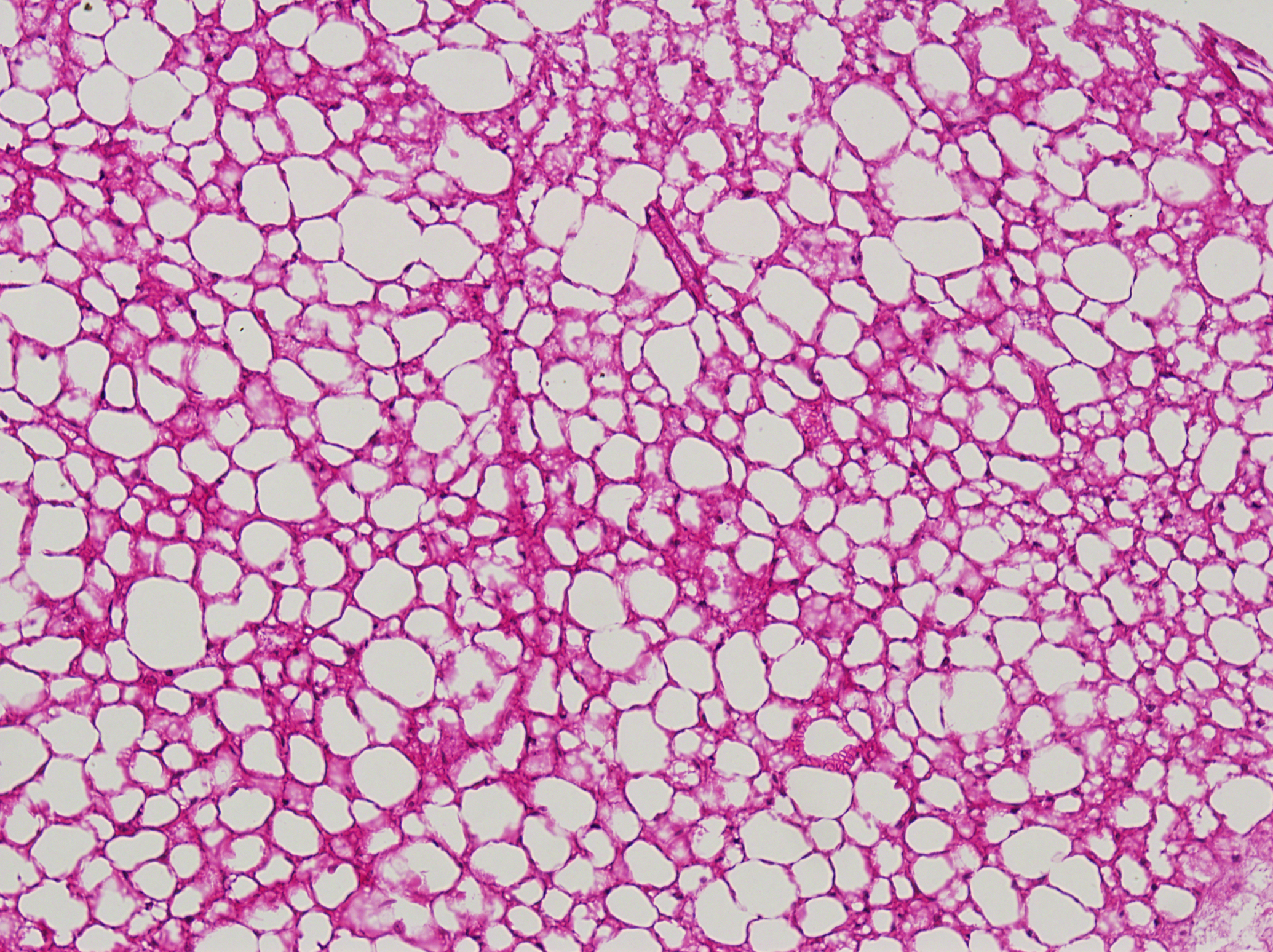

Supplement: Supplementary file 4 — Source data Fig. 2 [file 44318_2025_508_MOESM4_ESM.zip › Source data Fig.2/Figure 2G/iBAT-EP3Flox.tif]

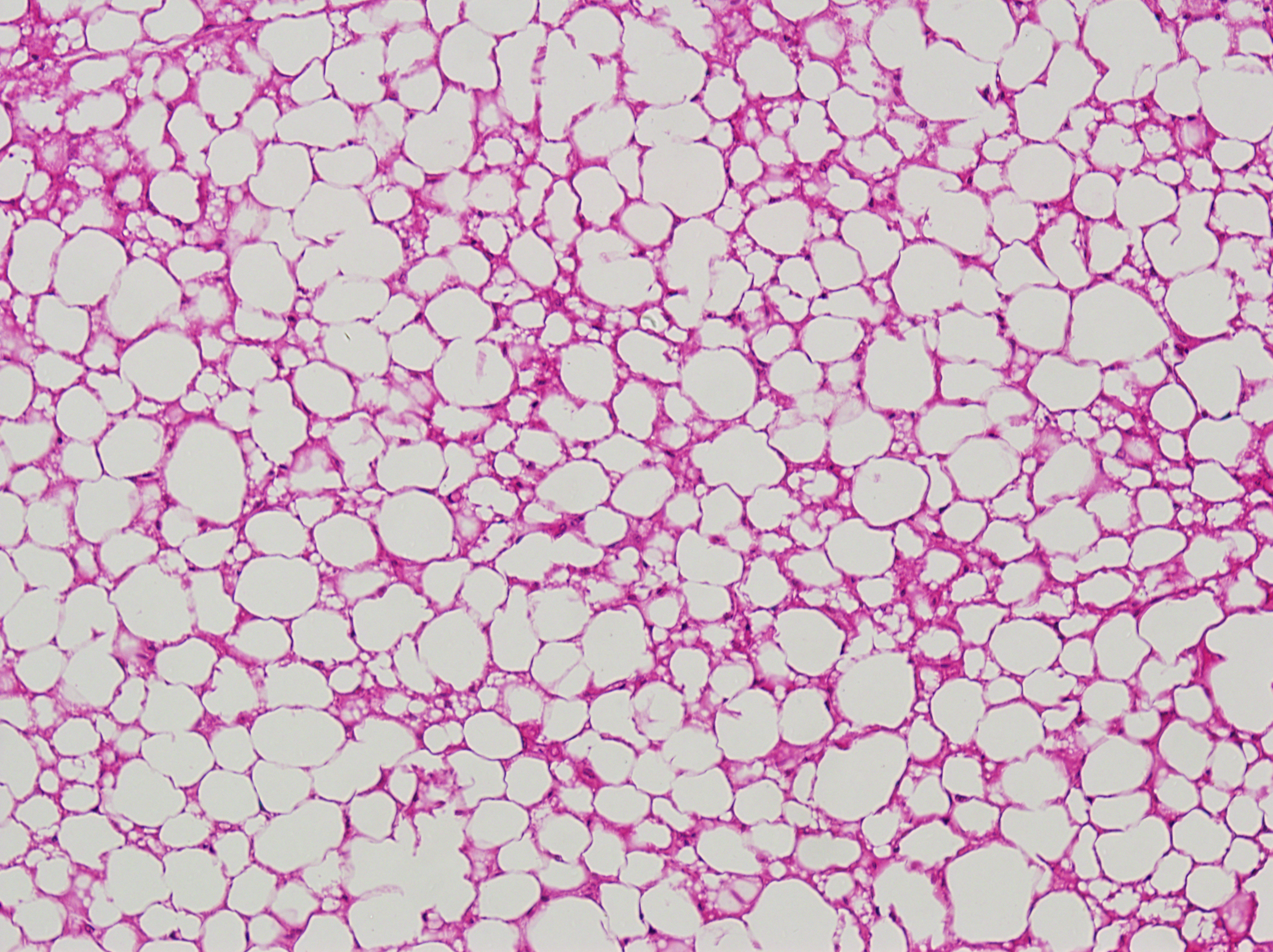

Supplement: Supplementary file 4 — Source data Fig. 2 [file 44318_2025_508_MOESM4_ESM.zip › Source data Fig.2/Figure 2G/iBAT-EP3FloxLysMCre.tif]

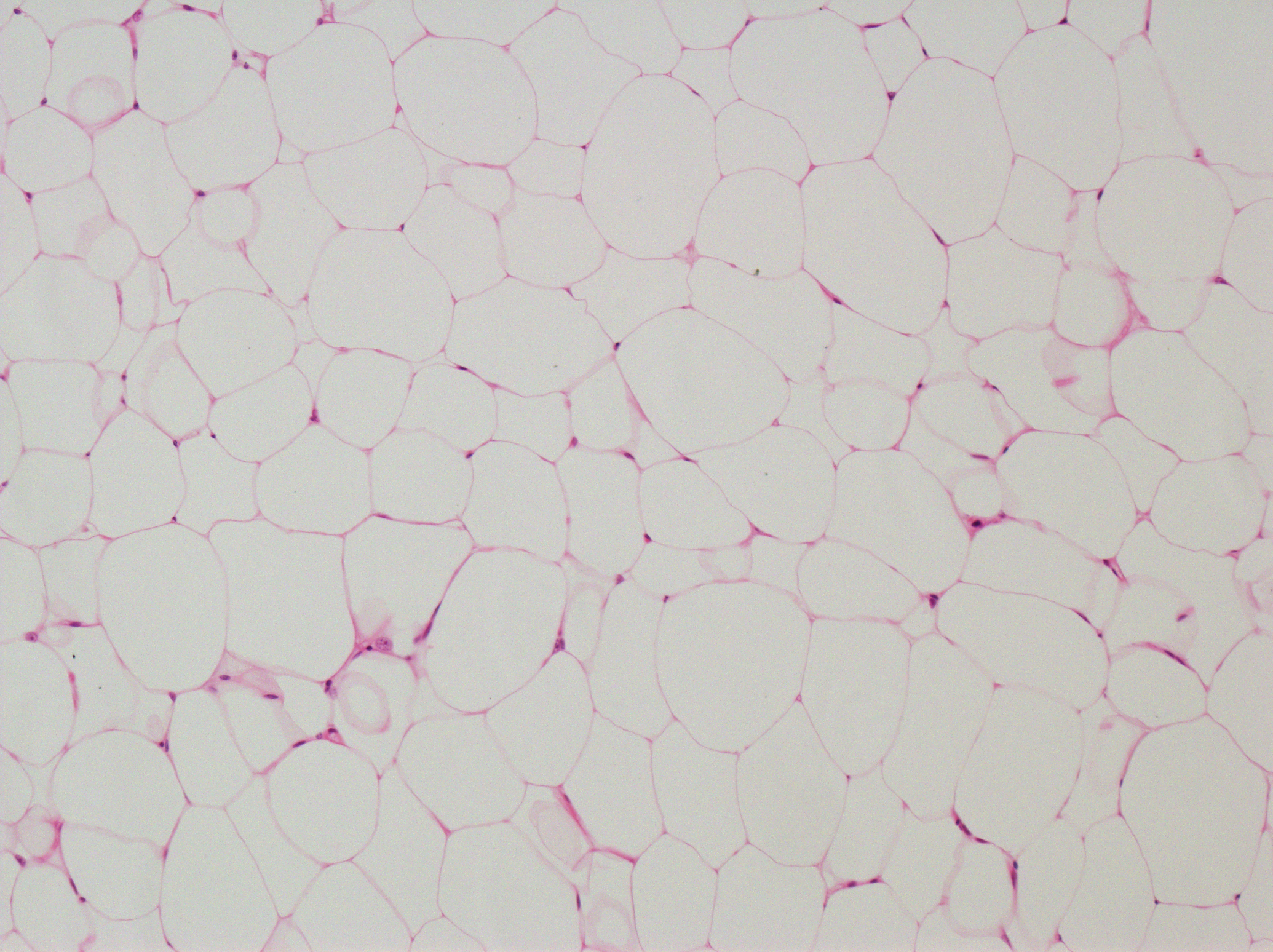

Supplement: Supplementary file 4 — Source data Fig. 2 [file 44318_2025_508_MOESM4_ESM.zip › Source data Fig.2/Figure 2G/iWAT-EP3Flox.tif]

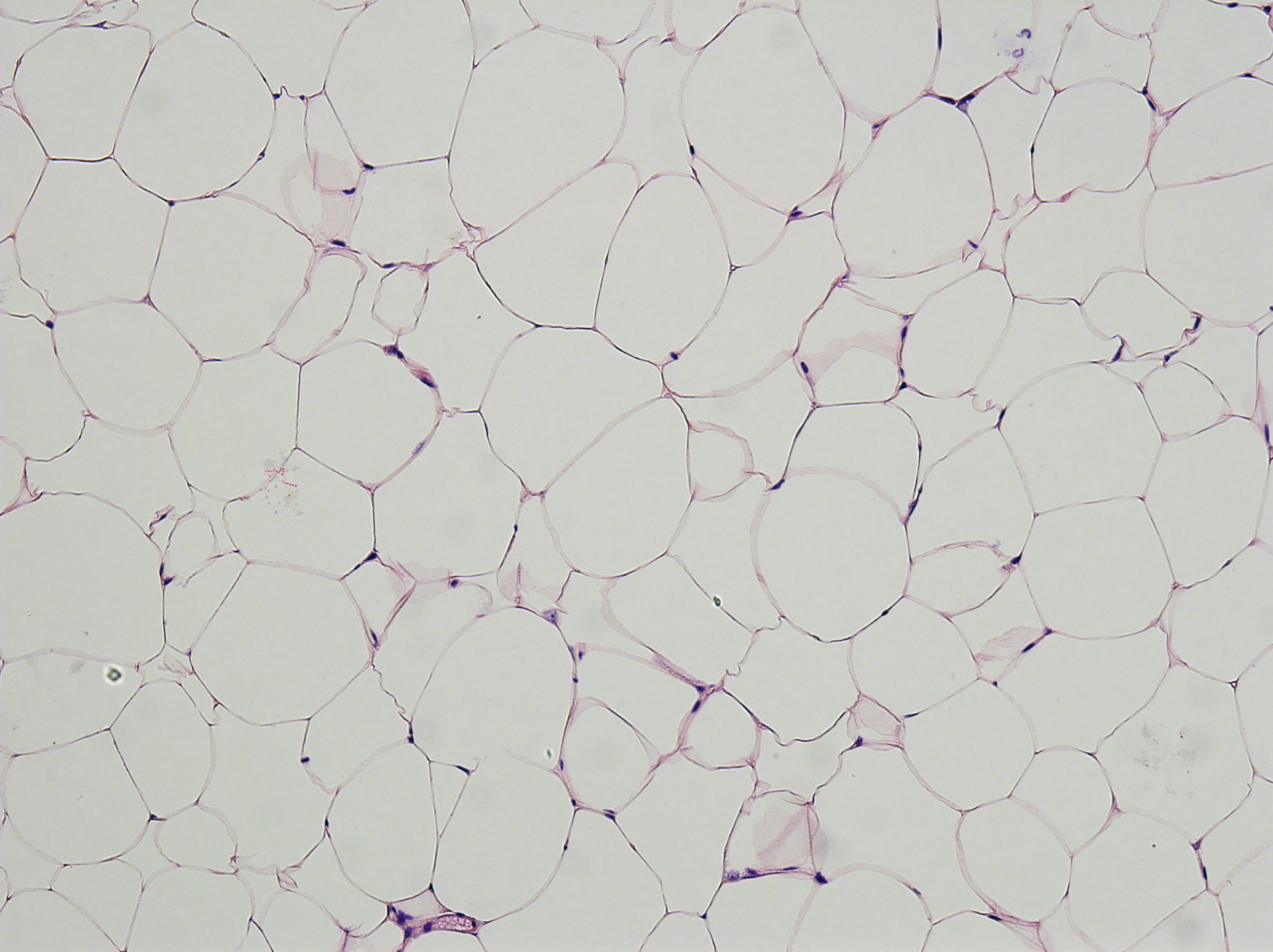

Supplement: Supplementary file 4 — Source data Fig. 2 [file 44318_2025_508_MOESM4_ESM.zip › Source data Fig.2/Figure 2G/iWAT-EP3FloxLysMCre.tif]

Figure 3E

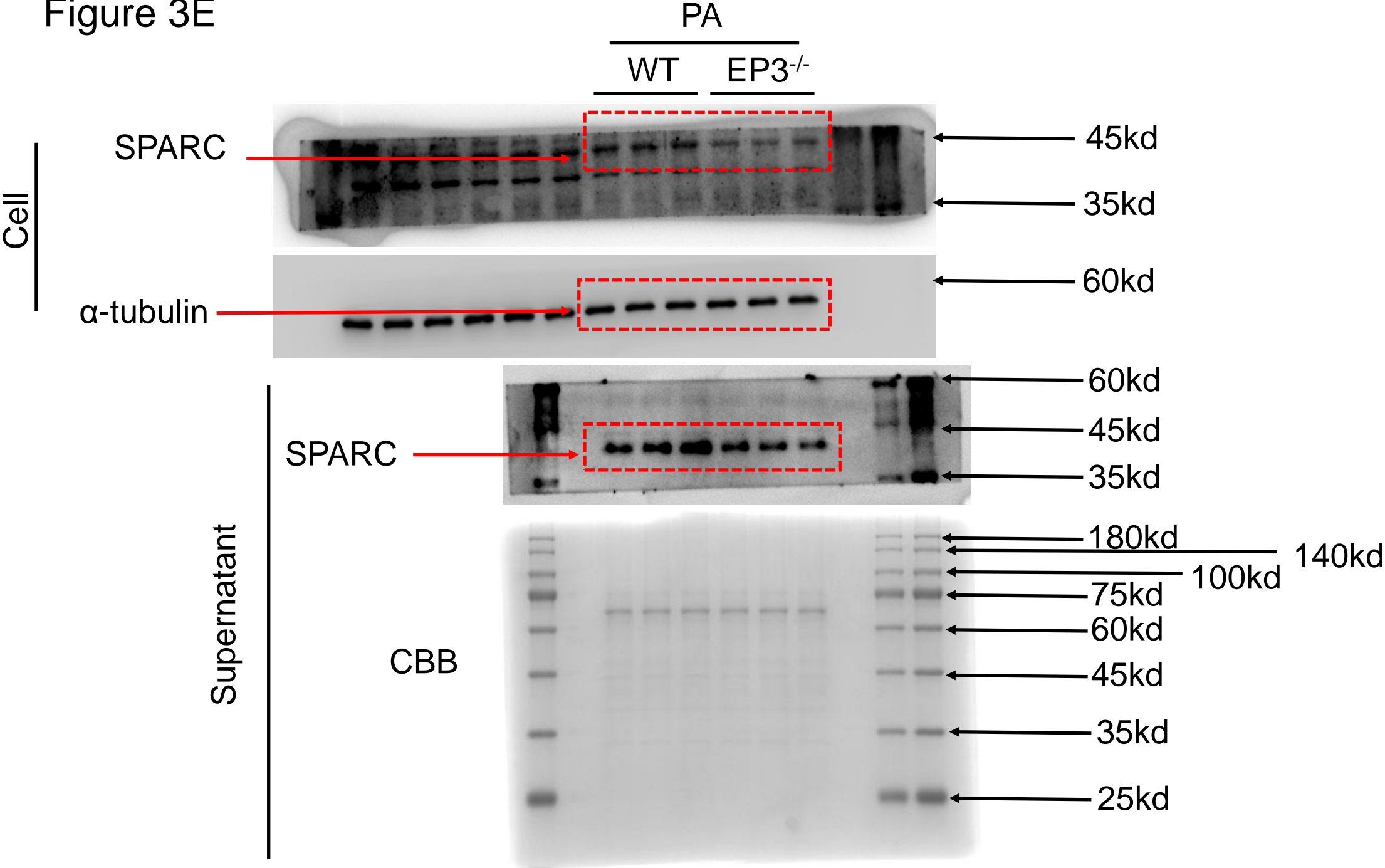

Supplement: Supplementary file 5 — Source data Fig. 3 [file 44318_2025_508_MOESM5_ESM.zip › Source data Fig.3/Figure 3E/Figure 3E.pdf]

Figure 3H

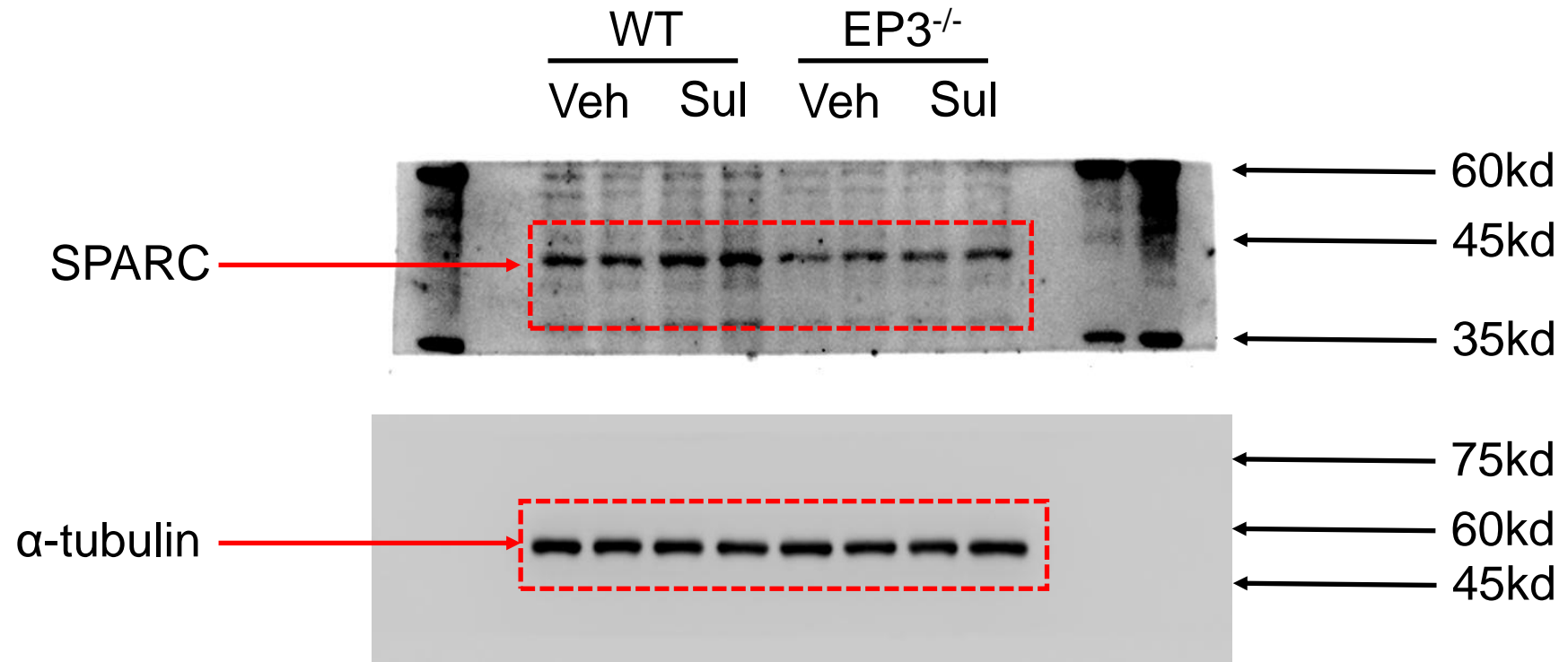

Supplement: Supplementary file 5 — Source data Fig. 3 [file 44318_2025_508_MOESM5_ESM.zip › Source data Fig.3/Figure 3H/Figure 3H.pdf]

Figure 3I

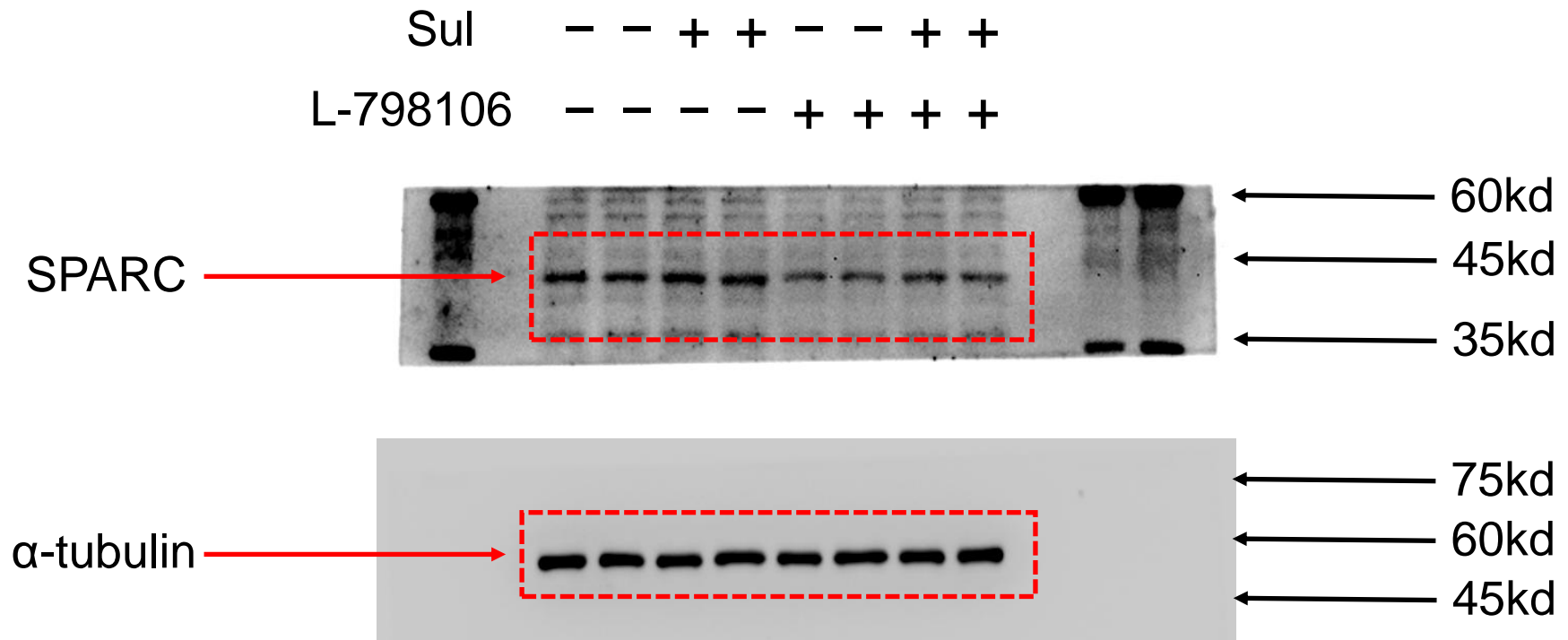

Supplement: Supplementary file 5 — Source data Fig. 3 [file 44318_2025_508_MOESM5_ESM.zip › Source data Fig.3/Figure 3I/Figure 3I.pdf]

Figure 3M

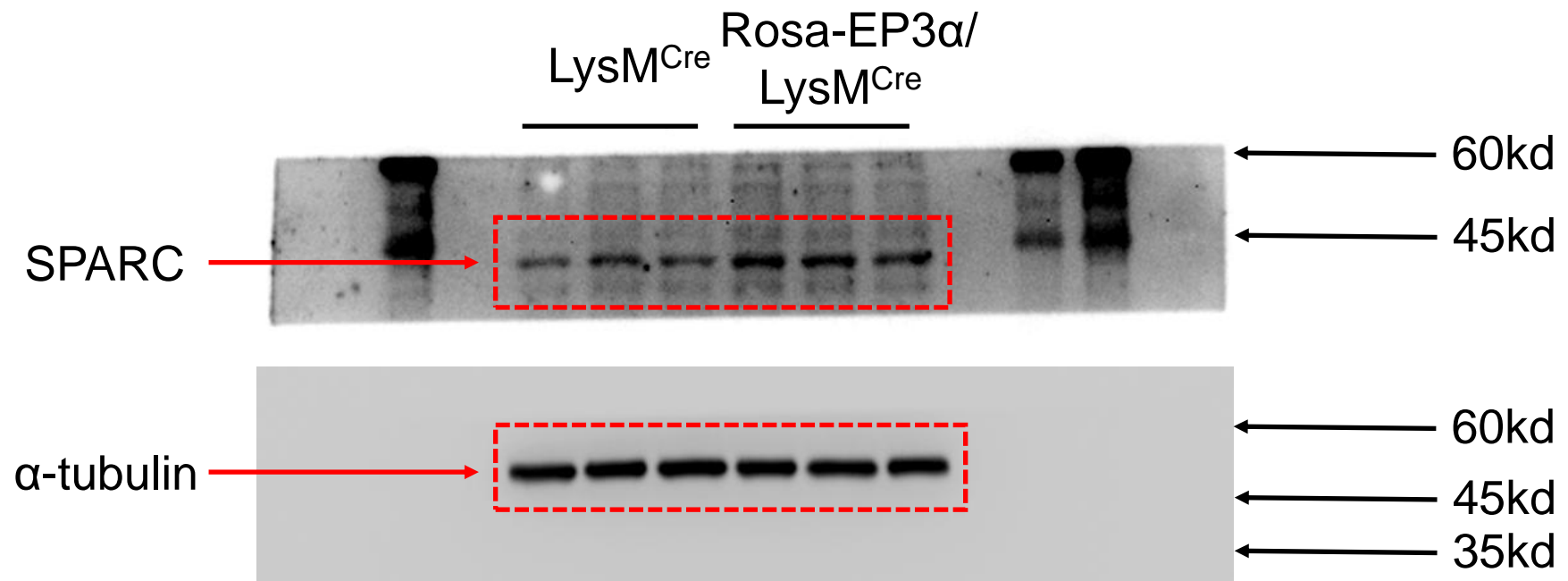

Supplement: Supplementary file 5 — Source data Fig. 3 [file 44318_2025_508_MOESM5_ESM.zip › Source data Fig.3/Figure 3M/Figure 3M.pdf]

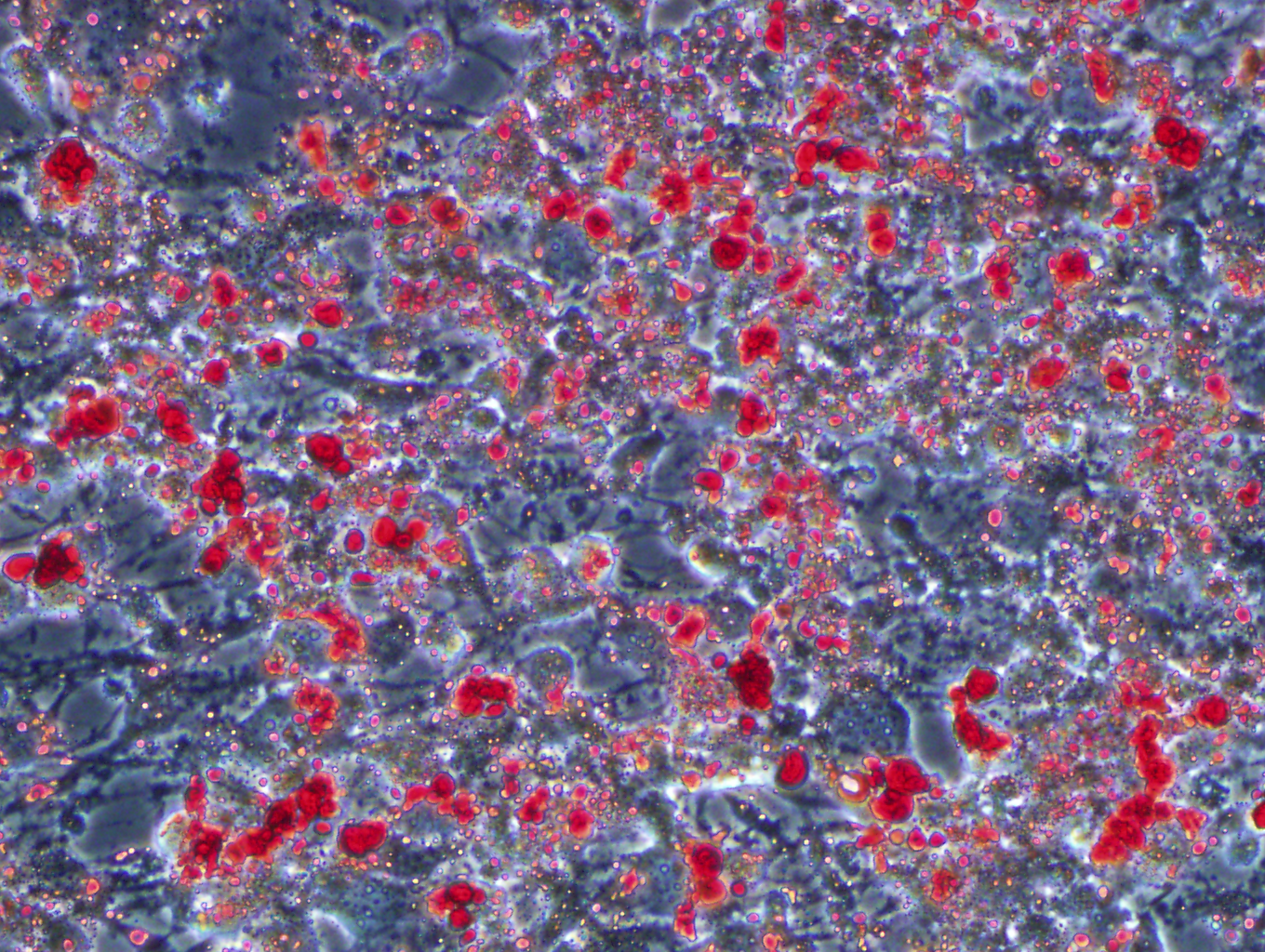

Supplement: Supplementary file 5 — Source data Fig. 3 [file 44318_2025_508_MOESM5_ESM.zip › Source data Fig.3/Figure 3O/Scram-LysMCre.tif]

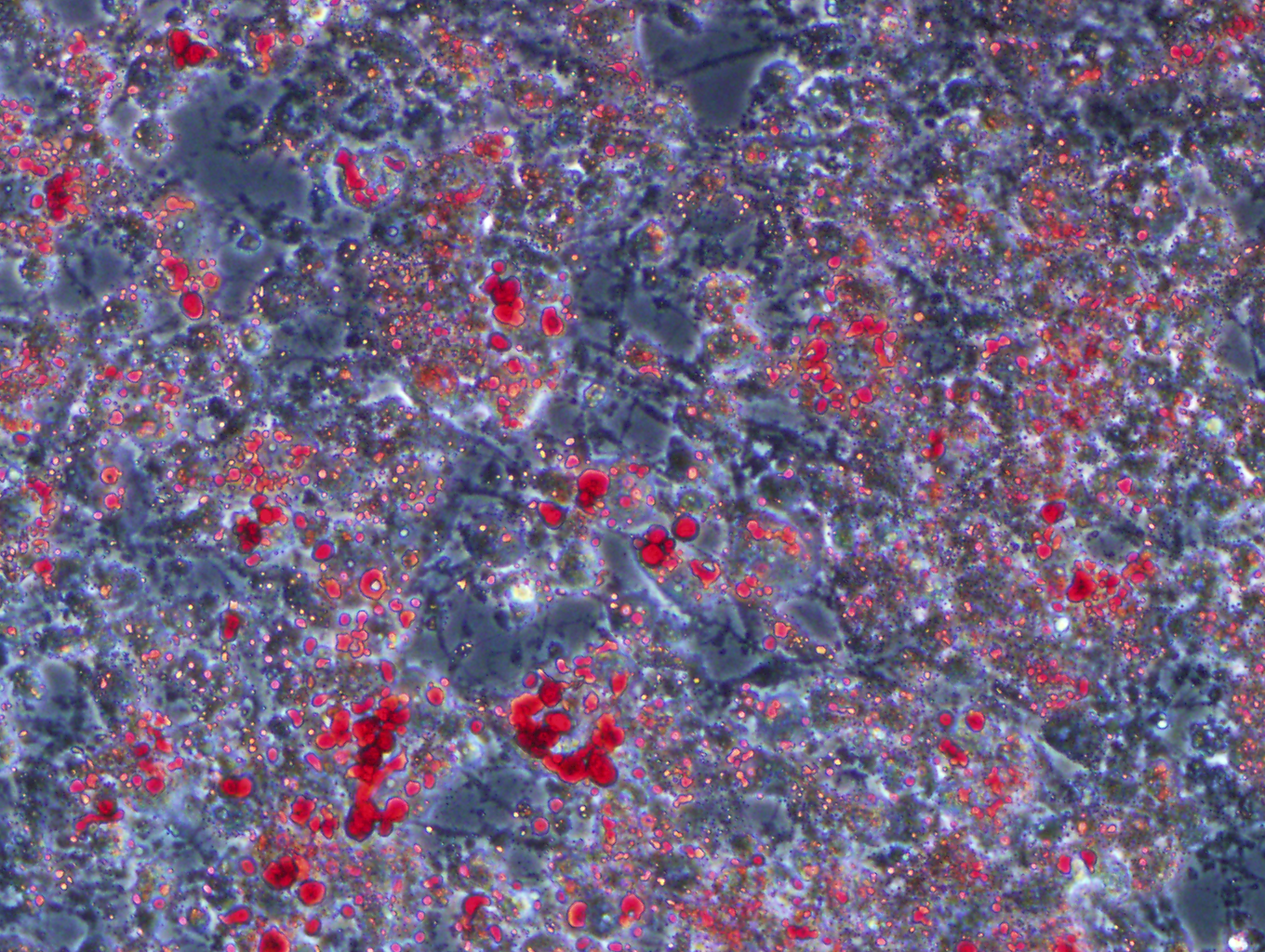

Supplement: Supplementary file 5 — Source data Fig. 3 [file 44318_2025_508_MOESM5_ESM.zip › Source data Fig.3/Figure 3O/Scram-Rosa-EP3α-LysMCre.tif]

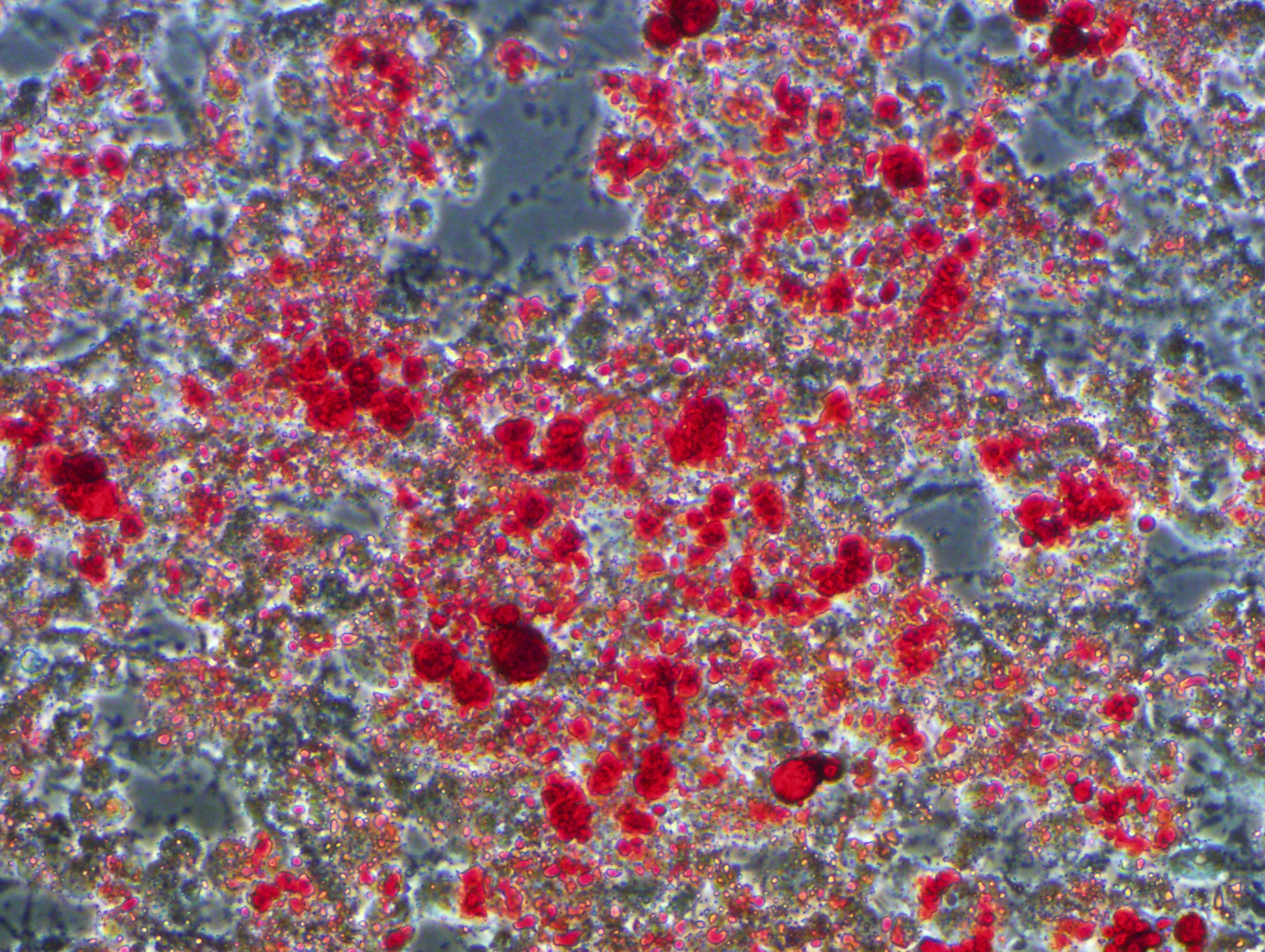

Supplement: Supplementary file 5 — Source data Fig. 3 [file 44318_2025_508_MOESM5_ESM.zip › Source data Fig.3/Figure 3O/siSPARC-LysMCre.tif]

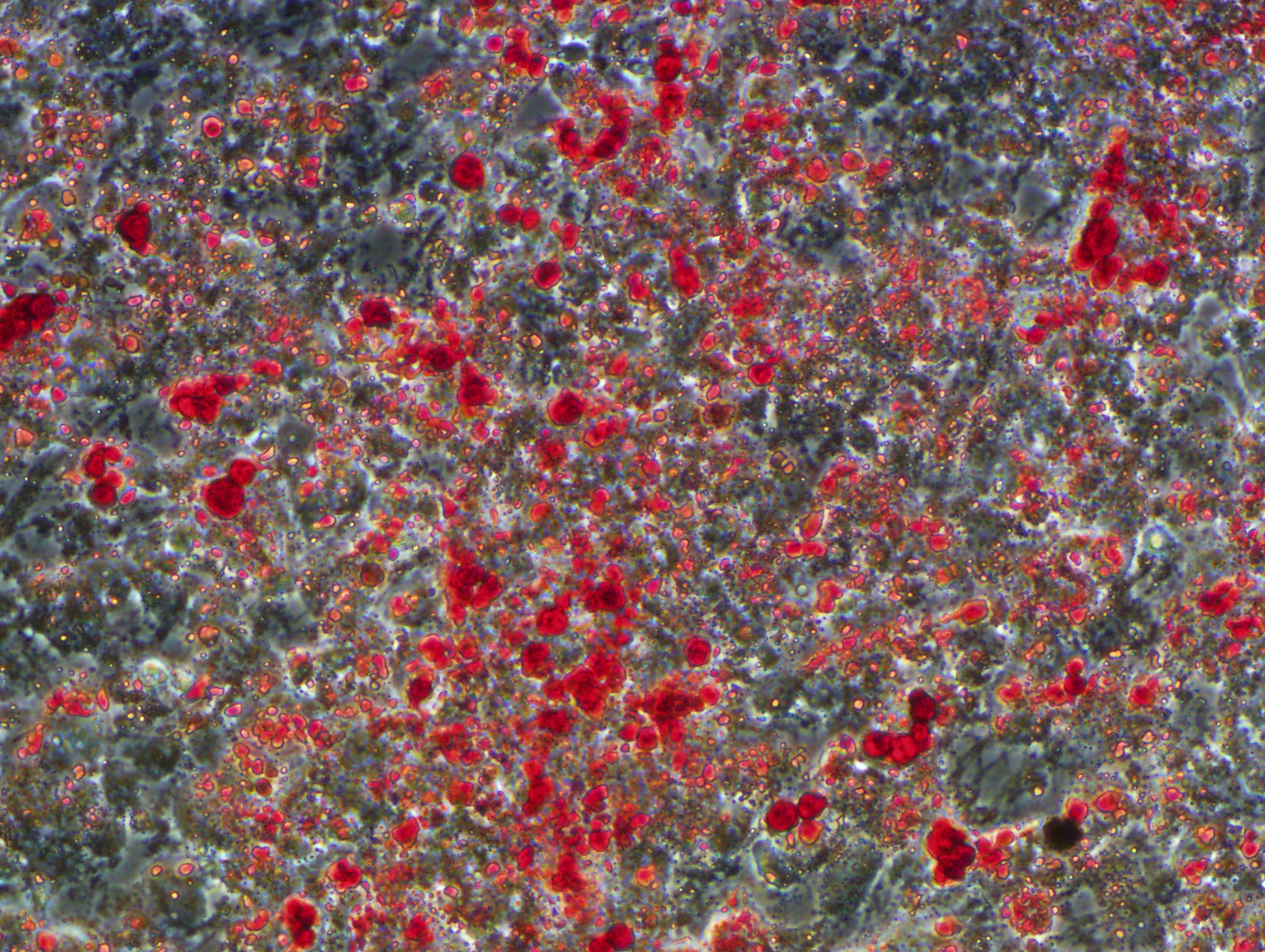

Supplement: Supplementary file 5 — Source data Fig. 3 [file 44318_2025_508_MOESM5_ESM.zip › Source data Fig.3/Figure 3O/siSPARC-Rosa-EP3α-LysMCre.tif]

Figure 3T

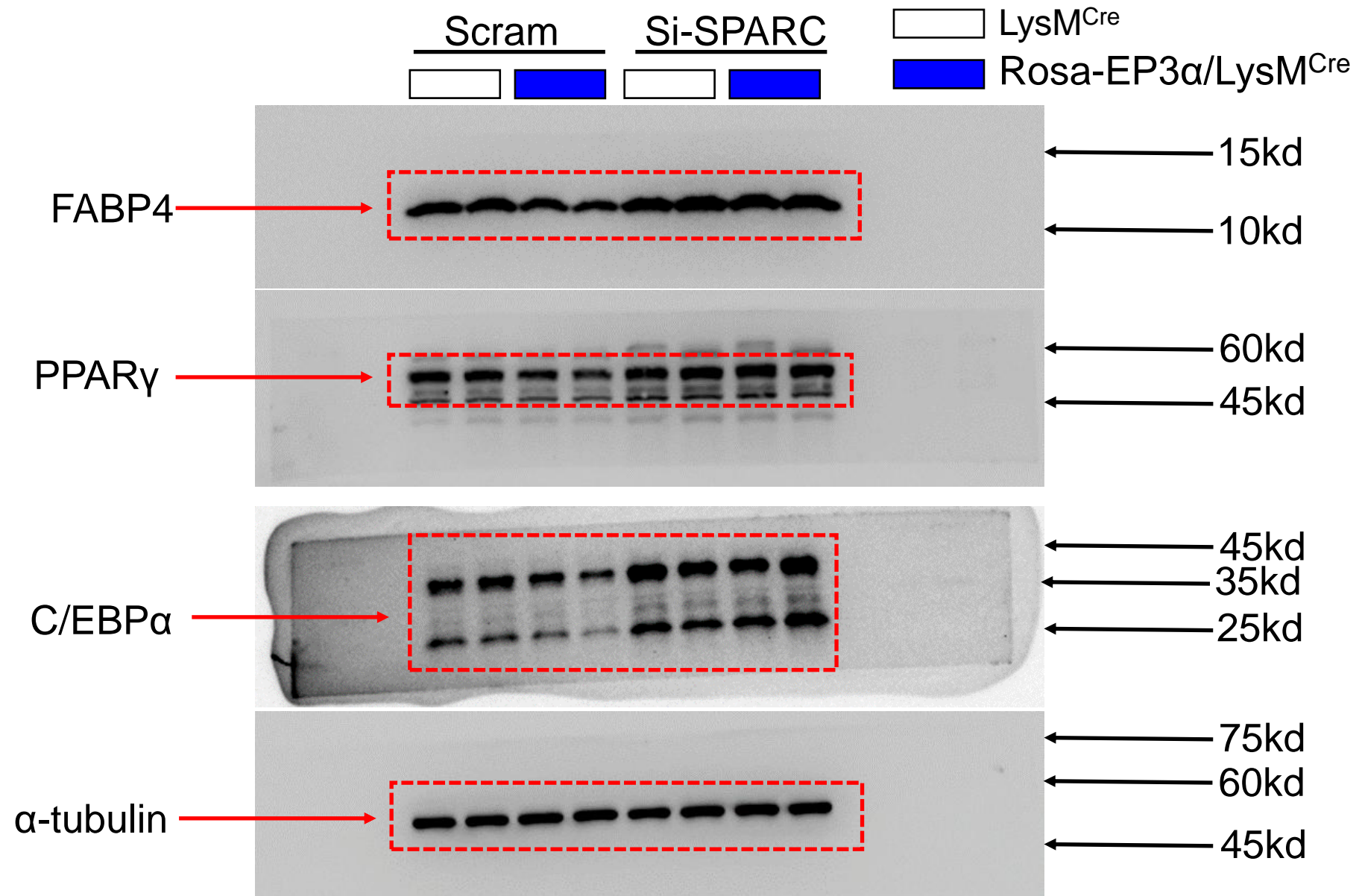

Supplement: Supplementary file 5 — Source data Fig. 3 [file 44318_2025_508_MOESM5_ESM.zip › Source data Fig.3/Figure 3T/Figure 3T.pdf]

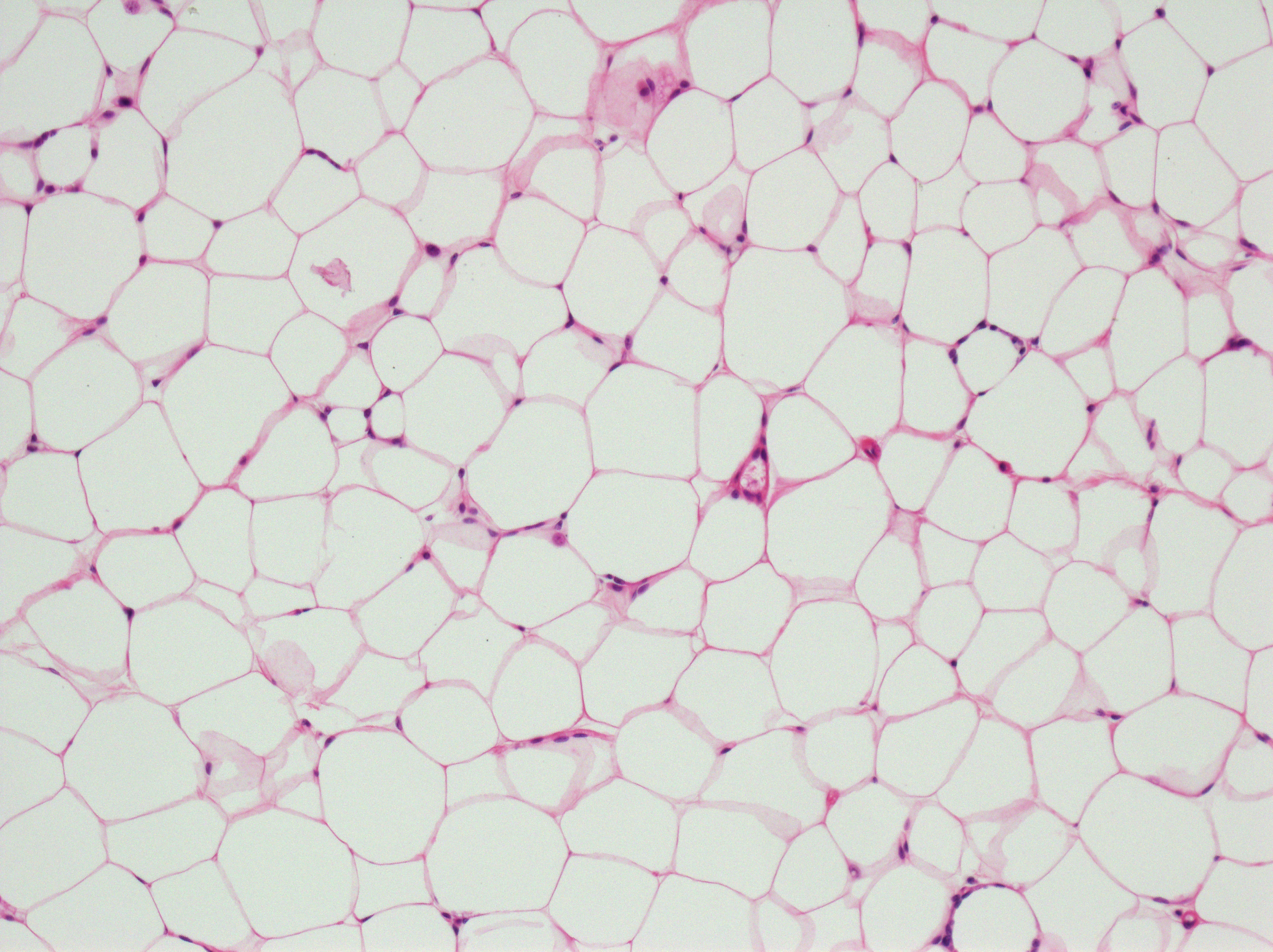

Supplement: Supplementary file 6 — Source data Fig. 4 [file 44318_2025_508_MOESM6_ESM.zip › Source data Fig.4/Figrue 4G/eWAT-EP3RosaLysMCre.tif]

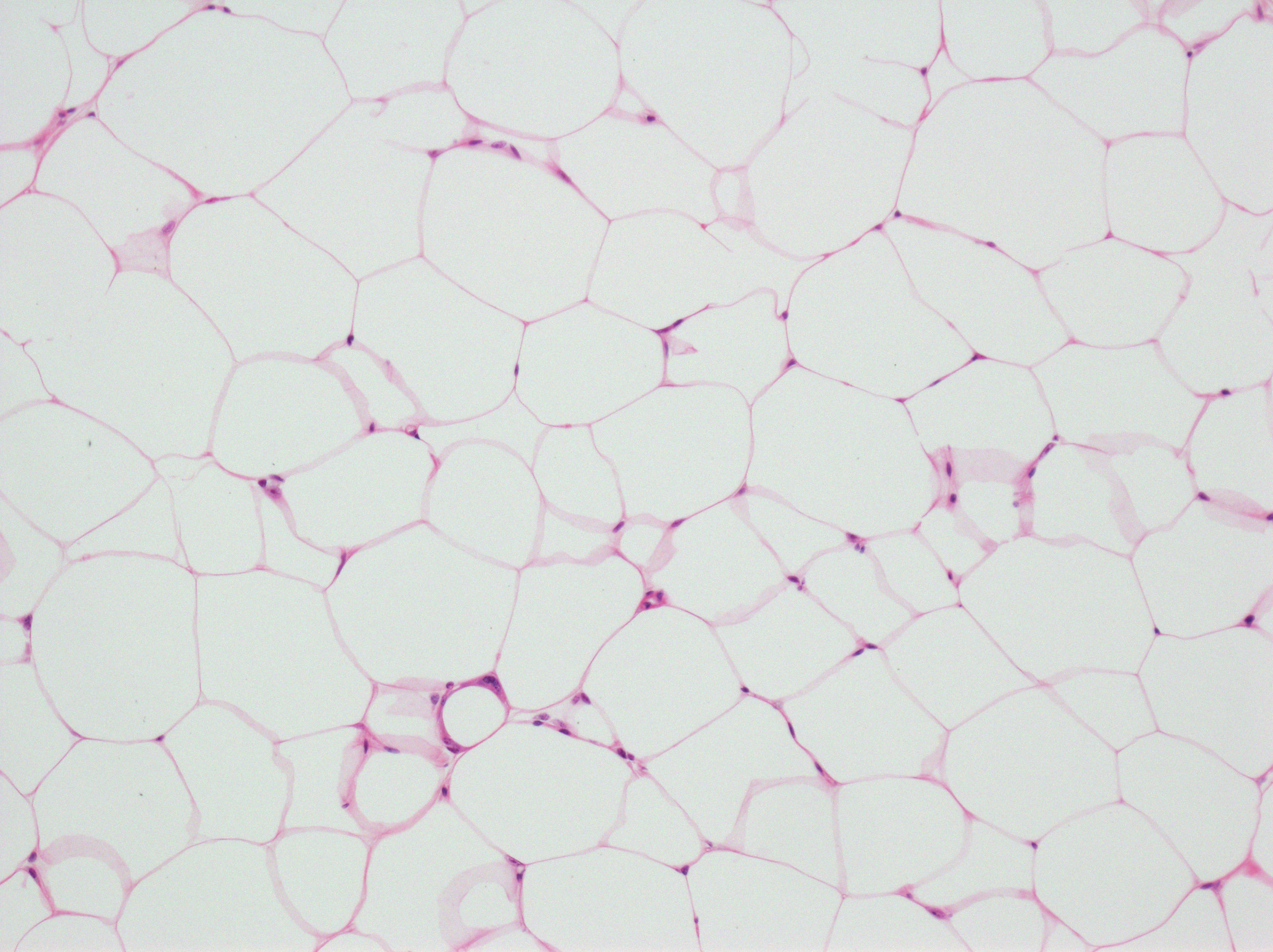

Supplement: Supplementary file 6 — Source data Fig. 4 [file 44318_2025_508_MOESM6_ESM.zip › Source data Fig.4/Figrue 4G/eWAT-EP3RosaSPARCFloxLysMCre.tif]

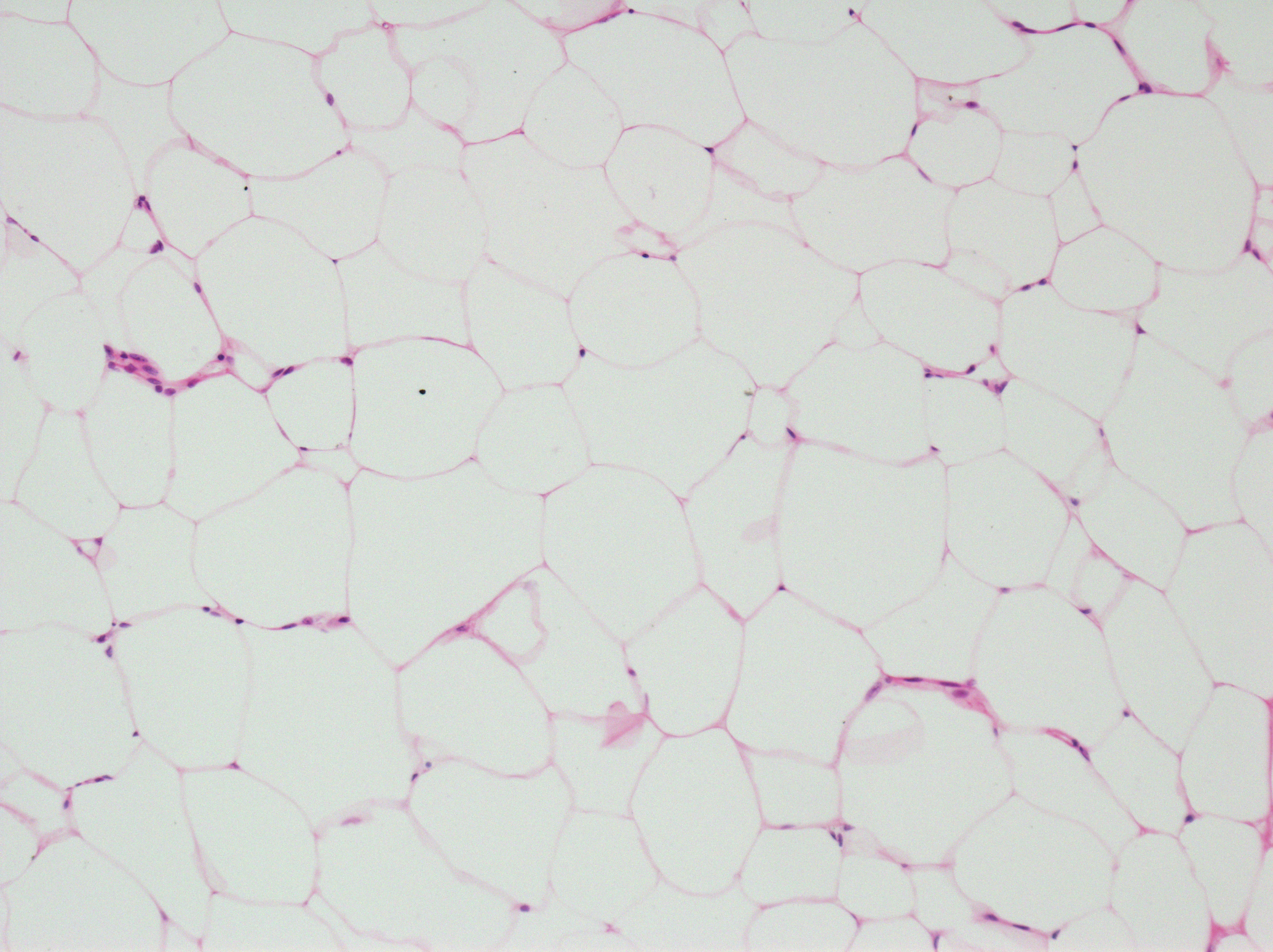

Supplement: Supplementary file 6 — Source data Fig. 4 [file 44318_2025_508_MOESM6_ESM.zip › Source data Fig.4/Figrue 4G/eWAT-LysMCre.tif]

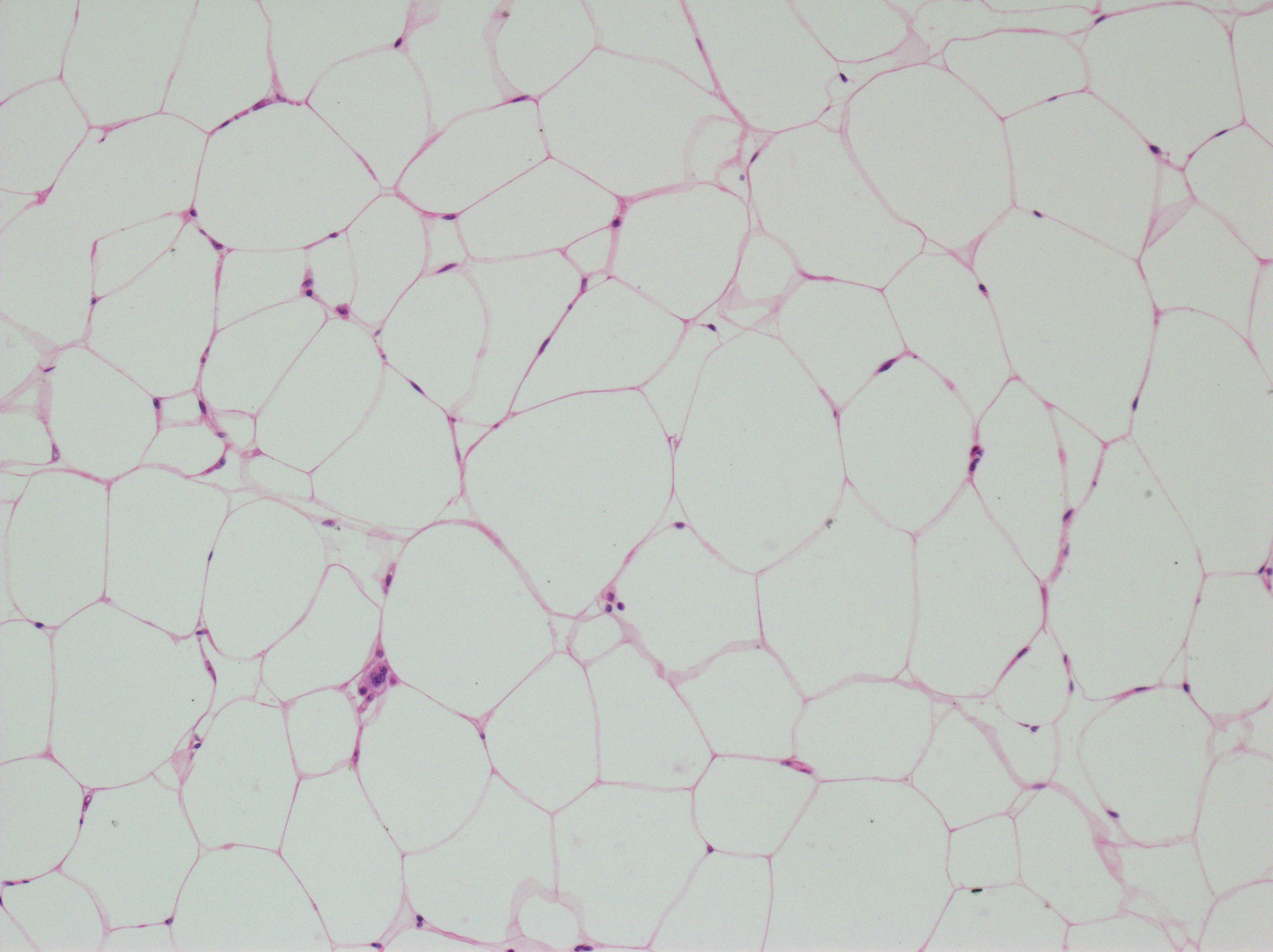

Supplement: Supplementary file 6 — Source data Fig. 4 [file 44318_2025_508_MOESM6_ESM.zip › Source data Fig.4/Figrue 4G/eWAT-SPARCFloxLysMCre.tif]

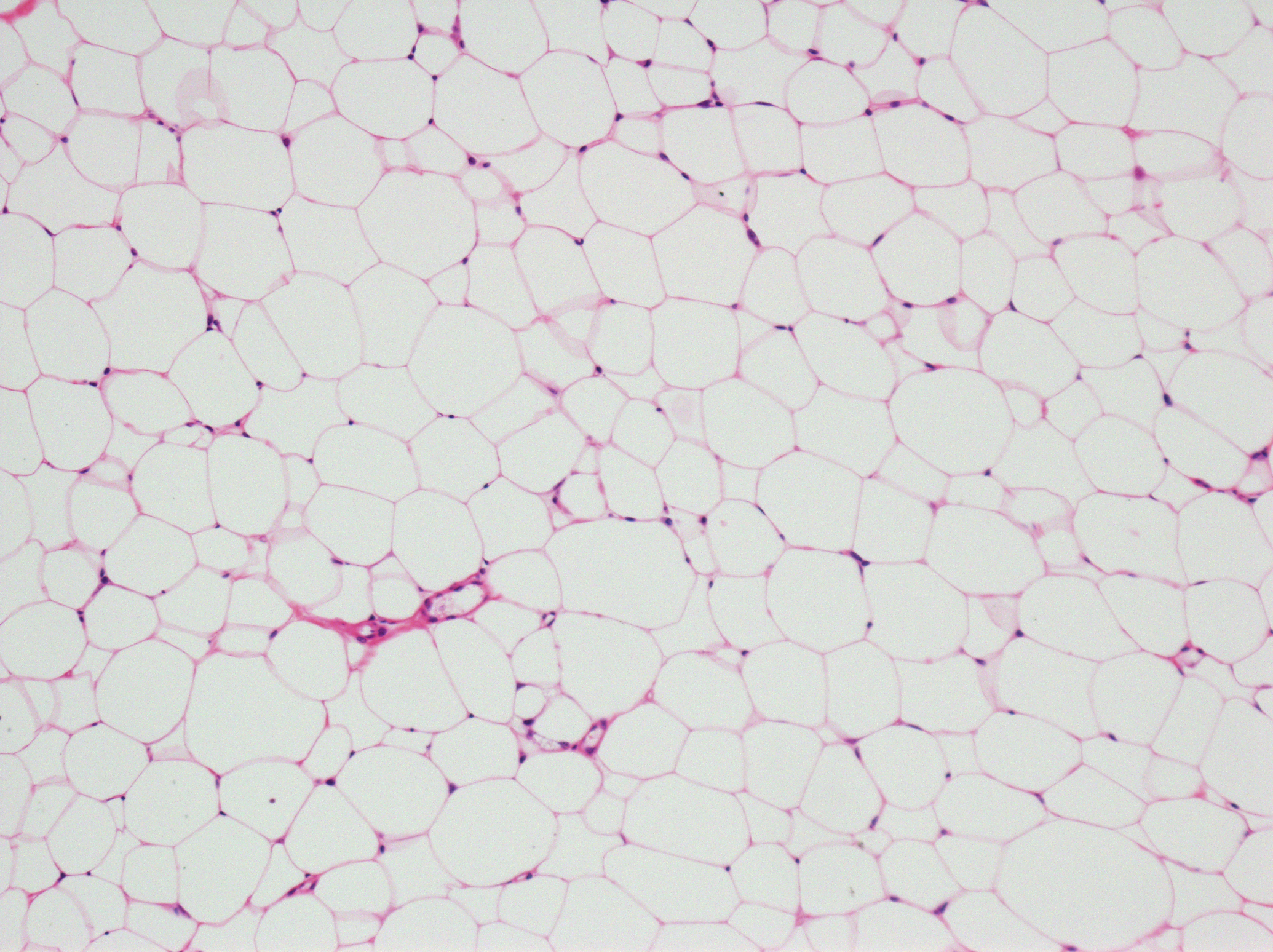

Supplement: Supplementary file 6 — Source data Fig. 4 [file 44318_2025_508_MOESM6_ESM.zip › Source data Fig.4/Figrue 4G/iWAT-EP3RosaLysMCre.tif]

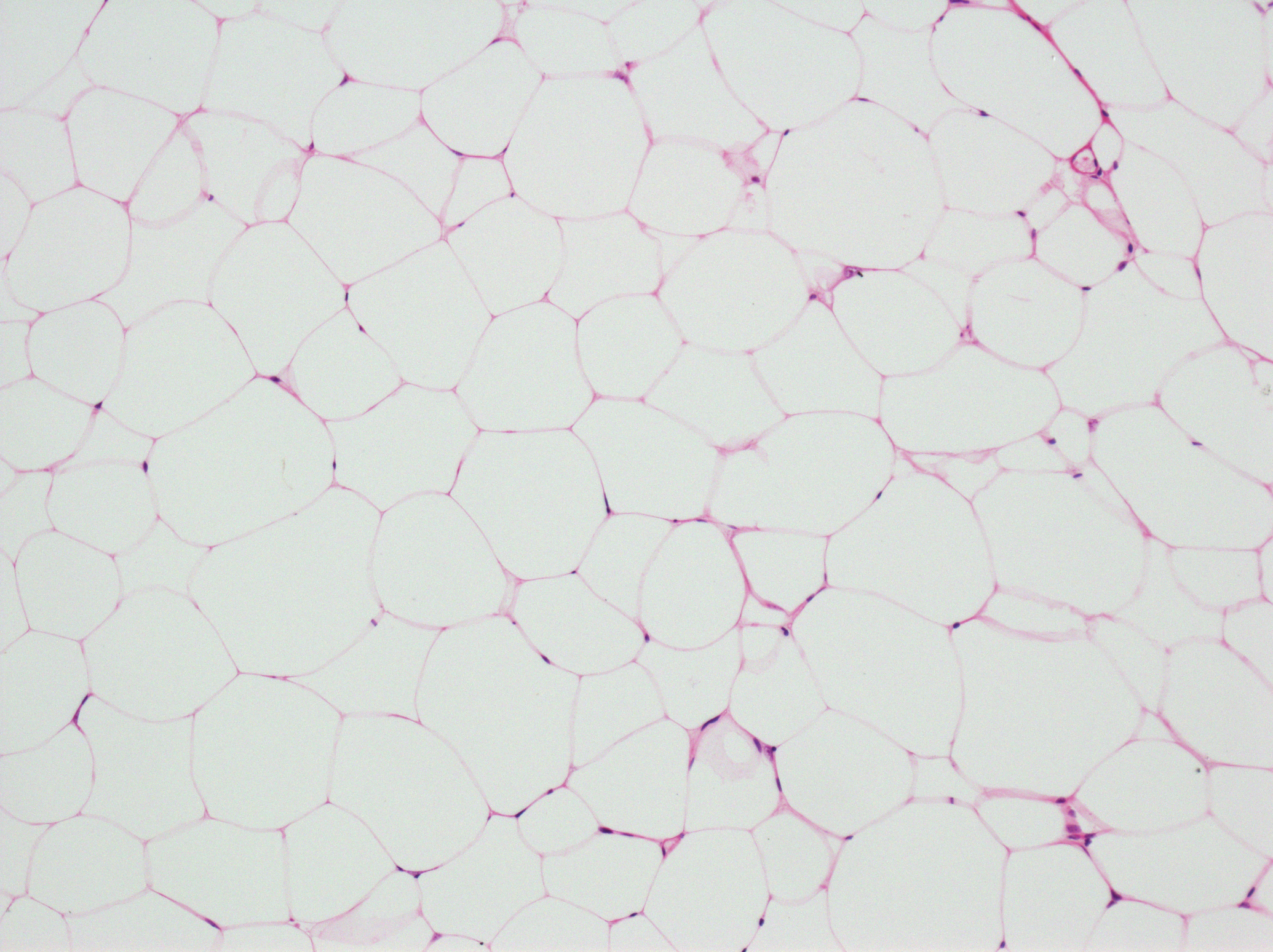

Supplement: Supplementary file 6 — Source data Fig. 4 [file 44318_2025_508_MOESM6_ESM.zip › Source data Fig.4/Figrue 4G/iWAT-EP3RosaSPARCFloxLysMCre.tif]

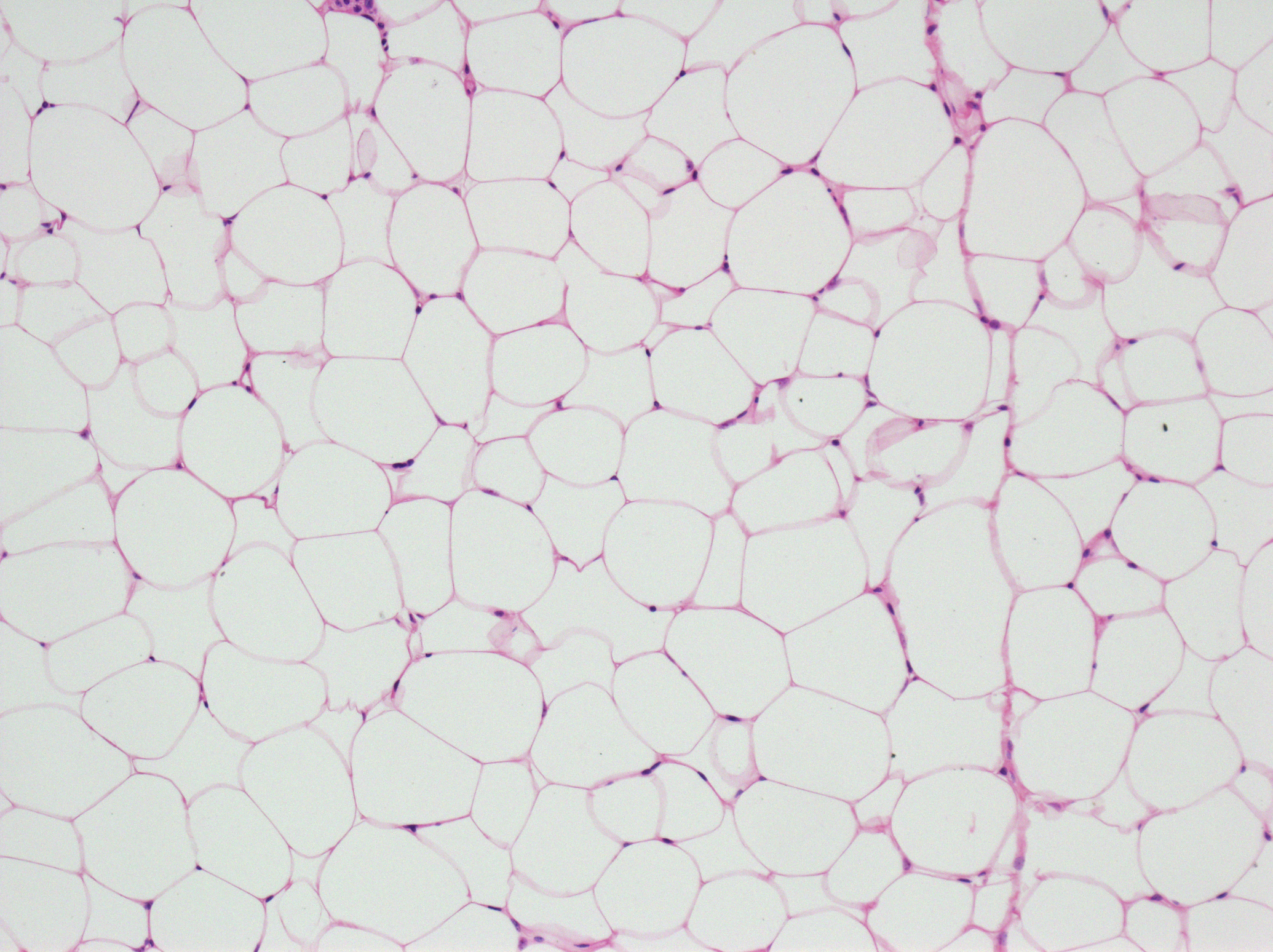

Supplement: Supplementary file 6 — Source data Fig. 4 [file 44318_2025_508_MOESM6_ESM.zip › Source data Fig.4/Figrue 4G/iWAT-LysMCre.tif]

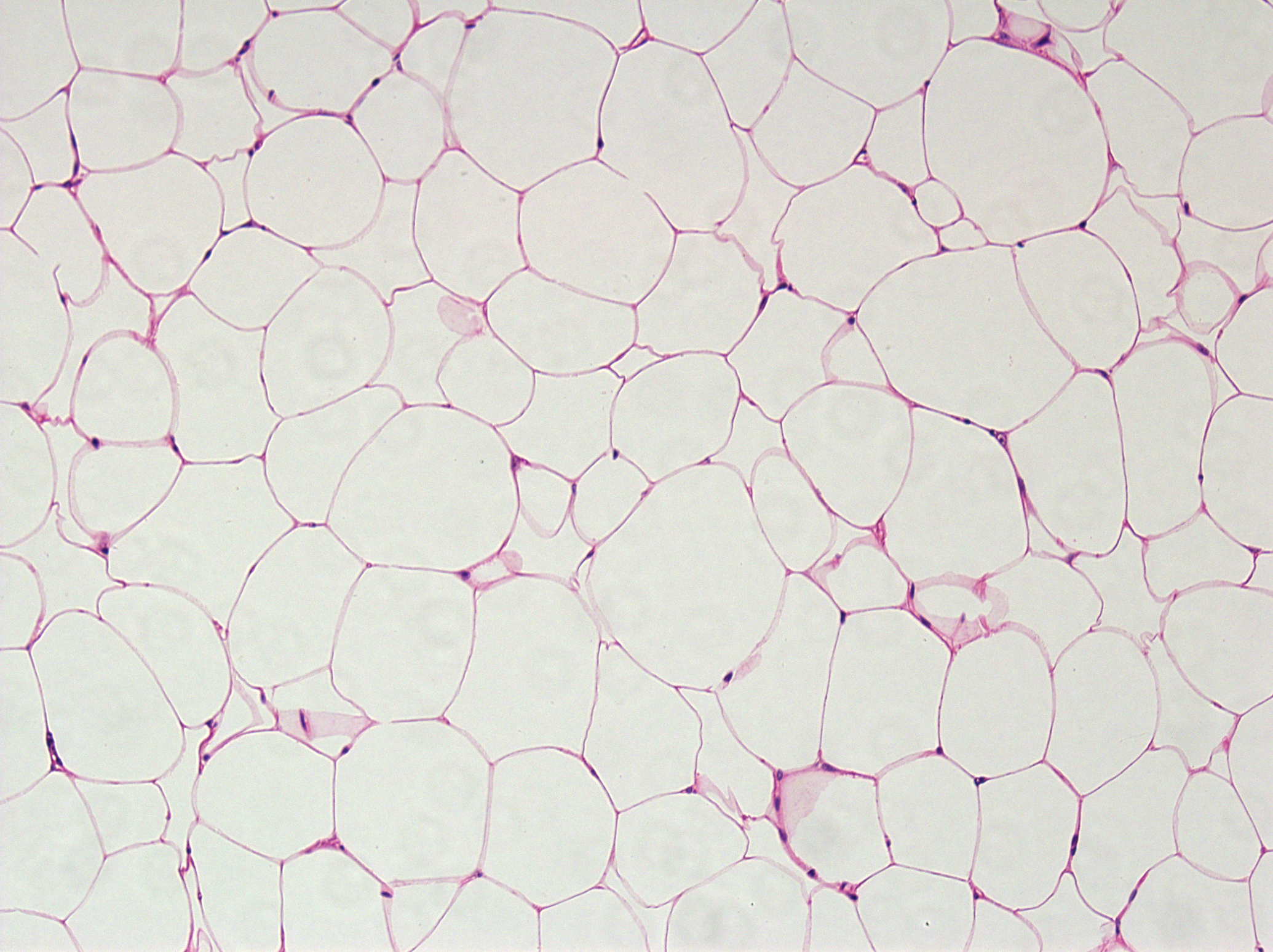

Supplement: Supplementary file 6 — Source data Fig. 4 [file 44318_2025_508_MOESM6_ESM.zip › Source data Fig.4/Figrue 4G/iWAT-SPARCFloxLysMCre.tif]

Figure 5E

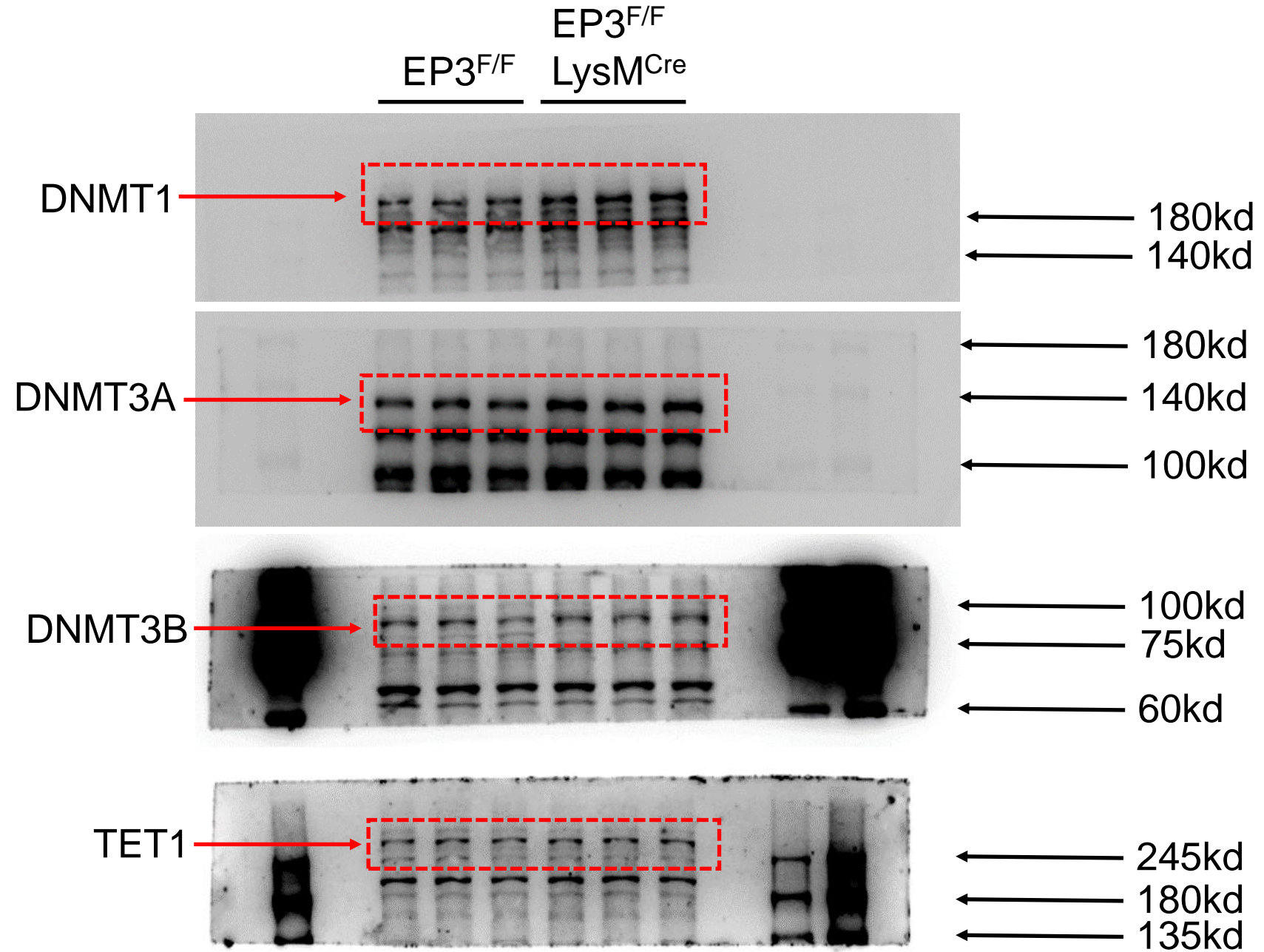

Figure 5E

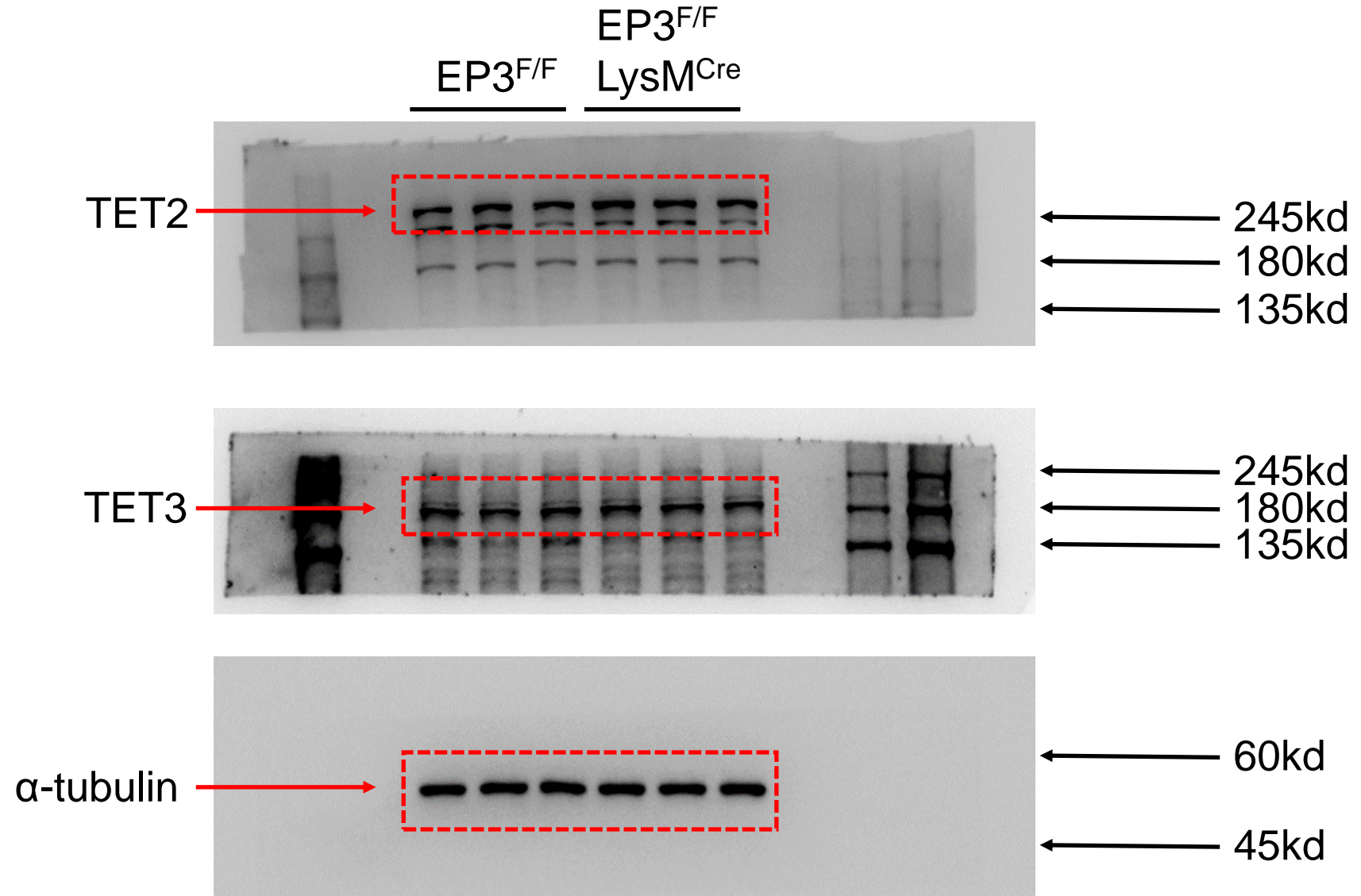

Supplement: Supplementary file 7 — Source data Fig. 5 [file 44318_2025_508_MOESM7_ESM.zip › Source data Fig.5/Figure 5E/Figure 5E.pdf]

Figure 5G

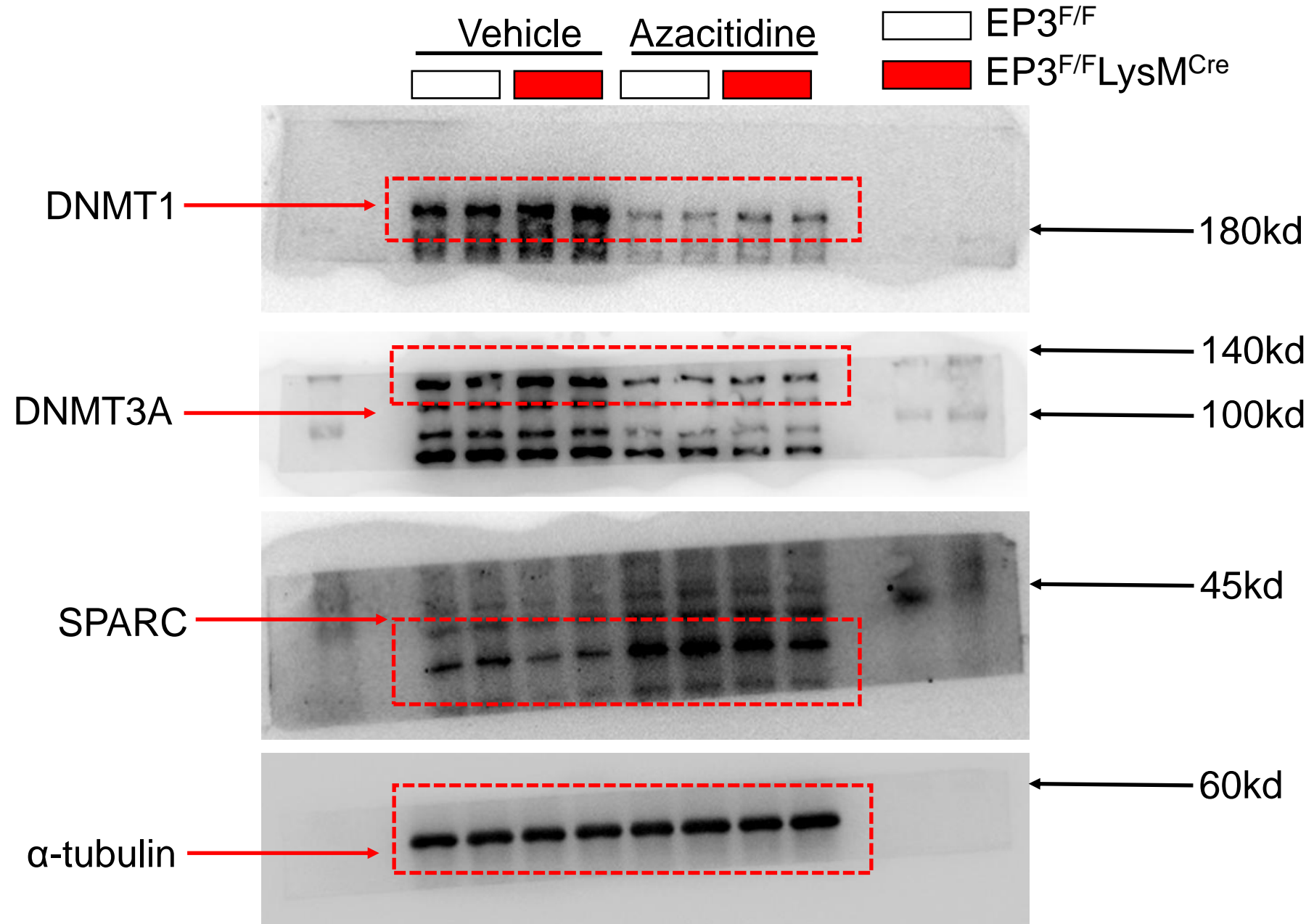

Supplement: Supplementary file 7 — Source data Fig. 5 [file 44318_2025_508_MOESM7_ESM.zip › Source data Fig.5/Figure 5G/Figure 5G.pdf]

Figure 5H

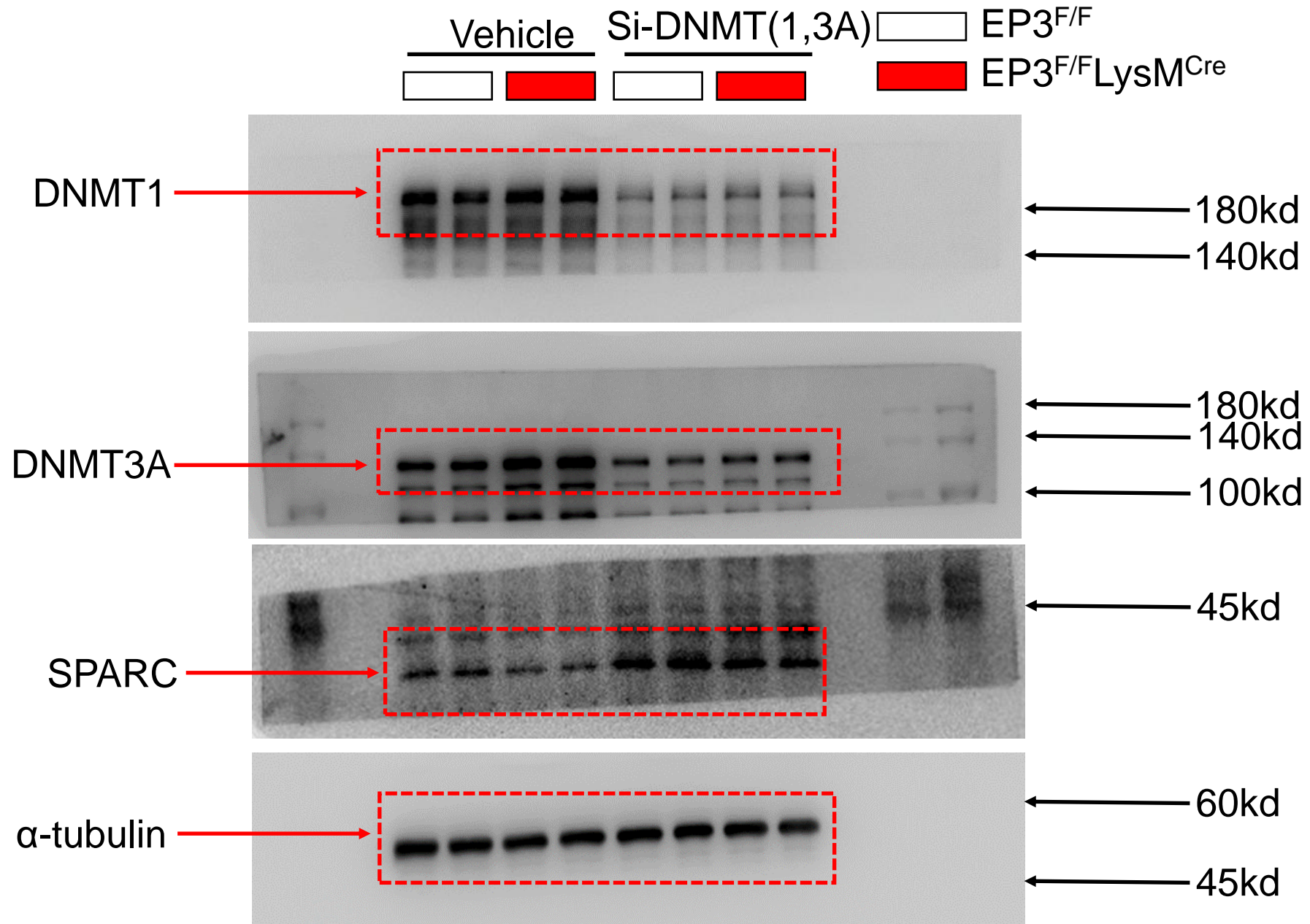

Supplement: Supplementary file 7 — Source data Fig. 5 [file 44318_2025_508_MOESM7_ESM.zip › Source data Fig.5/Figure 5H/Figure 5H.pdf]

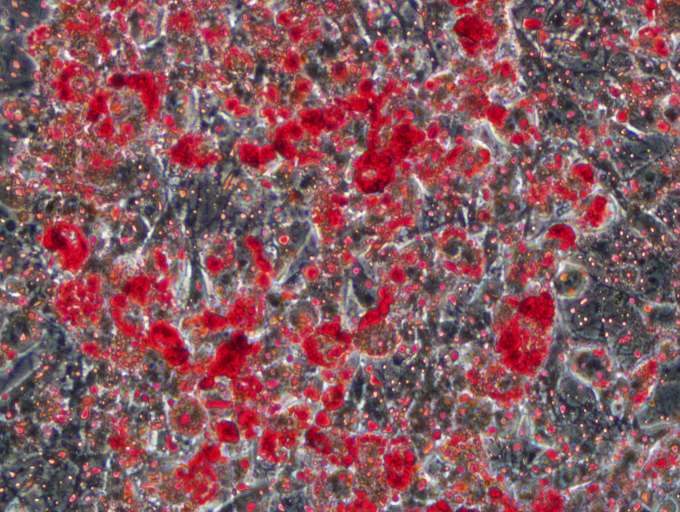

Supplement: Supplementary file 7 — Source data Fig. 5 [file 44318_2025_508_MOESM7_ESM.zip › Source data Fig.5/Figure 5I/Scram-EP3Flox.tif]

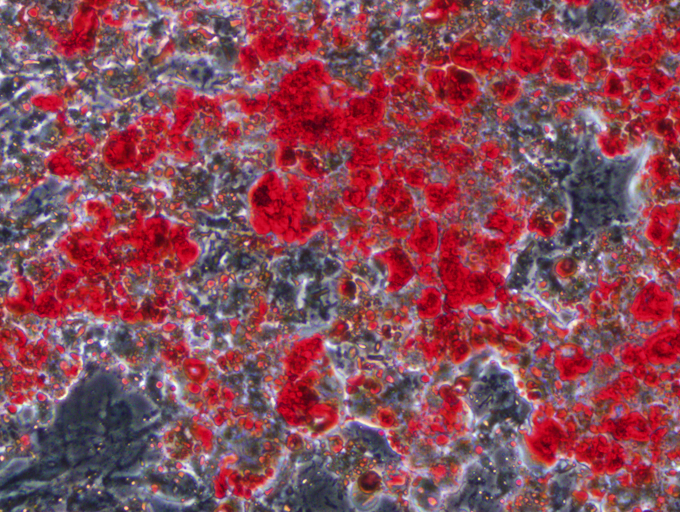

Supplement: Supplementary file 7 — Source data Fig. 5 [file 44318_2025_508_MOESM7_ESM.zip › Source data Fig.5/Figure 5I/Scram-EP3FloxLysMCre.tif]

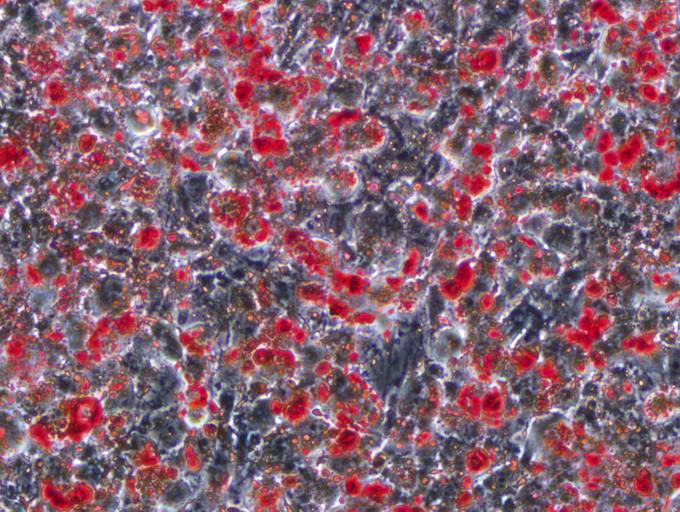

Supplement: Supplementary file 7 — Source data Fig. 5 [file 44318_2025_508_MOESM7_ESM.zip › Source data Fig.5/Figure 5I/Si-Dnmt1-3a-EP3Flox.tif]

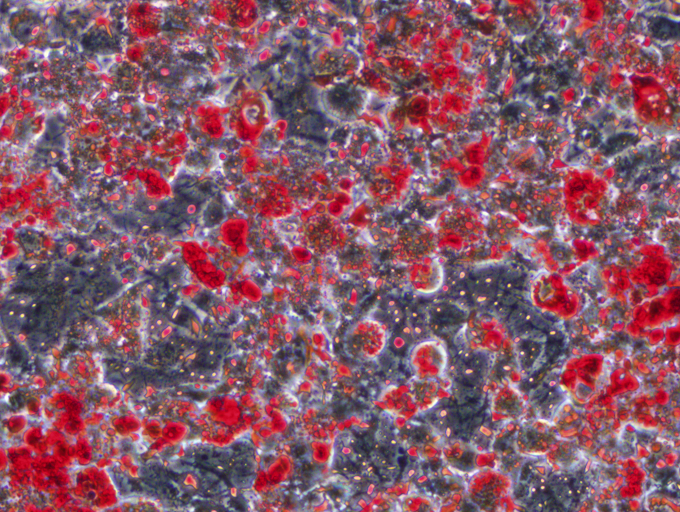

Supplement: Supplementary file 7 — Source data Fig. 5 [file 44318_2025_508_MOESM7_ESM.zip › Source data Fig.5/Figure 5I/Si-Dnmt1-3a-EP3FloxLysMCre.tif]

Figure 5N

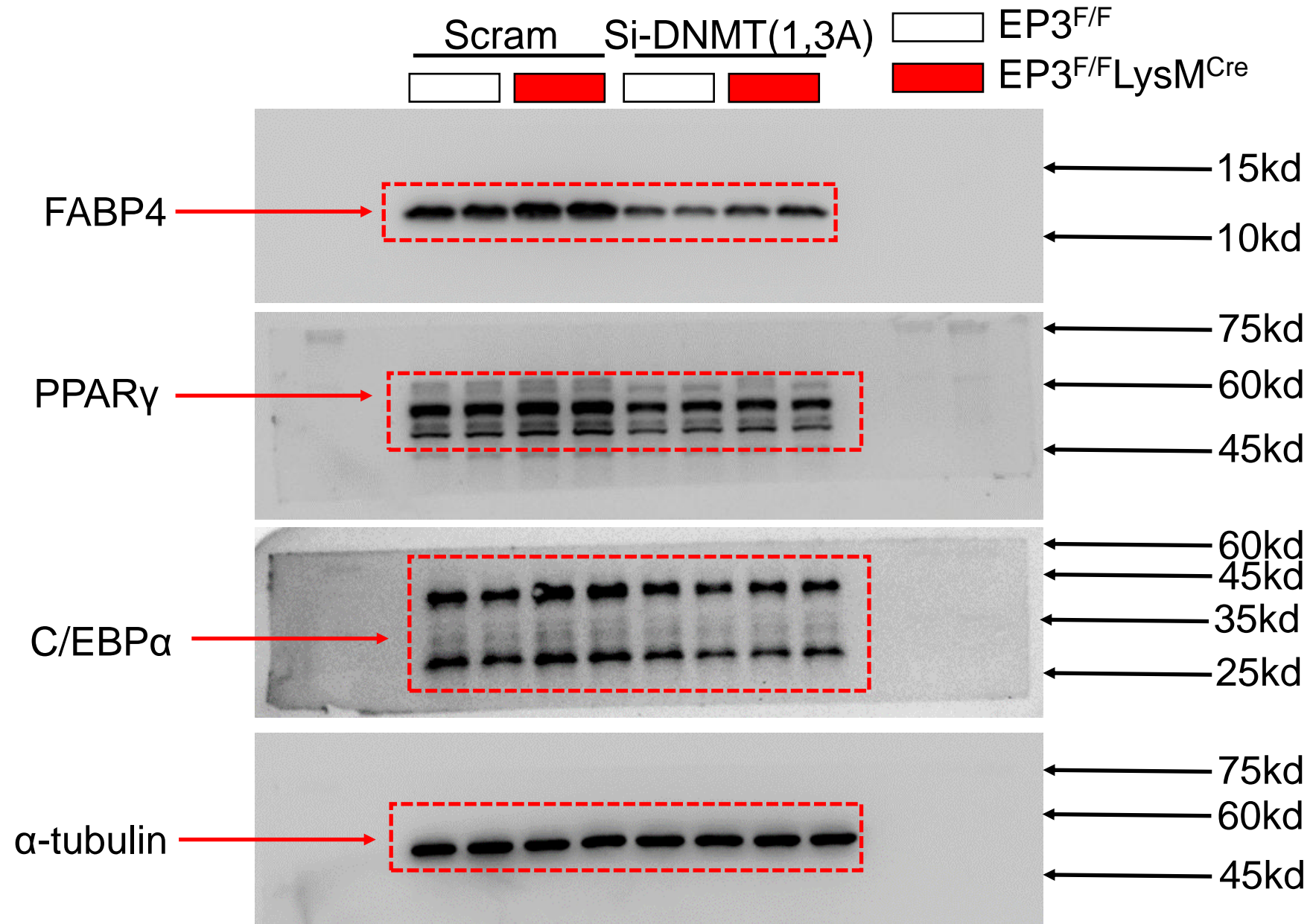

Supplement: Supplementary file 7 — Source data Fig. 5 [file 44318_2025_508_MOESM7_ESM.zip › Source data Fig.5/Figure 5N/Figure 5N.pdf]

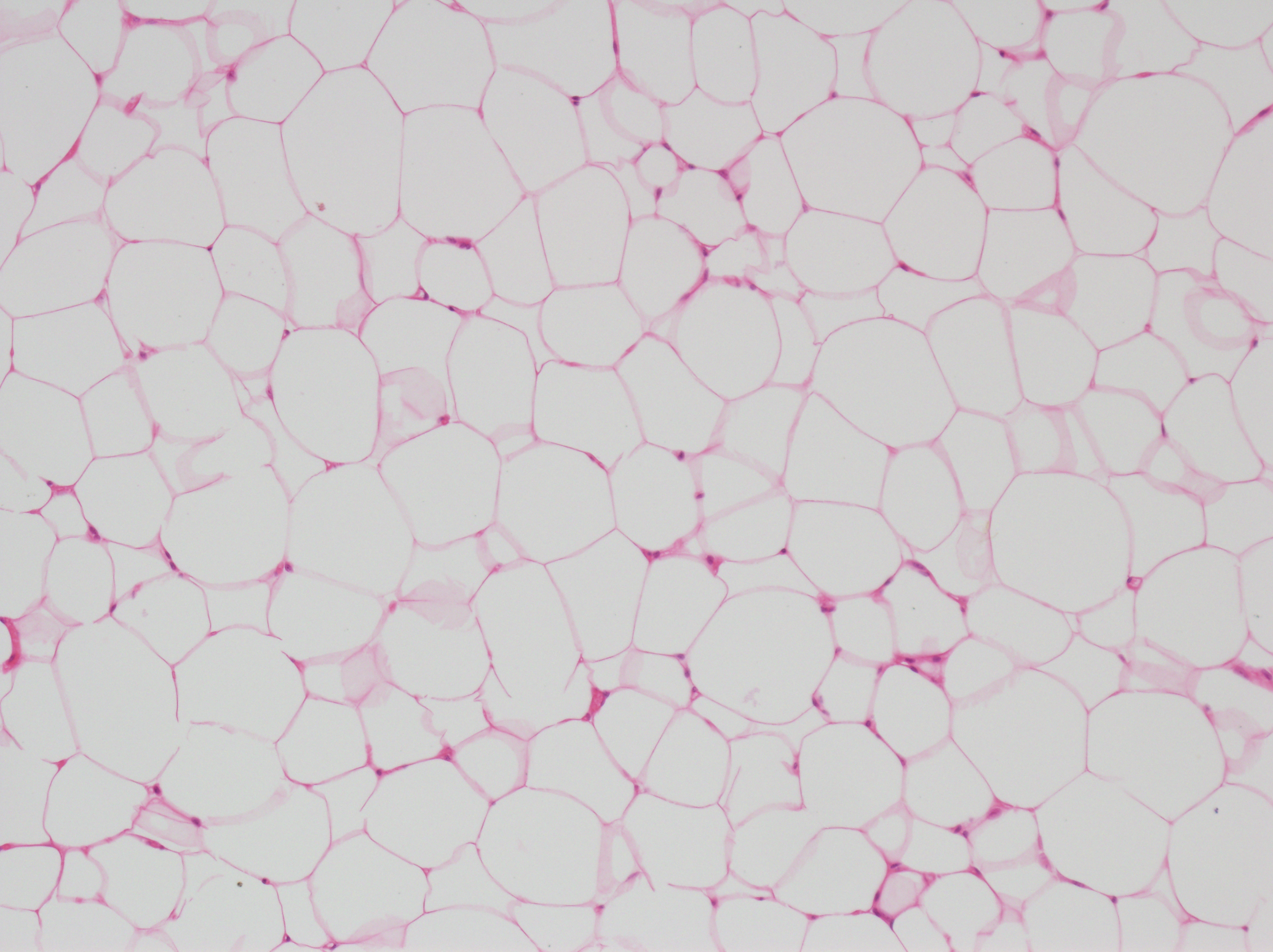

Supplement: Supplementary file 8 — Source data Fig. 6 [file 44318_2025_508_MOESM8_ESM.zip › Source data Fig.6/Figure 6G/eWAT-Dnmt1-3aFloxLysMCre.tif]

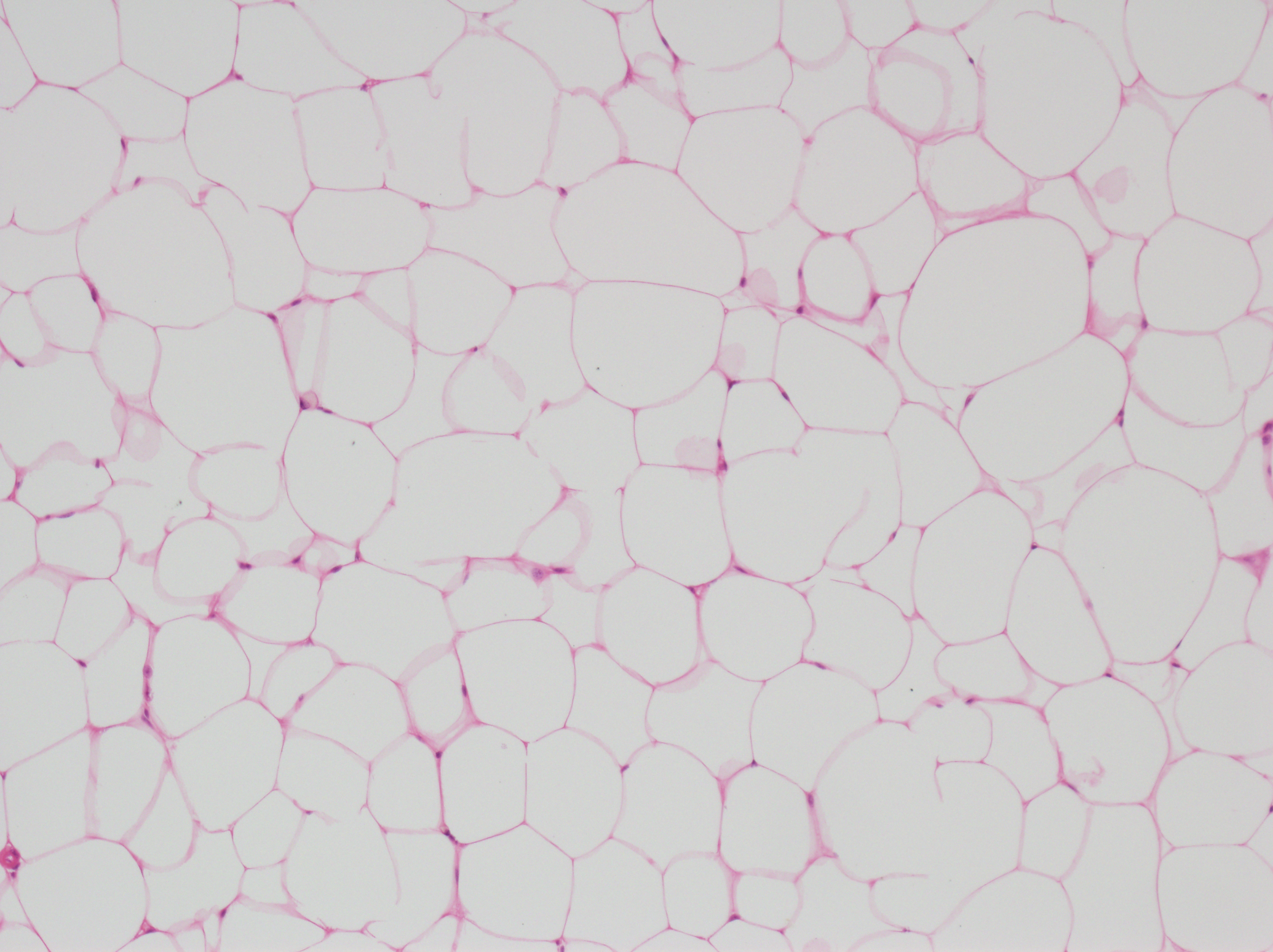

Supplement: Supplementary file 8 — Source data Fig. 6 [file 44318_2025_508_MOESM8_ESM.zip › Source data Fig.6/Figure 6G/eWAT-EP3FloxDnmt1-3aFloxLysMCre.tif]

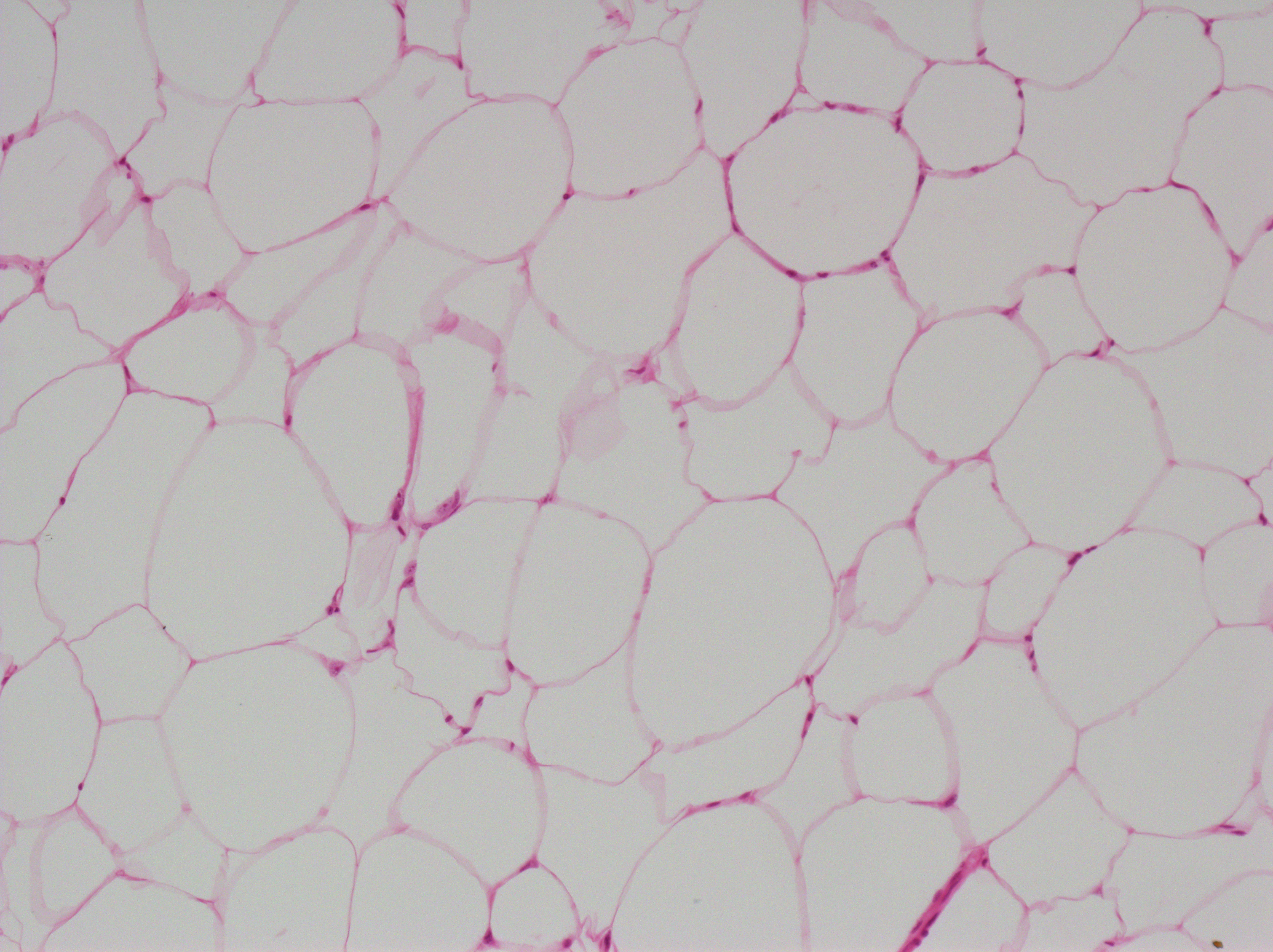

Supplement: Supplementary file 8 — Source data Fig. 6 [file 44318_2025_508_MOESM8_ESM.zip › Source data Fig.6/Figure 6G/eWAT-EP3FloxLysMCre.tif]

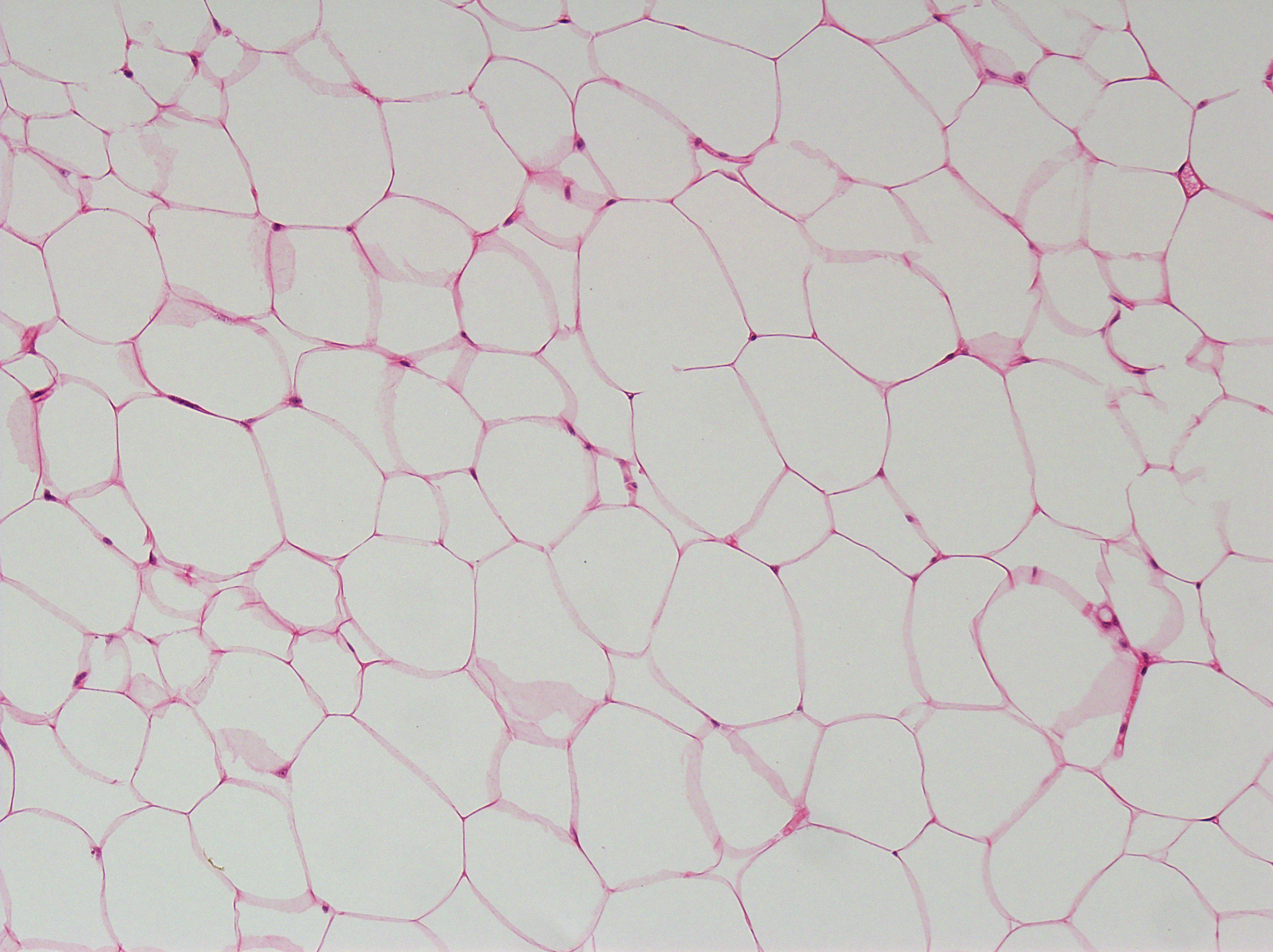

Supplement: Supplementary file 8 — Source data Fig. 6 [file 44318_2025_508_MOESM8_ESM.zip › Source data Fig.6/Figure 6G/eWAT-LysMCre.tif]

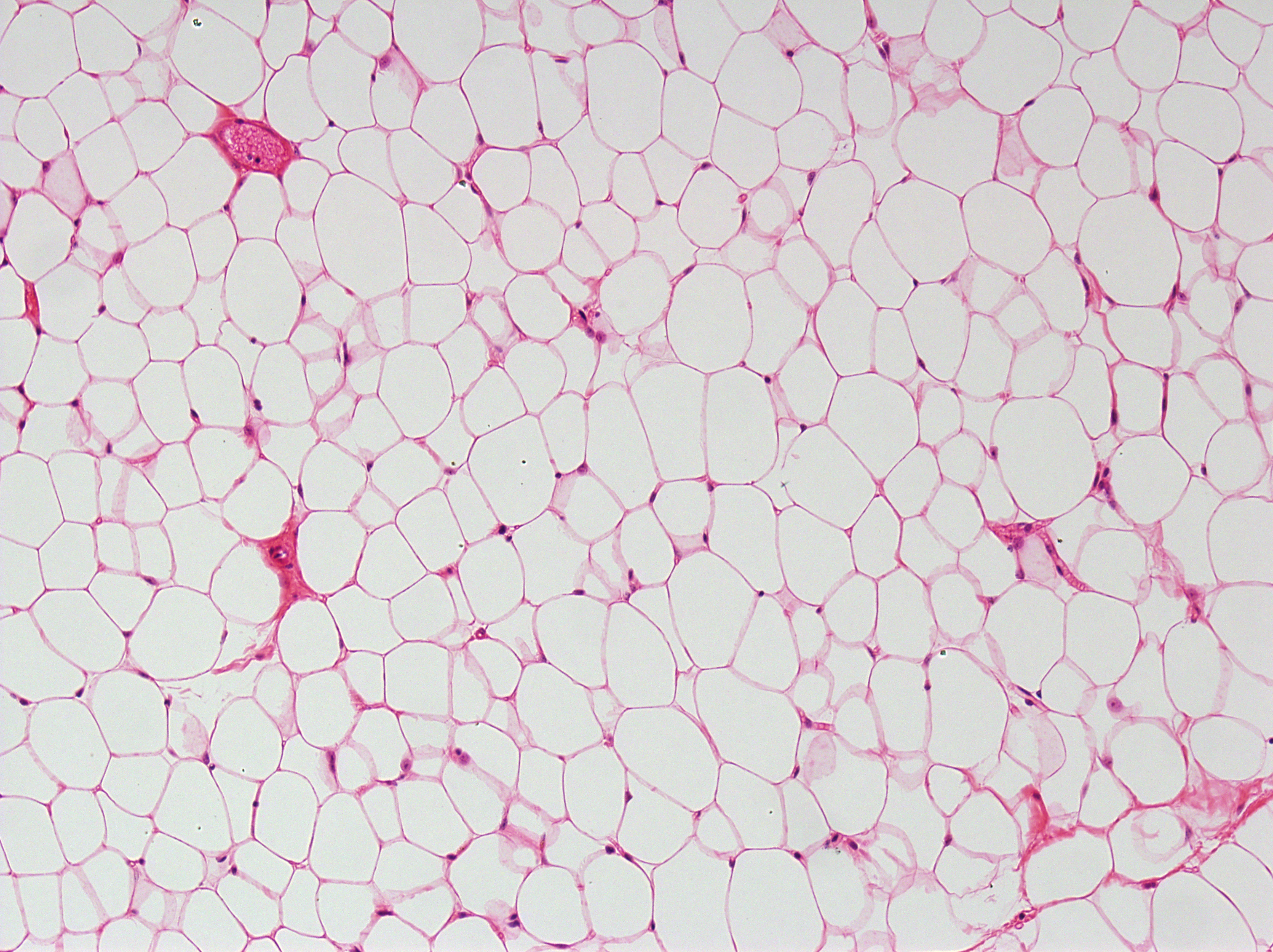

Supplement: Supplementary file 8 — Source data Fig. 6 [file 44318_2025_508_MOESM8_ESM.zip › Source data Fig.6/Figure 6G/iWAT-Dnmt1-3aFloxLysMCre.tif]

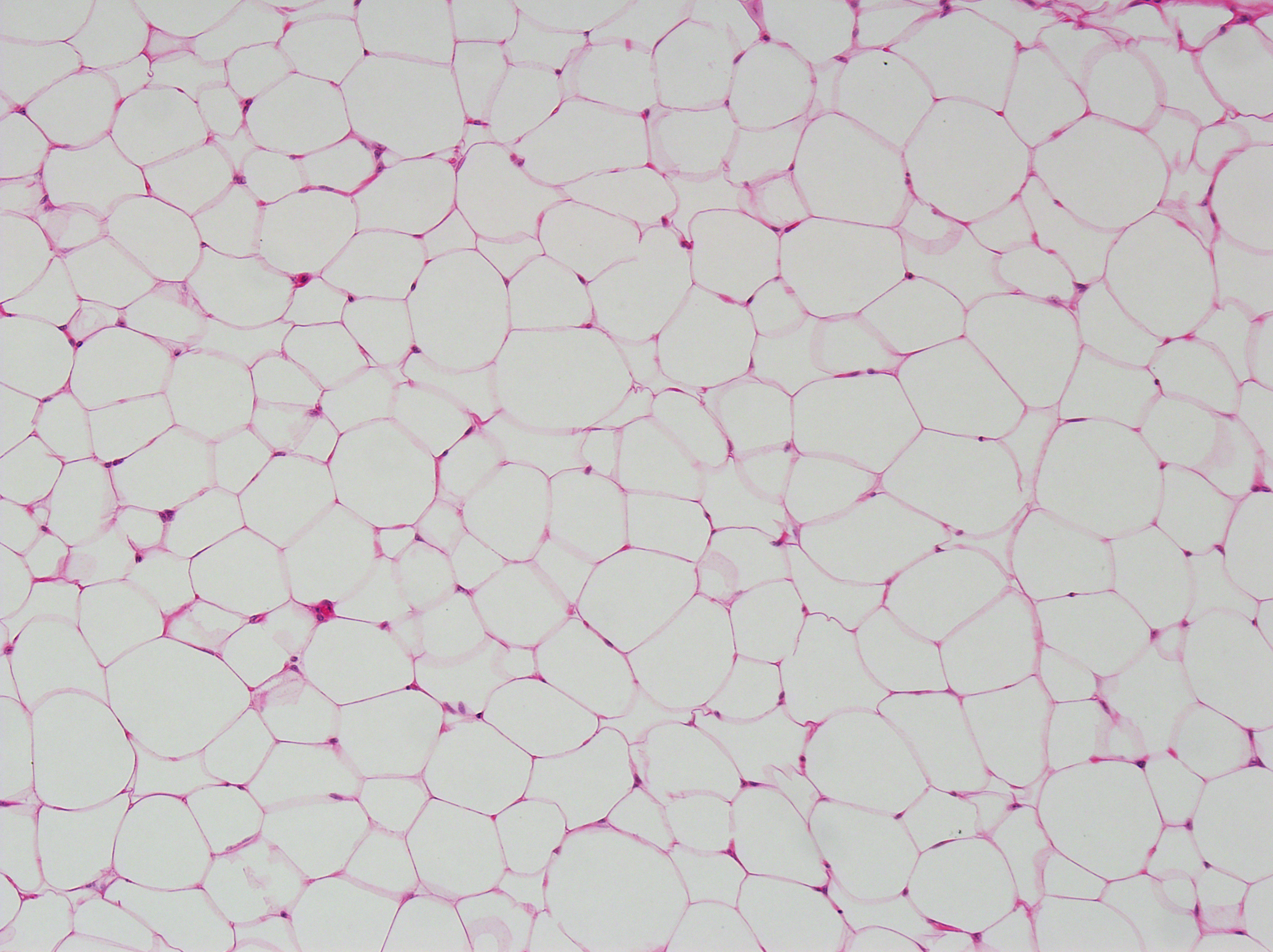

Supplement: Supplementary file 8 — Source data Fig. 6 [file 44318_2025_508_MOESM8_ESM.zip › Source data Fig.6/Figure 6G/iWAT-EP3FloxDnmt1-3aFloxLysMCre.tif]

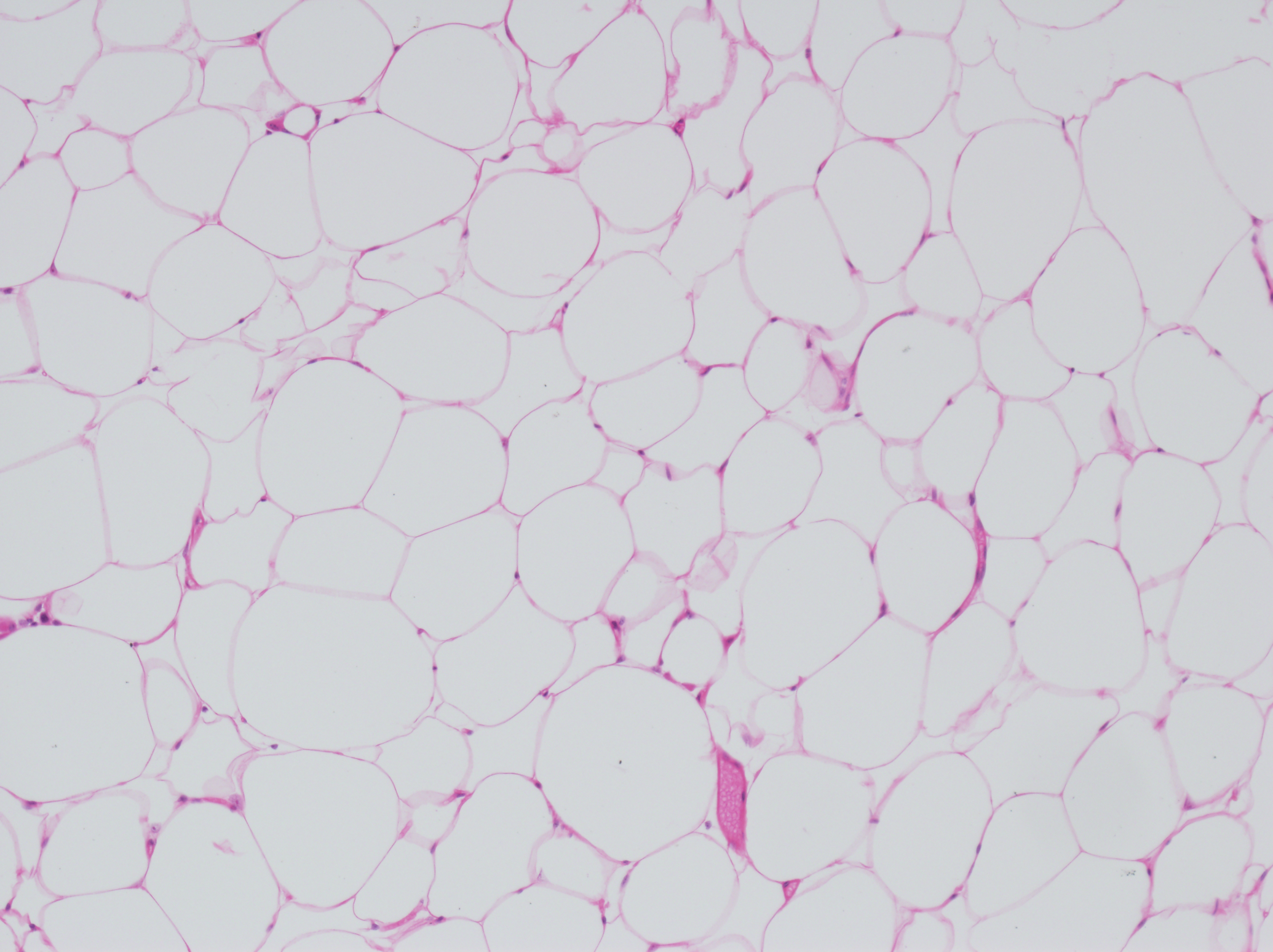

Supplement: Supplementary file 8 — Source data Fig. 6 [file 44318_2025_508_MOESM8_ESM.zip › Source data Fig.6/Figure 6G/iWAT-EP3FloxLysMCre.tif]

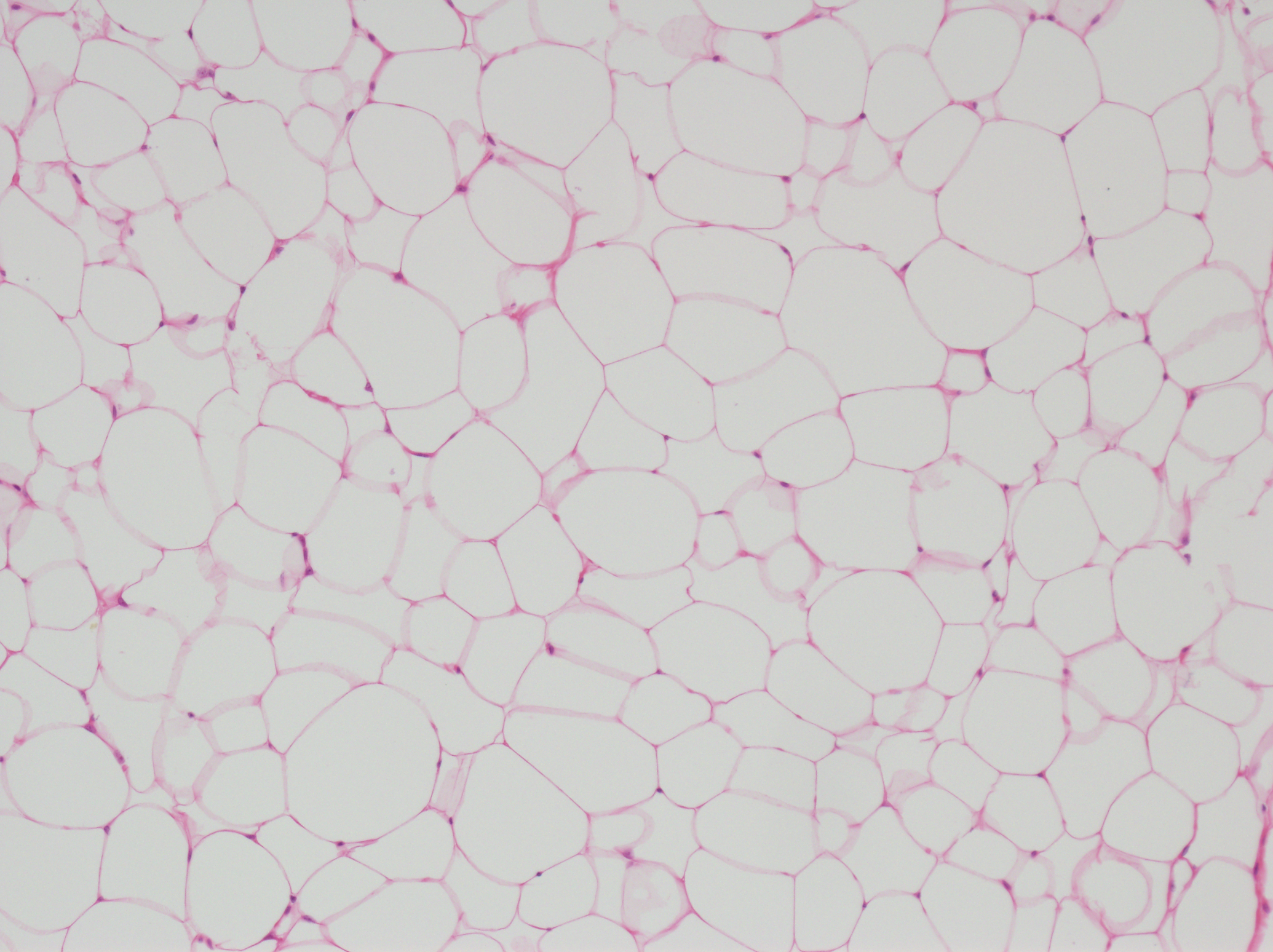

Supplement: Supplementary file 8 — Source data Fig. 6 [file 44318_2025_508_MOESM8_ESM.zip › Source data Fig.6/Figure 6G/iWAT-LysMCre.tif]

Figure 7G

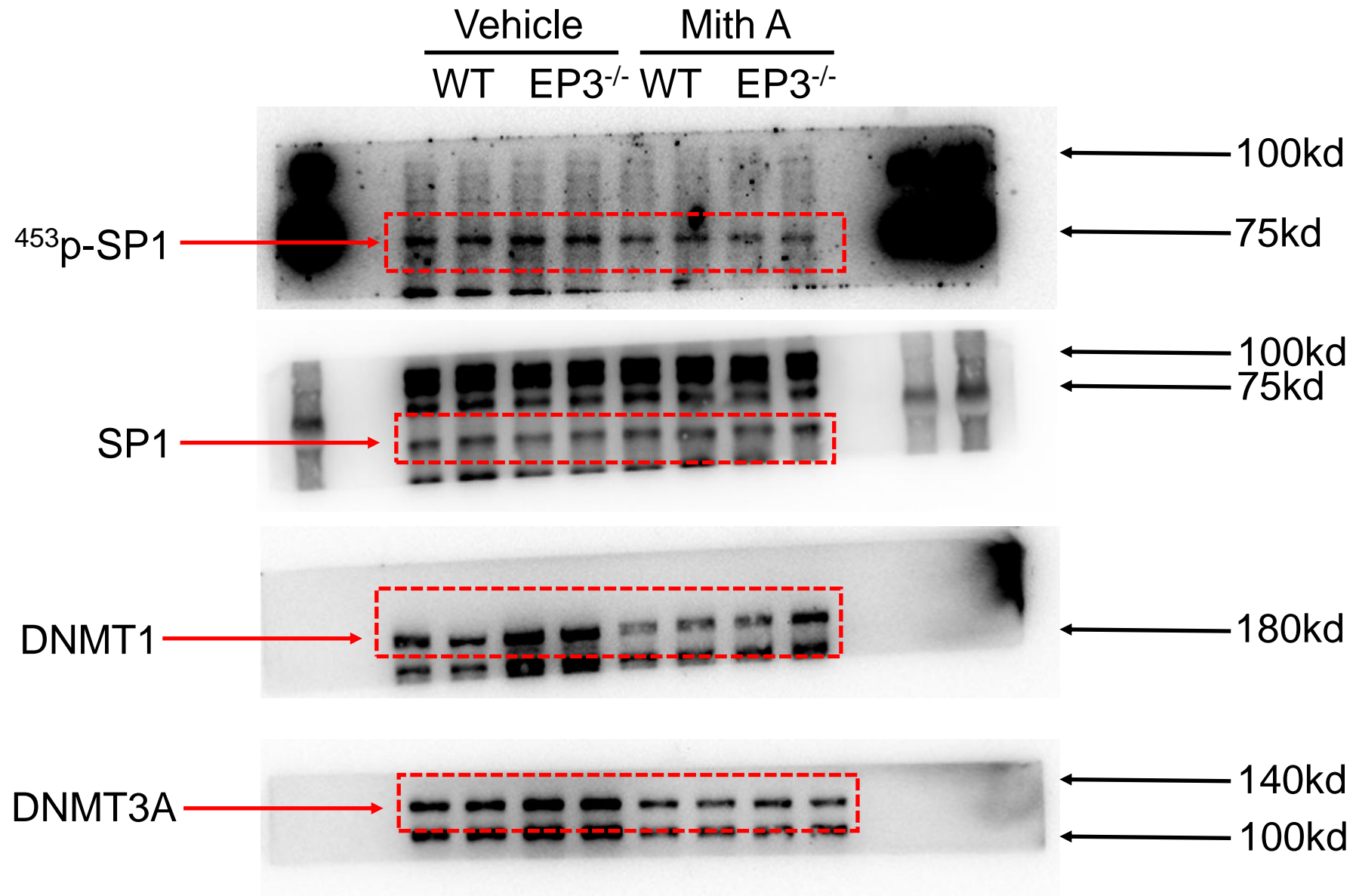

Figure 7G

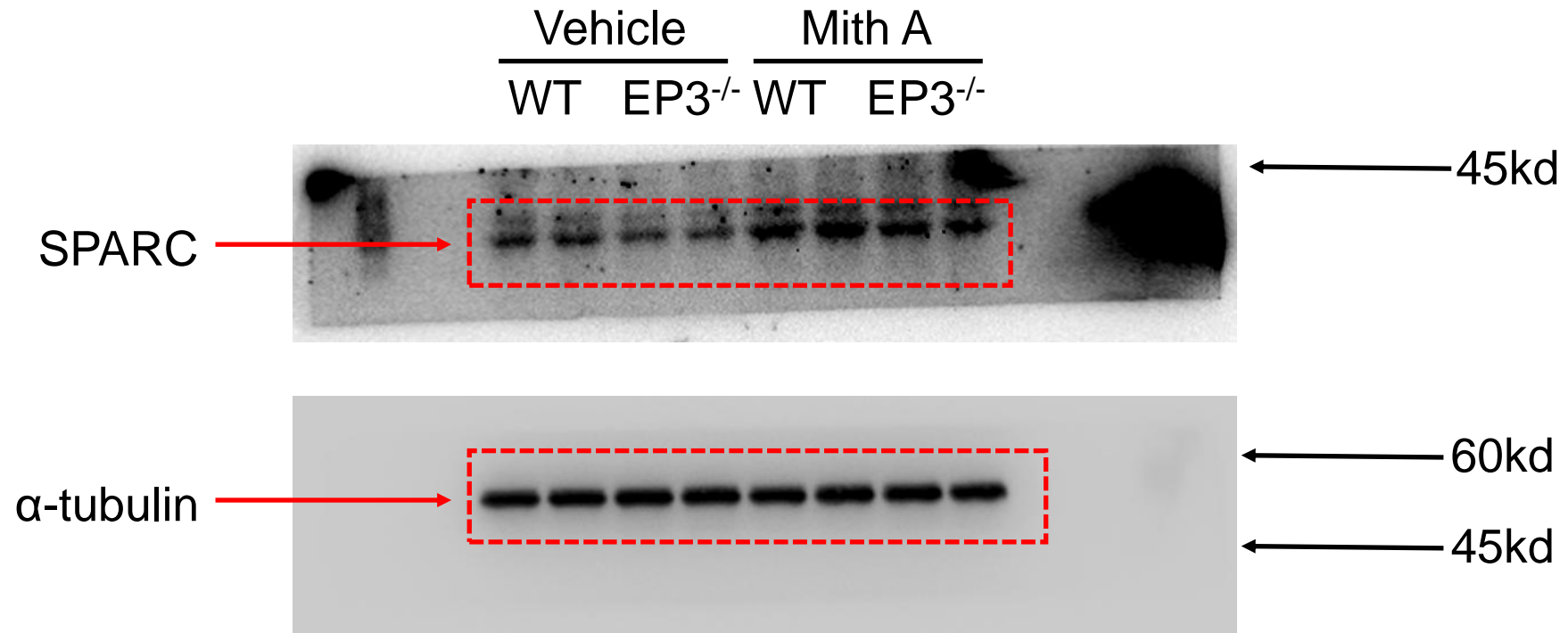

Supplement: Supplementary file 9 — Source data Fig. 7 [file 44318_2025_508_MOESM9_ESM.zip › Source data Fig.7/Figure 7G/Figure 7G.pdf]

Figure 7H

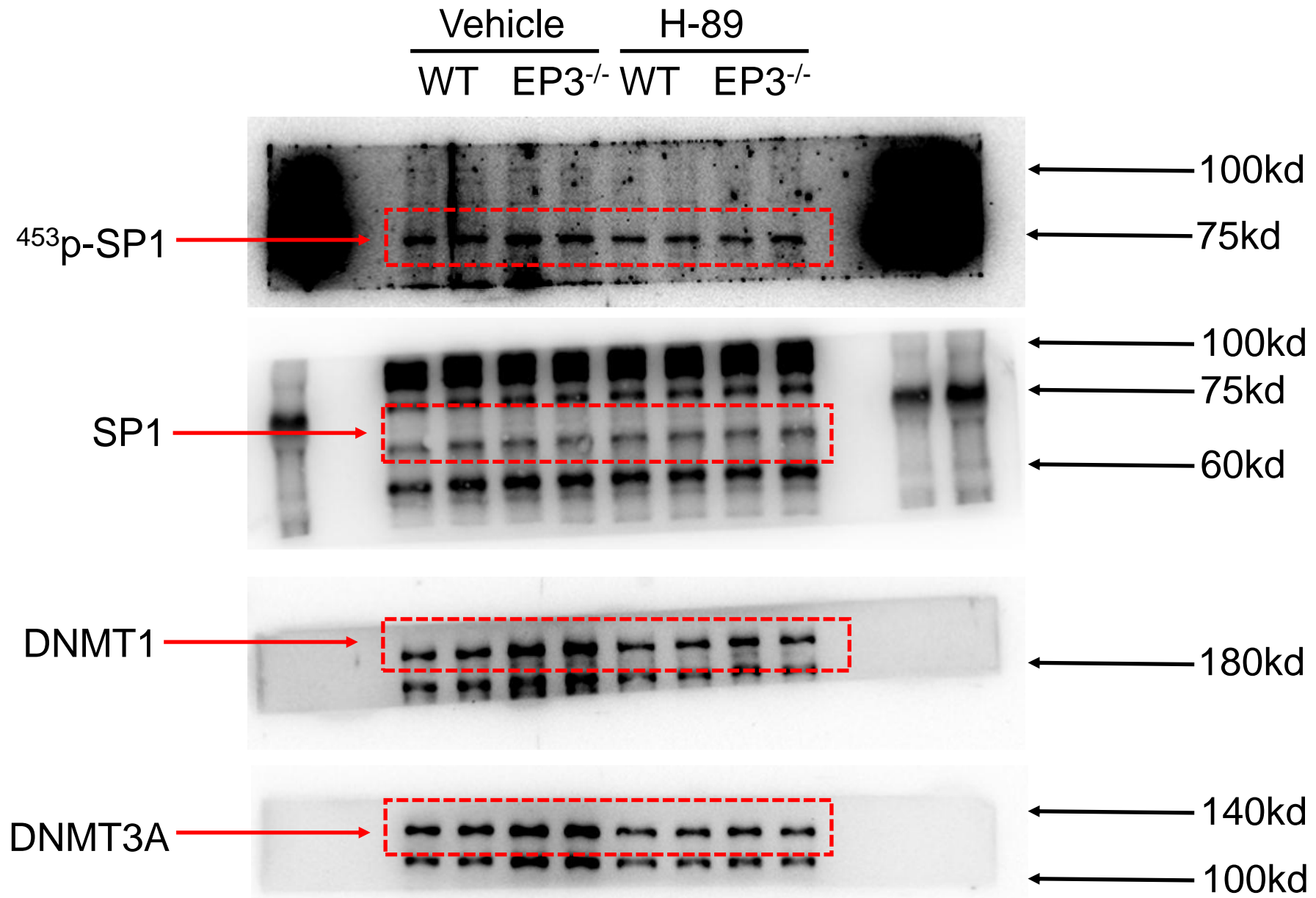

Figure 7H

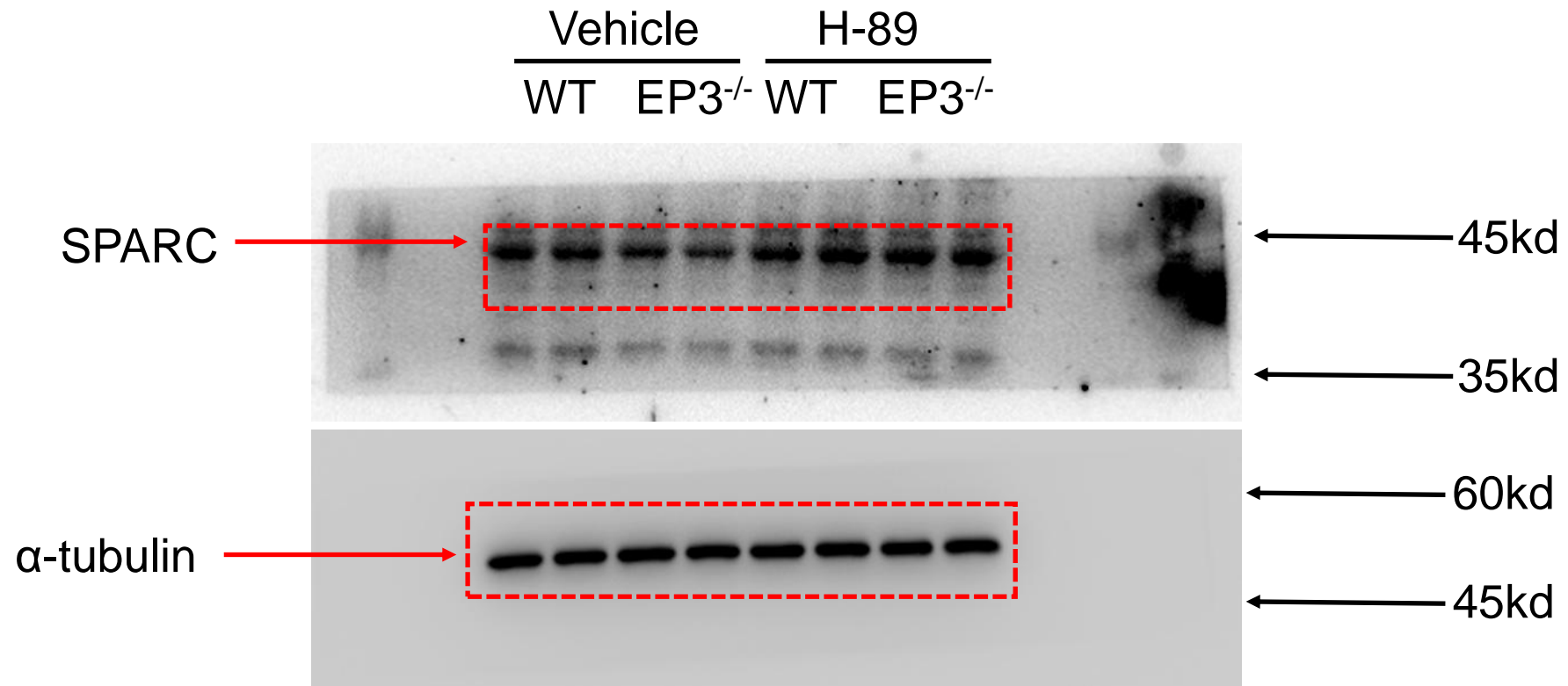

Supplement: Supplementary file 9 — Source data Fig. 7 [file 44318_2025_508_MOESM9_ESM.zip › Source data Fig.7/Figure 7H/Figure 7H.pdf]

Figure 7I

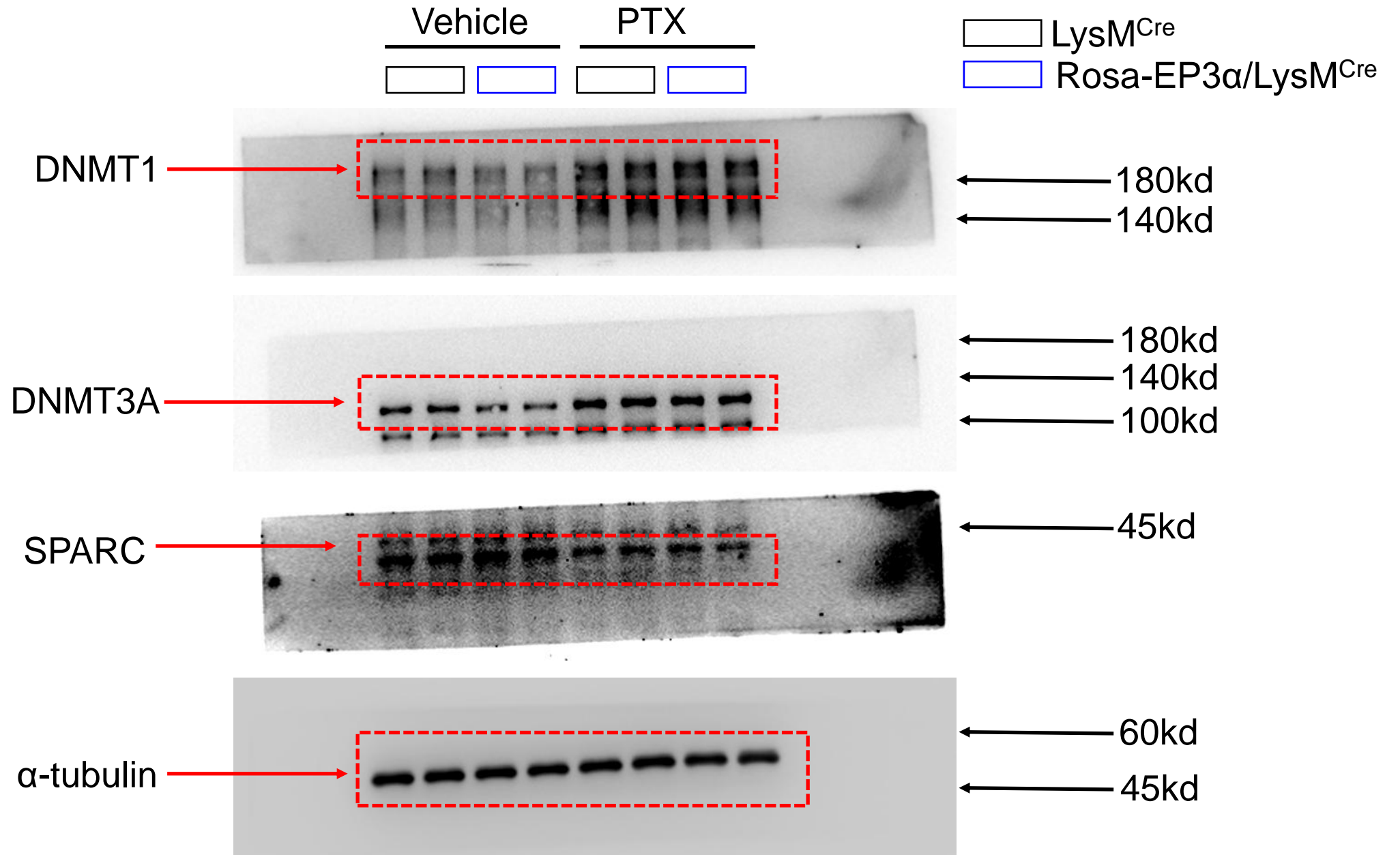

Supplement: Supplementary file 9 — Source data Fig. 7 [file 44318_2025_508_MOESM9_ESM.zip › Source data Fig.7/Figure 7I/Figure 7I.pdf]

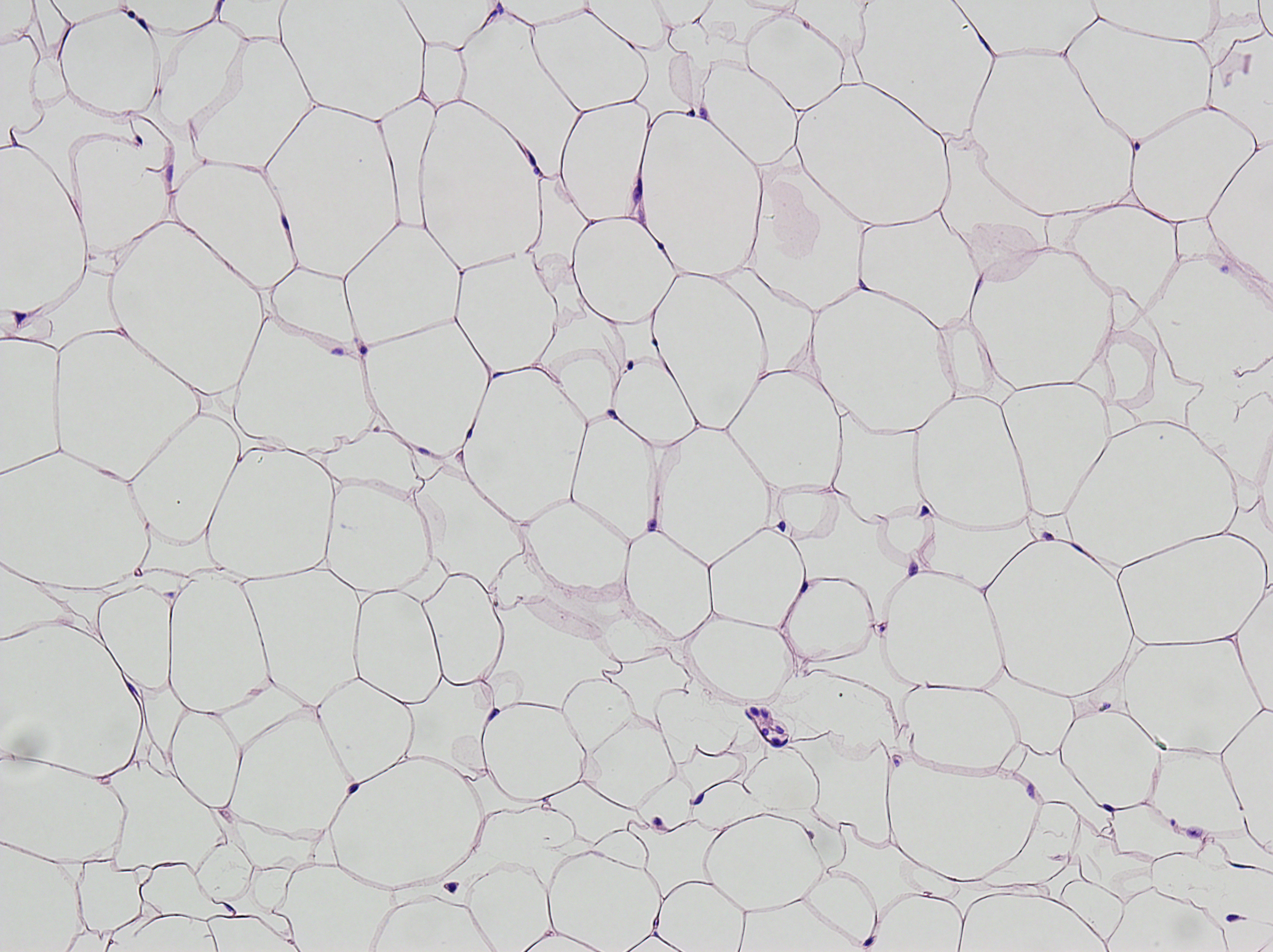

Supplement: Supplementary file 10 — Source data Fig. 8 [file 44318_2025_508_MOESM10_ESM.zip › Source data Fig.8/Figure 8H/eWAT-HFD-Sulprostone.tif]

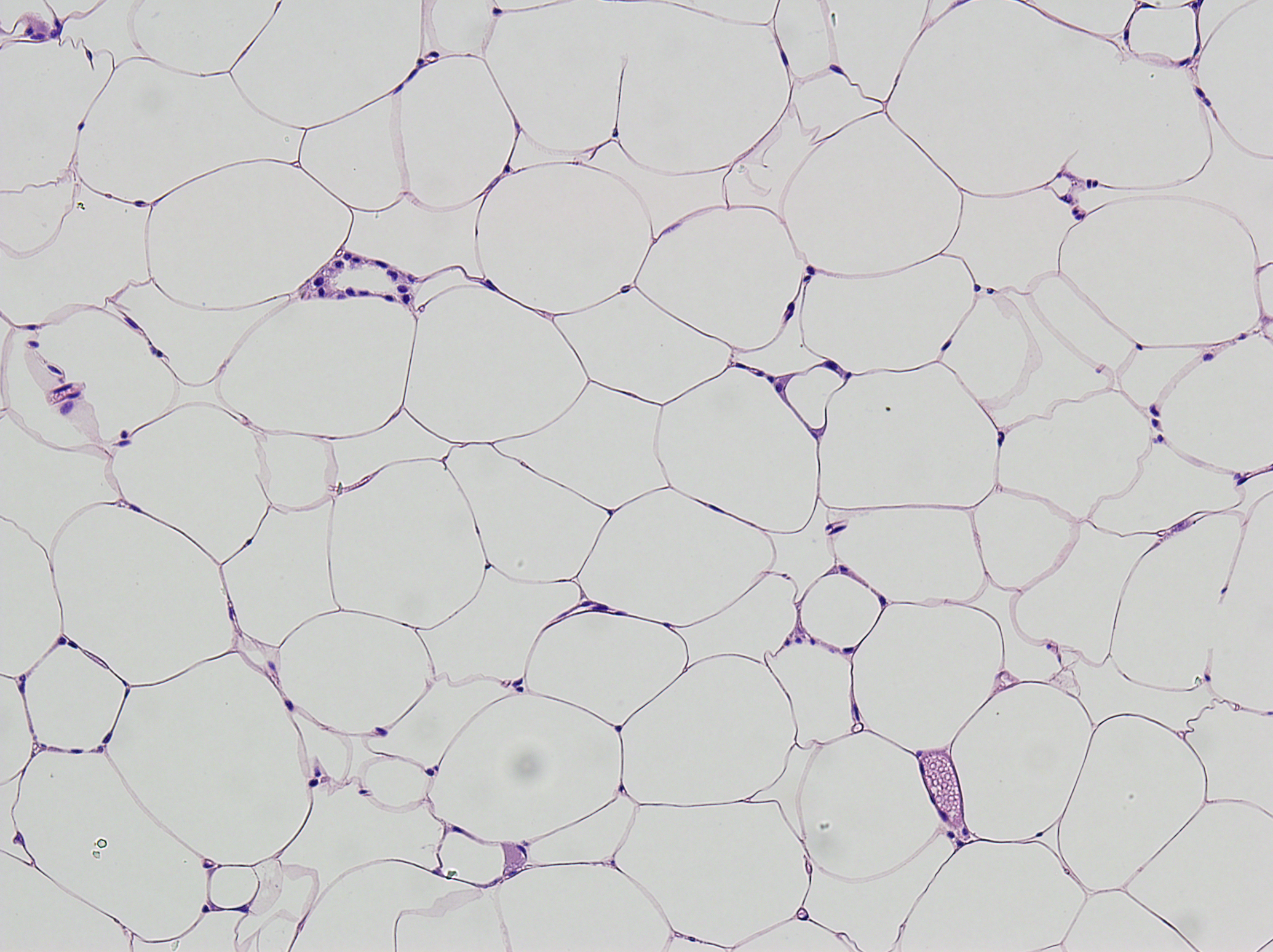

Supplement: Supplementary file 10 — Source data Fig. 8 [file 44318_2025_508_MOESM10_ESM.zip › Source data Fig.8/Figure 8H/eWAT-HFD-Vehicle.tif]

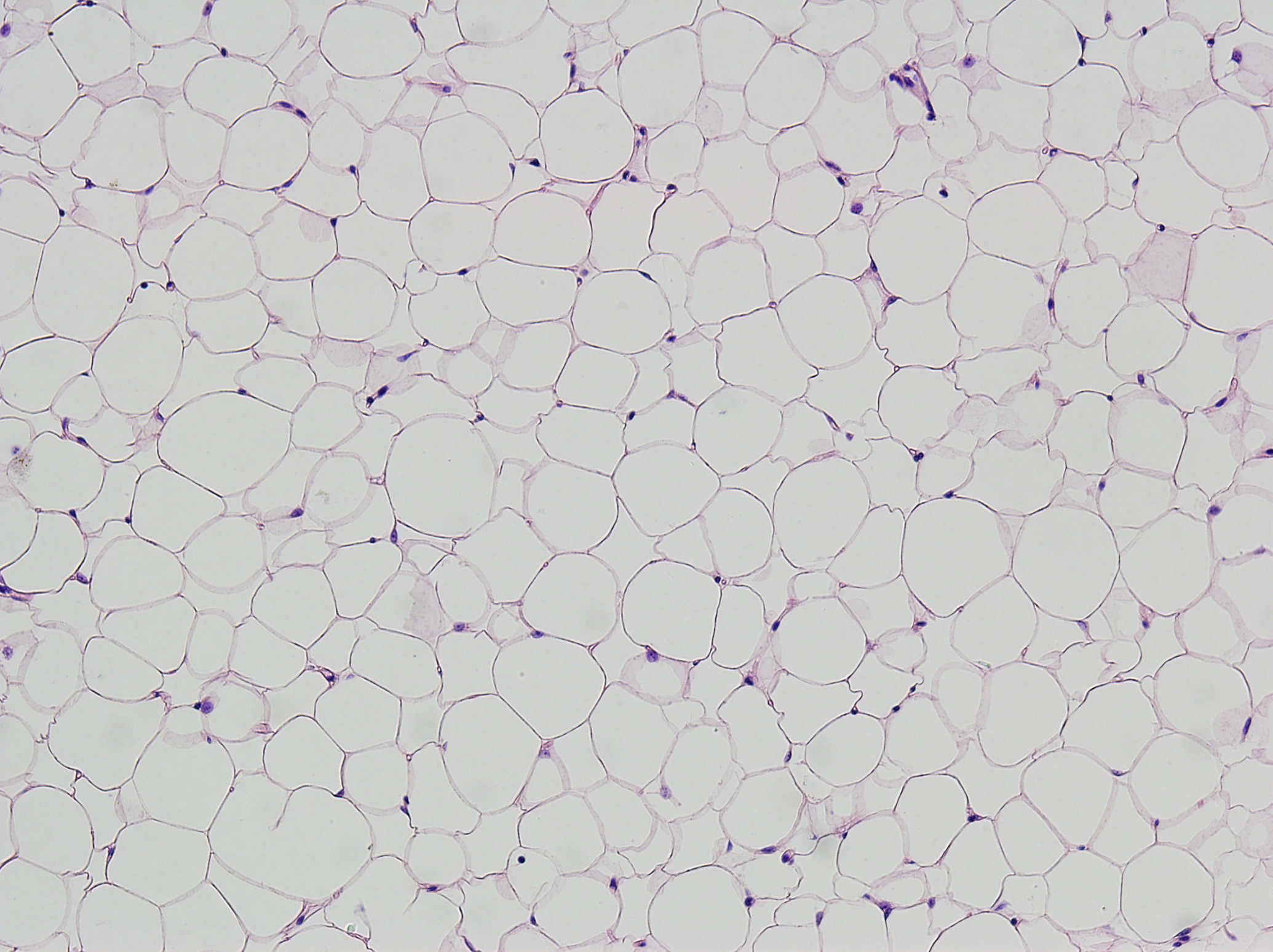

Supplement: Supplementary file 10 — Source data Fig. 8 [file 44318_2025_508_MOESM10_ESM.zip › Source data Fig.8/Figure 8H/eWAT-NCD-Vehicle.tif]

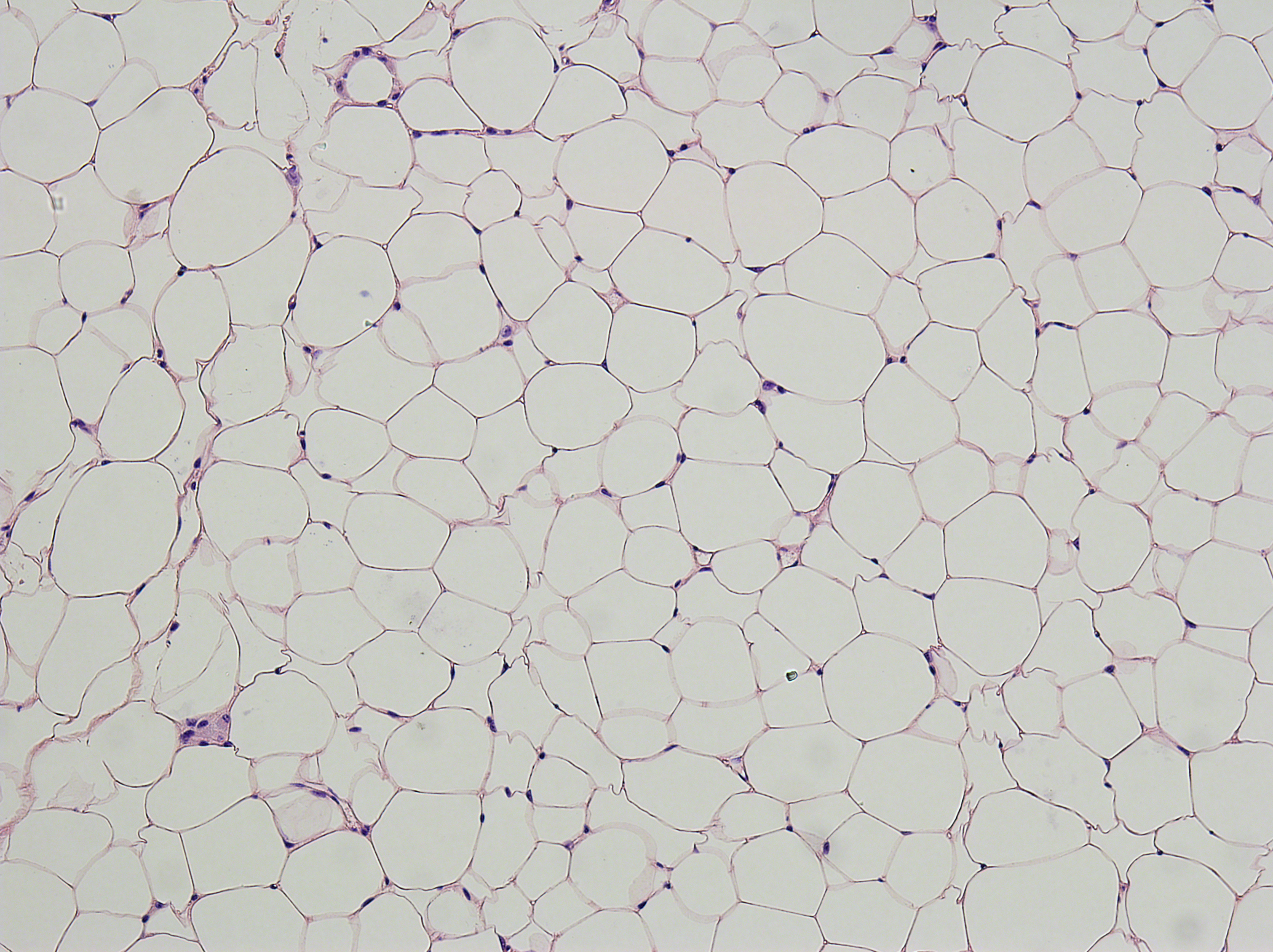

Supplement: Supplementary file 10 — Source data Fig. 8 [file 44318_2025_508_MOESM10_ESM.zip › Source data Fig.8/Figure 8H/iWAT-HFD-Sulprostone.tif]

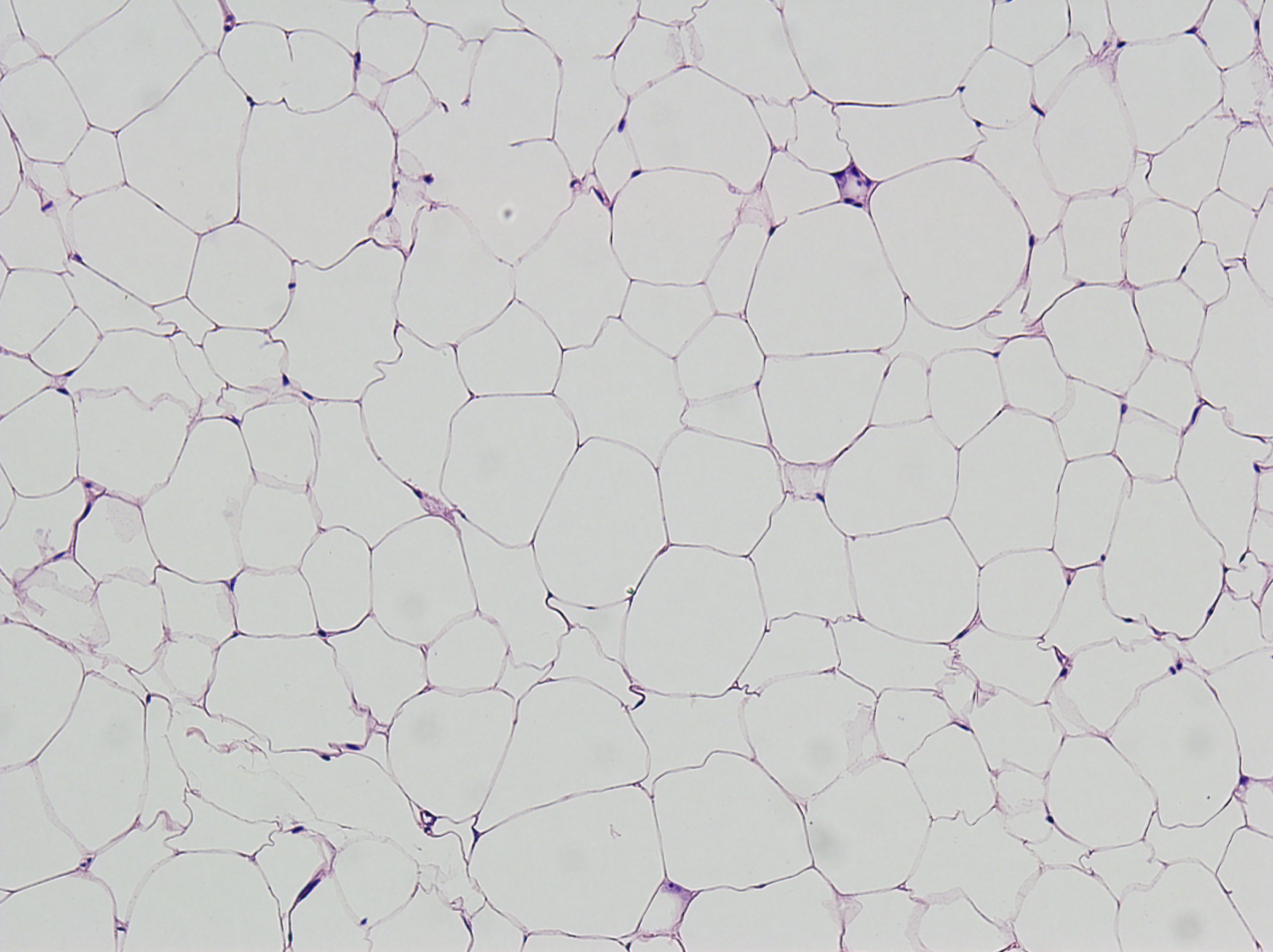

Supplement: Supplementary file 10 — Source data Fig. 8 [file 44318_2025_508_MOESM10_ESM.zip › Source data Fig.8/Figure 8H/iWAT-HFD-Vehicle.tif]

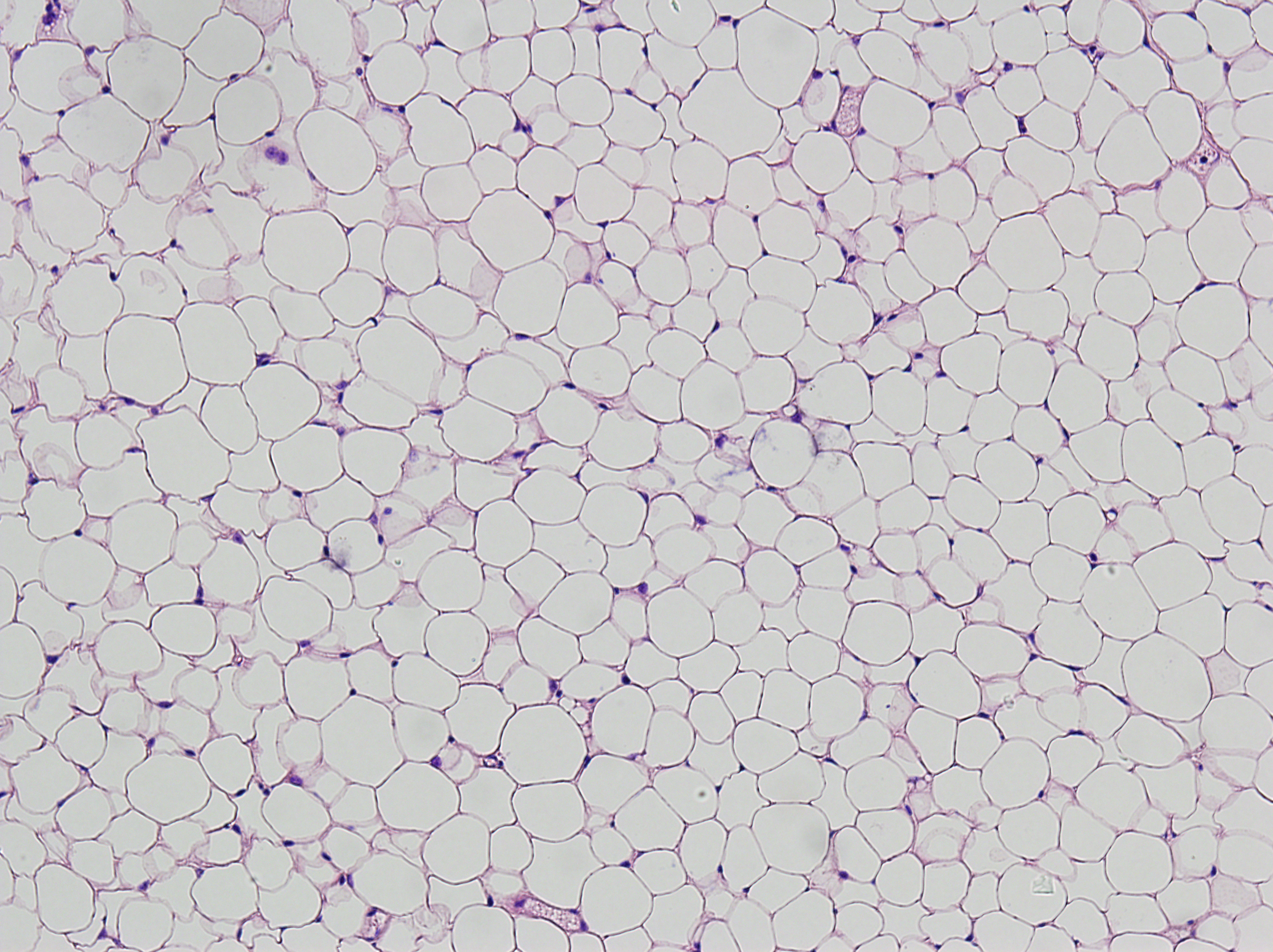

Supplement: Supplementary file 10 — Source data Fig. 8 [file 44318_2025_508_MOESM10_ESM.zip › Source data Fig.8/Figure 8H/iWAT-NCD-Vehicle.tif]

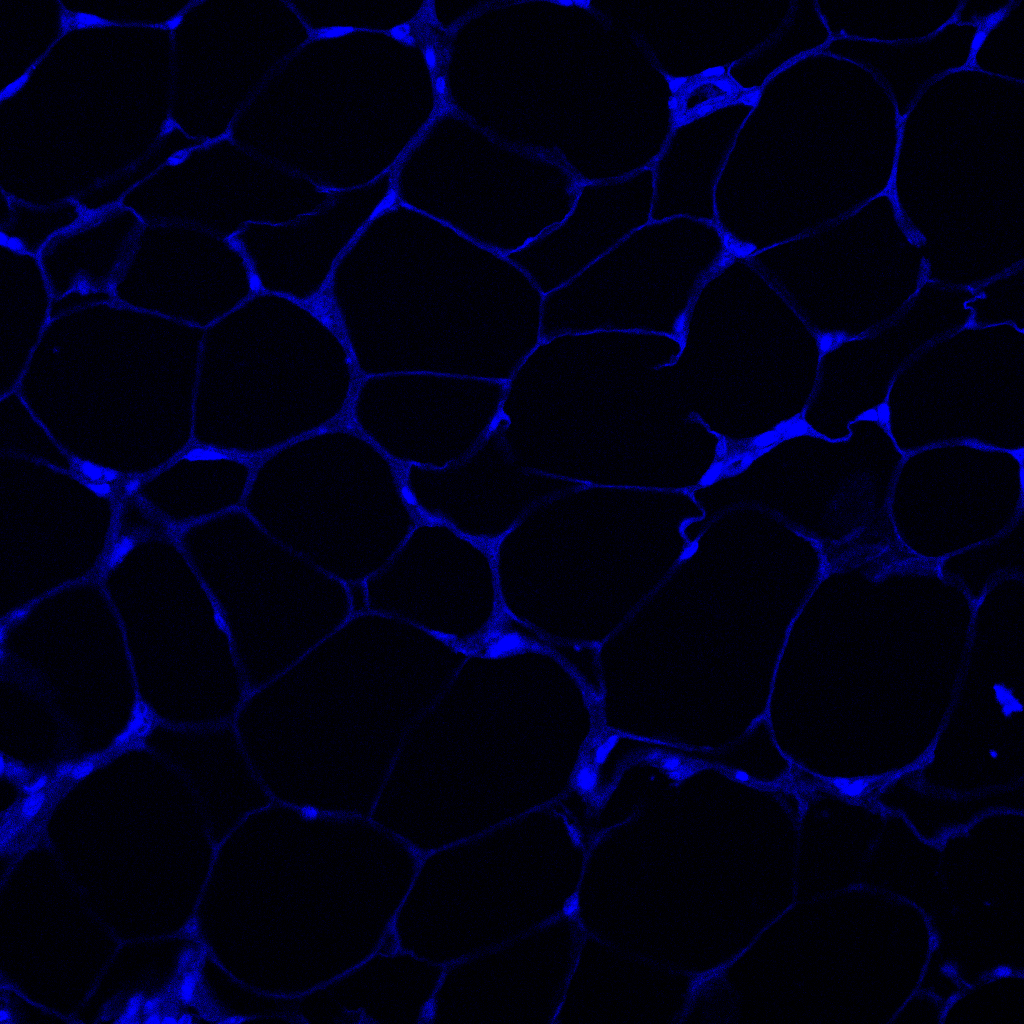

Supplement: Supplementary file 10 — Source data Fig. 8 [file 44318_2025_508_MOESM10_ESM.zip › Source data Fig.8/Figure 8M/HFD-Sulprostone/HFD-Sulprostone-DAPI.tif]

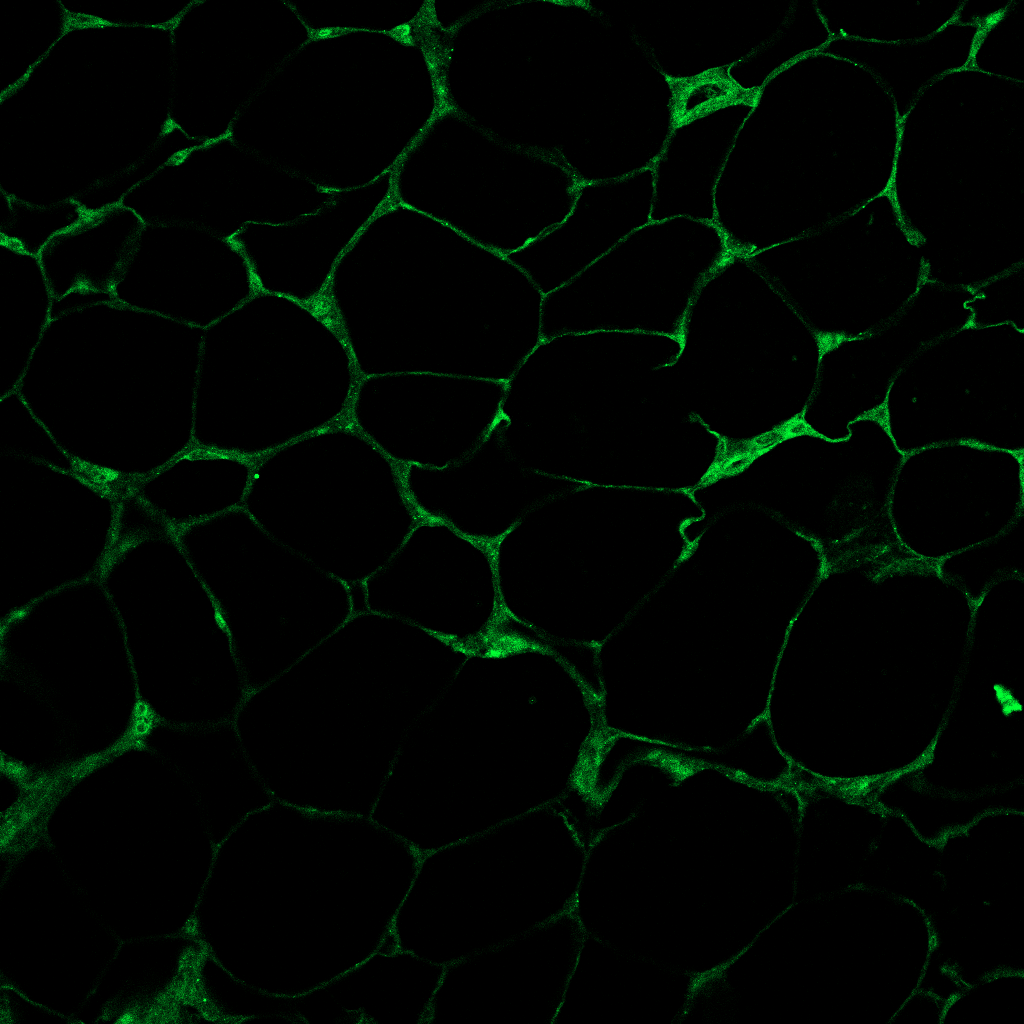

Supplement: Supplementary file 10 — Source data Fig. 8 [file 44318_2025_508_MOESM10_ESM.zip › Source data Fig.8/Figure 8M/HFD-Sulprostone/HFD-Sulprostone-DNMT1.tif]

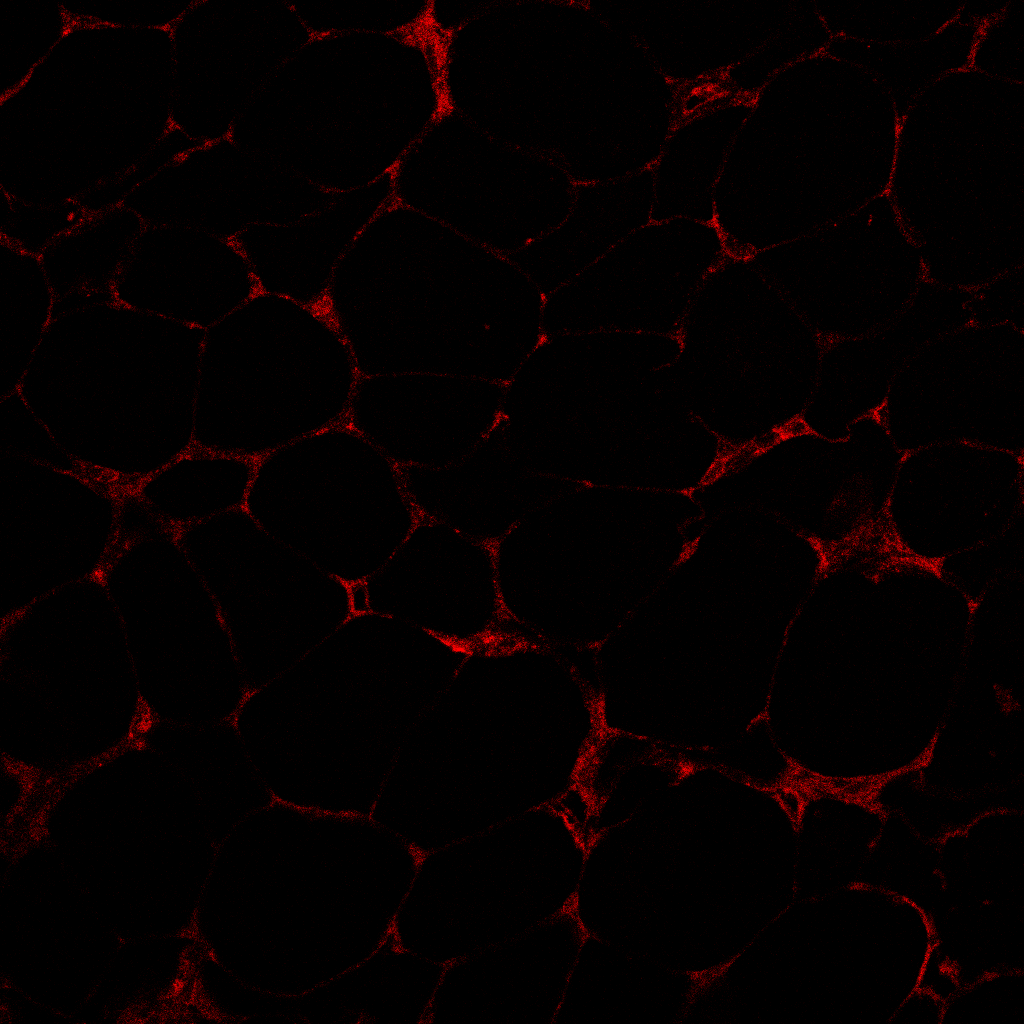

Supplement: Supplementary file 10 — Source data Fig. 8 [file 44318_2025_508_MOESM10_ESM.zip › Source data Fig.8/Figure 8M/HFD-Sulprostone/HFD-Sulprostone-MAC-3.tif]

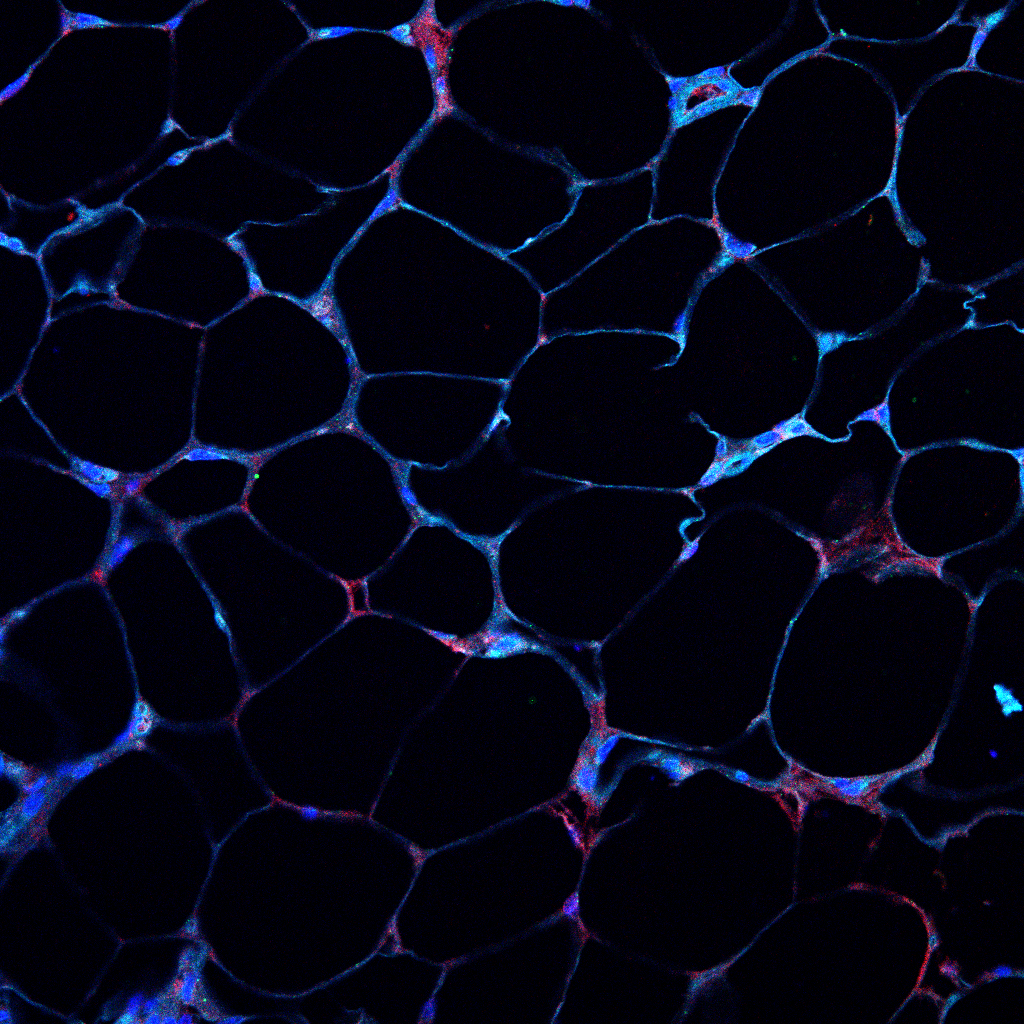

Supplement: Supplementary file 10 — Source data Fig. 8 [file 44318_2025_508_MOESM10_ESM.zip › Source data Fig.8/Figure 8M/HFD-Sulprostone/HFD-Sulprostone-Merge.tif]

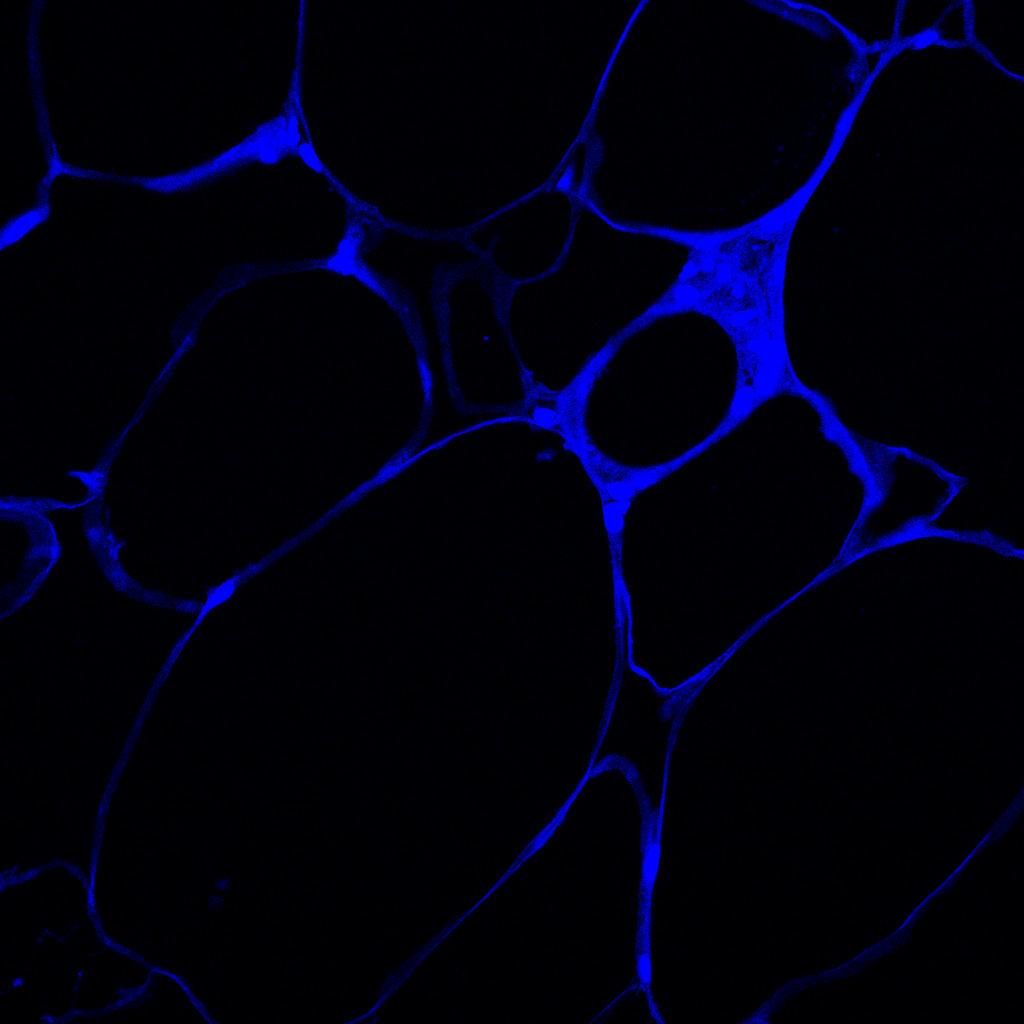

Supplement: Supplementary file 10 — Source data Fig. 8 [file 44318_2025_508_MOESM10_ESM.zip › Source data Fig.8/Figure 8M/HFD-Vehicle/HFD-Vehicle-DAPI.tif]

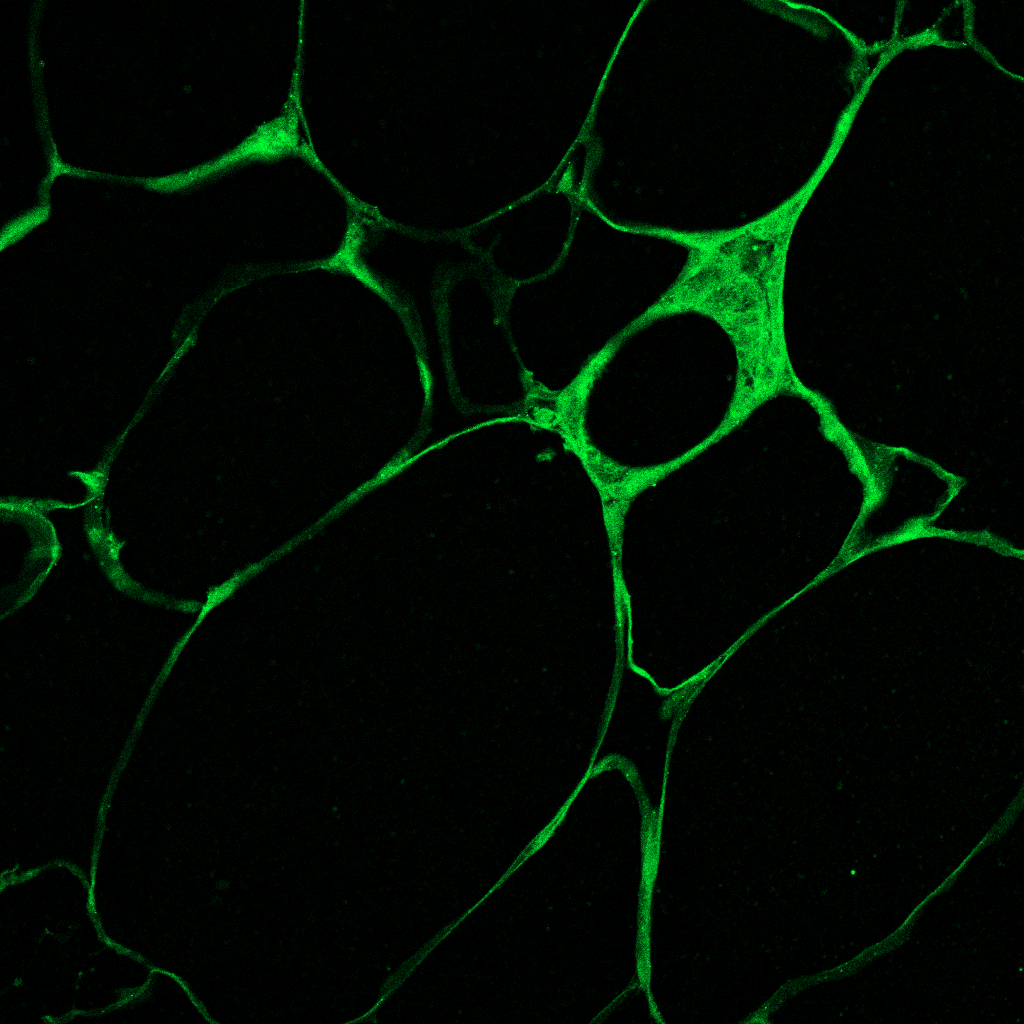

Supplement: Supplementary file 10 — Source data Fig. 8 [file 44318_2025_508_MOESM10_ESM.zip › Source data Fig.8/Figure 8M/HFD-Vehicle/HFD-Vehicle-DNMT1.tif]

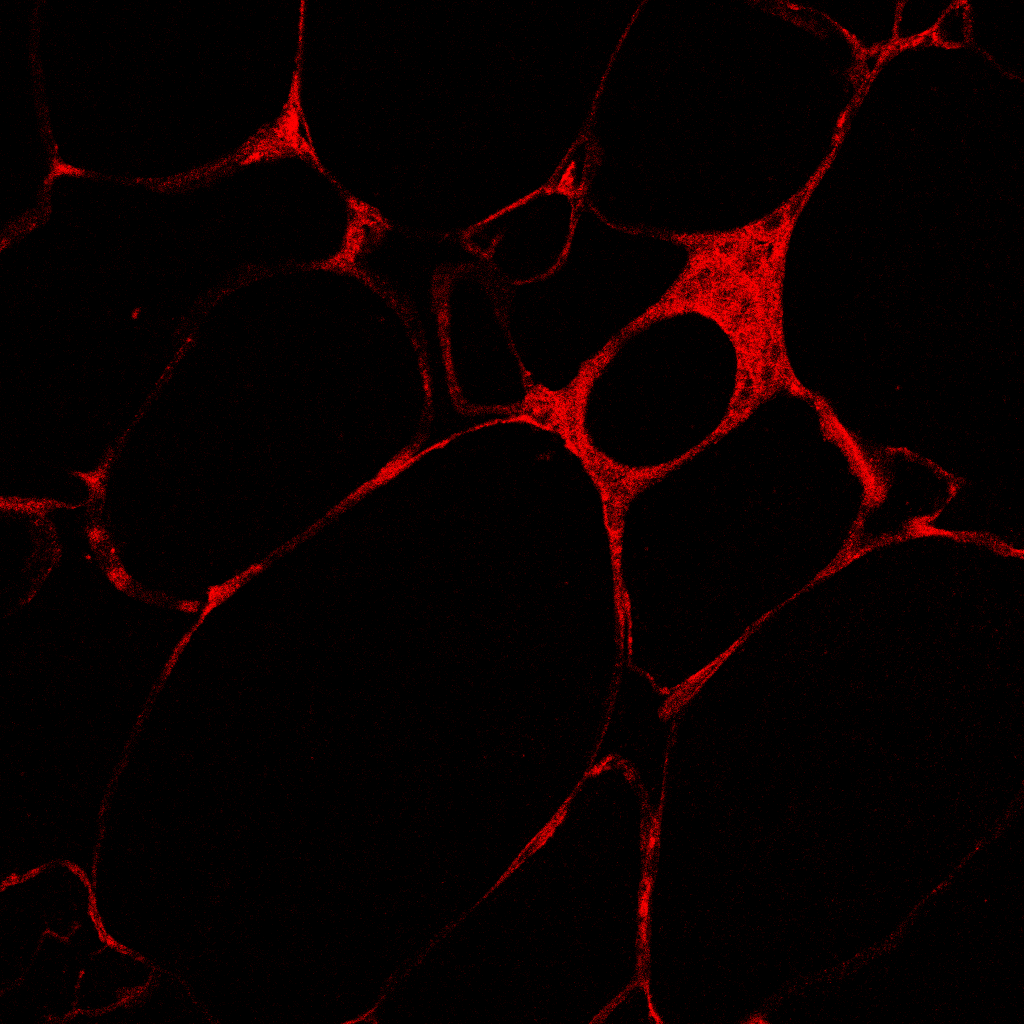

Supplement: Supplementary file 10 — Source data Fig. 8 [file 44318_2025_508_MOESM10_ESM.zip › Source data Fig.8/Figure 8M/HFD-Vehicle/HFD-Vehicle-MAC-3.tif]

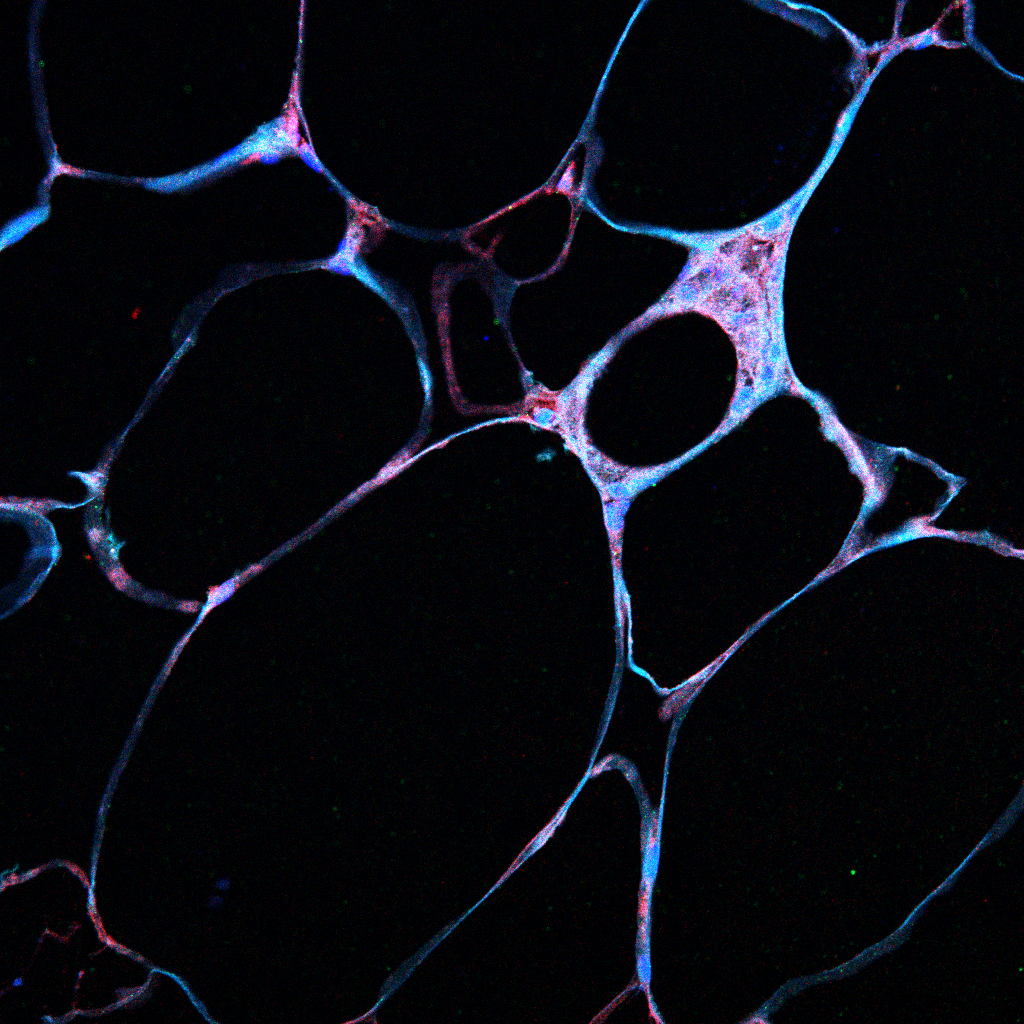

Supplement: Supplementary file 10 — Source data Fig. 8 [file 44318_2025_508_MOESM10_ESM.zip › Source data Fig.8/Figure 8M/HFD-Vehicle/HFD-Vehicle-Merge.tif]

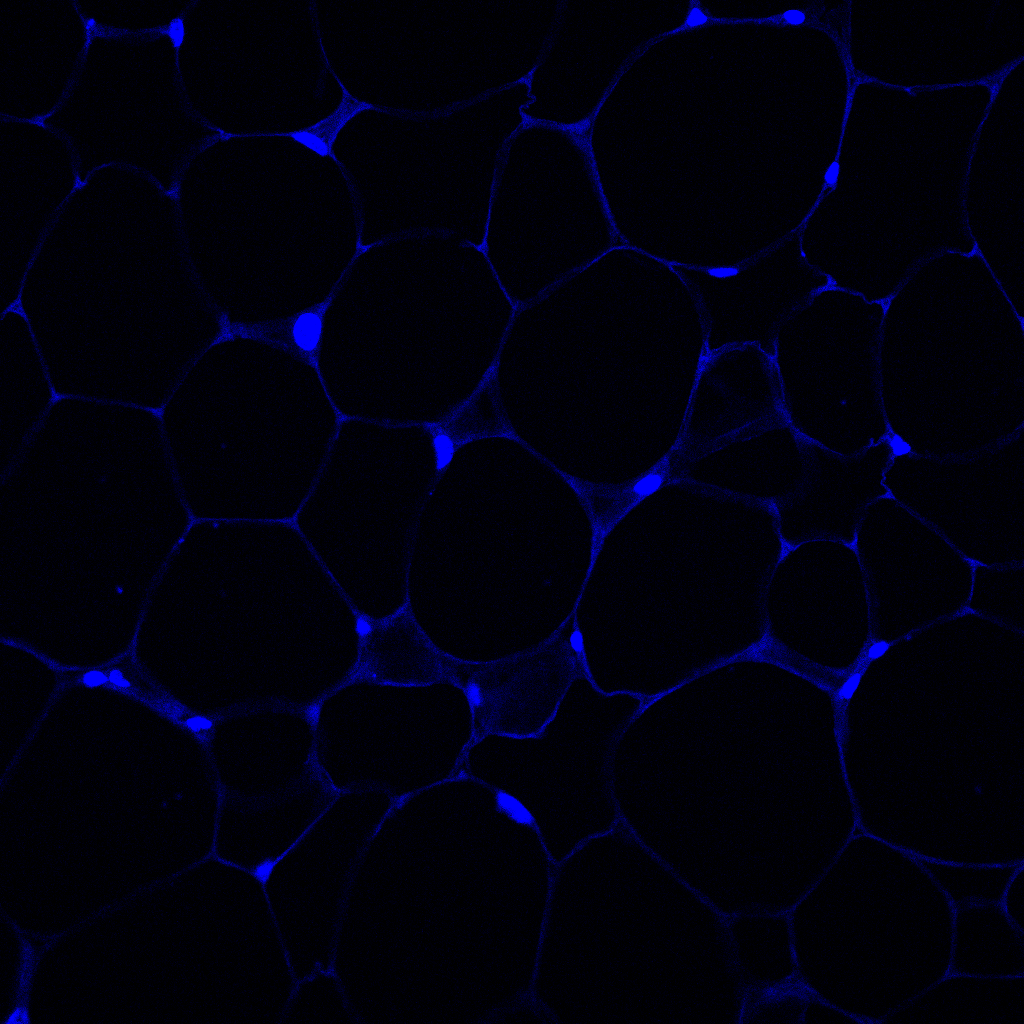

Supplement: Supplementary file 10 — Source data Fig. 8 [file 44318_2025_508_MOESM10_ESM.zip › Source data Fig.8/Figure 8M/NCD-Vehicle/NCD-Vehicle-DAPI.tif]

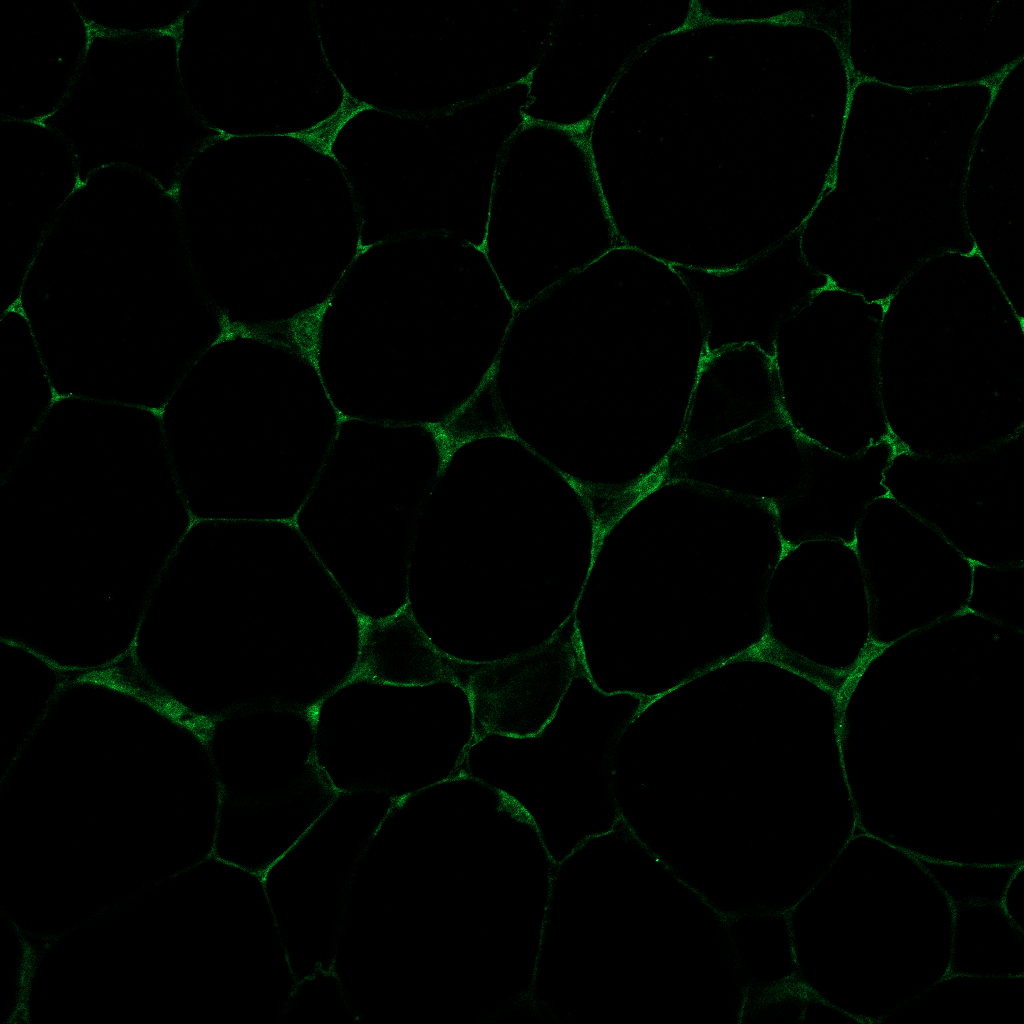

Supplement: Supplementary file 10 — Source data Fig. 8 [file 44318_2025_508_MOESM10_ESM.zip › Source data Fig.8/Figure 8M/NCD-Vehicle/NCD-Vehicle-DNMT1.tif]

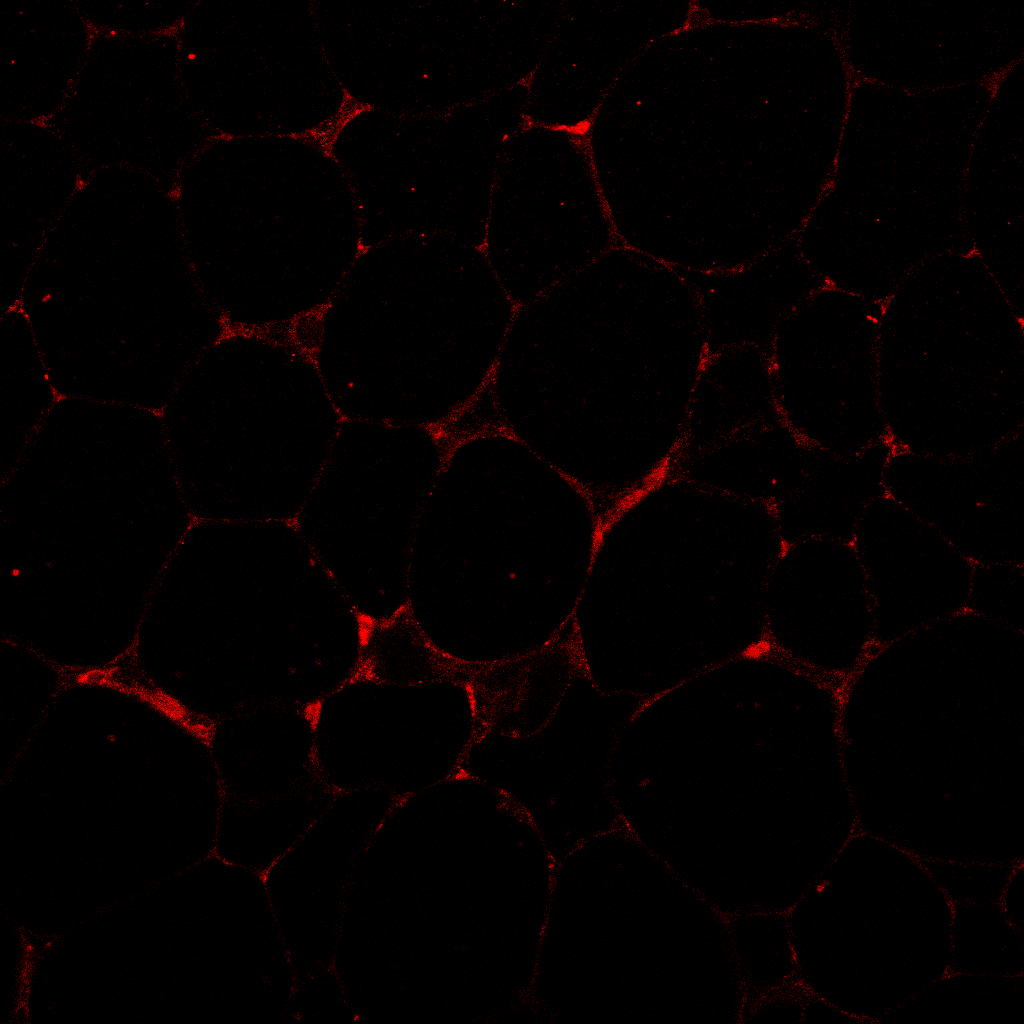

Supplement: Supplementary file 10 — Source data Fig. 8 [file 44318_2025_508_MOESM10_ESM.zip › Source data Fig.8/Figure 8M/NCD-Vehicle/NCD-Vehicle-MAC-3.tif]

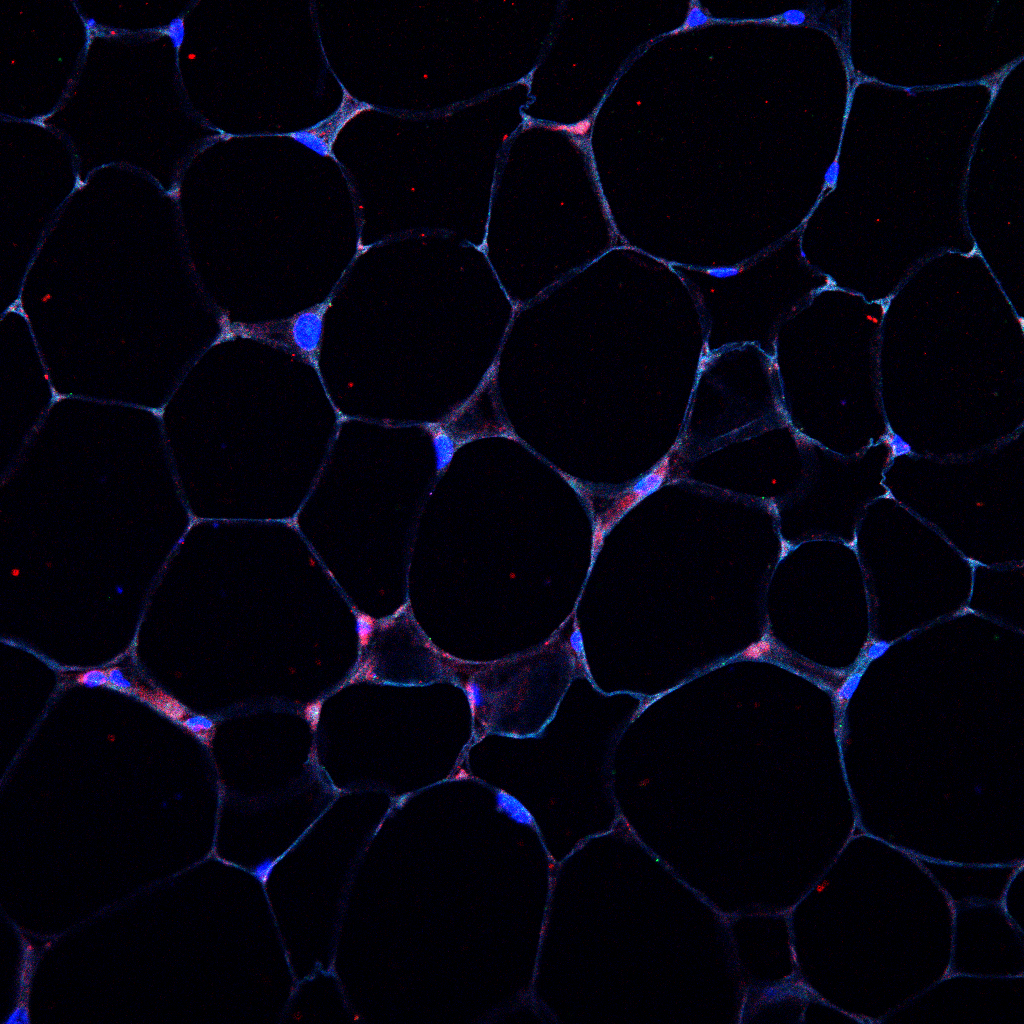

Supplement: Supplementary file 10 — Source data Fig. 8 [file 44318_2025_508_MOESM10_ESM.zip › Source data Fig.8/Figure 8M/NCD-Vehicle/NCD-Vehicle-Merge.tif]

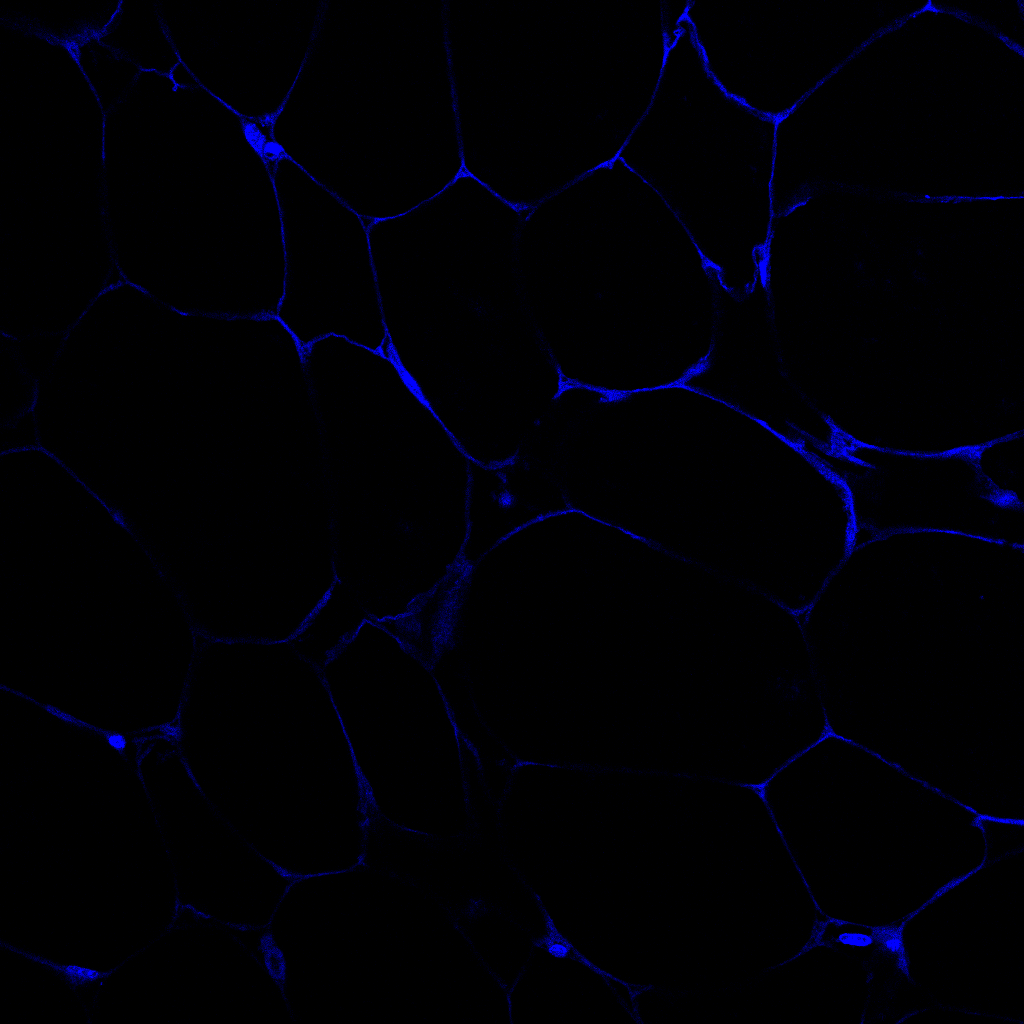

Supplement: Supplementary file 10 — Source data Fig. 8 [file 44318_2025_508_MOESM10_ESM.zip › Source data Fig.8/Figure 8O/HFD-Sulprostone/HFD-Sulprostone-DAPI.tif]

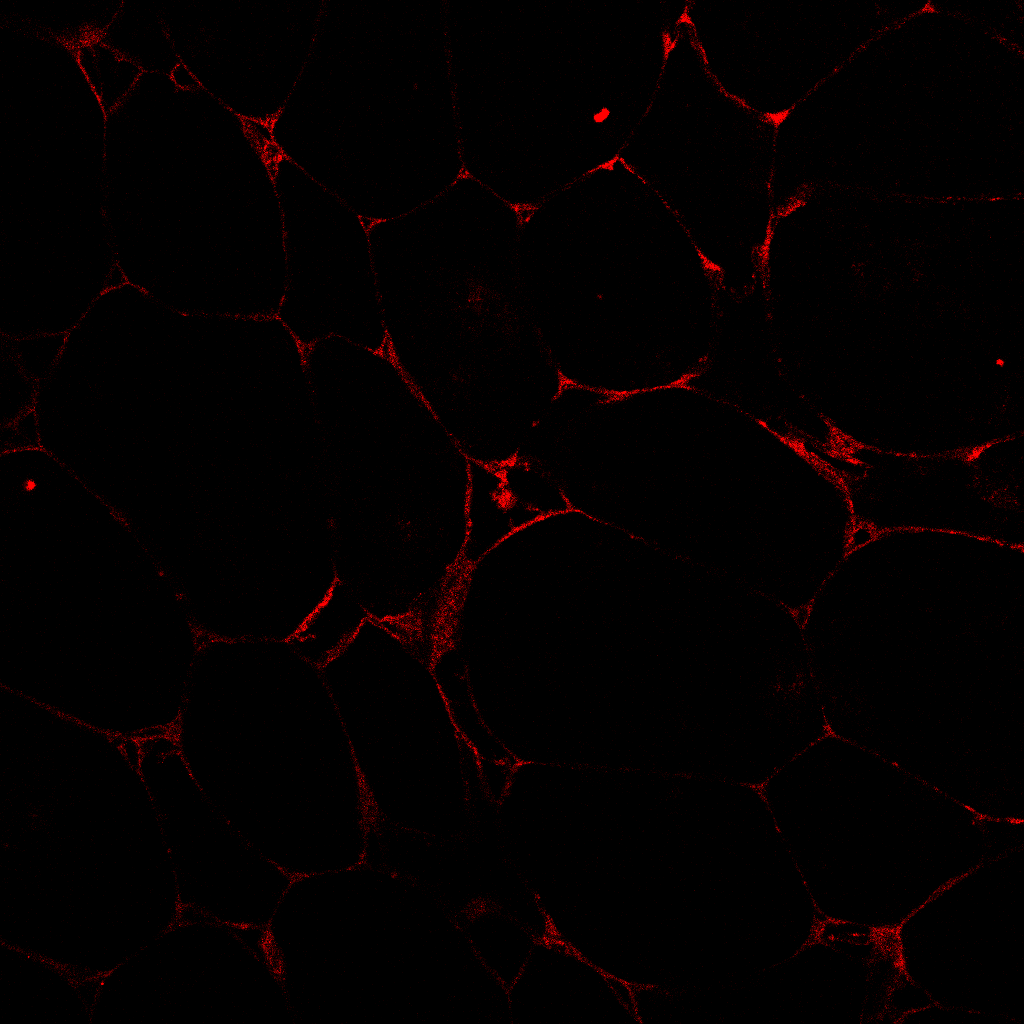

Supplement: Supplementary file 10 — Source data Fig. 8 [file 44318_2025_508_MOESM10_ESM.zip › Source data Fig.8/Figure 8O/HFD-Sulprostone/HFD-Sulprostone-MAC-3.tif]

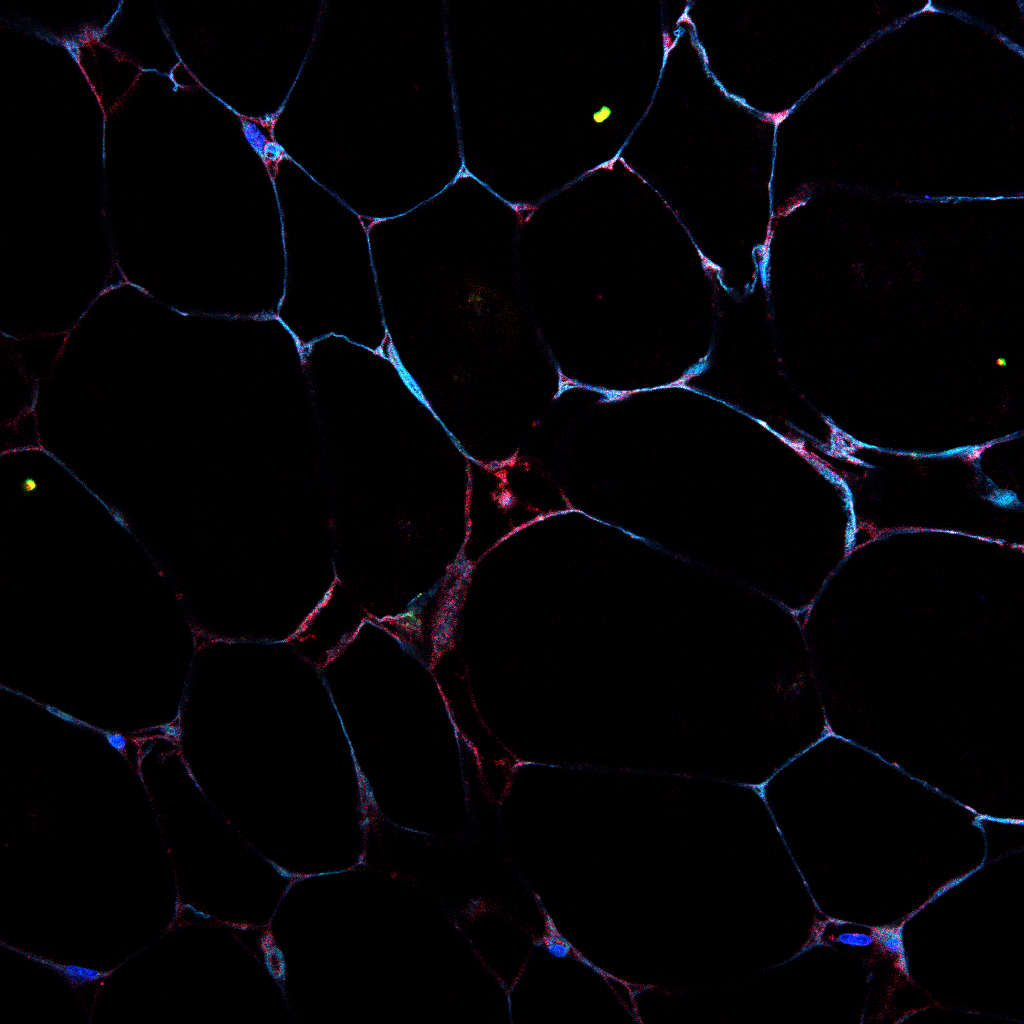

Supplement: Supplementary file 10 — Source data Fig. 8 [file 44318_2025_508_MOESM10_ESM.zip › Source data Fig.8/Figure 8O/HFD-Sulprostone/HFD-Sulprostone-Merge.tif]

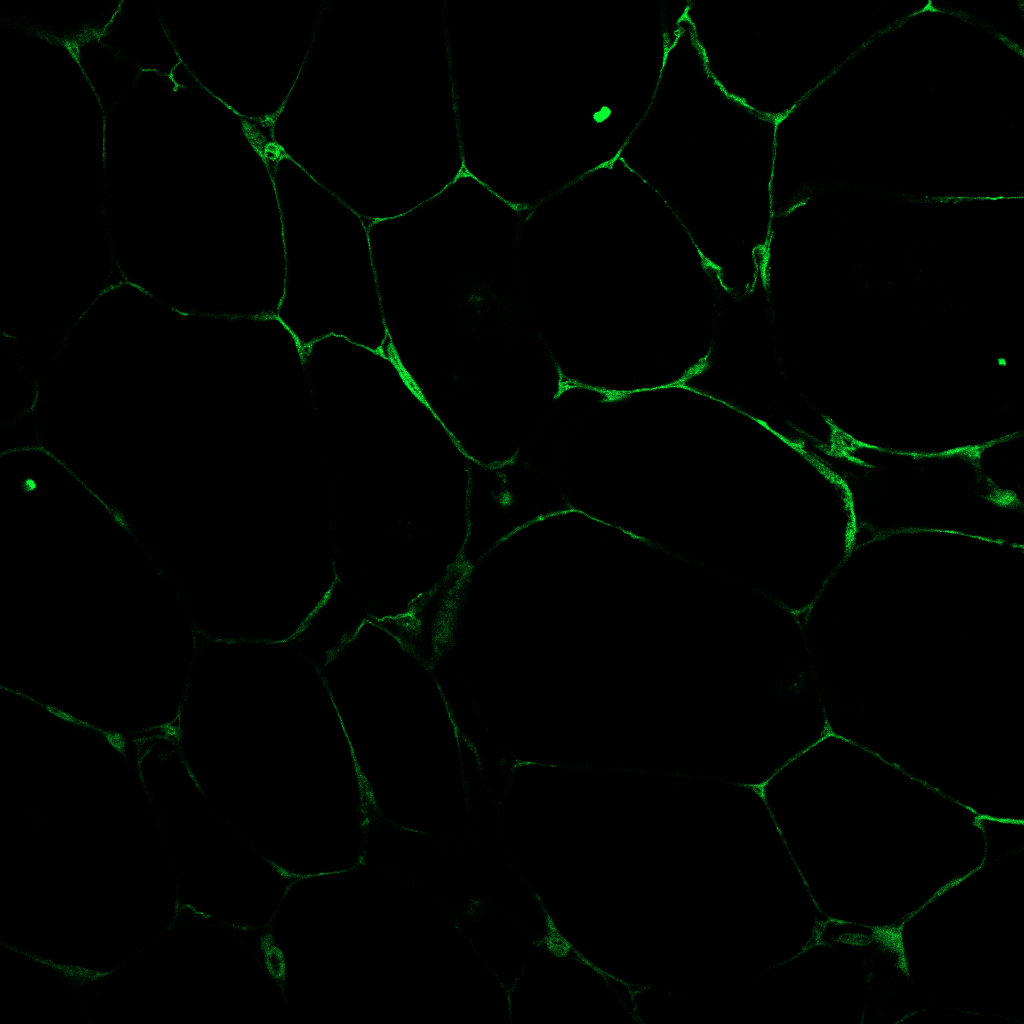

Supplement: Supplementary file 10 — Source data Fig. 8 [file 44318_2025_508_MOESM10_ESM.zip › Source data Fig.8/Figure 8O/HFD-Sulprostone/HFD-Sulprostone-SPARC.tif]

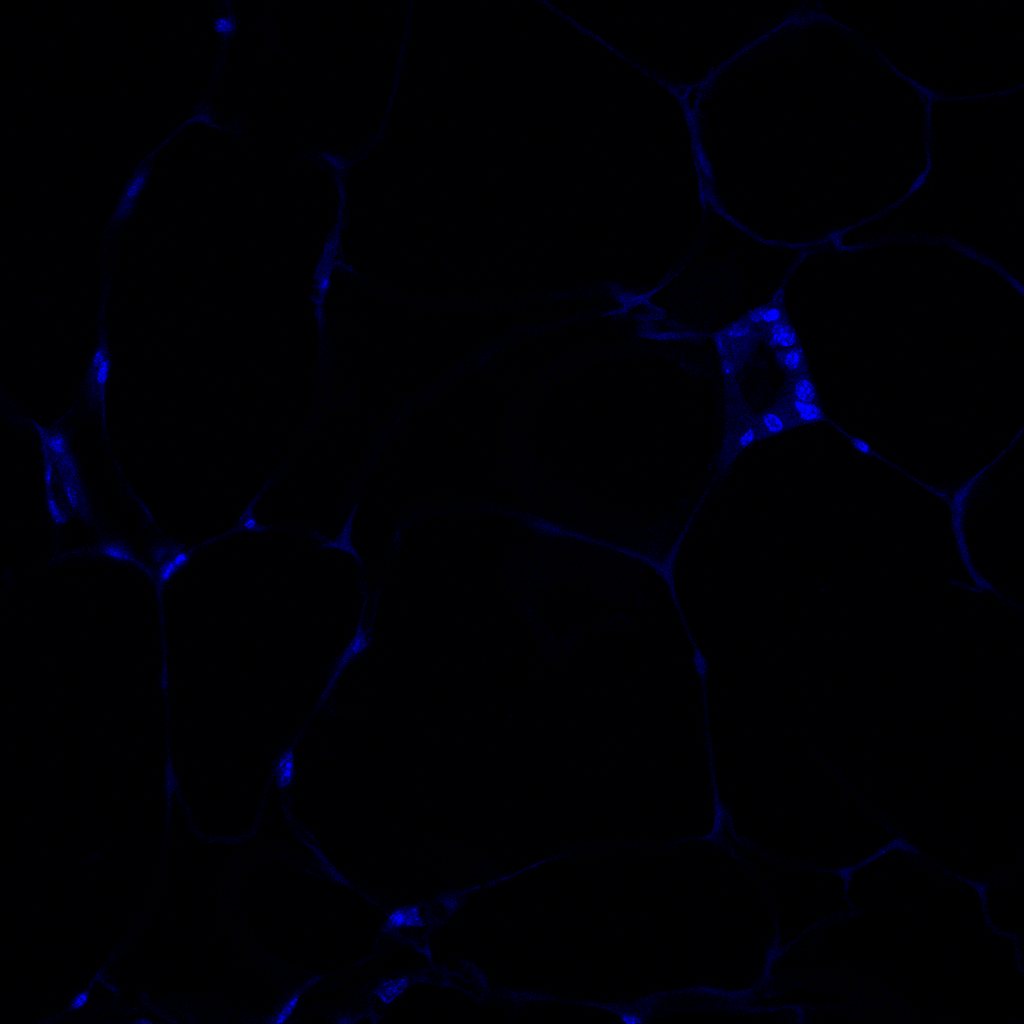

Supplement: Supplementary file 10 — Source data Fig. 8 [file 44318_2025_508_MOESM10_ESM.zip › Source data Fig.8/Figure 8O/HFD-Vehicle/HFD-Vehicle-DAPI.tif]

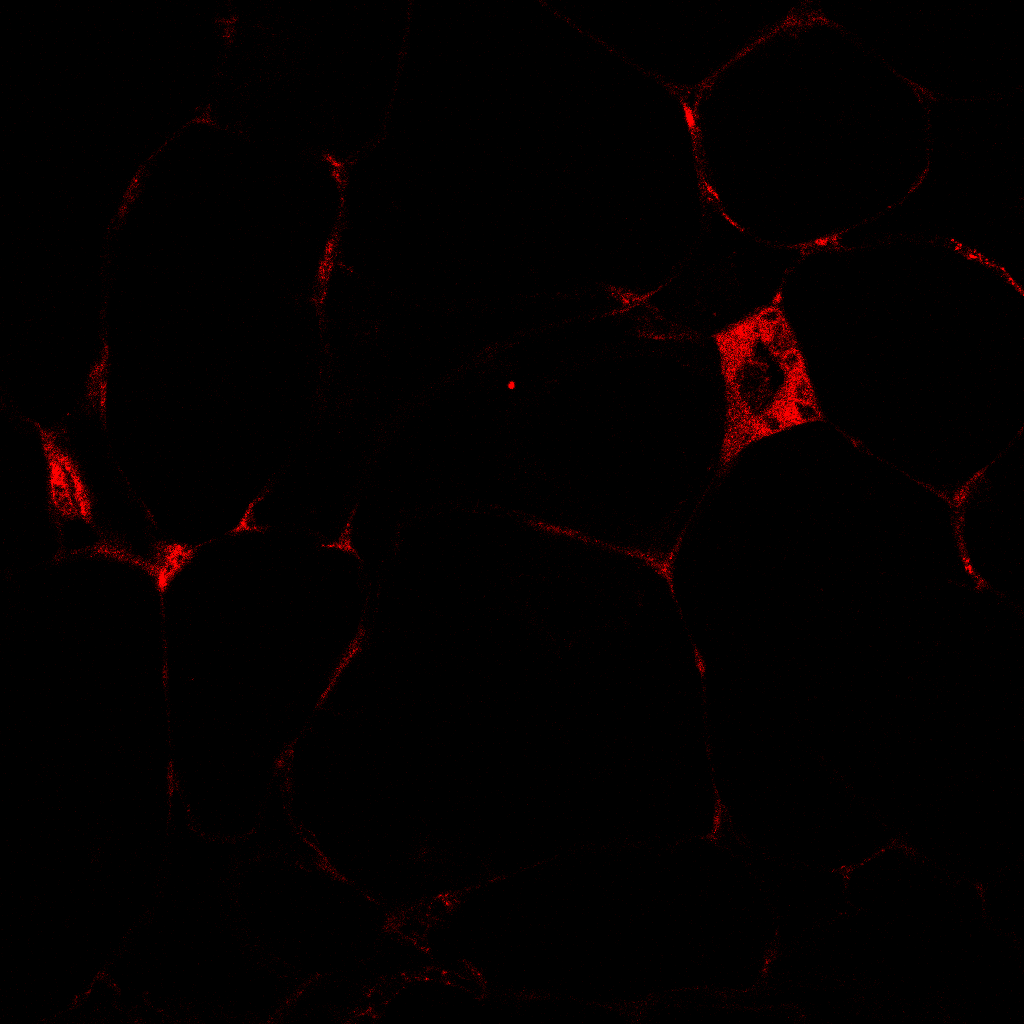

Supplement: Supplementary file 10 — Source data Fig. 8 [file 44318_2025_508_MOESM10_ESM.zip › Source data Fig.8/Figure 8O/HFD-Vehicle/HFD-Vehicle-MAC-3.tif]

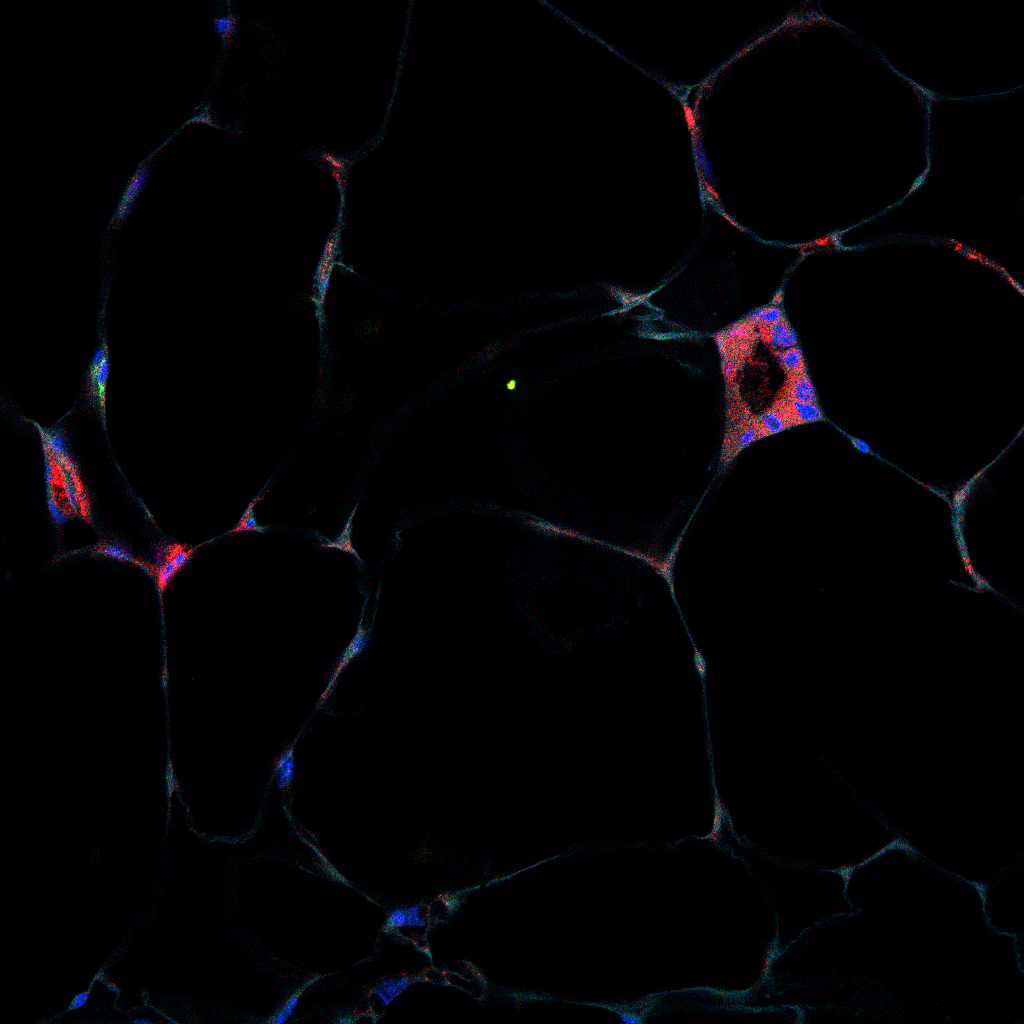

Supplement: Supplementary file 10 — Source data Fig. 8 [file 44318_2025_508_MOESM10_ESM.zip › Source data Fig.8/Figure 8O/HFD-Vehicle/HFD-Vehicle-Merge.tif]

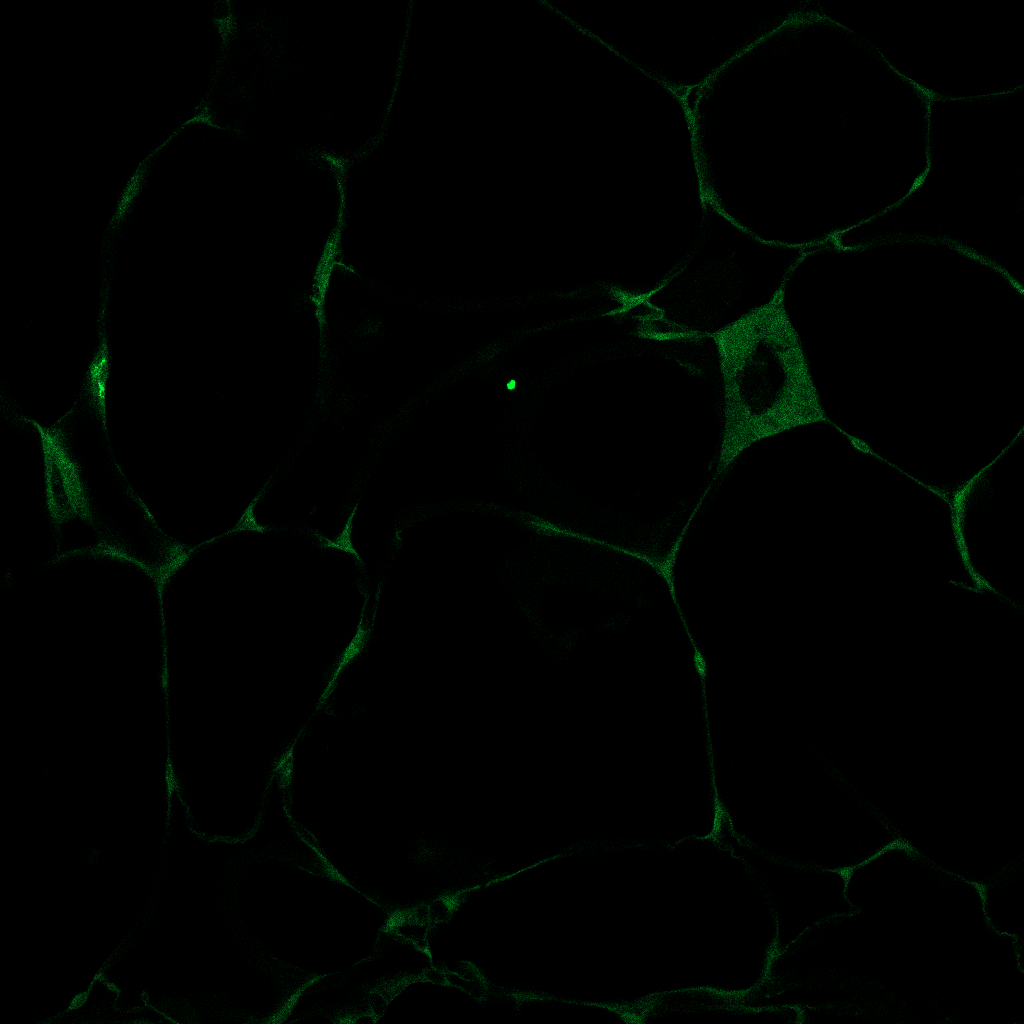

Supplement: Supplementary file 10 — Source data Fig. 8 [file 44318_2025_508_MOESM10_ESM.zip › Source data Fig.8/Figure 8O/HFD-Vehicle/HFD-Vehicle-SPARC.tif]

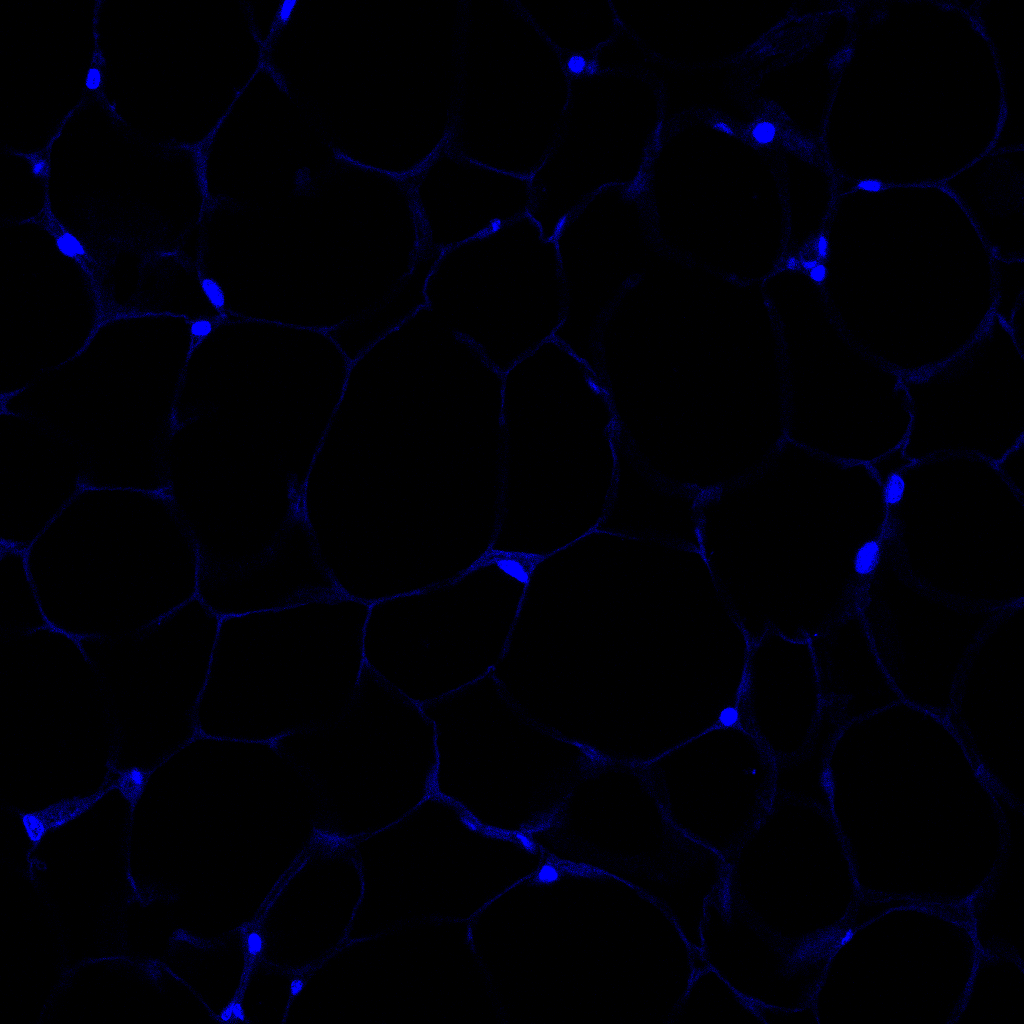

Supplement: Supplementary file 10 — Source data Fig. 8 [file 44318_2025_508_MOESM10_ESM.zip › Source data Fig.8/Figure 8O/NCD-Vehicle/NCD-Vehicle-DAPI.tif]

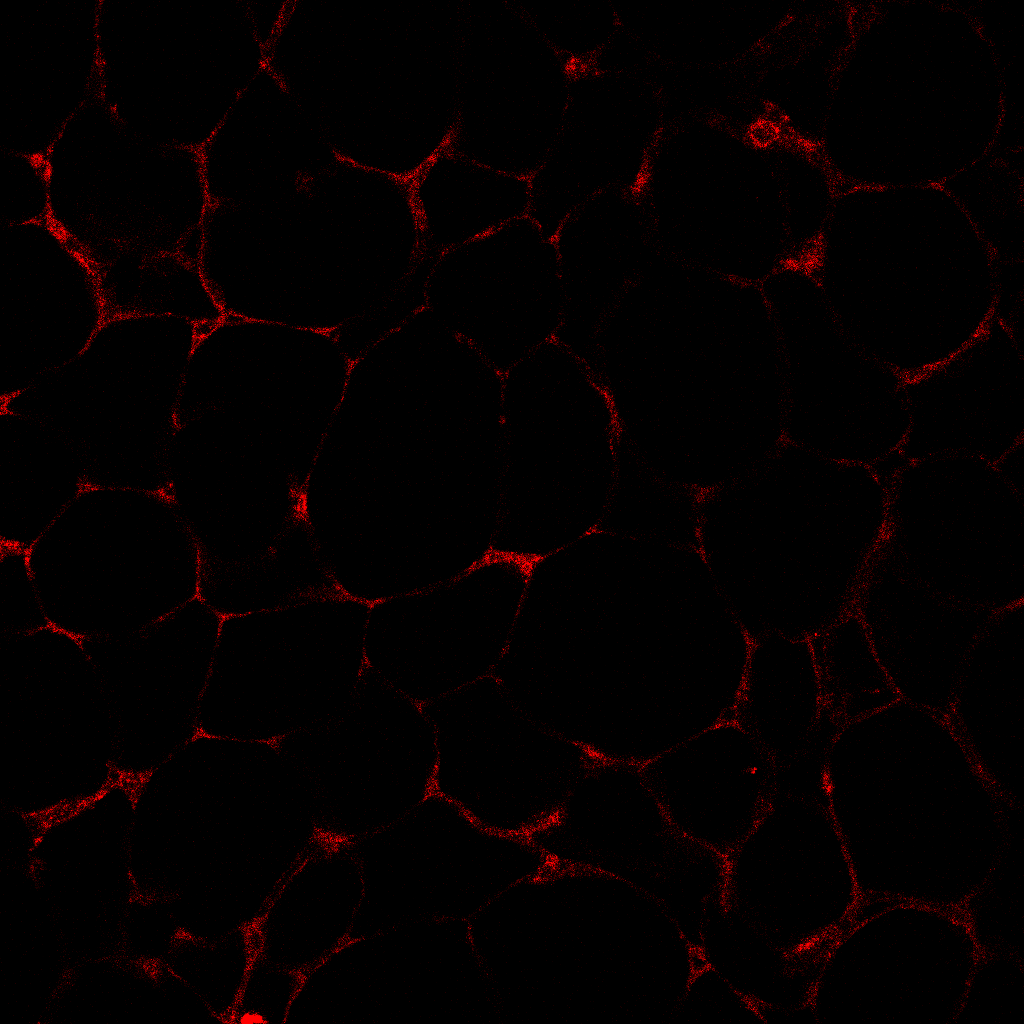

Supplement: Supplementary file 10 — Source data Fig. 8 [file 44318_2025_508_MOESM10_ESM.zip › Source data Fig.8/Figure 8O/NCD-Vehicle/NCD-Vehicle-MAC-3.tif]

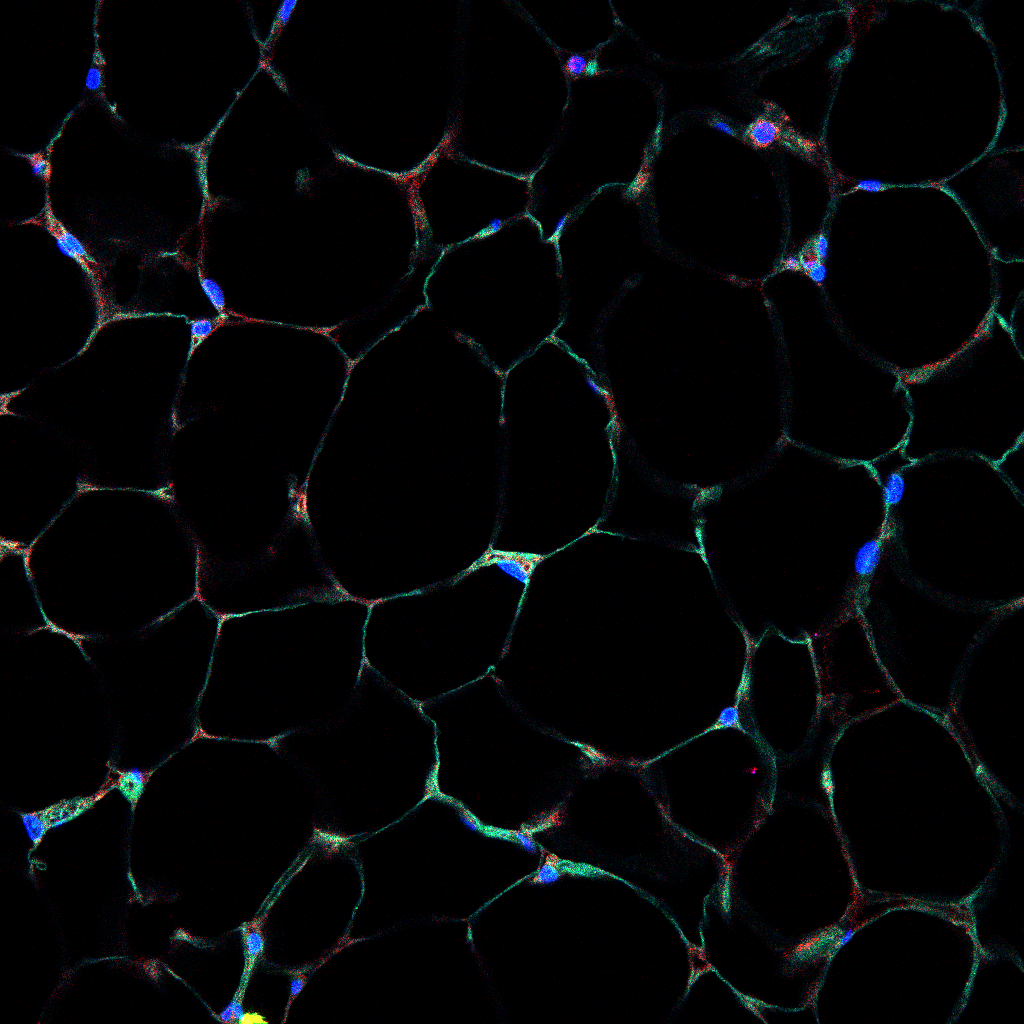

Supplement: Supplementary file 10 — Source data Fig. 8 [file 44318_2025_508_MOESM10_ESM.zip › Source data Fig.8/Figure 8O/NCD-Vehicle/NCD-Vehicle-Merge.tif]

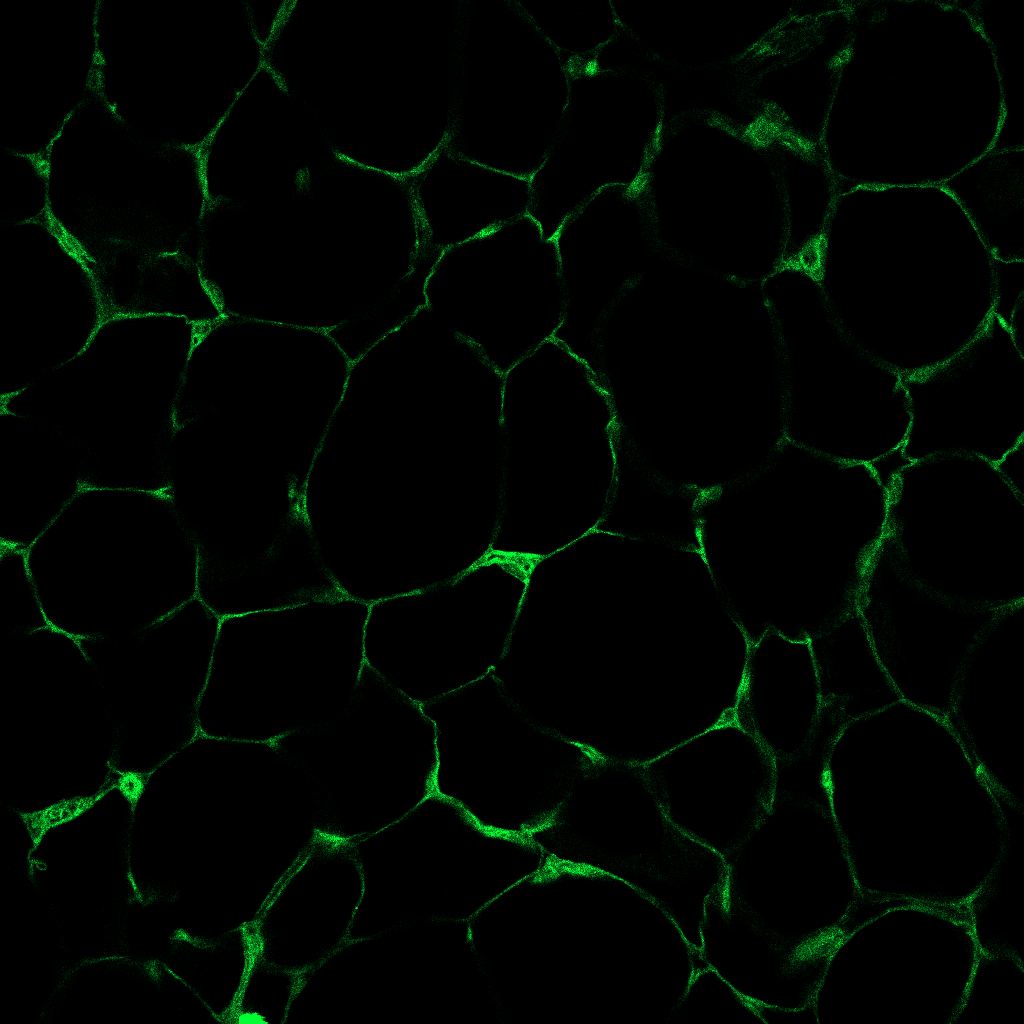

Supplement: Supplementary file 10 — Source data Fig. 8 [file 44318_2025_508_MOESM10_ESM.zip › Source data Fig.8/Figure 8O/NCD-Vehicle/NCD-Vehicle-SPARC.tif]

Appendix Figure S1A

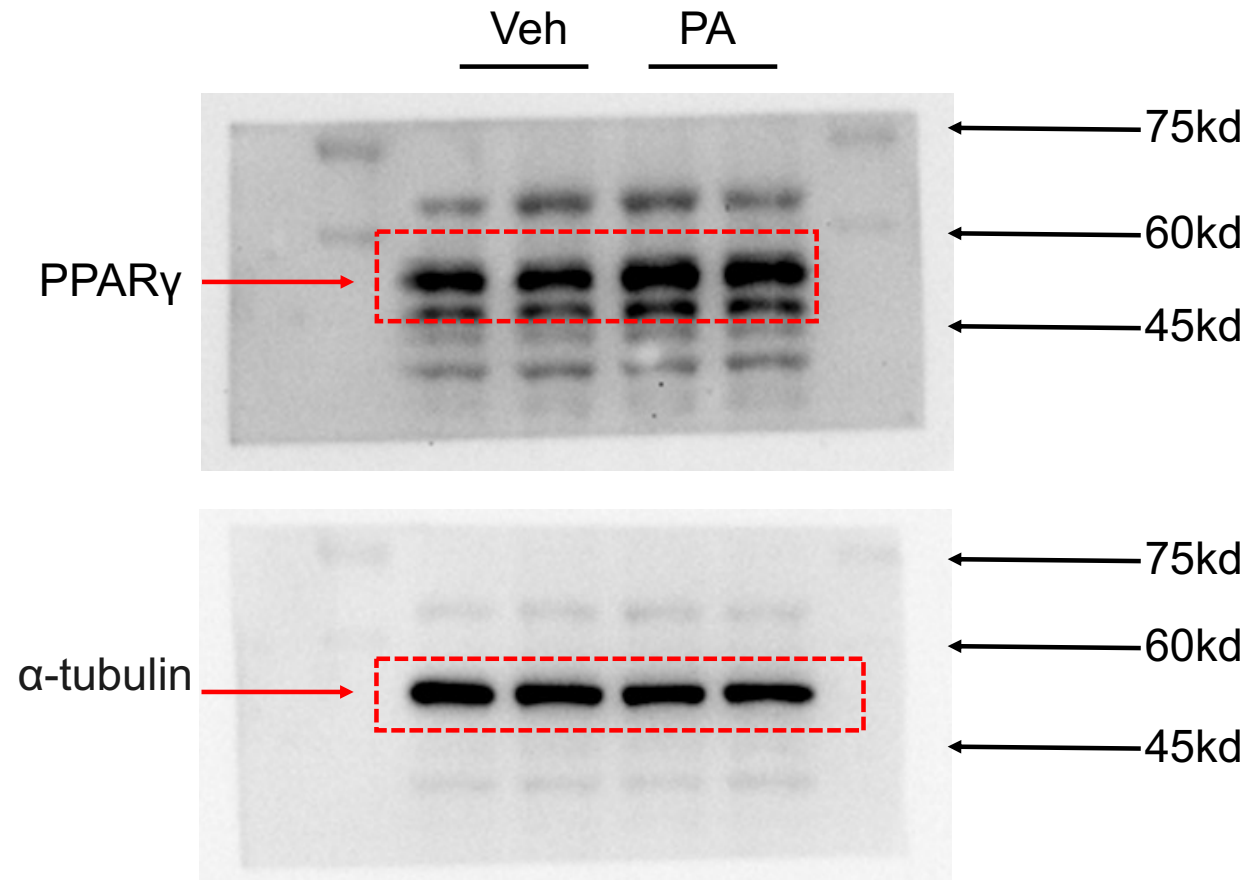

Supplement: Supplementary file 11 — Appendix Figure Source Data [file 44318_2025_508_MOESM11_ESM.zip › Source data Appendix Figure/Appendix Figure S1/Appendix Figure S1A/Appendix Figure S1A.pdf]

Appendix Figure S1B

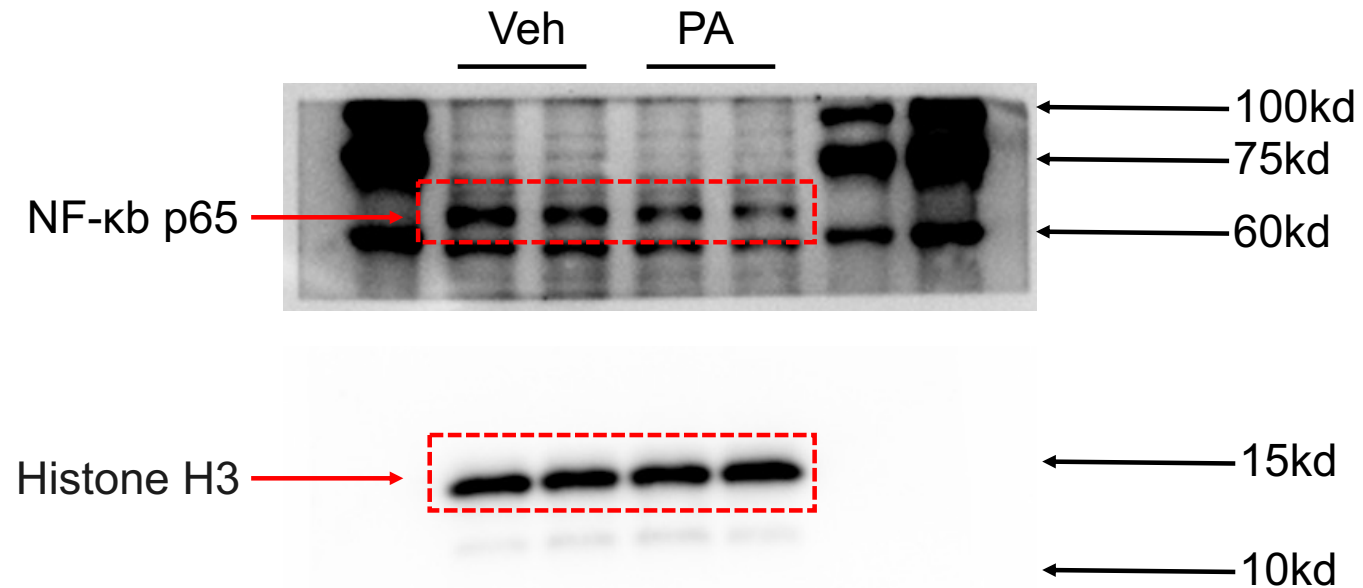

Supplement: Supplementary file 11 — Appendix Figure Source Data [file 44318_2025_508_MOESM11_ESM.zip › Source data Appendix Figure/Appendix Figure S1/Appendix Figure S1B/Appendix Figure S1B.pdf]

Appendix Figure S10A

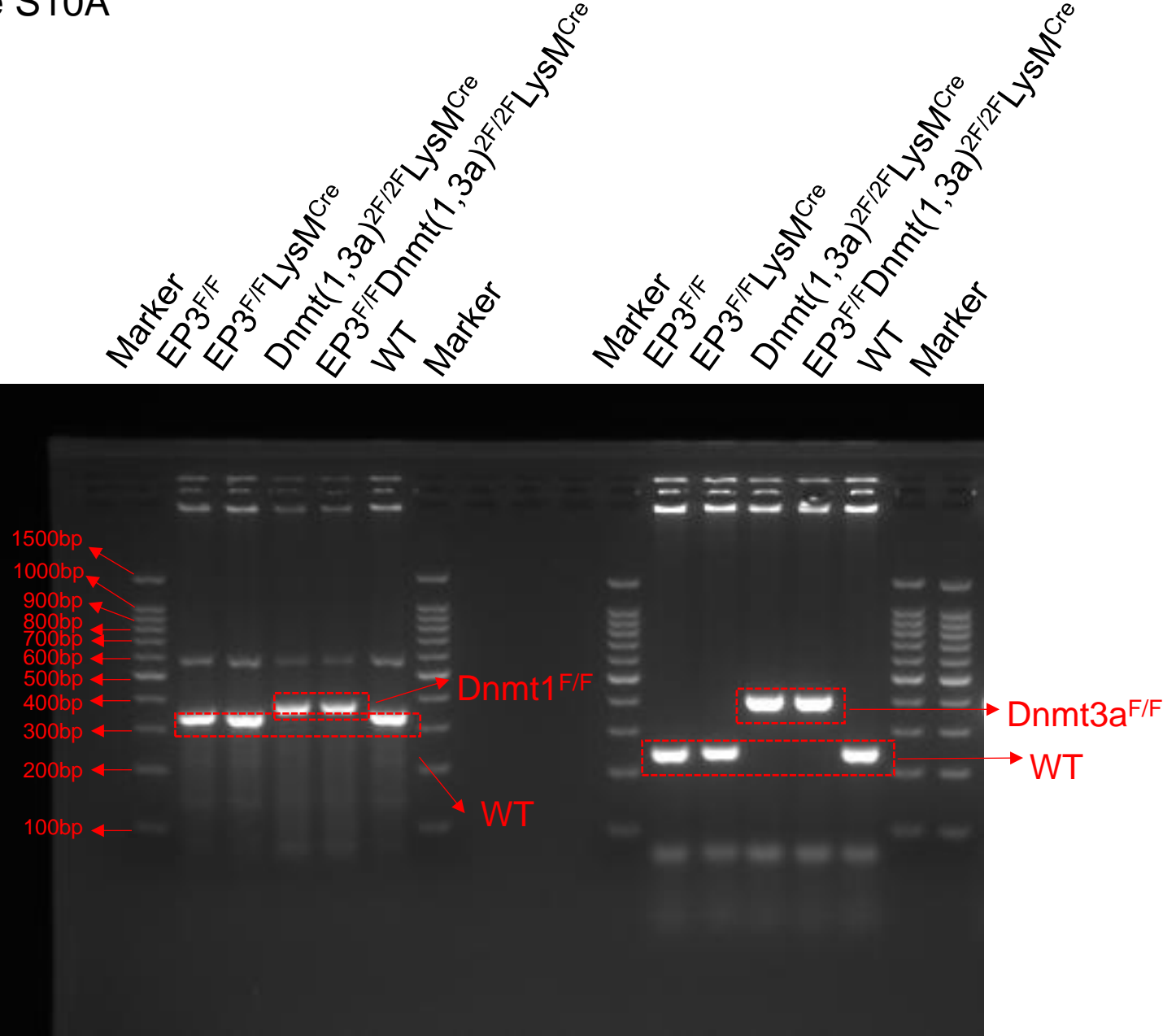

Appendix Figure S10A

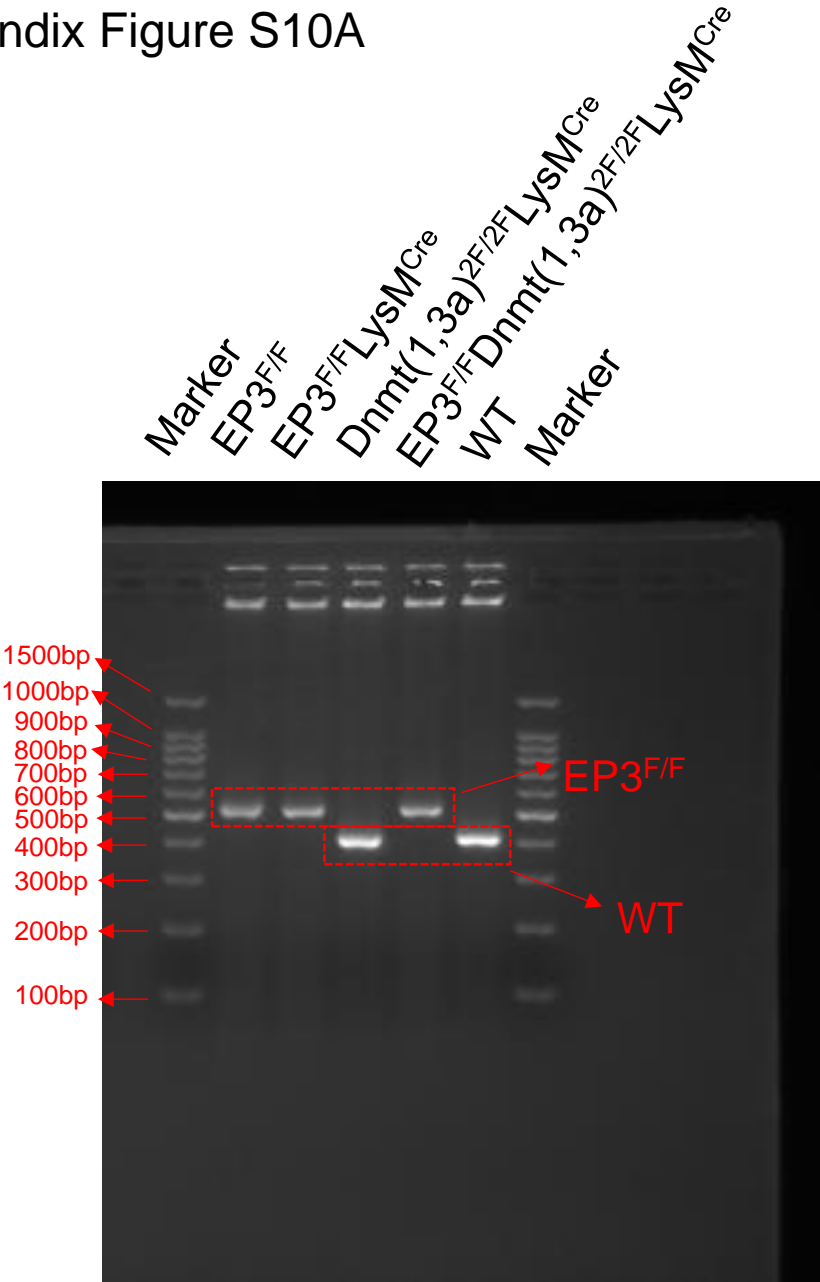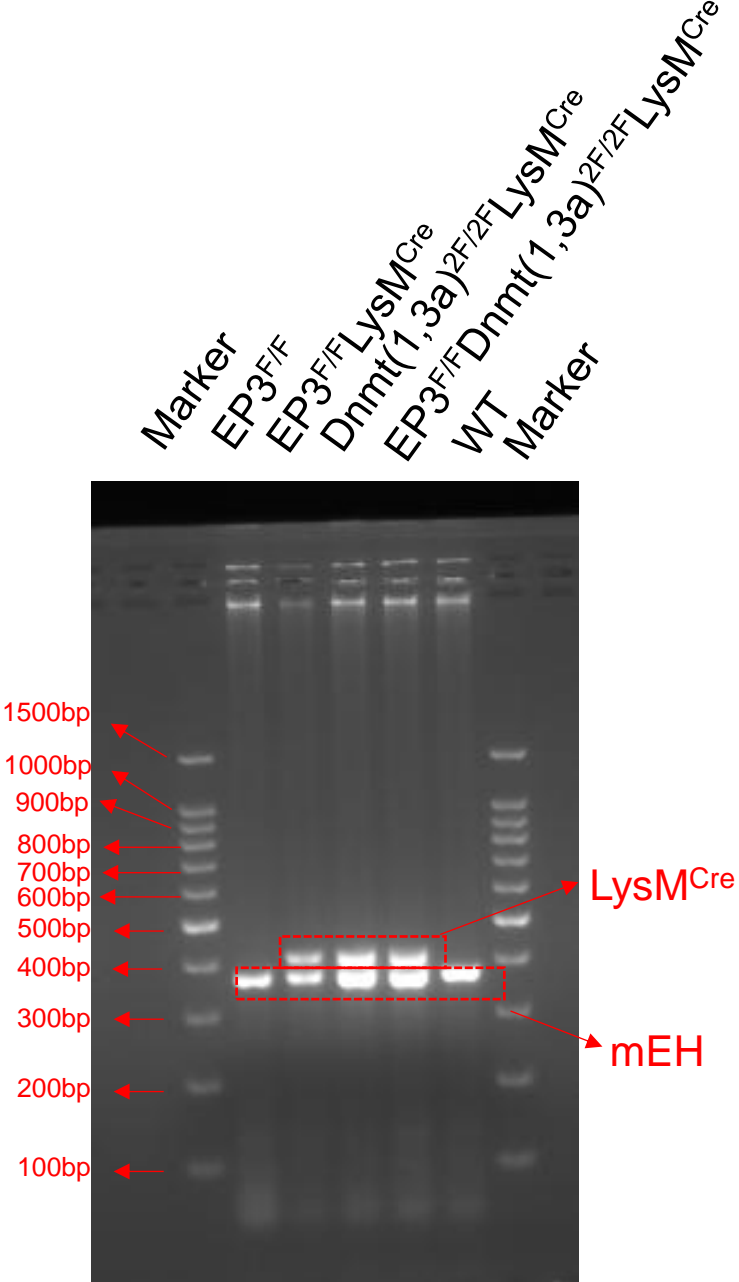

Supplement: Supplementary file 11 — Appendix Figure Source Data [file 44318_2025_508_MOESM11_ESM.zip › Source data Appendix Figure/Appendix Figure S10/Appendix Figure S10A/Appendix Figure S10A.pdf]

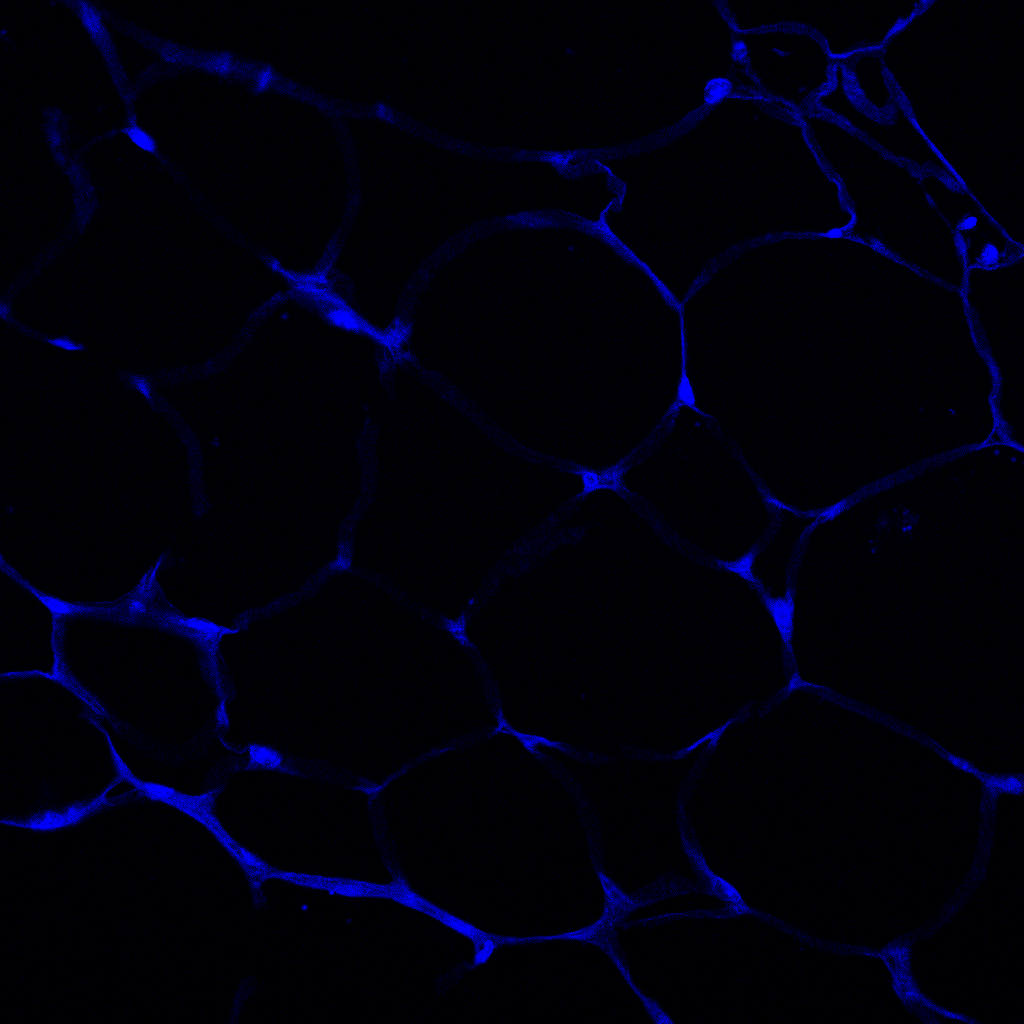

Supplement: Supplementary file 11 — Appendix Figure Source Data [file 44318_2025_508_MOESM11_ESM.zip › Source data Appendix Figure/Appendix Figure S12/Appendix Figure S12H/HFD-Sulprostone/HFD-Sulprostone-DAPI.tif]

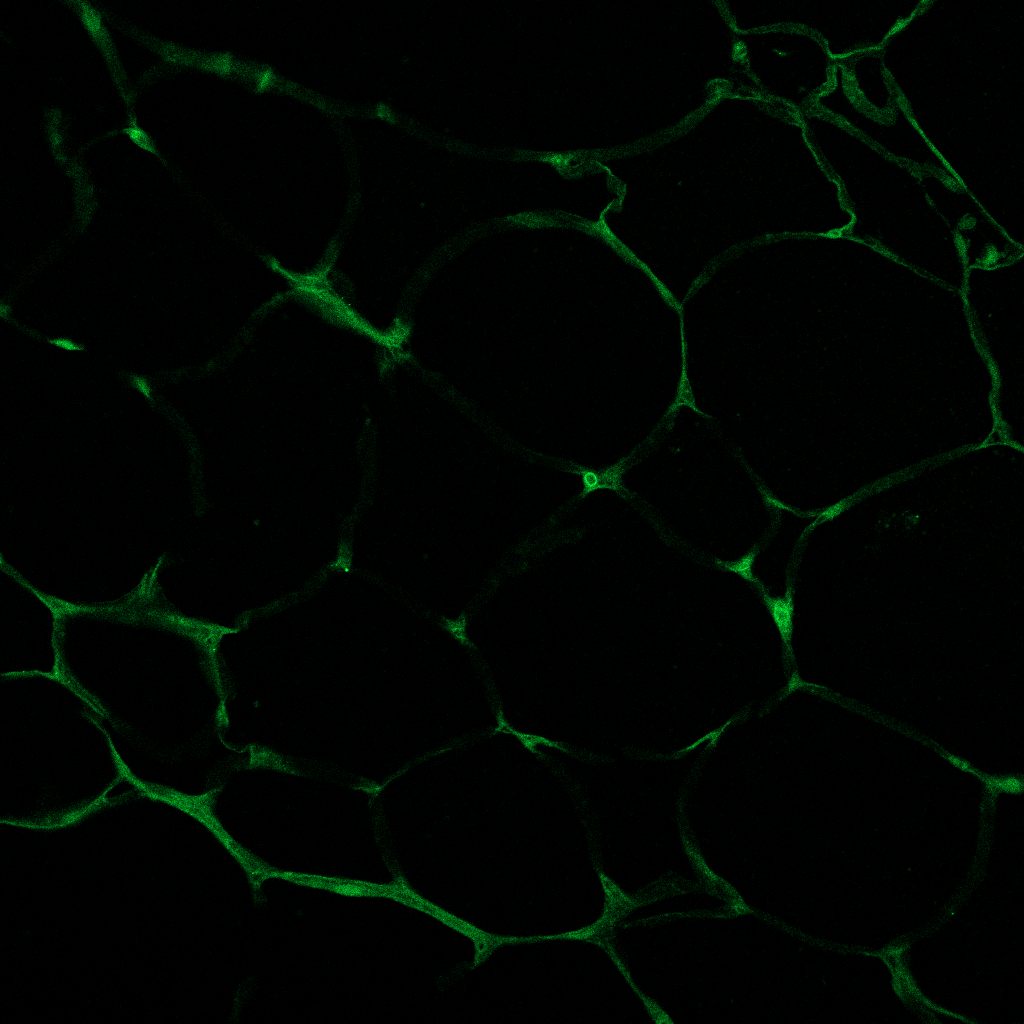

Supplement: Supplementary file 11 — Appendix Figure Source Data [file 44318_2025_508_MOESM11_ESM.zip › Source data Appendix Figure/Appendix Figure S12/Appendix Figure S12H/HFD-Sulprostone/HFD-Sulprostone-Dnmt3a.tif]

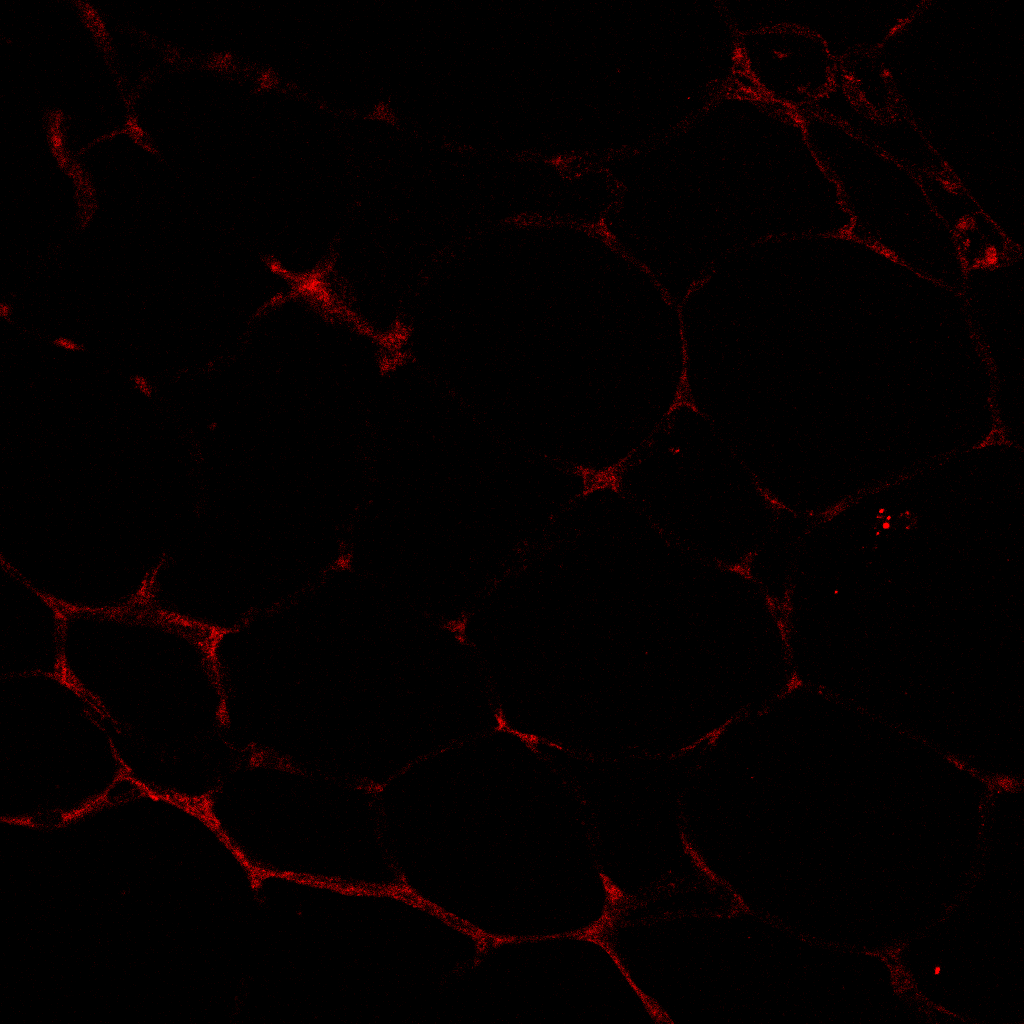

Supplement: Supplementary file 11 — Appendix Figure Source Data [file 44318_2025_508_MOESM11_ESM.zip › Source data Appendix Figure/Appendix Figure S12/Appendix Figure S12H/HFD-Sulprostone/HFD-Sulprostone-Mac-3.tif]

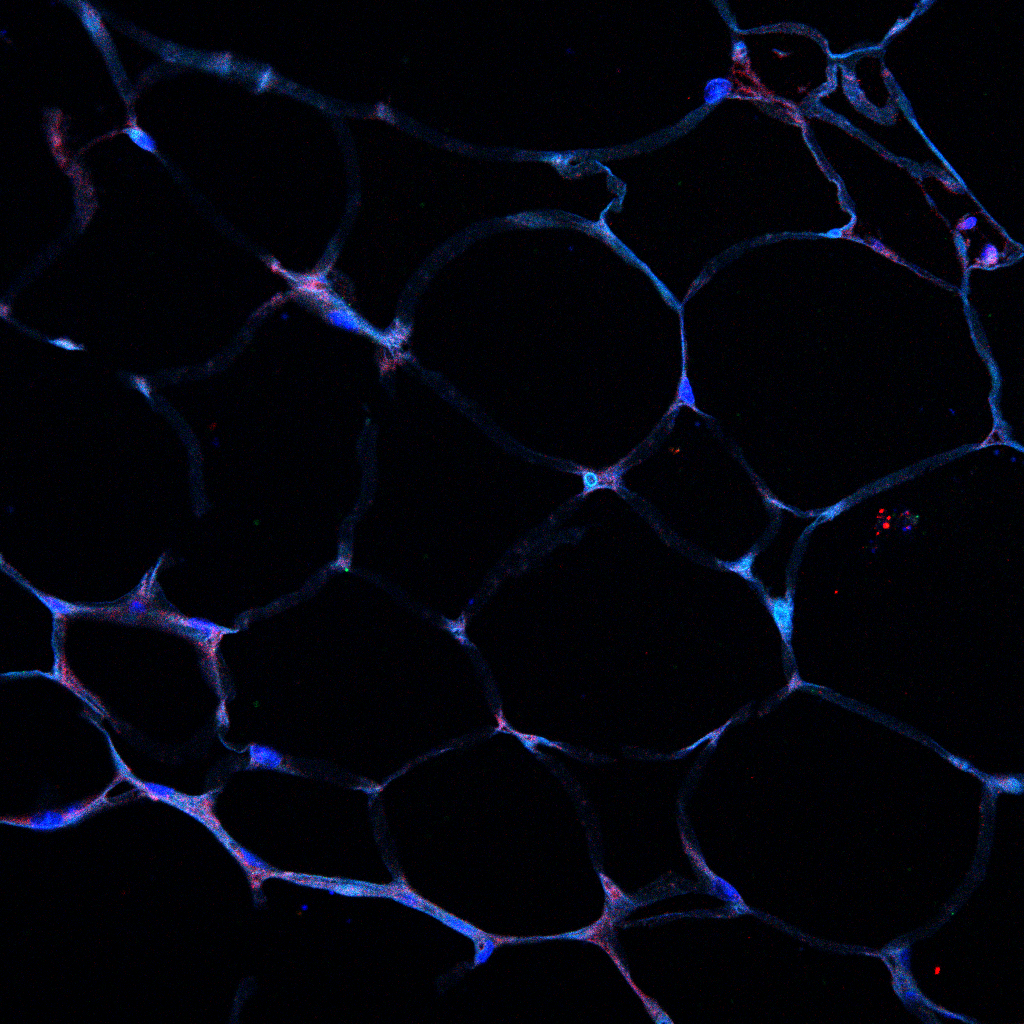

Supplement: Supplementary file 11 — Appendix Figure Source Data [file 44318_2025_508_MOESM11_ESM.zip › Source data Appendix Figure/Appendix Figure S12/Appendix Figure S12H/HFD-Sulprostone/HFD-Sulprostone-Merge.tif]

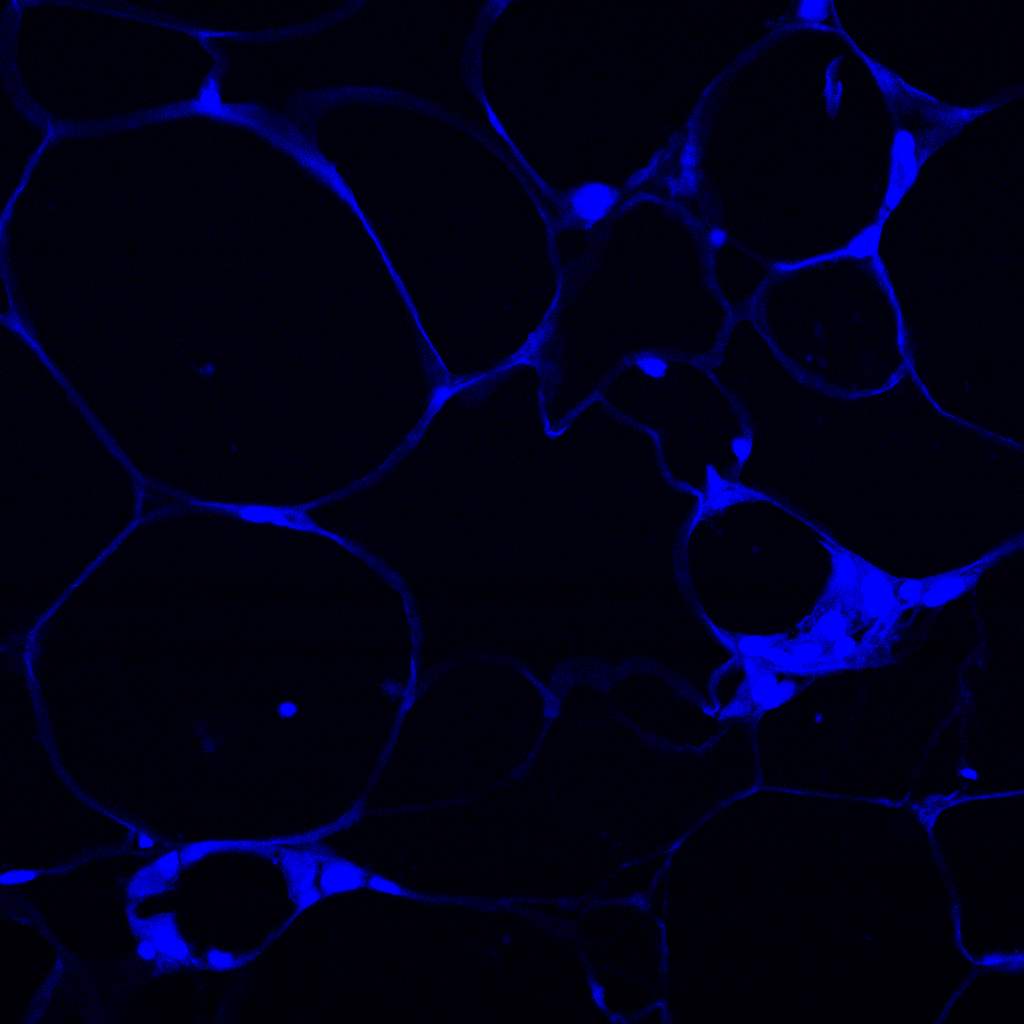

Supplement: Supplementary file 11 — Appendix Figure Source Data [file 44318_2025_508_MOESM11_ESM.zip › Source data Appendix Figure/Appendix Figure S12/Appendix Figure S12H/HFD-Vehicle/HFD-Vehicle-DAPI.tif]

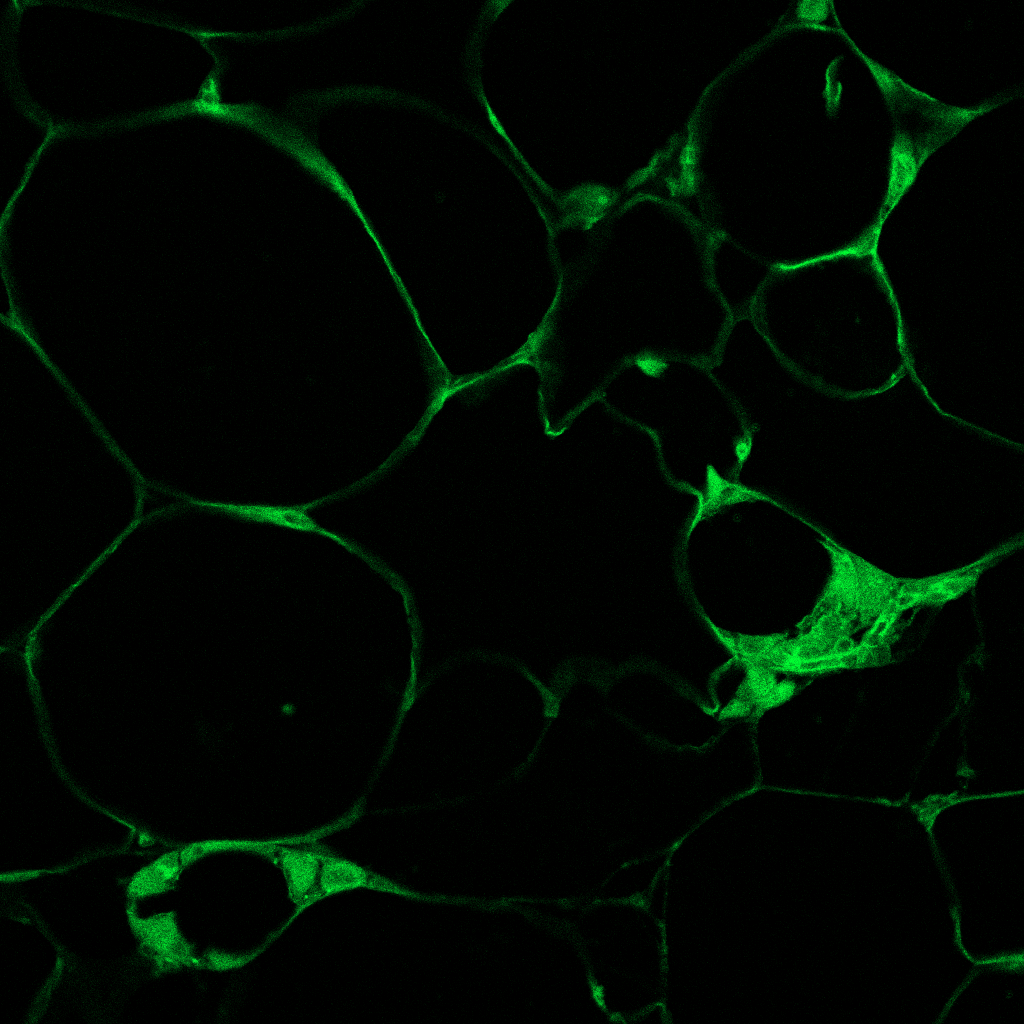

Supplement: Supplementary file 11 — Appendix Figure Source Data [file 44318_2025_508_MOESM11_ESM.zip › Source data Appendix Figure/Appendix Figure S12/Appendix Figure S12H/HFD-Vehicle/HFD-Vehicle-Dnmt3a.tif]

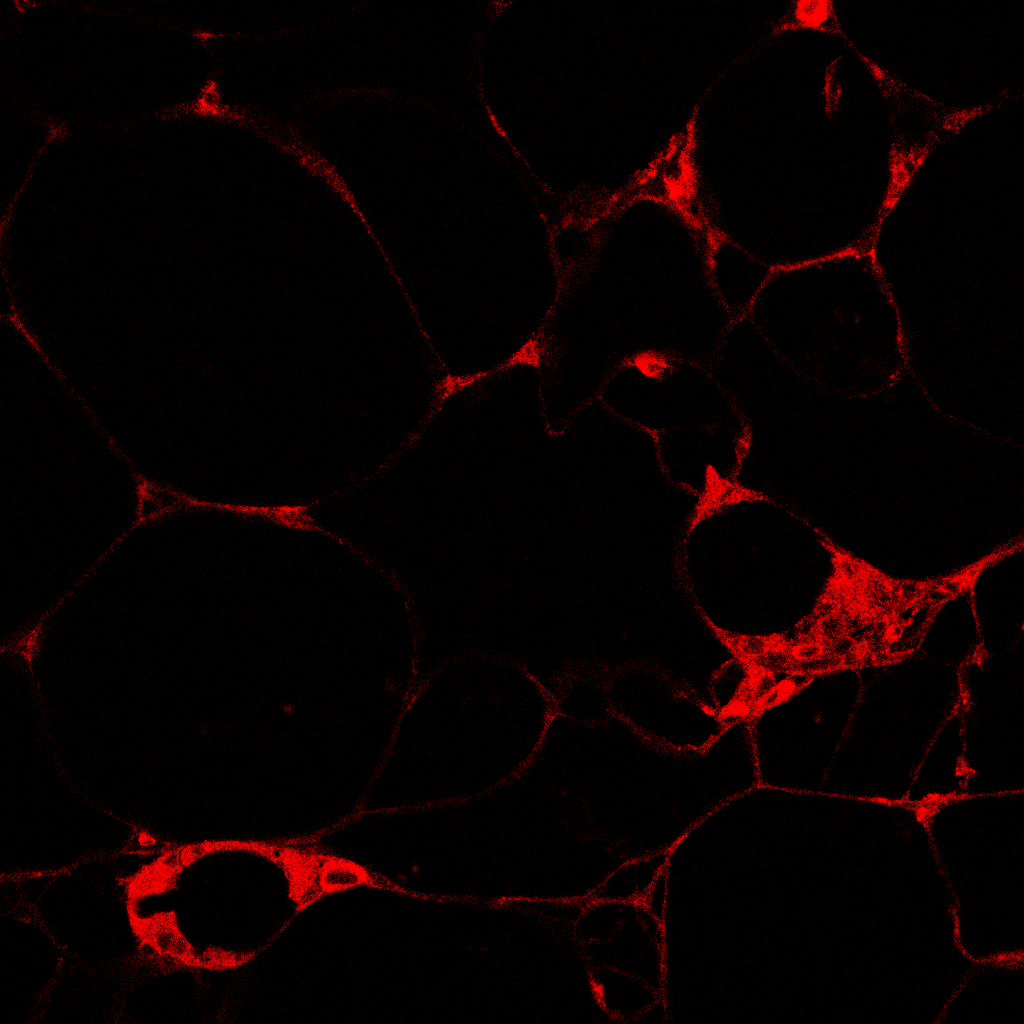

Supplement: Supplementary file 11 — Appendix Figure Source Data [file 44318_2025_508_MOESM11_ESM.zip › Source data Appendix Figure/Appendix Figure S12/Appendix Figure S12H/HFD-Vehicle/HFD-Vehicle-Mac-3.tif]

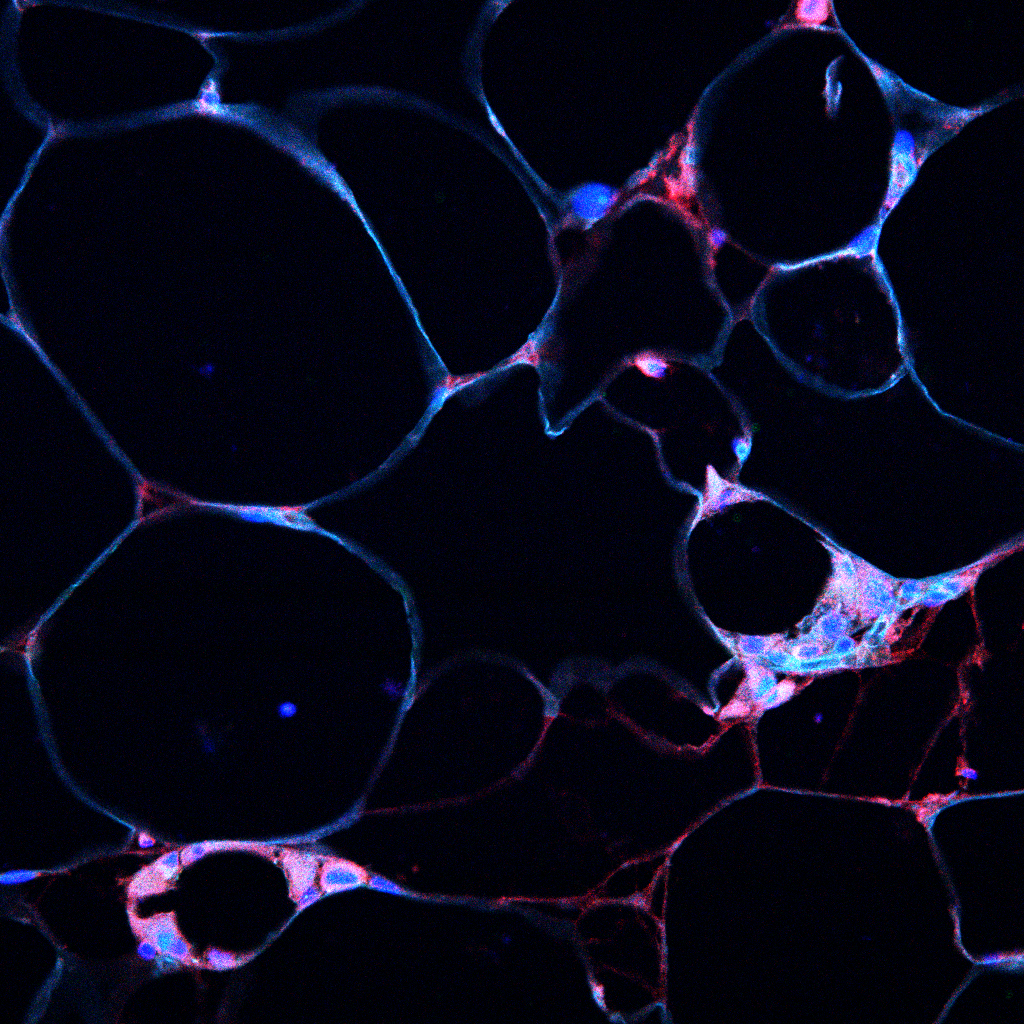

Supplement: Supplementary file 11 — Appendix Figure Source Data [file 44318_2025_508_MOESM11_ESM.zip › Source data Appendix Figure/Appendix Figure S12/Appendix Figure S12H/HFD-Vehicle/HFD-Vehicle-Merge.tif]

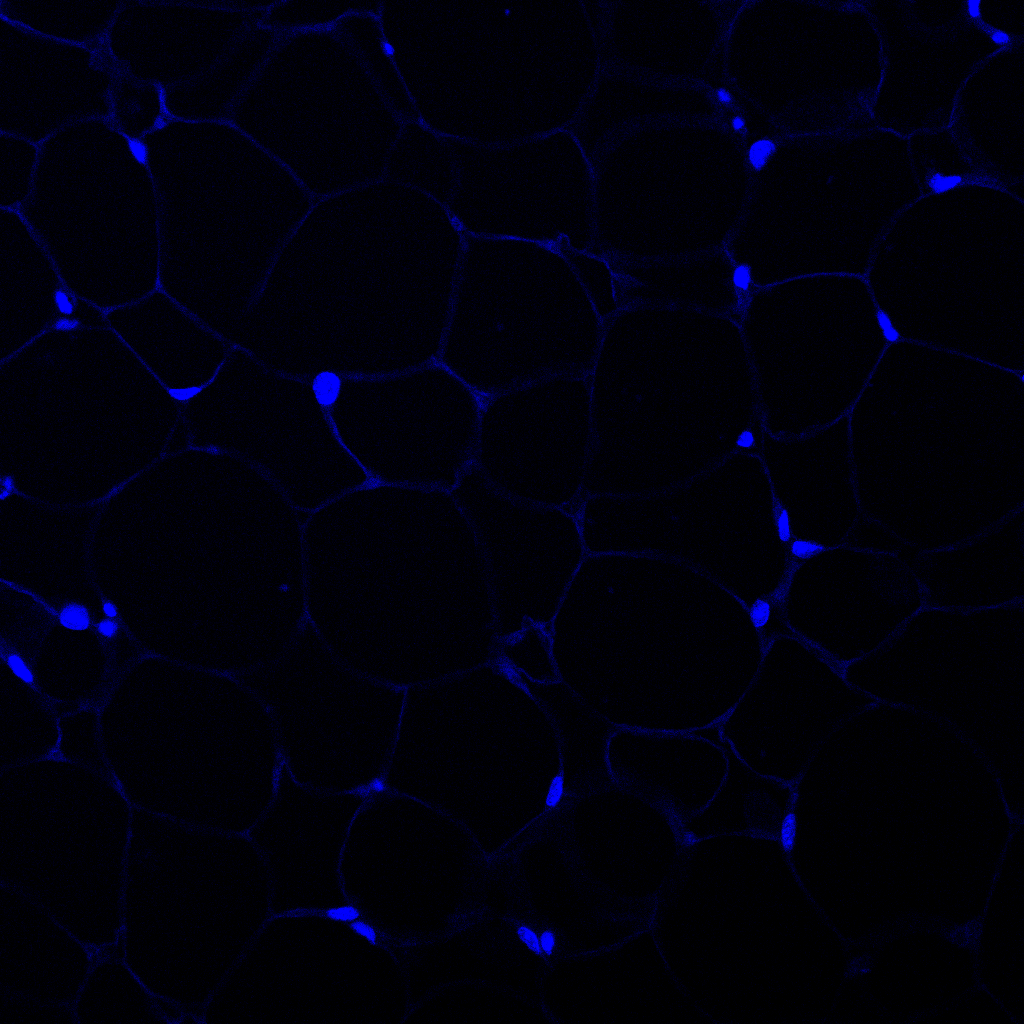

Supplement: Supplementary file 11 — Appendix Figure Source Data [file 44318_2025_508_MOESM11_ESM.zip › Source data Appendix Figure/Appendix Figure S12/Appendix Figure S12H/NCD-Vehicle/NCD-Vehicle-DAPI.tif]

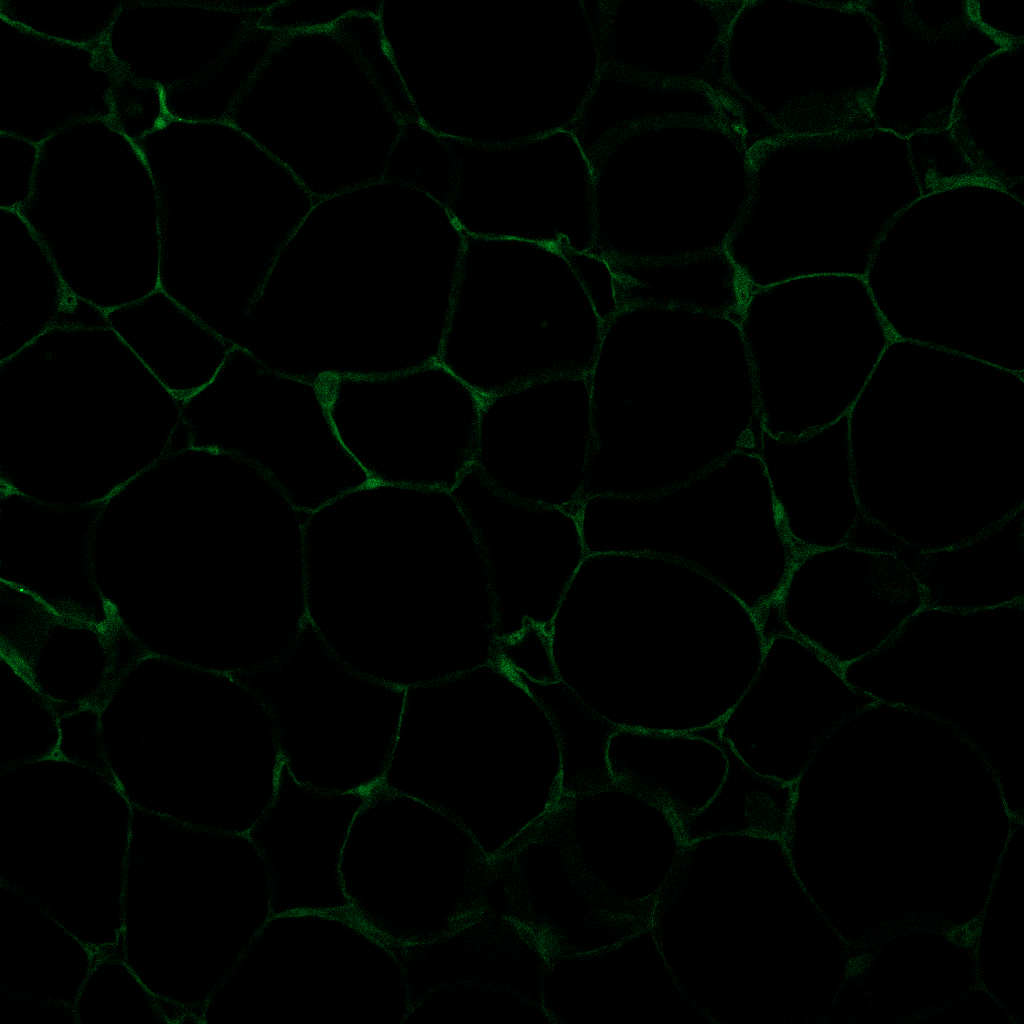

Supplement: Supplementary file 11 — Appendix Figure Source Data [file 44318_2025_508_MOESM11_ESM.zip › Source data Appendix Figure/Appendix Figure S12/Appendix Figure S12H/NCD-Vehicle/NCD-Vehicle-Dnmt3a.tif]

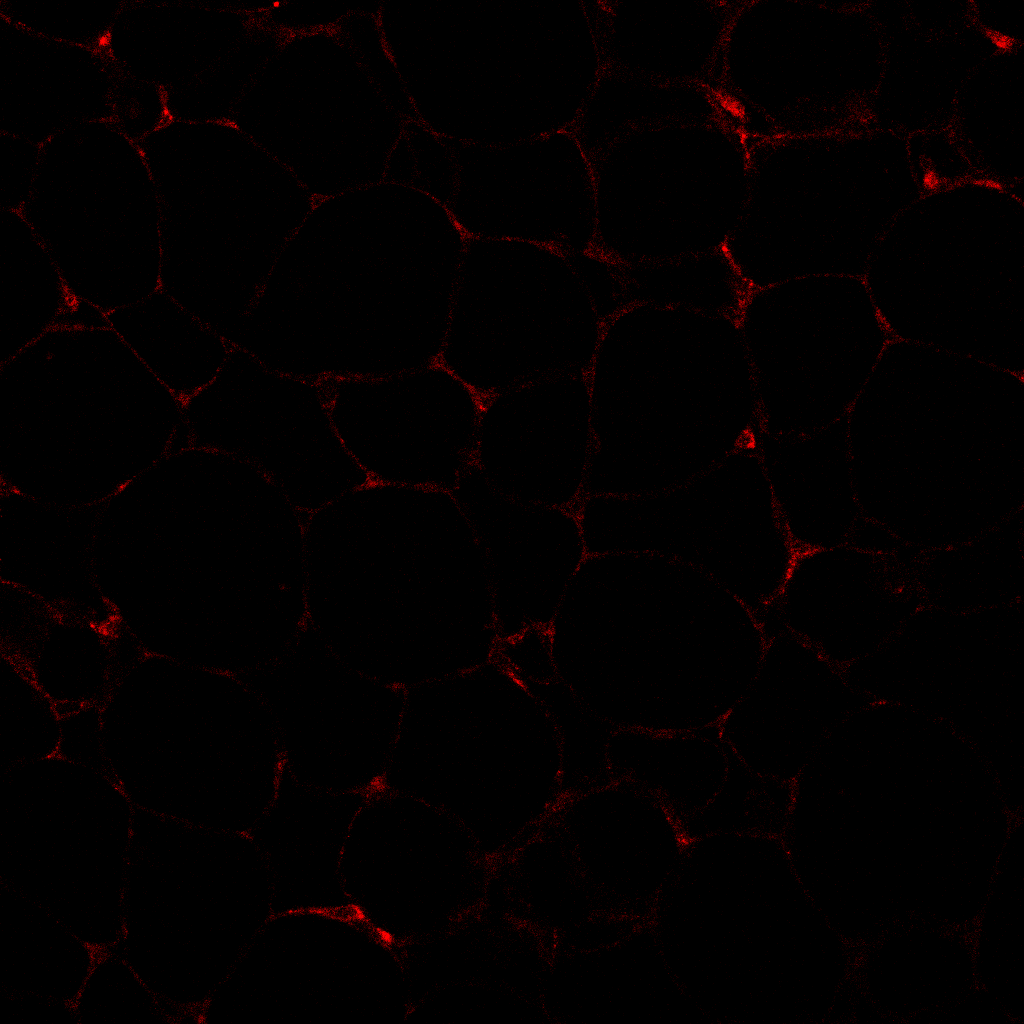

Supplement: Supplementary file 11 — Appendix Figure Source Data [file 44318_2025_508_MOESM11_ESM.zip › Source data Appendix Figure/Appendix Figure S12/Appendix Figure S12H/NCD-Vehicle/NCD-Vehicle-Mac-3.tif]

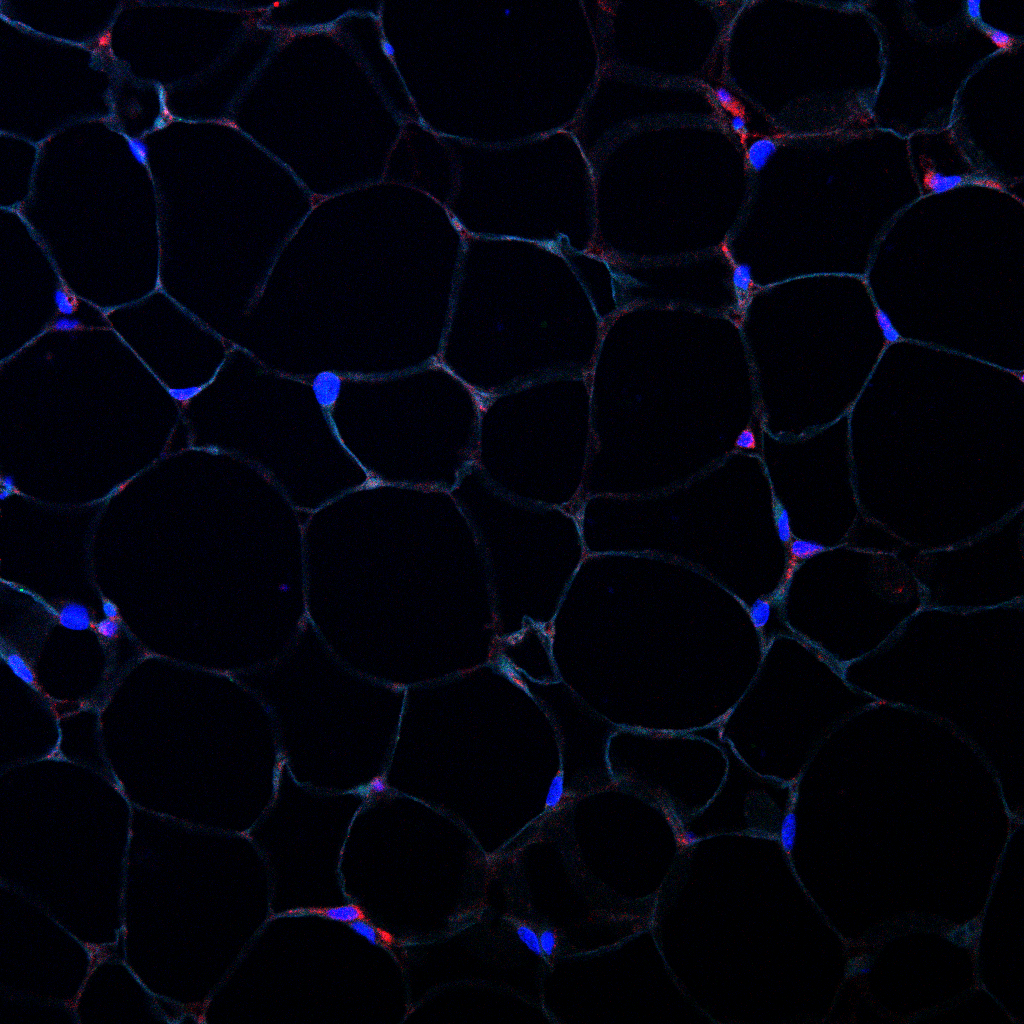

Supplement: Supplementary file 11 — Appendix Figure Source Data [file 44318_2025_508_MOESM11_ESM.zip › Source data Appendix Figure/Appendix Figure S12/Appendix Figure S12H/NCD-Vehicle/NCD-Vehicle-Merge.tif]

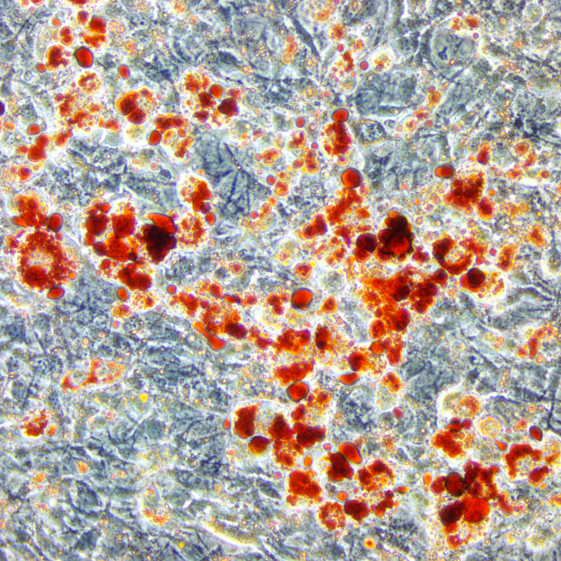

Supplement: Supplementary file 11 — Appendix Figure Source Data [file 44318_2025_508_MOESM11_ESM.zip › Source data Appendix Figure/Appendix Figure S2/Appendix Figure S2A/EP3KO-Sul.tif]

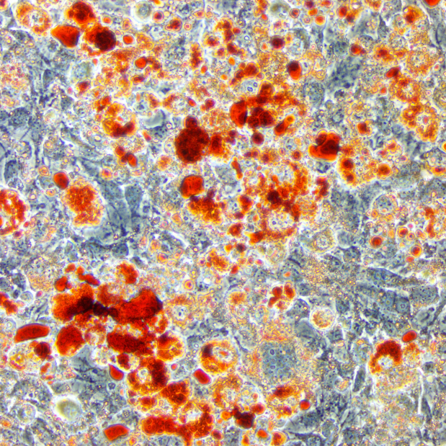

Supplement: Supplementary file 11 — Appendix Figure Source Data [file 44318_2025_508_MOESM11_ESM.zip › Source data Appendix Figure/Appendix Figure S2/Appendix Figure S2A/EP3KO-Veh.tif]

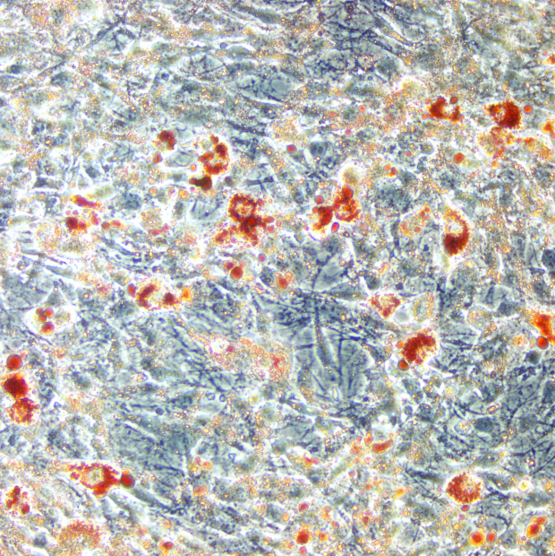

Supplement: Supplementary file 11 — Appendix Figure Source Data [file 44318_2025_508_MOESM11_ESM.zip › Source data Appendix Figure/Appendix Figure S2/Appendix Figure S2A/WT-Sul.tif]

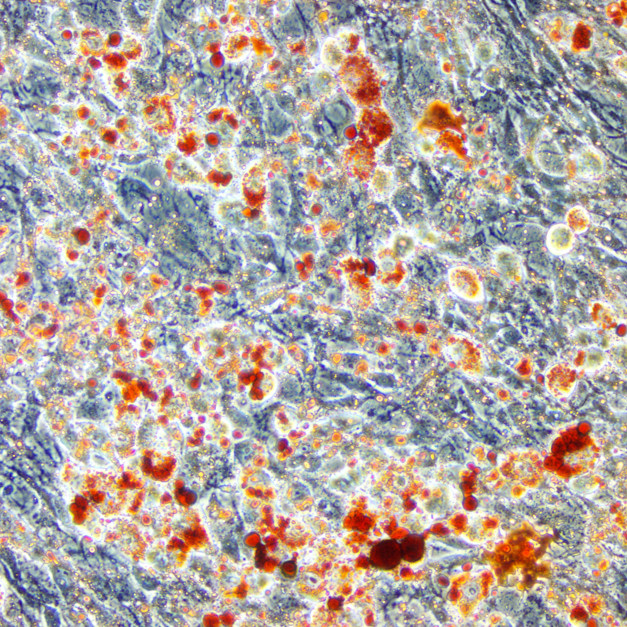

Supplement: Supplementary file 11 — Appendix Figure Source Data [file 44318_2025_508_MOESM11_ESM.zip › Source data Appendix Figure/Appendix Figure S2/Appendix Figure S2A/WT-Veh.tif]
