# Supplementary material for: Deletion of EP3 prostaglandin receptor in murine macrophages aggravates diet-induced obesity by suppressing SPARC
Source: EMBO J. 2025 Jul 23;44(18):4962–83. doi: 10.1038/s44318-025-00508-y (PMC12436609; doi:10.1038/s44318-025-00508-y)
Supplement: Supplementary file 11 — Appendix Figure Source Data [file 44318_2025_508_MOESM11_ESM.zip › Source data Appendix Figure/Appendix Figure S10/Appendix Figure S10A/Appendix Figure S10A.pptx]

## Slide 1
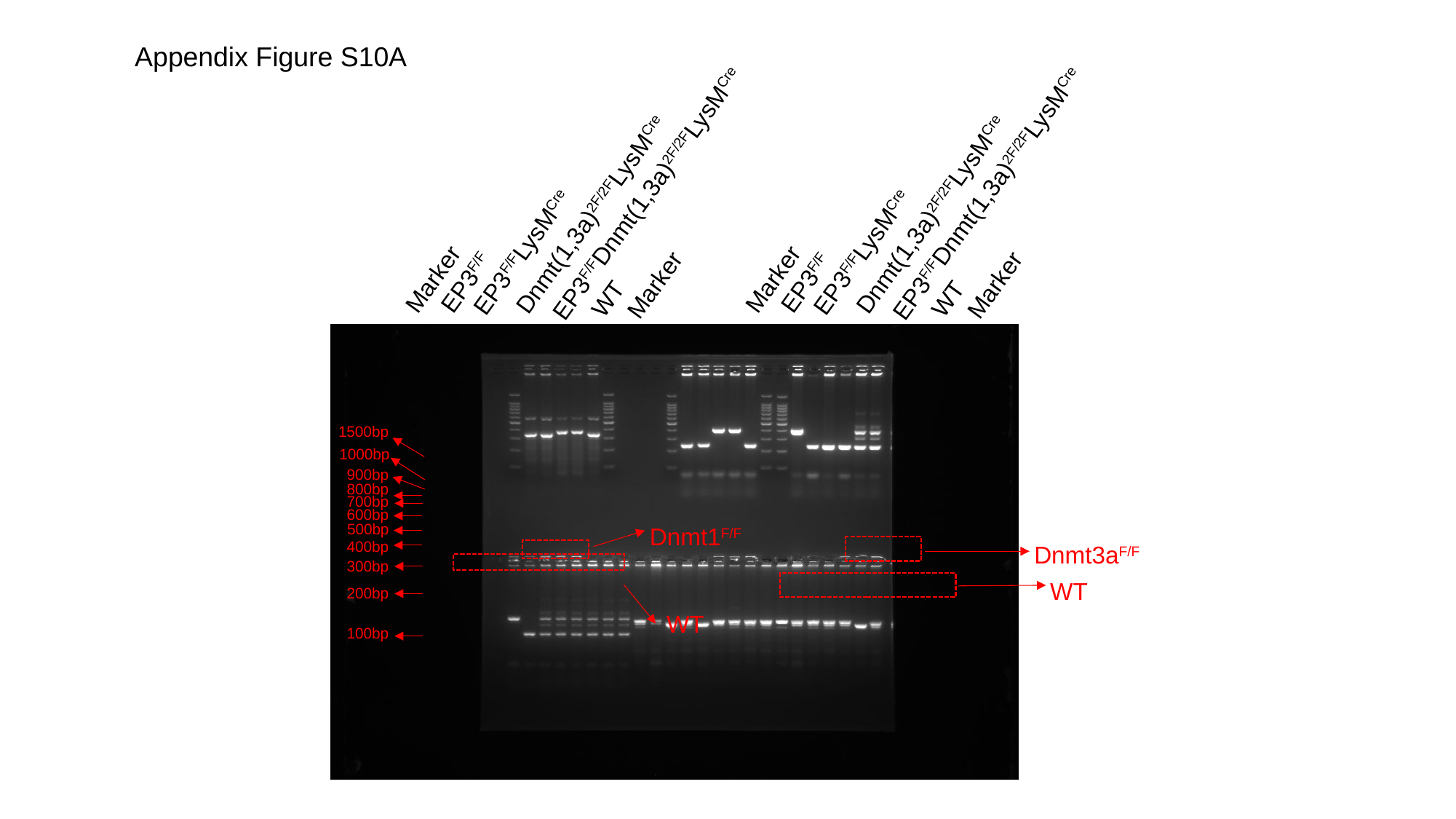

EP3F/FDnmt(1,3a)2F/2FLysMCre
Dnmt(1,3a)2F/2FLysMCre
EP3F/FLysMCre
EP3F/F
WT
Marker
Marker
EP3F/FDnmt(1,3a)2F/2FLysMCre
Dnmt(1,3a)2F/2FLysMCre
EP3F/FLysMCre
EP3F/F
WT
Marker
Marker
1500bp
1000bp
900bp
800bp
700bp
600bp
500bp
400bp
300bp
200bp
100bp
Dnmt1F/F
Dnmt3aF/F
WT
WT
Appendix Figure S10A

## Slide 2
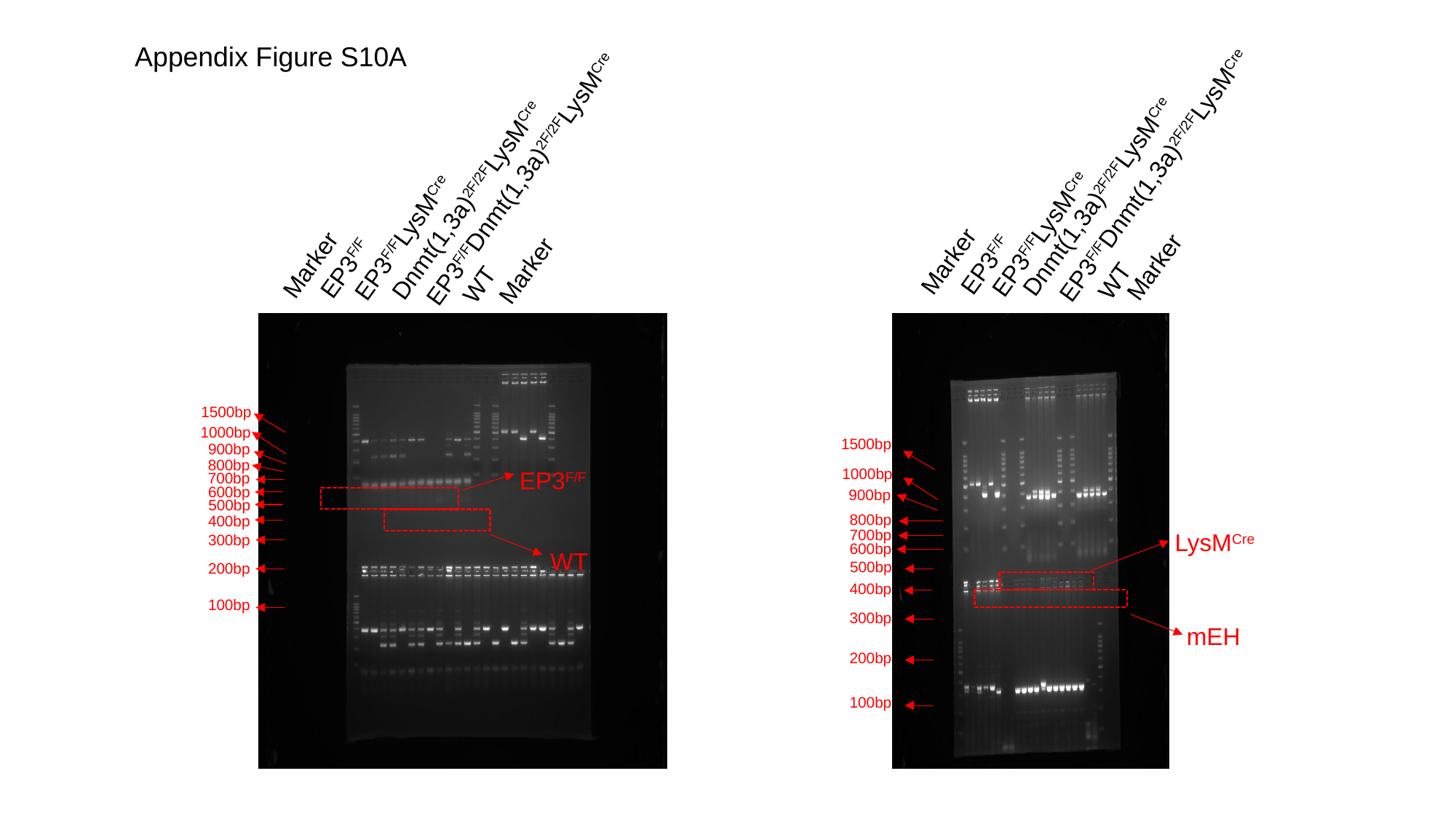

EP3F/FDnmt(1,3a)2F/2FLysMCre
Dnmt(1,3a)2F/2FLysMCre
EP3F/FLysMCre
EP3F/F
WT
Marker
Marker
1500bp
1000bp
900bp
800bp
700bp
LysMCre
600bp
500bp
400bp
300bp
mEH
200bp
100bp
EP3F/FDnmt(1,3a)2F/2FLysMCre
Dnmt(1,3a)2F/2FLysMCre
EP3F/FLysMCre
EP3F/F
WT
Marker
Marker
1500bp
1000bp
900bp
800bp
EP3F/F
700bp
600bp
500bp
400bp
300bp
WT
200bp
100bp
Appendix Figure S10A
