# Supplementary material for: Deletion of EP3 prostaglandin receptor in murine macrophages aggravates diet-induced obesity by suppressing SPARC
Source: EMBO J. 2025 Jul 23;44(18):4962–83. doi: 10.1038/s44318-025-00508-y (PMC12436609; doi:10.1038/s44318-025-00508-y)
Supplement: Supplementary file 11 — Appendix Figure Source Data [file 44318_2025_508_MOESM11_ESM.zip › Source data Appendix Figure/Appendix Figure S9/Appendix Figure S9A/Appendix Figure S9A.pptx]

## Slide 1
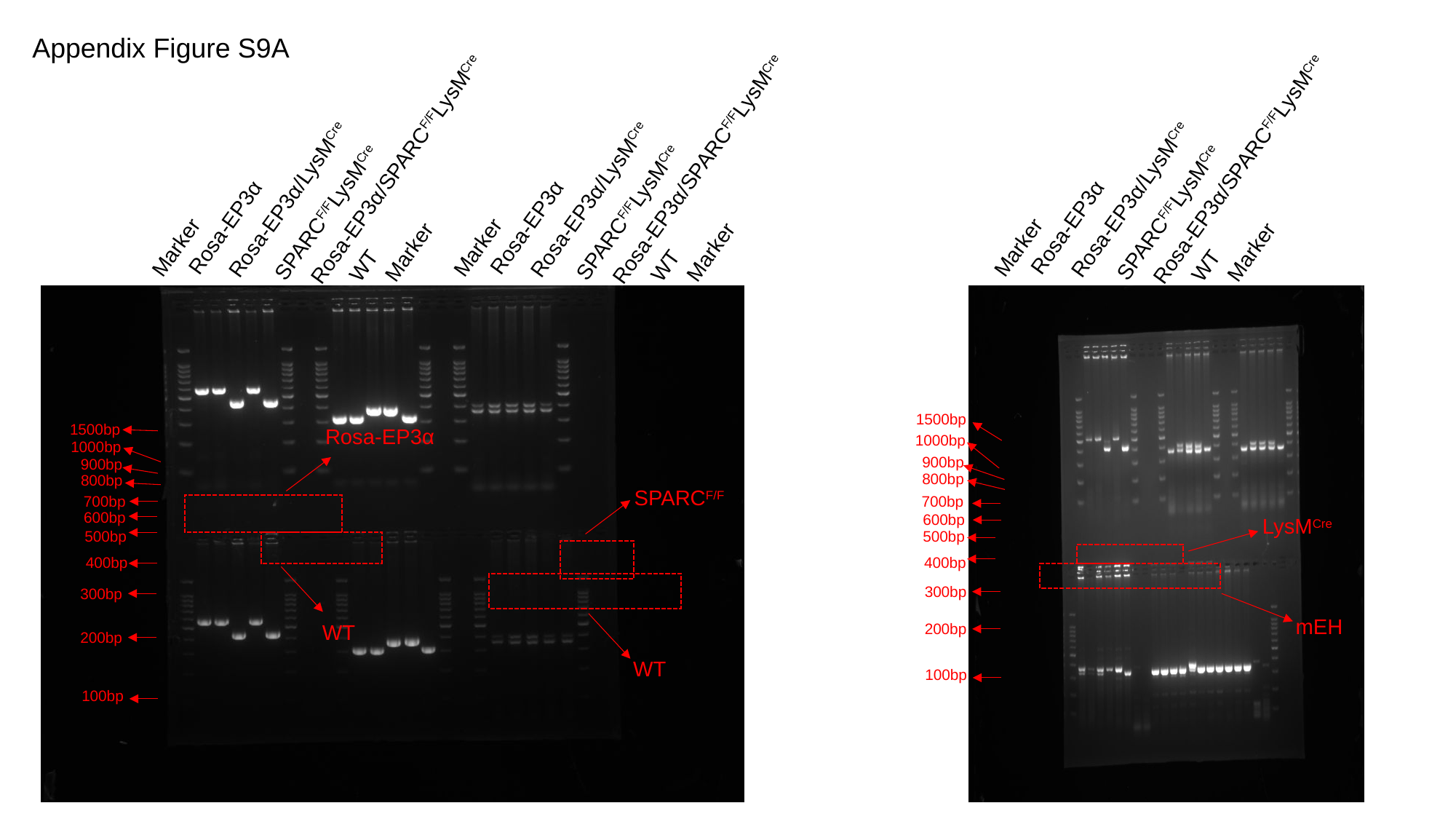

Rosa-EP3α/SPARCF/FLysMCre
Rosa-EP3α/LysMCre
Rosa-EP3α
SPARCF/FLysMCre
Marker
Marker
WT
Rosa-EP3α/SPARCF/FLysMCre
Rosa-EP3α/LysMCre
Rosa-EP3α
SPARCF/FLysMCre
Marker
Marker
WT
Rosa-EP3α/SPARCF/FLysMCre
Rosa-EP3α/LysMCre
Rosa-EP3α
SPARCF/FLysMCre
Marker
Marker
WT
Appendix Figure S9A
1500bp
1500bp
1000bp
900bp
800bp
700bp
600bp
500bp
400bp
300bp
200bp
100bp
Rosa-EP3α
1000bp
900bp
800bp
SPARCF/F
700bp
600bp
LysMCre
500bp
400bp
300bp
mEH
WT
200bp
WT
100bp
