# Supplementary figures and images for: Deletion of EP3 prostaglandin receptor in murine macrophages aggravates diet-induced obesity by suppressing SPARC (part 2 of 2)
Source: EMBO J. 2025 Jul 23;44(18):4962–83. doi: 10.1038/s44318-025-00508-y (PMC12436609; doi:10.1038/s44318-025-00508-y)

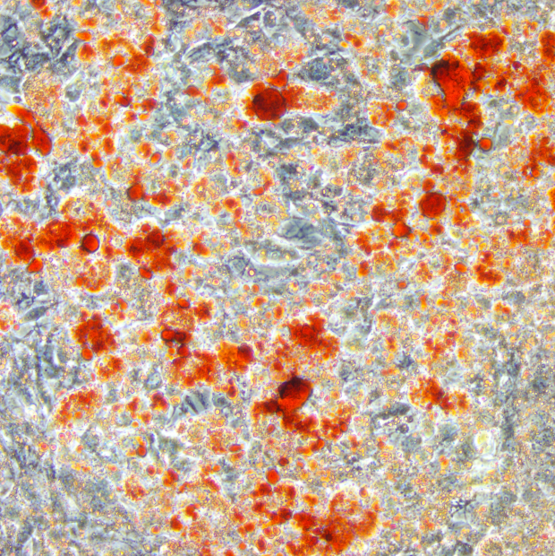

Supplement: Supplementary file 11 — Appendix Figure Source Data [file 44318_2025_508_MOESM11_ESM.zip › Source data Appendix Figure/Appendix Figure S2/Appendix Figure S2C/L-198106-Veh.tif]

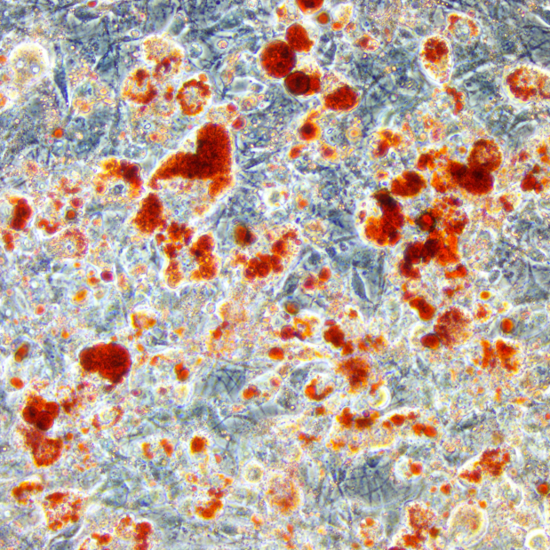

Supplement: Supplementary file 11 — Appendix Figure Source Data [file 44318_2025_508_MOESM11_ESM.zip › Source data Appendix Figure/Appendix Figure S2/Appendix Figure S2C/L-798106-Sul.tif]

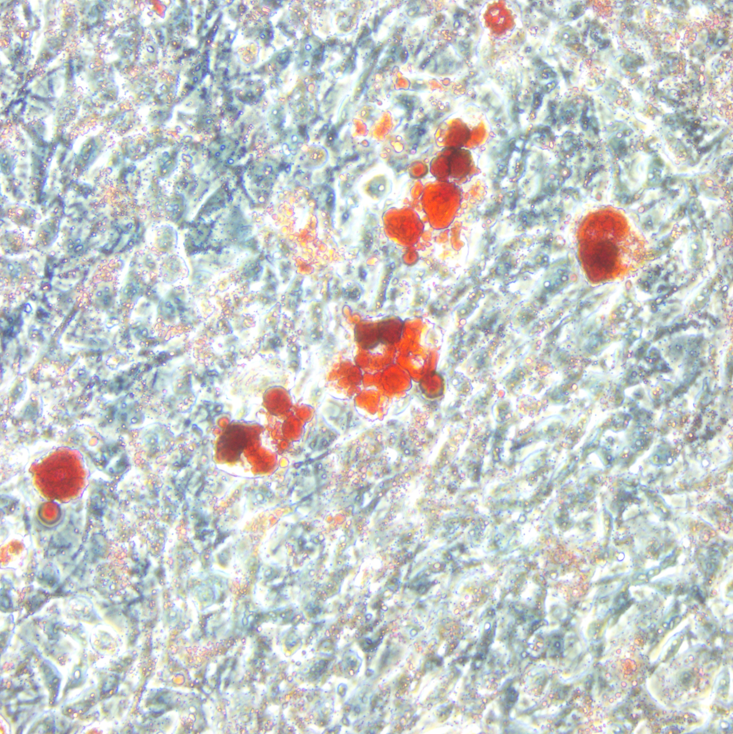

Supplement: Supplementary file 11 — Appendix Figure Source Data [file 44318_2025_508_MOESM11_ESM.zip › Source data Appendix Figure/Appendix Figure S2/Appendix Figure S2C/Veh-Sul.tif]

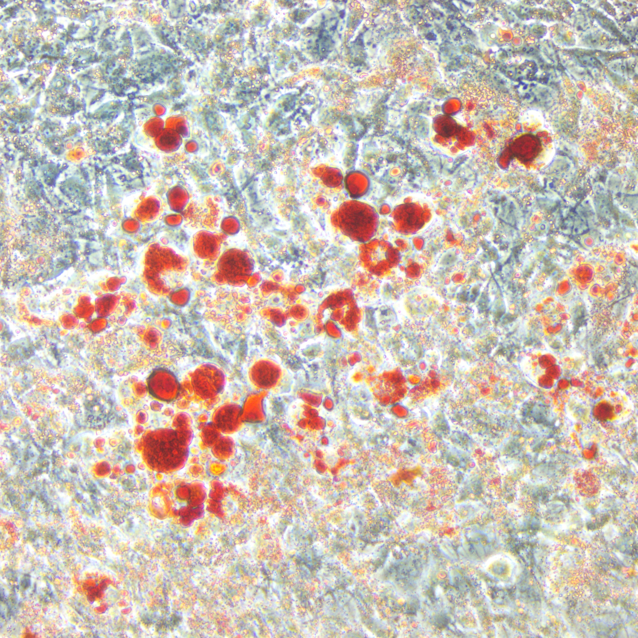

Supplement: Supplementary file 11 — Appendix Figure Source Data [file 44318_2025_508_MOESM11_ESM.zip › Source data Appendix Figure/Appendix Figure S2/Appendix Figure S2C/Veh-Veh.tif]

Appendix Figure S3C

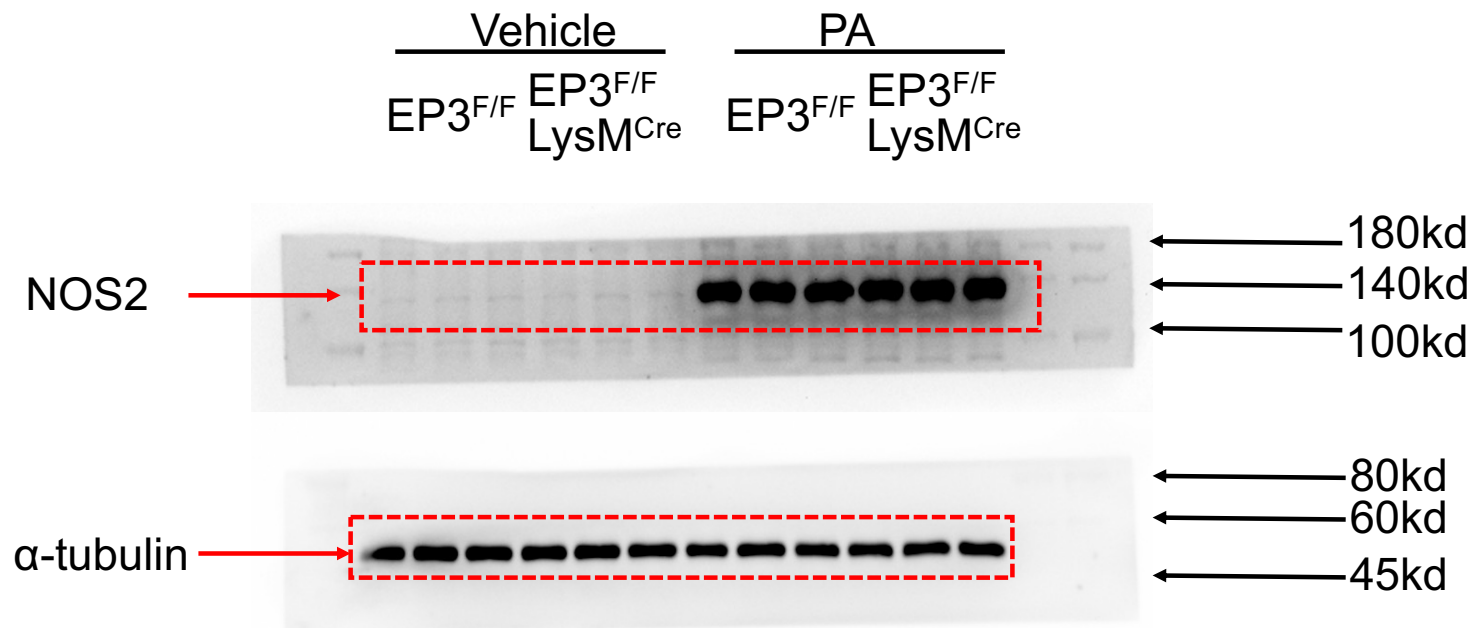

Supplement: Supplementary file 11 — Appendix Figure Source Data [file 44318_2025_508_MOESM11_ESM.zip › Source data Appendix Figure/Appendix Figure S3/Appendix Figure S3C/Appendix Figure S3C.pdf]

Appendix Figure S3C

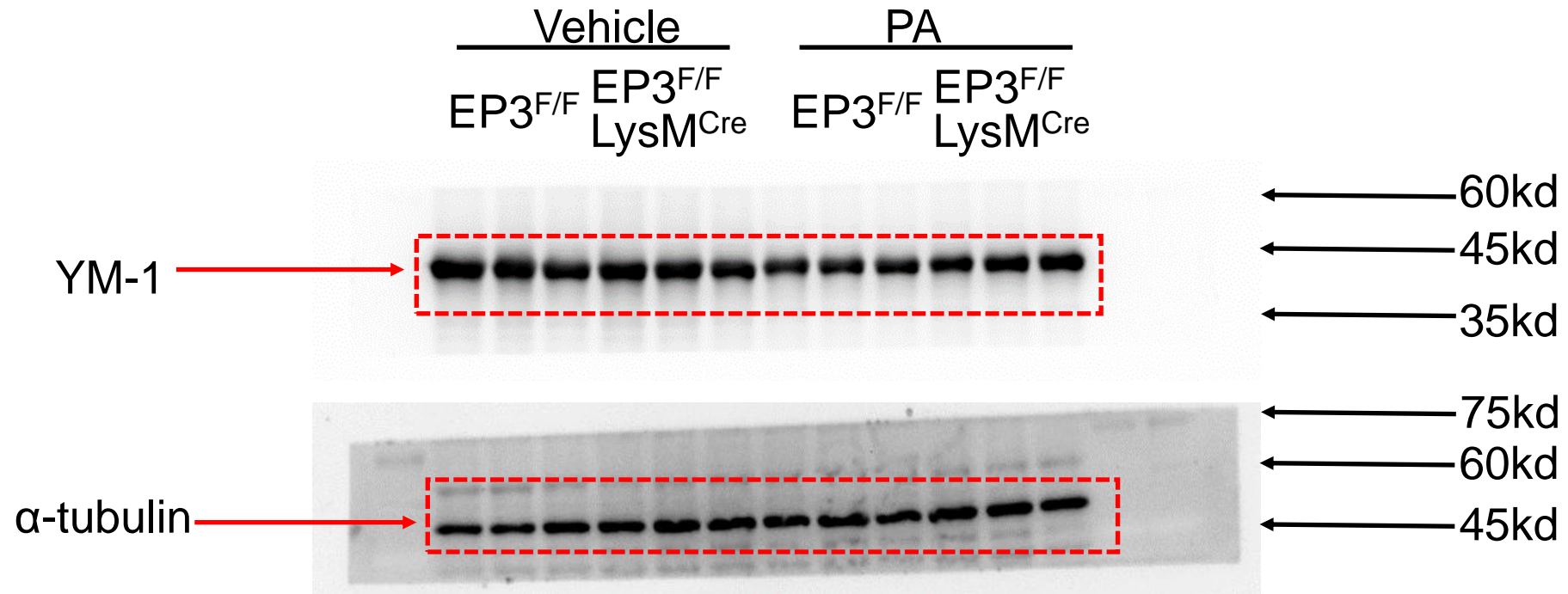

Supplement: Supplementary file 11 — Appendix Figure Source Data [file 44318_2025_508_MOESM11_ESM.zip › Source data Appendix Figure/Appendix Figure S3/Appendix Figure S3E/Appendix Figure S3E.pdf]

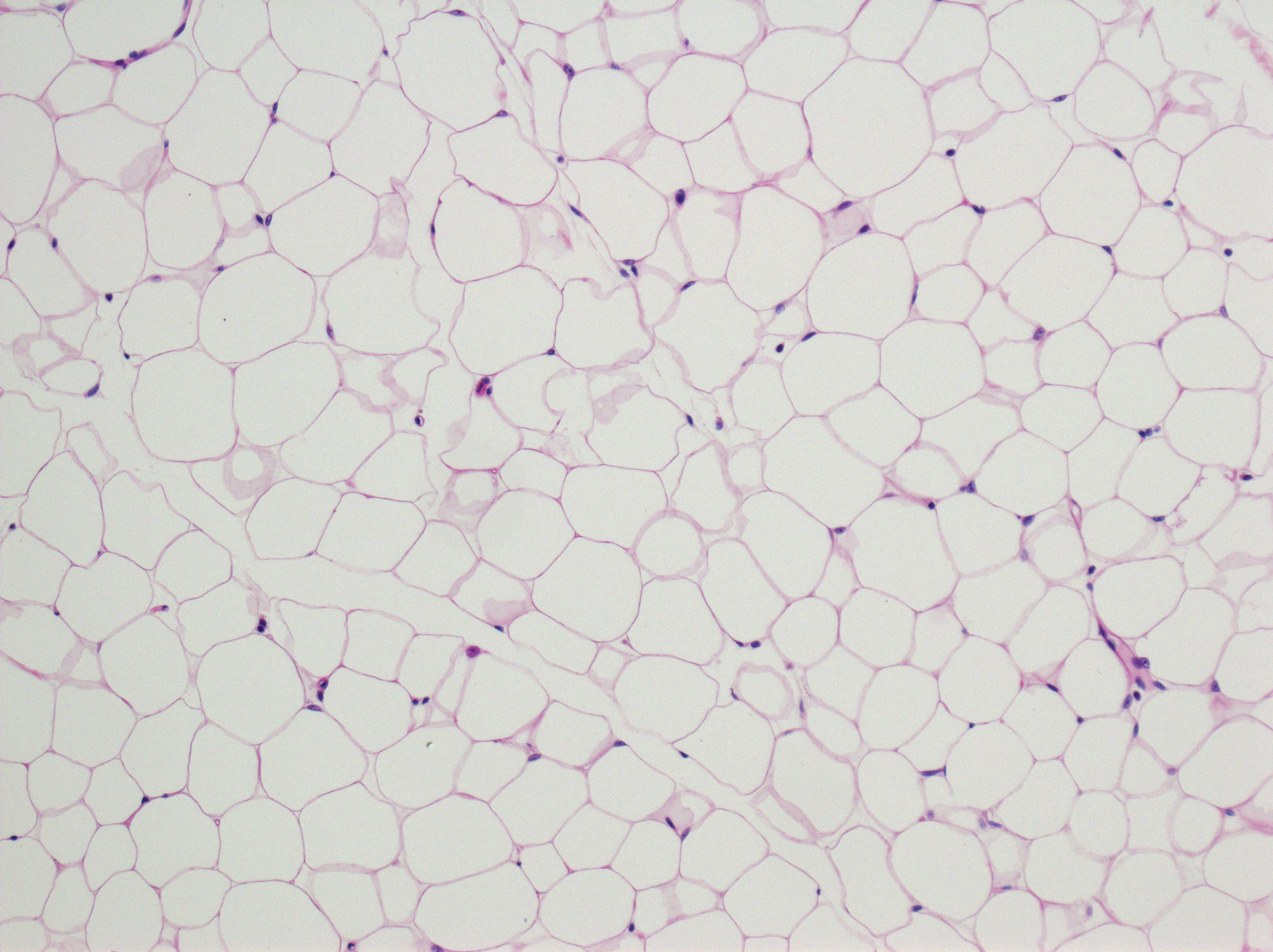

Supplement: Supplementary file 11 — Appendix Figure Source Data [file 44318_2025_508_MOESM11_ESM.zip › Source data Appendix Figure/Appendix Figure S4/Appendix Figure S4G/eWAT-EP3Flox.tif]

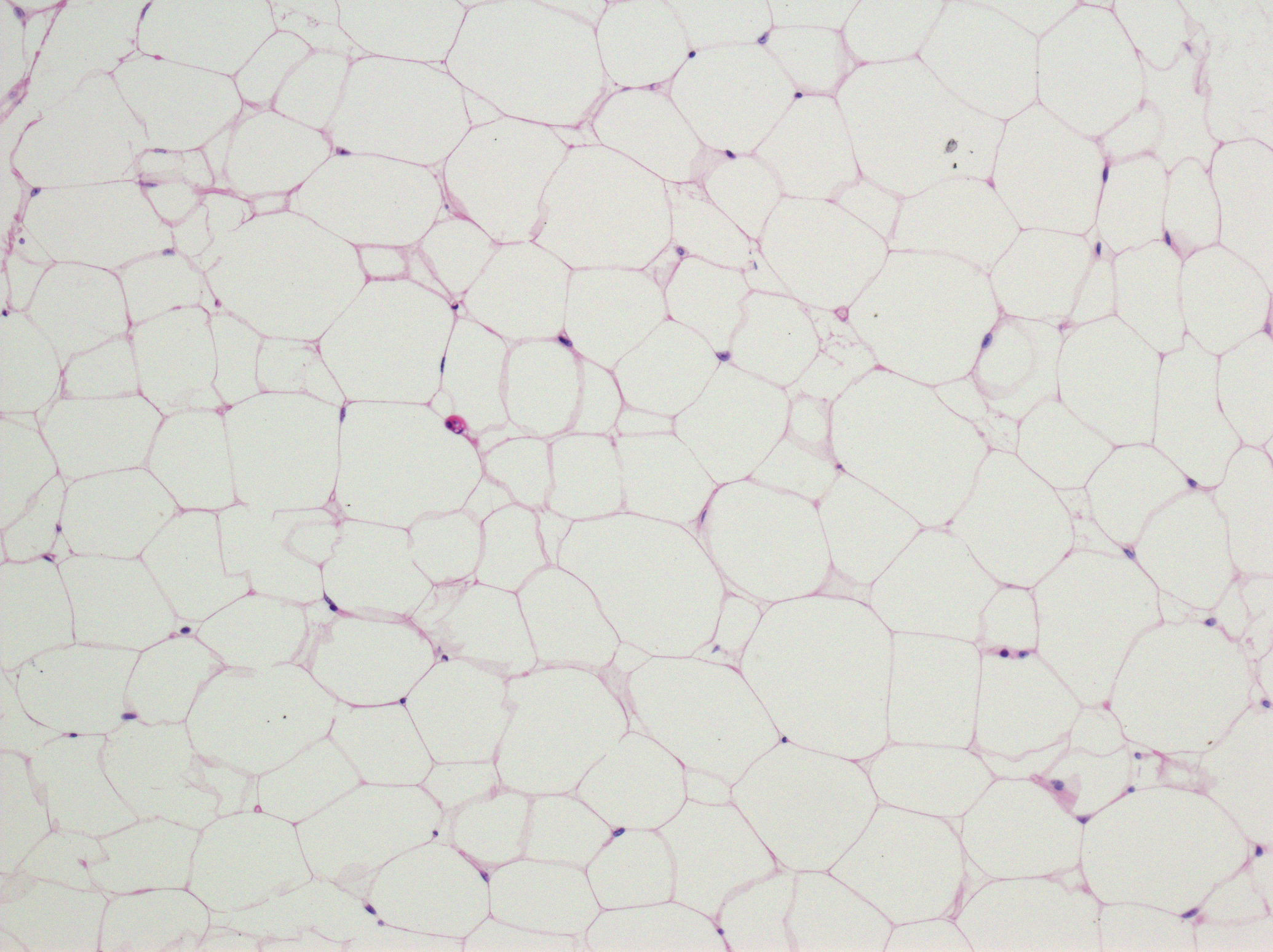

Supplement: Supplementary file 11 — Appendix Figure Source Data [file 44318_2025_508_MOESM11_ESM.zip › Source data Appendix Figure/Appendix Figure S4/Appendix Figure S4G/eWAT-EP3FloxLysMCre.tif]

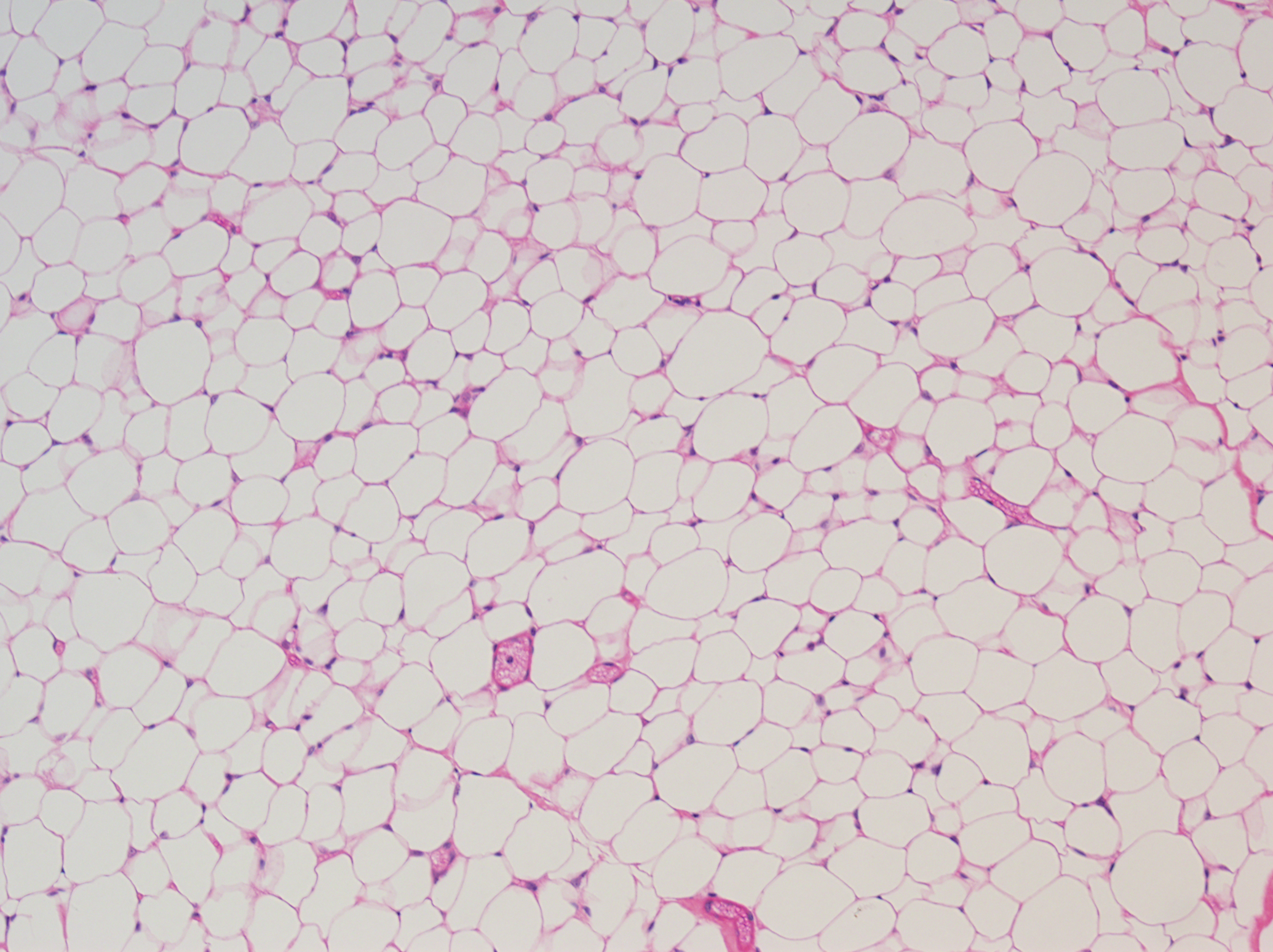

Supplement: Supplementary file 11 — Appendix Figure Source Data [file 44318_2025_508_MOESM11_ESM.zip › Source data Appendix Figure/Appendix Figure S4/Appendix Figure S4G/iWAT-EP3Flox.tif]

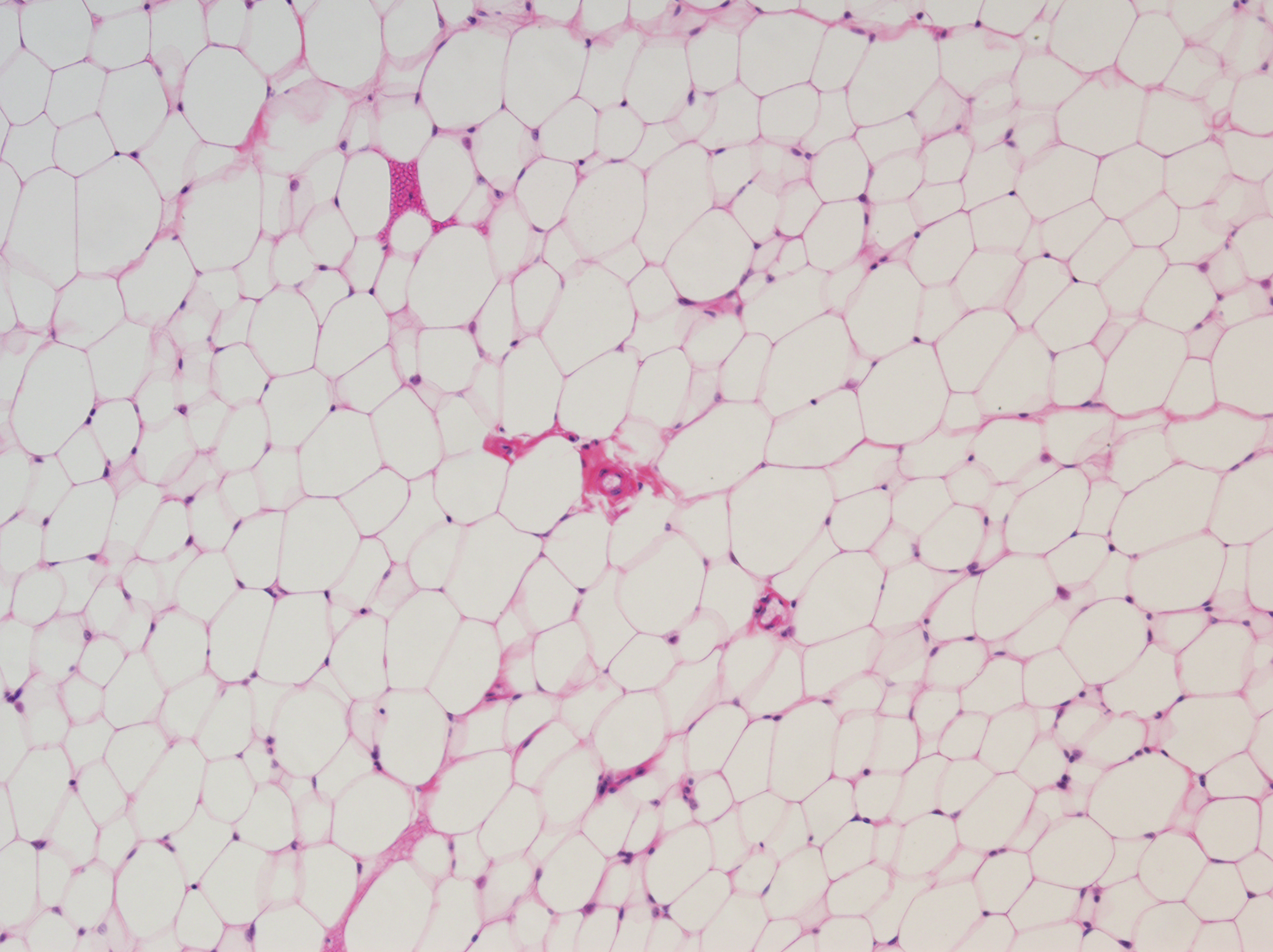

Supplement: Supplementary file 11 — Appendix Figure Source Data [file 44318_2025_508_MOESM11_ESM.zip › Source data Appendix Figure/Appendix Figure S4/Appendix Figure S4G/iWAT-EP3FloxLysMCre.tif]

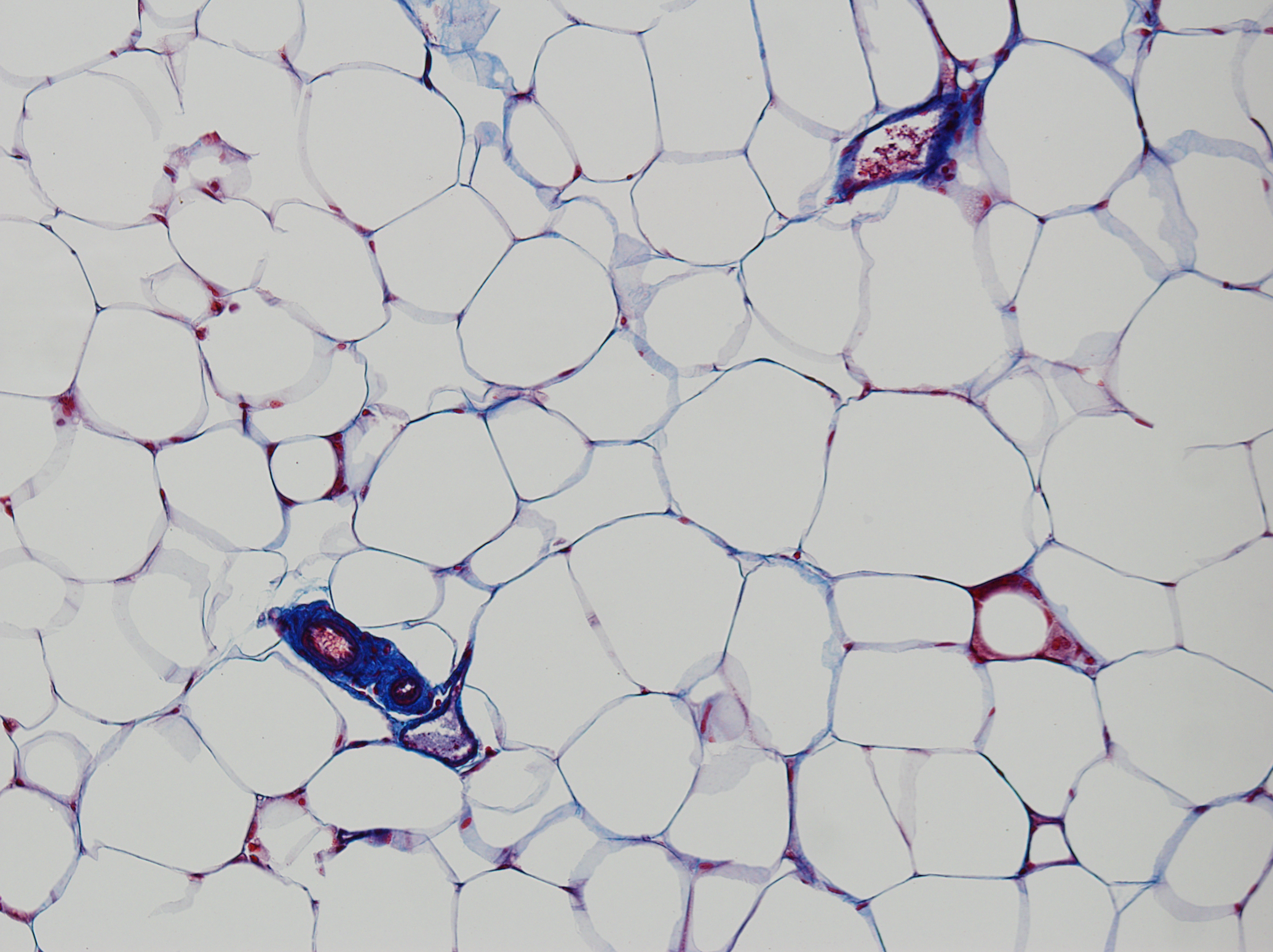

Supplement: Supplementary file 11 — Appendix Figure Source Data [file 44318_2025_508_MOESM11_ESM.zip › Source data Appendix Figure/Appendix Figure S6/Appendix Figure S6F/HFD-EP3Flox.tif]

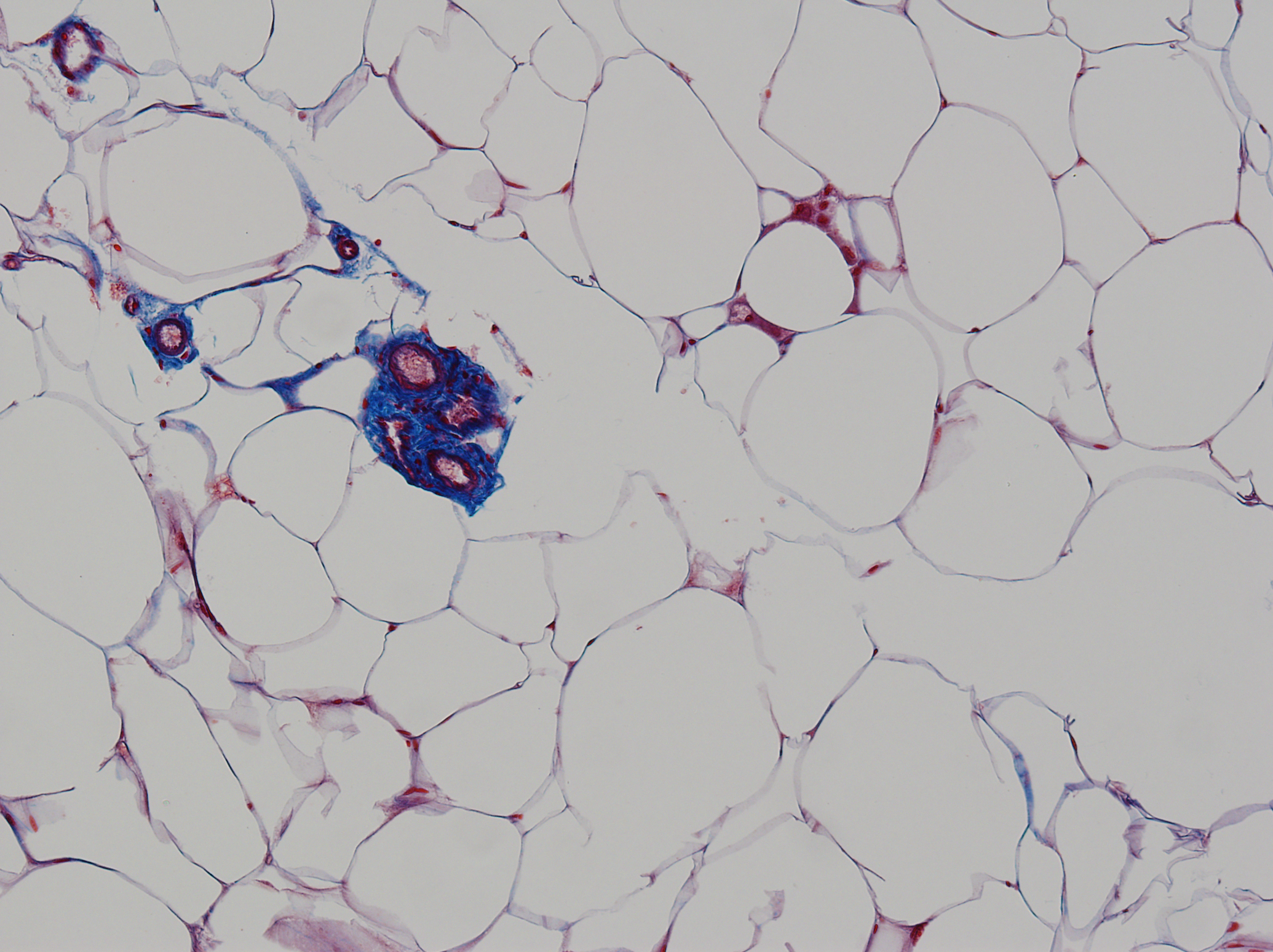

Supplement: Supplementary file 11 — Appendix Figure Source Data [file 44318_2025_508_MOESM11_ESM.zip › Source data Appendix Figure/Appendix Figure S6/Appendix Figure S6F/HFD-EP3FloxLysMCre.tif]

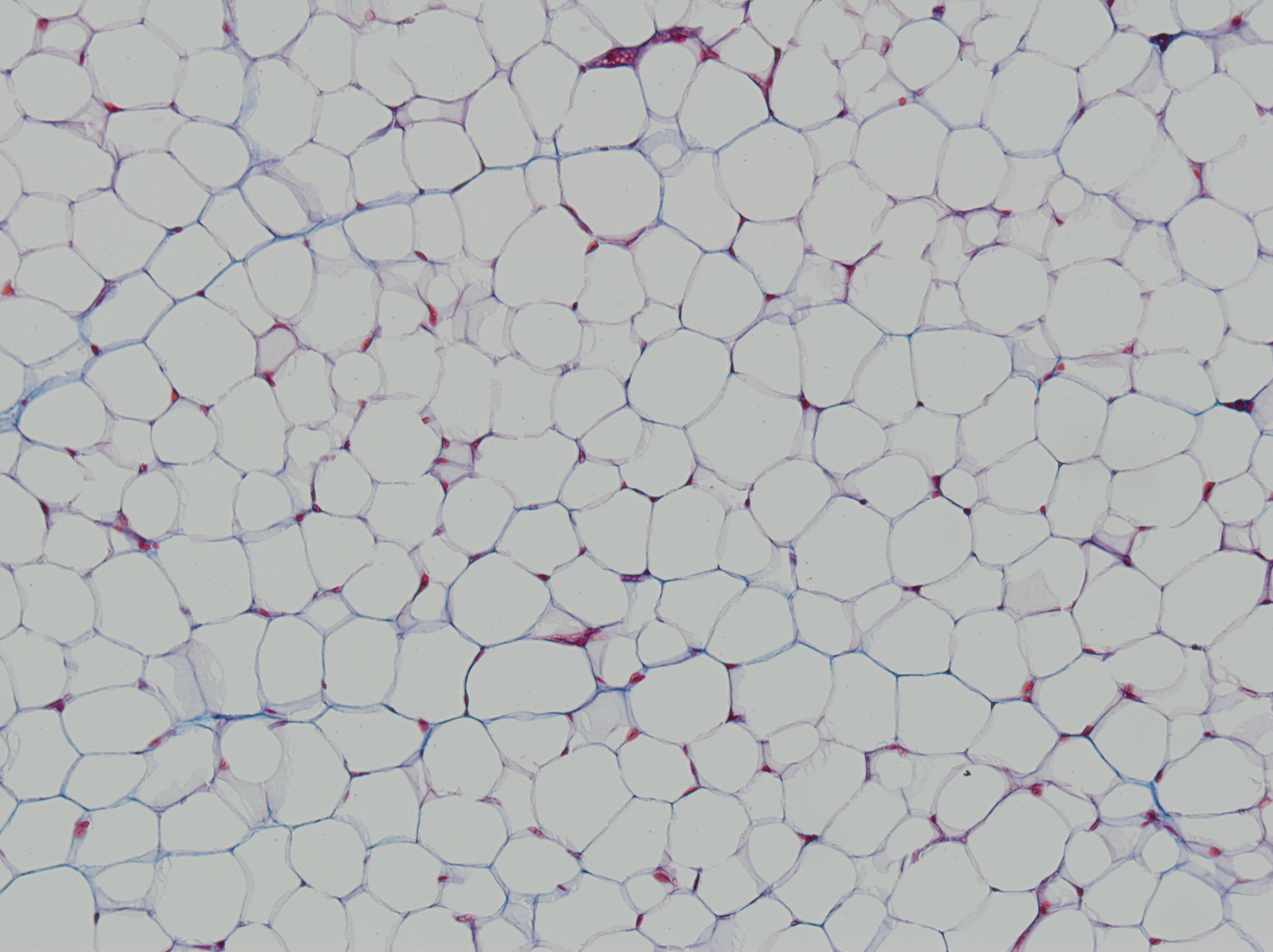

Supplement: Supplementary file 11 — Appendix Figure Source Data [file 44318_2025_508_MOESM11_ESM.zip › Source data Appendix Figure/Appendix Figure S6/Appendix Figure S6F/NCD-EP3Flox.tif]

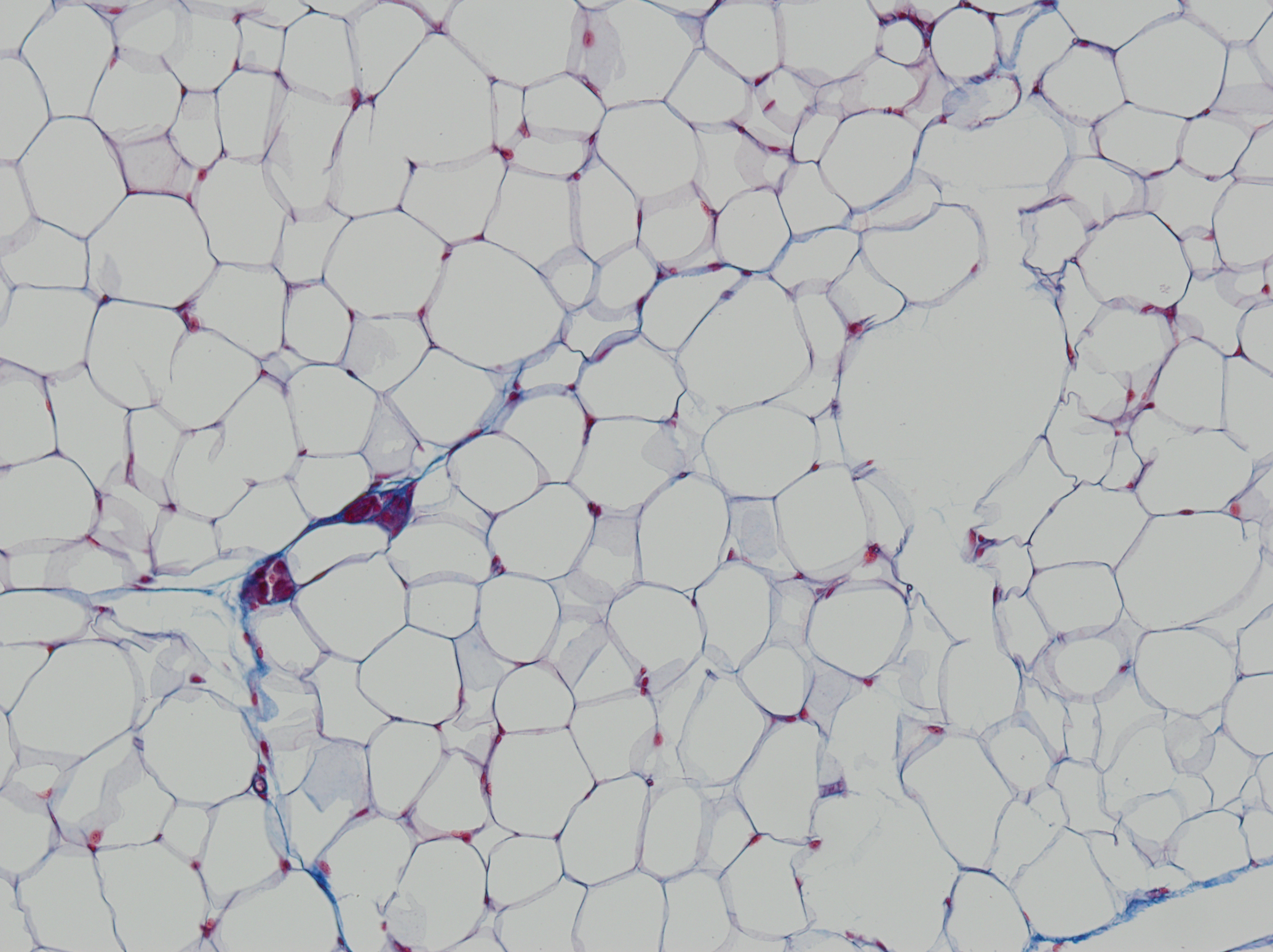

Supplement: Supplementary file 11 — Appendix Figure Source Data [file 44318_2025_508_MOESM11_ESM.zip › Source data Appendix Figure/Appendix Figure S6/Appendix Figure S6F/NCD-EP3FloxLysMCre.tif]

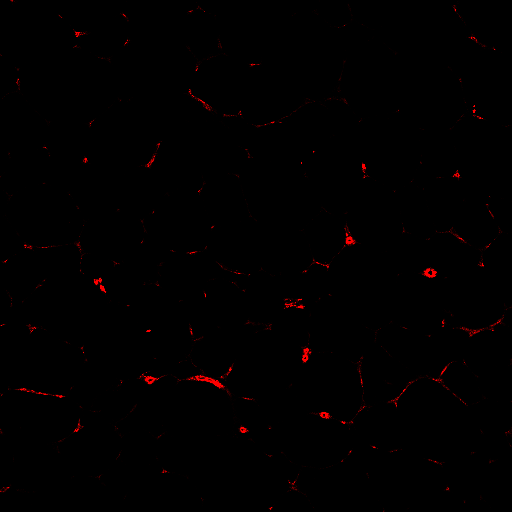

Supplement: Supplementary file 11 — Appendix Figure Source Data [file 44318_2025_508_MOESM11_ESM.zip › Source data Appendix Figure/Appendix Figure S7/Appendix Figure S7B/EP3Flox/EP3Flox-CD31.tif]

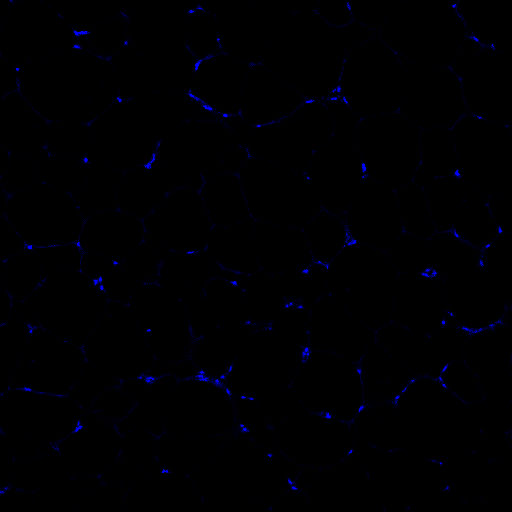

Supplement: Supplementary file 11 — Appendix Figure Source Data [file 44318_2025_508_MOESM11_ESM.zip › Source data Appendix Figure/Appendix Figure S7/Appendix Figure S7B/EP3Flox/EP3Flox-DAPI.tif]

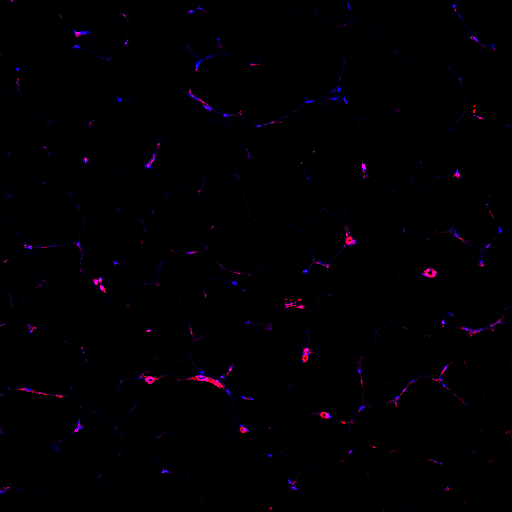

Supplement: Supplementary file 11 — Appendix Figure Source Data [file 44318_2025_508_MOESM11_ESM.zip › Source data Appendix Figure/Appendix Figure S7/Appendix Figure S7B/EP3Flox/EP3Flox-Merge.tif]

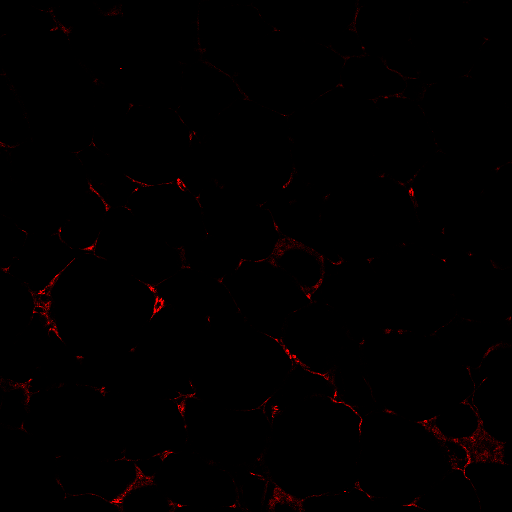

Supplement: Supplementary file 11 — Appendix Figure Source Data [file 44318_2025_508_MOESM11_ESM.zip › Source data Appendix Figure/Appendix Figure S7/Appendix Figure S7B/EP3FloxLysMCre/EP3FloxLysMCre-CD31.tif]

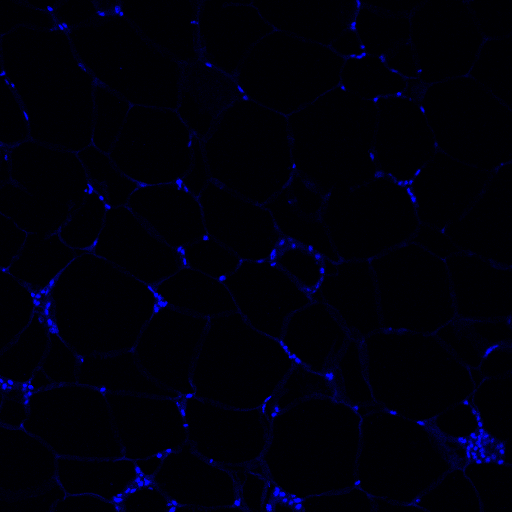

Supplement: Supplementary file 11 — Appendix Figure Source Data [file 44318_2025_508_MOESM11_ESM.zip › Source data Appendix Figure/Appendix Figure S7/Appendix Figure S7B/EP3FloxLysMCre/EP3FloxLysMCre-DAPI.tif]

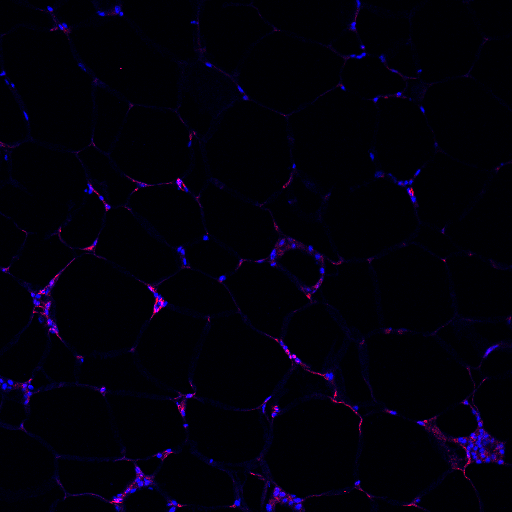

Supplement: Supplementary file 11 — Appendix Figure Source Data [file 44318_2025_508_MOESM11_ESM.zip › Source data Appendix Figure/Appendix Figure S7/Appendix Figure S7B/EP3FloxLysMCre/EP3FloxLysMCre-Merge.tif]

Appendix Figure S9A

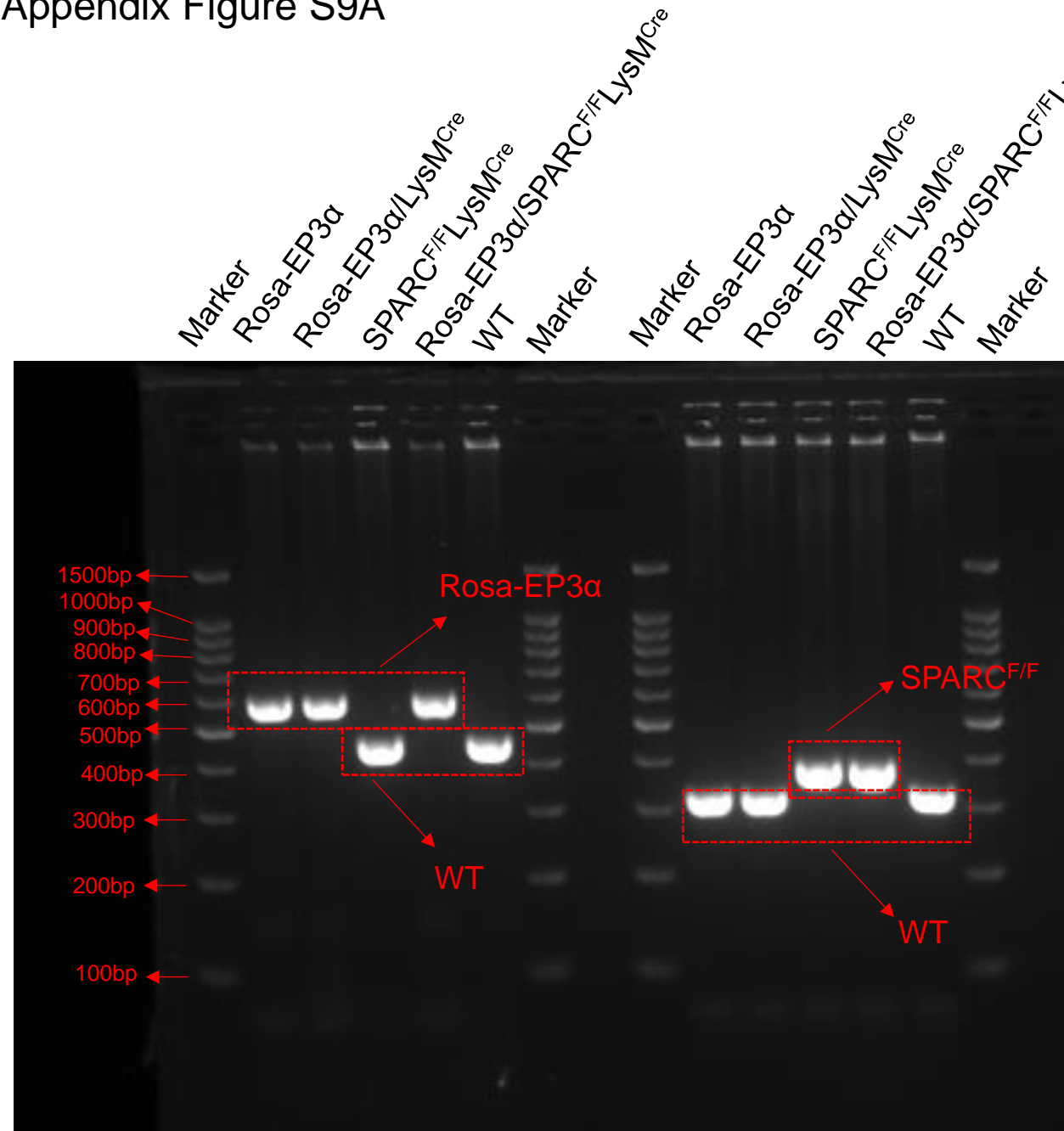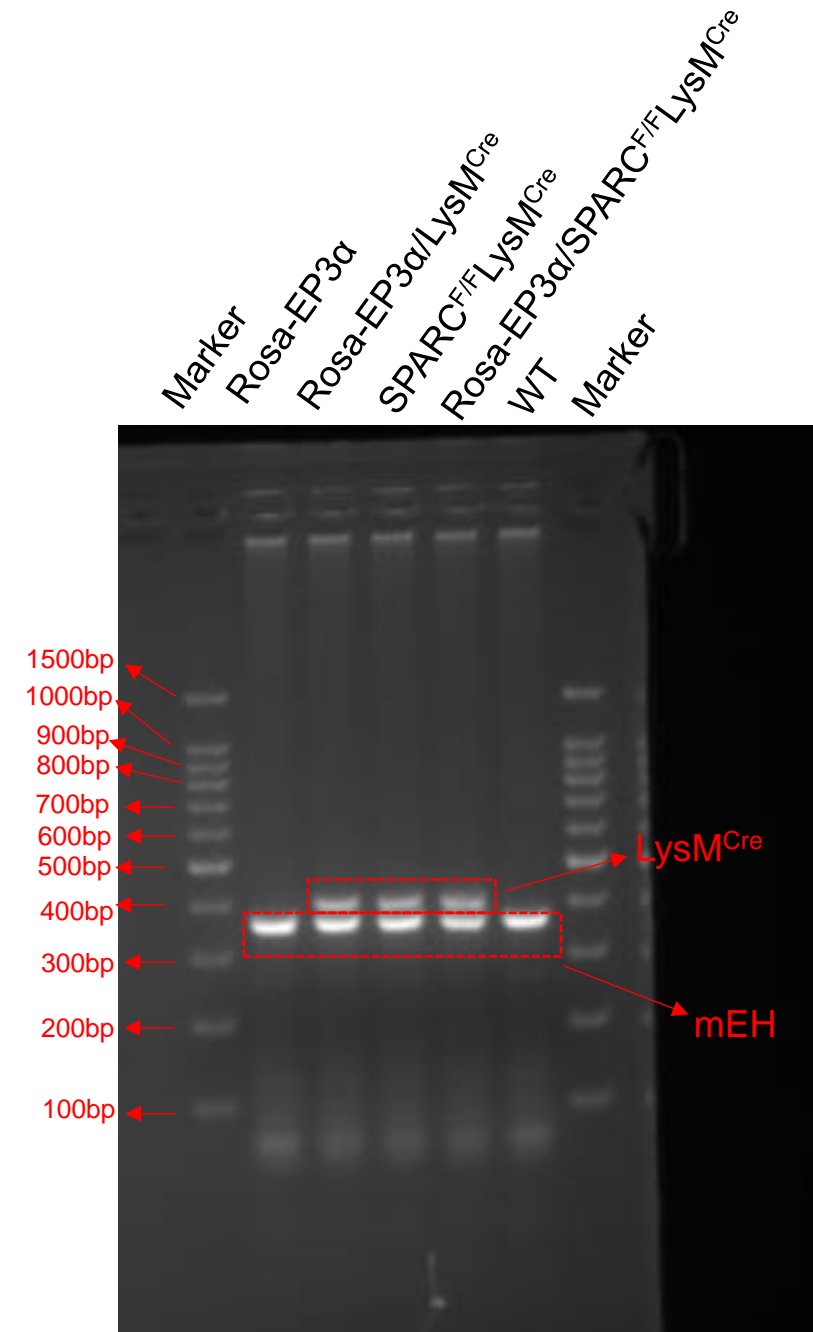

Supplement: Supplementary file 11 — Appendix Figure Source Data [file 44318_2025_508_MOESM11_ESM.zip › Source data Appendix Figure/Appendix Figure S9/Appendix Figure S9A/Appendix Figure S9A.pdf]
